# Supplementary material for: Discovery of diarylpyrimidine derivatives bearing piperazine sulfonyl as potent HIV-1 nonnucleoside reverse transcriptase inhibitors
Source: Commun Chem. 2023 Apr 29;6:83. doi: 10.1038/s42004-023-00888-4 (PMC10148624; doi:10.1038/s42004-023-00888-4)
Supplement: Supplementary file 2 — Supplementary Information [file 42004_2023_888_MOESM2_ESM.pdf]

Supporting Information for

Original article

Discovery of diarylpyrimidine derivatives bearing piperazine sulfonyl as potent HIV-1 nonnucleoside reverse transcriptase inhibitors

Xiangyi Jiang[1], Boshi Huang[1], Shawn Rumrill[2,3], David Pople[2,3], Waleed A Zalloum[4], Dongwei Kang[1,5], Fabao Zhao[1], Xiangkai Ji[1], Zhen Gao[1], Lide Hu[1], Zhao Wang[1], Minghui Xie[1], Erik De Clercq[6], Francesc X. Ruiz[2,3,*], Eddy Arnold[2,3,*], Christophe Pannecouque[6, *], Xinyong Liu[1, 5, *], and Peng Zhan[1, 5, *]

[1] Department of Medicinal Chemistry, Key Laboratory of Chemical Biology (Ministry of Education), School of Pharmaceutical Sciences, Cheeloo College of Medicine, Shandong University, 44 West Culture Road, 250012 Jinan, Shandong, PR China

[2] Center for Advanced Biotechnology and Medicine, Rutgers University, Piscataway, New Jersey 08854, United States

[3] Department of Chemistry and Chemical Biology, Rutgers University, Piscataway, New Jersey 08854, United States

[4] Department of Pharmacy, Faculty of Health Science, American University of Madaba, P.O Box 2882, Amman, 11821, Jordan

[5] China-Belgium Collaborative Research Center for Innovative Antiviral Drugs of Shandong Province, 44 West Culture Road, 250012, Jinan, Shandong, PR China

[6] Rega Institute for Medical Research, Laboratory of Virology and Chemotherapy, K.U.Leuven, Herestraat 49 Postbus 1043 (09.A097), B-3000 Leuven, Belgium

Supplementary Methods

Section 1. Molecular simulation studies 3

Section 2. Syhthetic procedures 3

Section 3. *In vitro* anti-HIV assays 19

Section 4. HIV-1 RT inhibition assays 20

Section 5. HIV-1 RT Crystallization and Structure Determination 21

Section 6. Molecular dynamics simulation methods 23

Section 7. Water solubility measurements 27

Section 8. CYP enzyme inhibition assay 27

Section 9. Pharmacokinetics studies 28

Supplementary Methods

Section 1. Molecular simulation studies

The molecular modelling study was performed by Sybyl-X 2.0 software. All the molecules were built using standard bond lengths and angles from Sybyl-X 2.0/Base Builder and optimized using the Tripos force field for 1000 generations, until the minimized conformers of the ligand were the same. The flexible docking method (Surflex-Dock) docks the ligand automatically into the ligand-binding site of the receptor with a protocol-based approach and an empirically derived scoring function. The protocol is a computational representation of a putative ligand that binds to the intended binding site and is a unique and essential element of the docking algorithm. The scoring function in Surflex-Dock, containing hydrophobic, polar, repulsive, entropic, and solvation terms, was trained to estimate the dissociation constant (K_d). The protein was prepared by removing the ligand and other unnecessary small molecules from the cocrystal structures (PDB code: 3MEC), polar hydrogen atoms and charges were added to the protein before docking. During the docking procedure, all of the single bonds in residue sidechains inside the defined RT binding pocket were regarded as rotatable or flexible, and the ligand was allowed to rotate at all single bonds and to move flexibly within the tentative binding pocket. The atomic charges were recalculated using the Kollman all-atom approach for the protein and the Gasteiger-Hückel approach for the ligand. The binding interaction energy was calculated, including Van der Waals, electrostatic, and torsional energy terms defined in the Tripos force field. The structure optimization was performed for 10,000 generations using a genetic algorithm, and the 20-best-scoring ligand-protein complexes were kept for further analysis. The -log (Kd)[2] values of the 20-best-scoring complexes, representing the binding affinities of ligand with RT, encompassed a wide range of functional classes (10^{-2}-10^{-9}). The highest-scoring 3D structural model of ligand-bound RT was chosen to define the binding interaction[1].

Section 2. Syhthetic procedures

The melting point (mp) datas of target compounds were measured on a micro melting

point apparatus and were uncorrected. ^{1}H NMR and ^{13}C NMR spectra were determined in DMSO-d_6 on a Bruker Avance-400 NMR spectrometer or a Bruker Avance-600 NMR spectrometer, and TMS was used as an internal standard. Chemical shifts and coupling constants were expressed in δ units (ppm) and *J* values (Hz) respectively. The mass spectra (MS) datas were measured *via* an electrospray ionization by an LC Autosampler Device: Standard G1313A instrument. Thin-layer chromatography (TLC) was used to detect reactions on Silica Gel GF254 and spots were visualized by irradiation with UV light (λ = 254 nm). The flash column chromatography was used for the purification of compounds on column packed with Silica Gel 60 (200 - 300 mesh). Reagent-grade solvents were applied, and purified *via* standard methods if necessary. The reaction solutions were concentrated through a rotary evaporator under reduced pressure condition (EYELA N-1300D). The purity of final compounds was > 95%, which was analyzed by a high performance liquid chromatography (HPLC) system (Shimadzu SPD-20A/20AV).

General procedure for the synthesis of intermediates 8a-c. To a solution of 1-Boc-piperazine (1.86 g, 0.01 mol) and 2-bromo-5-nitropyridine (2.03 g, 0.01 mol), or 2-bromo-5-nitrobenzonitrile (2.27 g, 0.01 mol), or 1-bromo-4-nitro-2-(trifluoromethyl)benzene (2.70 g, 0.01 mol) in dimethylformamide (20 mL), triethylamine (1.21 g, 0.012 mol) was added. The resulting mixture was stirred at 120°C for 8 h. After cooling to room temperature, water (50 mL) was added to the reaction solution, and the mixture was extracted with ethyl acetate (3 × 15 mL). Next, the organic layer was washed with saturated sodium chloride solution (3 × 25 mL), and dried by anhydrous sodium sulfate. After distilling the solvent under reduced pressure, the corresponding intermediates **8a-c** were recrystallized using ethyl acetate/petroleum ether.

*tert-butyl 4-(5-nitropyridin-2-yl)piperazine-1-carboxylate (**8a**).* Yellow solid, yield: 93.4%. ^{1}H NMR (600 MHz, DMSO-d_6) δ 8.97 (d, *J* = 2.8 Hz, 1H, pyrimidinyl-H), 8.24 (dd, *J* = 9.6, 2.8 Hz, 1H, pyrimidinyl-H), 6.93 (d, *J* = 9.6 Hz, 1H, pyrimidinyl-H), 3.87 – 3.70 (m, 4H, pyrimidinyl-H), 3.53 – 3.38 (m, 4H, pyrimidinyl-H), 1.43 (s, 9H, CH_3 × 3). ESI-MS: 309.4 m/z (M + H) $^+$, $C_{14}H_{20}N_4O_4$ (308.15).

*tert-butyl 4-(4-nitro-2-(trifluoromethyl)phenyl)piperazine-1-carboxylate (**8b**).* Yellow solid, yield: 89.2%. ^{1}H NMR (600 MHz, DMSO-d_6) δ 8.43 (dd, *J* = 9.0, 2.7 Hz, 1H, Ph-H), 8.40 (d, *J* = 2.7 Hz, 1H, Ph-H), 7.61 (d, *J* = 9.0 Hz, 1H, Ph-H), 3.47 (s, 4H, pyrimidinyl-H), 3.10 – 3.02 (m, 4H, pyrimidinyl-H), 1.43 (s, 9H, CH_3 × 3). ESI-MS: 376.3 m/z (M + H) $^+$, $C_{16}H_{20}F_3N_3O_4$ (375.14).

*tert-butyl 4-(2-cyano-4-nitrophenyl)piperazine-1-carboxylate (**8c**).* Yellow solid, yield: 91.3%. ^{1}H NMR (600 MHz, DMSO-d_6) δ 8.55 (d, *J* = 2.8 Hz, 1H, Ph-H), 8.31 (dd, *J* = 9.4, 2.8 Hz, 1H, Ph-H), 7.25 (d, *J* = 9.4 Hz, 1H, Ph-H), 3.60 – 3.46 (m, 8H, pyrimidinyl-H), 1.43 (s, 9H, CH_3 × 3). ESI-MS: 355.29 m/z (M + Na) $^+$, $C_{16}H_{20}N_4O_4$ (332.15).

General procedure for the synthesis of intermediates 9a-c. Compounds **8a-c** (1.0 eq) was dissolved in 20 mL tetrahydrofuran, then 10% Pd/C (0.1 eq) was added. The reaction mixture was degassed under a hydrogen atmosphere, and stirred at room temperature for 4 h. Then the mixture was filtered and concentrated, and recrystallized using ethyl acetate/petroleum ether to obtain the compounds **9a-c**.

General procedure for the synthesis of intermediates 11-12. 2,4-dichloropyrimidine **10** (2.98 g, 0.02 mol) and potassium carbonate (3.32 g, 0.024 mol) were dissolved in dimethylformamide (35 mL), and 4-hydroxy-3,5-dimethylbenzonitrile (2.94 g, 0.02 mol) or (*E*)-3-(4-hydroxy-3,5-dimethylphenyl) acrylonitrile (3.46 g, 0.02 mol) was added. The reaction mixture was stirred at 45 °C for 6 h until completion monitored by TLC. Subsequently, the mixture was diluted with 200 mL water, and finally recrystallized using dimethylformamide and water to produce the intermediates **11-12**.

*4-((2-chloropyrimidin-4-yl)oxy)-3,5-dimethylbenzonitrile (**11**).* White solid, yield: 85.3%. ^{1}H NMR (400 MHz, DMSO-d_6) δ 8.70 (d, *J* = 5.7 Hz, 1H, pyrimidinyl-H), 7.75 (s, 2H, Ph-H), 7.32 (d, *J* = 5.7 Hz, 1H, pyrimidinyl-H), 2.10 (s, 6H, CH_3 × 2). ESI-MS: m/z 259.97 (M + H) $^+$, $C_{13}H_{10}ClN_3O$ (259.05).

*(E)-3-(4-((2-chloropyrimidin-4-yl)oxy)-3,5-dimethylphenyl)acrylonitrile (**12**).* White solid, yield: 86.5%. ^{1}H NMR (400 MHz, DMSO-d_6) δ 8.67 (d, *J* = 5.7 Hz, 1H, pyrimidinyl-H), 7.62 (d, *J* = 16.7 Hz, 1H, CH=), 7.52 (s, 2H, Ph-H), 7.25 (d, *J* = 5.7

Hz, 1H, pyrimidinyl-H), 6.45 (d, *J* = 16.7 Hz, 1H, CH=), 2.07 (s, 6H, CH_3 × 2). ESI-MS: m/z 286.3 (M + H) $^+$, $C_{15}H_{12}ClN_3O$ (285.07).

General procedure for the synthesis of intermediates 13(a-c) and 14(a-c). The intermediates **11** or **12** (1.0 eq), well-prepared **9a-c** (1.0 eq), palladium acetate (0.05 eq), xantphos (0.05 eq), and cesium carbonate (1.5 eq) were dissolved in 1,4-dioxane (12 mL). The reaction mixture was degassed under a nitrogen atmosphere, and stirred at 90°C for 8 h. After cooling to room temperature, the reaction mixture was filtered and concentrated under reduced pressure. The residue was finally purified by column chromatography with methanol/dichloromethane (1:50) as eluent to gain corresponding intermediates **13(a-c) and 14(a-c)**.

*tert-butyl 4-(5-((4-(4-cyano-2,6-dimethylphenoxy)pyrimidin-2-yl)amino)pyridin-2-yl)piperazine-1-carboxylate **(13a)**.* White solid, yield: 55.3%. ^1H NMR (400 MHz, DMSO-d_6) δ 9.35 (s, 1H, NH), 8.34 (d, *J* = 5.4 Hz, 1H, pyrimidinyl-H), 8.08 (s, 1H, pyridinyl-H), 7.72 (s, 2H, PhH), 7.48 (s, 1H, pyridinyl-H), 6.60 (s, 1H, pyridinyl-H), 6.48 (d, *J* = 5.3 Hz, 1H, pyrimidinyl-H), 3.40 (s, 4H, piperazinyl-4H), 3.36 (s, 4H, piperazinyl-4H), 2.10 (s, 6H, CH_3 × 2), 1.42 (s, 9H, CH_3 × 3). ESI-MS: m/z 500.27 (M - H) $^-$, $C_{27}H_{31}N_7O_3$ (501.59).

*tert-butyl (E)-4-(5-((4-(4-(2-cyanovinyl)-2,6-dimethylphenoxy)pyrimidin-2-yl)amino)pyridin-2-yl)piperazine-1-carboxylate **(14a)**.* White solid, yield: 62.1%. ^1H NMR (400 MHz, DMSO-d_6) δ 9.38 (s, 1H, NH), 8.31 (d, *J* = 5.3 Hz, 1H, pyrimidinyl-H), 8.10 (s, 1H, pyridinyl-H), 7.65 (d, *J* = 16.5 Hz, 1H, CH=), 7.50 (s, 2H, PhH), 6.46 (d, *J* = 16.9 Hz, 3H, pyridinyl-2H + CH=), 3.41 (s, 4H, piperazinyl-4H), 3.32 (s, 4H, piperazinyl-4H), 2.07 (s, 6H, CH_3 × 2), 1.42 (s, 9H, CH_3 × 3). ESI-MS: m/z 527.98 (M + H) $^+$, $C_{29}H_{33}N_7O_3$ (527.26).

*tert-butyl 4-(2-cyano-4-((4-(4-cyano-2,6-dimethylphenoxy)pyrimidin-2-yl)amino)phenyl)piperazine-1-carboxylate(**13b**).* White solid, yield: 59.2%. ^1H NMR (400 MHz, DMSO-d_6) δ 9.78 (s, 1H, NH), 8.43 (d, *J* = 5.6 Hz, 1H, pyrimidinyl-H), 7.71 (s, 3H, PhH), 7.47 (s, 1H, PhH), 6.98 (d, *J* = 8.9 Hz, 1H, PhH), 6.62 (d, *J* = 5.6 Hz, 1H, pyrimidinyl-H), 3.48 (s, 4H, piperazinyl-4H), 3.04 – 2.88 (m, 4H, piperazinyl-4H),

2.12 (s, 6H, $CH_3 \times 2$), 1.42 (s, 9H, $CH_3 \times 3$). ESI-MS: m/z 548.51 $(M + H)^+$, $C_{29}H_{31}N_7O_3$ (525.25).

*tert-butyl (E)-4-(2-cyano-4-((4-(4-(2-cyanovinyl)-2,6-dimethylphenoxy)pyrimidin-2-yl)amino)phenyl)piperazine-1-carboxylate(**14b**).* White solid, yield: 62.4%. ^1H NMR (400 MHz, DMSO-d_6) δ 9.75 (s, 1H, NH), 8.40 (d, J = 5.5 Hz, 1H, pyrimidinyl-H), 7.62 (d, J = 16.7 Hz, 1H, CH=), 7.66 (s, 1H, PhH), 7.51 (s, 3H, PhH), 6.85 (d, J = 8.5 Hz, 1H, PhH), 6.58 (d, J = 5.5 Hz, 1H, pyrimidinyl-H), 6.43 (d, J = 16.7 Hz, 1H, CH=), 3.48 (s, 4H, piperazinyl-4H), 2.93 (s, 4H, piperazinyl-4H), 2.08 (s, 6H, $CH_3 \times 2$), 1.42 (s, 9H, $CH_3 \times 3$).

*tert-butyl 4-(4-((4-(4-cyano-2,6-dimethylphenoxy)pyrimidin-2-yl)amino)-2-(trifluoromethyl)phenyl)piperazine-1-carboxylate(**13c**).* White solid, yield: 62.4%.^1H NMR (400 MHz, DMSO-d_6) δ 9.81 (s, 1H, NH), 8.44 (d, J = 5.6 Hz, 1H, pyrimidinyl-H), 7.72 (s, 3H, PhH), 7.57 (s, 1H, PhH), 7.25 (d, J = 8.5 Hz, 1H, PhH), 6.62 (d, J = 5.6 Hz, 1H, pyrimidinyl-H), 3.40 (s, 4H, piperazinyl-4H), 2.71 (t, J = 4.6 Hz, 4H, piperazinyl-4H), 2.10 (s, 6H, $CH_3 \times 2$), 1.42 (s, 9H, $CH_3 \times 3$). ESI-MS: m/z 568.85 $(M + H)^+$, $C_{29}H_{31}F_3N_6O_3$ (568.24).

*tert-butyl (E)-4-(4-((4-(4-(2-cyanovinyl)-2,6-dimethylphenoxy)pyrimidin-2-yl)amino)-2-(trifluoromethyl)phenyl)piperazine-1-carboxylate(**14c**).* White solid, yield: 59.1%. ^1H NMR (400 MHz, DMSO-d_6) δ 9.79 (s, 1H, NH), 8.41 (d, J = 5.6 Hz, 1H, pyrimidinyl-H), 7.75 (s, 1H, PhH), 7.65 (d, J = 16.7 Hz, 1H, CH=), 7.59 (s, 1H, PhH), 7.51 (s, 2H, PhH), 7.16 (d, J = 8.3 Hz, 1H, PhH), 6.57 (d, J = 5.6 Hz, 1H, pyrimidinyl-H), 6.46 (d, J = 16.7 Hz, 1H, CH=), 3.41 (s, 4H, piperazinyl-4H), 2.70 (s, 4H, piperazinyl-4H), 2.08 (s, 6H, $CH_3 \times 2$), 1.43 (s, 9H, $CH_3 \times 3$). ESI-MS: m/z 595.6 $(M + H)^+$, $C_{31}H_{33}F_3N_6O_3$ (594.26).

General procedure for the synthesis of target compounds 17a(1-5), 17b(1-5), 17c(1-5), 18a(1-6), 18b(1-6), and 18c(1-6). To a solution of 1.0 g **13(a-c)** or **14(a-c)** in dichloromethane (10 mL), trifluoroacetic acid (3 mL) was added, and the mixture was stirred at room temperature for 1 h. Next, the reaction mixture was adjust pH 9.0-10.0 with saturated potassium carbonate solution, and was extracted with dichloromethane (3 × 10 mL). The organic phase was washed with saturated NaCl solution (3 × 20 mL),

dried over anhydrous sodium sulphate, filtered, and concentrated under reduced pressure to yield the corresponding intermediates **15(a-c)** or **16(a-c)**. Without further purification, **15(a-c)** or **16(a-c)** (1.0 eq) and triethylamine (1.5 eq) were dissolved in 10 mL dichloromethane at 0-5°C, then various sulfonyl chlorides (1.1 eq) or acryloyl chloride (1.1 eq) was added. Four hours later, saturated $NaHCO_3$ solution (20 mL) was added, and the mixture was extracted with dichloromethane (3 × 12 mL). The generated organic layers were washed with saturated NaCl solution (3 × 16 mL), dried over anhydrous sodium sulphate, filtered and concentrated under reduced pressure. Finally, the residue was separated on a silica gel column chromatography using a methanol/dichloromethane (1:30) system to get target compounds **17a(1-5)**, **17b(1-5)**, **17c(1-5)**, **18a(1-6)**, **18b(1-6)**, and **18c(1-6)**.

*3,5-dimethyl-4-((2-((6-(4-(methylsulfonyl)piperazin-1-yl)pyridin-3-yl)amino)pyrimidin-4-yl)oxy)benzonitrile (**17a1**).* White solid, yield: 67.8%, mp: 204-206°C. ^{1}H NMR (400 MHz, DMSO-d_6) δ 9.39 (s, 1H, NH), 8.34 (d, *J* = 5.6 Hz, 1H, pyrimidinyl-H), 8.12 (s, 1H, pyridinyl-H), 7.72 (s, 2H, PhH), 7.53 (s, 1H, pyridinyl-H), 6.67 (s, 1H, pyridinyl-H), 6.49 (d, *J* = 5.6 Hz, 1H, pyrimidinyl-H), 3.60 – 3.44 (m, 4H, piperazinyl-4H), 3.27 – 3.12 (m, 4H, piperazinyl-4H), 2.91 (s, 3H, CH_3), 2.11 (s, 6H, CH_3 × 2). ^{13}C NMR (100 MHz, DMSO-d_6) δ 168.34, 161.00, 160.32, 154.64, 153.86, 139.56, 133.08, 130.33, 128.63, 119.15, 108.90, 107.24, 97.39, 45.52, 45.36, 34.26, 16.27. ESI-MS: m/z 480.11 (M + H) $^+$, $C_{23}H_{25}N_7O_3S$ (479.17). HPLC purity: 100% (λ = 254 nM).

*4-((2-((6-(4-(ethylsulfonyl)piperazin-1-yl)pyridin-3-yl)amino)pyrimidin-4-yl)oxy)-3,5-dimethylbenzonitrile (**17a2**).* White solid, yield: 64.5%, mp: 208-210°C. ^{1}H NMR (400 MHz, DMSO-d_6) δ 9.38 (s, 1H, NH), 8.47 – 8.26 (m, 1H, pyrimidinyl-H), 8.10 (s, 1H, pyridinyl-H), 7.72 (s, 2H, PhH), 7.53 (s, 1H, pyridinyl-H), 6.65 (s, 1H, pyridinyl-H), 6.56 – 6.18 (m, 1H pyrimidinyl-H), 3.47 (s, 4H, piperazinyl-4H), 3.26 (s, 4H, piperazinyl-4H), 3.16 – 2.95 (m, 2H, CH_2), 2.10 (s, 6H, CH_3 × 2), 1.23 (t, *J* = 7.4 Hz, 3H, CH_3). ^{13}C NMR (100 MHz, DMSO-d_6) δ 168.34, 161.02, 160.32, 154.69, 153.83, 139.53, 133.08, 130.20, 128.63, 119.15, 108.90, 107.23, 97.42, 45.70, 45.39, 42.77, 16.27, 7.91. ESI-MS: m/z 494.12 (M + H) $^+$, $C_{24}H_{27}N_7O_3S$ (493.19). HPLC purity: 100%

(λ = 254 nM).

*4-((2-((6-(4-(isopropylsulfonyl)piperazin-1-yl)pyridin-3-yl)amino)pyrimidin-4-yl)oxy)-3,5-dimethylbenzonitrile (**17a3**).* White solid, yield: 69.3%, mp: 212-214℃. ^{1}H NMR (400 MHz, DMSO-d_6) δ 9.38 (s, 1H, NH), 8.34 (d, *J* = 5.6 Hz, 1H, pyrimidinyl-H), 8.09 (s, 1H, pyridinyl-H), 7.72 (s, 2H, PhH), 7.51 (s, 1H, pyridinyl-H), 6.64 (s, 1H, pyridinyl-H), 6.49 (d, *J* = 5.6 Hz, 1H, pyrimidinyl-H), 3.51 – 3.41 (m, 4H, piperazinyl-4H), 3.41 – 3.36 (m, 1H, CH), 3.34 (s, 4H, piperazinyl-4H), 2.10 (s, 6H, $CH_3 \times 2$), 1.24 (d, *J* = 6.8 Hz, 6H, $CH_3 \times 2$). ^{13}C NMR (100 MHz, DMSO-d_6) δ 168.34, 161.00, 160.33, 154.78, 153.85, 139.48, 133.08, 130.23, 128.61, 119.13, 108.90, 107.19, 97.34, 52.21, 46.15, 45.78, 16.93, 16.27. ESI-MS: m/z 508.14 (M + H) $^+$, $C_{25}H_{29}N_7O_3S$ (507.21). HPLC purity: 99.85% (λ = 254 nM).

*4-(5-((4-(4-cyano-2,6-dimethylphenoxy)pyrimidin-2-yl)amino)pyridin-2-yl)-N,N-dimethylpiperazine-1-sulfonamide (**17a4**).* White solid, yield: 69.3%, mp: 200-202℃. ^{1}H NMR (400 MHz, DMSO-d_6) δ 9.38 (s, 1H, NH), 8.34 (d, *J* = 5.5 Hz, 1H, pyrimidinyl-H), 8.12 (s, 1H, pyridinyl-H), 7.72 (s, 2H, PhH), 7.52 (s, 1H, pyridinyl-H), 6.64 (s, 1H, pyridinyl-H), 6.49 (d, *J* = 5.5 Hz, 1H, pyrimidinyl-H), 3.45 (s, 4H, piperazinyl-4H), 3.24 (s, 4H, piperazinyl-4H), 2.80 (s, 6H, $CH_3 \times 2$), 2.10 (s, 6H, $CH_3 \times 2$). ^{13}C NMR (100 MHz, DMSO-d_6) δ 168.34, 160.96, 160.33, 154.75, 153.85, 139.52, 133.08, 130.25, 128.63, 119.14, 108.89, 107.17, 97.33, 46.21, 45.56, 38.38, 16.27. ESI-MS: m/z 509.05 (M + H) $^+$, $C_{24}H_{28}N_8O_3S$ (508.20). HPLC purity: 100% (λ = 254 nM).

*4-((2-((6-(4-(cyclopropylsulfonyl)piperazin-1-yl)pyridin-3-yl)amino)pyrimidin-4-yl)oxy)-3,5-dimethylbenzonitrile (**17a5**).* White solid, yield: 62.6%, mp: 220-222℃. ^{1}H NMR (400 MHz, DMSO-d_6) δ 9.37 (s, 1H, NH), 8.34 (d, *J* = 5.6 Hz, 1H, pyrimidinyl-H), 8.11 (s, 1H, pyridinyl-H), 7.72 (s, 2H, PhH), 7.55 (s, 1H, pyridinyl-H), 6.66 (s, 1H, pyridinyl-H), 6.49 (d, *J* = 5.6 Hz, 1H, pyrimidinyl-H), 3.58 – 3.43 (m, 4H, piperazinyl-4H), 3.27 (t, *J* = 5.0 Hz, 4H, piperazinyl-4H), 2.63 (tt, *J* = 7.8, 4.9 Hz, 1H, CH), 2.10 (s, 6H, $CH_3 \times 2$), 1.04 – 0.92 (m, 4H, $CH_2 \times 2$). ^{13}C NMR (100 MHz, DMSO-d_6) δ 168.35, 161.00, 160.35, 154.69, 153.86, 139.58, 133.08, 130.31, 128.62, 119.13, 108.91, 107.26, 97.36, 45.93, 45.51, 25.15, 16.27, 4.34. ESI-MS: m/z 506.11 (M + H) +, $C_{25}H_{27}N_7O_3S$ (505.19). HPLC purity: 100% (λ = 254 nM).

(E)-3-(3,5-dimethyl-4-((2-((6-(4-(methylsulfonyl)piperazin-1-yl)pyridin-3-yl)amino)pyrimidin-4-yl)oxy)phenyl)acrylonitrile ***(17b1)***. White solid, yield: 69.1%, mp: 201-203°C. ^{1}H NMR (400 MHz, DMSO-d_6) δ 9.37 (s, 1H, NH), 8.32 (d, *J* = 5.6 Hz, 1H, pyrimidinyl-H), 8.14 (s, 1H, pyridinyl-H), 7.65 (d, *J* = 16.7 Hz, 1H, CH=), 7.50 (s, 2H, PhH), 6.57 (s, 1H, pyridinyl-H), 6.51 – 6.40 (m, 2H, pyridinyl-H + CH=), 3.54 – 3.43 (m, 4H, piperazinyl-4H), 3.26 – 3.15 (m, 4H, piperazinyl-4H), 2.90 (s, 3H, CH_3), 2.08 (s, 6H, $CH_3 \times 2$). ^{13}C NMR (100 MHz, DMSO-d_6) δ 168.74, 160.78, 160.37, 154.53, 152.22, 150.62, 139.32, 131.78, 131.71, 129.68, 128.85, 128.72, 119.38, 107.18, 97.35, 96.66, 45.55, 45.36, 34.28, 16.53. ESI-MS: 504.40 m/z $(M - H)^-$, $C_{25}H_{27}N_7O_3S$ (505.60). HPLC purity: 100% (λ = 254 nM).

(E)-3-(4-((2-((6-(4-(ethylsulfonyl)piperazin-1-yl)pyridin-3-yl)amino)pyrimidin-4-yl)oxy)-3,5-dimethylphenyl)acrylonitrile ***(17b2)***. White solid, yield: 62.5%, mp: 184-186°C. ^{1}H NMR (400 MHz, DMSO-d_6) δ 9.31 (s, 1H, NH), 8.25 (d, *J* = 5.5 Hz, 1H, pyrimidinyl-H), 8.08 (s, 1H, pyridinyl-H), 7.58 (d, *J* = 16.7 Hz, 1H, CH=), 7.43 (s, 2H, PhH), 6.49 (s, 1H, pyridinyl-H), 6.44 – 6.29 (m, 2H, pyridinyl-H + CH=), 3.37 (s, 4H, piperazinyl-4H), 3.24 – 3.13 (m, 4H, piperazinyl-4H), 3.01 (q, *J* = 7.4 Hz, 2H, CH_2), 2.01 (s, 6H, $CH_3 \times 2$), 1.16 (t, *J* = 7.4 Hz, 3H, CH_3). ^{13}C NMR (100 MHz, DMSO-d_6) δ 168.73, 160.82, 160.35, 154.57, 152.22, 150.62, 139.23, 131.77, 131.70, 129.68, 128.86, 128.73, 119.39, 107.18, 97.46, 96.65, 45.70, 45.42, 42.79, 16.53, 7.91. ESI-MS: 518.39 m/z $(M - H)^-$, $C_{26}H_{29}N_7O_3S$ (519.21). HPLC purity: 99.99% (λ = 254 nM).

(E)-3-(4-((2-((6-(4-(isopropylsulfonyl)piperazin-1-yl)pyridin-3-yl)amino)pyrimidin-4-yl)oxy)-3,5-dimethylphenyl)acrylonitrile ***(17b3)***. White solid, yield: 63.5%, mp: 208-210°C. ^{1}H NMR (400 MHz, DMSO-d_6) δ 9.31 (s, 1H, NH), 8.25 (d, *J* = 5.5 Hz, 1H, pyrimidinyl-H), 8.06 (s, 1H, pyridinyl-H), 7.58 (d, *J* = 16.7 Hz, 1H, CH=), 7.43 (s, 2H, PhH), 6.48 (s, 1H, pyridinyl-H), 6.41 – 6.29 (m, 2H, pyridinyl-H + CH=), 3.32 (dt, *J* = 9.9, 5.5 Hz, 5H, piperazinyl-4H + CH), 3.26 (d, *J* = 8.6 Hz, 4H, piperazinyl-4H), 2.01 (s, 6H, $CH_3 \times 2$), 1.17 (d, *J* = 6.8 Hz, 6H, $CH_3 \times 2$). ^{13}C NMR (100 MHz, DMSO-d_6) δ 168.73, 160.82, 160.34, 154.65, 152.23, 150.62, 139.19, 131.77, 131.70, 129.68, 128.85, 128.72, 119.38, 107.15, 97.43, 96.65, 52.27, 46.16, 45.82, 16.94, 16.53. ESI-MS: 534.16 m/z $(M + H)^+$, $C_{27}H_{31}N_7O_3S$ (533.22). HPLC purity: 99.99% (λ = 254 nM).

(E)-4-(5-((4-(4-(2-cyanovinyl)-2,6-dimethylphenoxy)pyrimidin-2-yl)amino)pyridin-2-yl)-N,N-dimethylpiperazine-1-sulfonamide ***(17b4)***. White solid, yield: 69.1%, mp: 226-228℃. ^{1}H NMR (400 MHz, DMSO-d_6) δ 9.37 (s, 1H, NH), 8.32 (d, *J* = 5.6 Hz, 1H, pyrimidinyl-H), 8.12 (s, 1H, pyridinyl-H), 7.65 (d, *J* = 16.7 Hz, 1H, CH=), 7.50 (s, 2H, PhH), 6.53 (s, 1H, pyridinyl-H), 6.49 – 6.41 (m, 2H, pyridinyl-H + CH=), 3.41 (d, *J* = 4.8 Hz, 4H, piperazinyl-4H), 3.27 – 3.21 (m, 4H, piperazinyl-4H), 2.81 (s, 6H, CH_3 × 2), 2.08 (s, 6H, CH_3 × 2). ^{13}C NMR (100 MHz, DMSO-d_6) δ 168.74, 160.80, 160.34, 154.62, 152.26, 150.62, 139.25, 131.78, 131.71, 129.67, 128.88, 128.71, 119.37, 107.12, 97.37, 96.63, 46.24, 45.58, 38.38, 16.51. ESI-MS: 535.13 m/z (M + H) $^+$, 557.15 m/z (M + Na)$^+$, $C_{26}H_{30}N_8O_3S$ (534.22). HPLC purity: 99.99% (λ = 254 nM).

(E)-3-(4-((2-((6-(4-(cyclopropylsulfonyl)piperazin-1-yl)pyridin-3-yl)amino)pyrimidin-4-yl)oxy)-3,5-dimethylphenyl)acrylonitrile ***(17b5)***. White solid, yield: 71.4%, mp: 220-222℃. ^{1}H NMR (400 MHz, DMSO-d_6) δ 9.37 (s, 1H, NH), 8.32 (d, *J* = 5.6 Hz, 1H, pyrimidinyl-H), 8.13 (s, 1H, pyridinyl-H), 7.65 (d, *J* = 16.7 Hz, 1H, CH=), 7.50 (s, 2H, PhH), 6.55 (s, 1H, pyridinyl-H), 6.50 – 6.34 (m, 2H, pyridinyl-H + CH=), 3.53 – 3.41 (m, 4H, piperazinyl-4H), 3.28 (t, *J* = 5.0 Hz, 4H, piperazinyl-4H), 2.62 (tt, *J* = 7.9, 5.0 Hz, 1H, CH), 2.08 (s, 6H, CH_3 × 2), 0.98 (ddt, *J* = 14.5, 5.1, 3.0 Hz, 4H, CH_2 × 2). ^{13}C NMR (100 MHz, DMSO-d_6) δ 168.74, 160.80, 160.36, 154.55, 152.24, 150.63, 139.31, 131.78, 131.71, 130.07, 128.86, 128.72, 119.38, 107.19, 97.41, 96.65, 45.96, 45.50, 25.18, 16.53, 4.35. ESI-MS: 532.17 m/z (M + H) $^+$, 554.25 m/z (M + Na)$^+$, $C_{27}H_{29}N_7O_3S$ (531.21). HPLC purity: 99.98% (λ = 254 nM).

4-((2-((3-cyano-4-(4-(methylsulfonyl)piperazin-1-yl)phenyl)amino)pyrimidin-4-yl)oxy)-3,5-dimethylbenzonitrile ***(18a1)***. White solid, yield: 71.8%, mp: 244-246℃. ^{1}H NMR (400 MHz, DMSO-d_6) δ 9.80 (s, 1H, NH), 8.44 (d, *J* = 5.6 Hz, 1H, pyrimidinyl-H), 7.71 (s, 3H, PhH), 7.50 (s, 1H, PhH), 7.03 (d, *J* = 9.0 Hz, 1H, PhH), 6.63 (d, *J* = 5.6 Hz, 1H, pyrimidinyl-H), 3.31 – 3.24 (m, 4H, piperazinyl-4H), 3.16 – 3.07 (m, 4H, piperazinyl-4H), 2.97 (s, 3H, CH_3), 2.12 (s, 6H, CH_3 × 2). ^{13}C NMR (100 MHz, DMSO-d_6) δ 168.38, 161.08, 159.83, 153.65, 149.44, 135.89, 133.20, 132.88, 125.10, 123.03, 120.50, 119.06, 118.17, 109.32, 106.24, 98.46, 51.65, 46.08, 34.49, 16.28. ESI-MS: m/z 502.46 (M - H)$^-$, $C_{25}H_{25}N_7O_3S$ (503.17). HPLC purity: 99.14% (λ = 254 nM).

*4-((2-((3-cyano-4-(4-(ethylsulfonyl)piperazin-1-yl)phenyl)amino)pyrimidin-4-yl)oxy)-3,5-dimethylbenzonitrile (**18a2**).* White solid, yield: 72.5%, mp: 238-240°C. ^{1}H NMR (400 MHz, DMSO-d_6) δ 9.73 (s, 1H, NH), 8.37 (d, *J* = 5.6 Hz, 1H, pyrimidinyl-H), 7.64 (s, 3H, PhH), 7.43 (s, 1H, PhH), 6.95 (d, *J* = 9.0 Hz, 1H, PhH), 6.56 (d, *J* = 5.6 Hz, 1H, pyrimidinyl-H), 3.30 – 3.27 (m, 4H, piperazinyl-4H), 3.07 (q, *J* = 7.4 Hz, 2H, CH_2), 3.03 – 2.97 (m, 4H, piperazinyl-4H), 2.05 (s, 6H, CH_3 × 2), 1.18 (t, *J* = 7.3 Hz, 3H, CH_3). ^{13}C NMR (100 MHz, DMSO-d_6) δ 168.38, 161.07, 159.84, 153.65, 149.50, 135.87, 133.20, 132.88, 125.09, 123.04, 120.51, 119.06, 118.18, 109.31, 106.25, 98.46, 51.99, 45.93, 43.03, 16.28, 7.96. ESI-MS: m/z 516.25 $(M - H)^-$, $C_{26}H_{27}N_7O_3S$ (517.19). HPLC purity: 99.99% (λ = 254 nM).

*4-((2-((3-cyano-4-(4-(isopropylsulfonyl)piperazin-1-yl)phenyl)amino)pyrimidin-4-yl)oxy)-3,5-dimethylbenzonitrile (**18a3**).* White solid, yield: 70.1%, mp: 240-242°C. ^{1}H NMR (400 MHz, DMSO-d_6) δ 9.88 – 9.64 (m, 1H, NH), 8.37 (d, *J* = 5.6 Hz, 1H, pyrimidinyl-H), 7.64 (s, 3H, PhH), 7.42 (s, 1H, PhH), 6.94 (d, *J* = 9.0 Hz, 1H, PhH), 6.56 (d, *J* = 5.6 Hz, 1H, pyrimidinyl-H), 3.40 – 3.32 (m, 5H, piperazinyl-4H + CH), 3.03 – 2.93 (m, 4H, piperazinyl-4H), 2.05 (s, 6H, CH_3 × 2), 1.19 (d, *J* = 6.8 Hz, 6H, CH_3 × 2). ^{13}C NMR (100 MHz, DMSO-d_6) δ 168.38, 161.08, 159.83, 153.65, 149.58, 135.84, 133.20, 132.88, 125.09, 120.52, 119.06, 118.19, 109.31, 106.26, 98.46, 52.45, 52.37, 46.32, 16.95, 16.28. ESI-MS: m/z 530.35 $(M - H)^-$, $C_{27}H_{29}N_7O_3S$ (531.21). HPLC purity: 100% (λ = 254 nM).

*4-(2-cyano-4-((4-(4-cyano-2,6-dimethylphenoxy)pyrimidin-2-yl)amino)phenyl)-N,N-dimethylpiperazine-1-sulfonamide (**18a4**).* White solid, yield: 65.3%, mp: 220-222°C. ^{1}H NMR (400 MHz, DMSO-d_6) δ 9.80 (s, 1H, NH), 8.44 (d, *J* = 5.6 Hz, 1H, pyrimidinyl-H), 7.71 (s, 3H, PhH), 7.51 (s, 1H, PhH), 7.01 (d, *J* = 8.9 Hz, 1H, PhH), 6.62 (d, *J* = 5.6 Hz, 1H, pyrimidinyl-H), 3.31 (s, 4H, piperazinyl-4H), 3.10 – 3.02 (m, 4H, piperazinyl-4H), 2.81 (s, 6H, CH_3 × 2), 2.12 (s, 6H, CH_3 × 2). ^{13}C NMR (100 MHz, DMSO-d_6) δ 168.38, 161.07, 159.84, 153.65, 149.50, 135.83, 133.20, 132.89, 125.10, 123.03, 120.43, 119.07, 118.18, 109.30, 106.20, 98.45, 51.81, 46.73, 38.38, 16.28. ESI-MS: m/z 531.23 $(M - H)^-$, $C_{26}H_{28}N_8O_3S$ (532.20). HPLC purity: 99.92% (λ = 254 nM).

4-((2-((3-cyano-4-(4-(cyclopropylsulfonyl)piperazin-1-yl)phenyl)amino)pyrimidin-

4-yl)oxy)-3,5-dimethylbenzonitrile (***18a5***). White solid, yield: 65.9%, mp: 240-242℃. ^{1}H NMR (400 MHz, DMSO-d_6) δ 9.81 (s, 1H, NH), 8.44 (d, *J* = 5.6 Hz, 1H, pyrimidinyl-H), 7.71 (s, 3H, PhH), 7.51 (s, 1H, PhH), 7.03 (d, *J* = 8.9 Hz, 1H, PhH), 6.63 (d, *J* = 5.6 Hz, 1H, pyrimidinyl-H), 3.37 (t, *J* = 4.9 Hz, 4H, piperazinyl-4H), 3.09 (dd, *J* = 6.1, 3.5 Hz, 4H, piperazinyl-4H), 2.76 – 2.66 (m, 1H, CH), 2.12 (s, 6H, CH_3 × 2), 1.12 – 0.86 (m, 4H, CH_2 × 2). ^{13}C NMR (100 MHz, DMSO-d_6) δ 168.38, 161.08, 159.83, 153.65, 149.47, 135.91, 133.20, 132.89, 125.09, 123.00, 120.52, 119.07, 118.18, 109.31, 106.27, 98.46, 51.78, 46.49, 25.19, 16.28, 4.36. ESI-MS: m/z 528.45 $(M - H)^-$, $C_{27}H_{27}N_7O_3S$ (529.19). HPLC purity: 100% (λ = 254 nM).

(E)-5-((4-(4-(2-cyanovinyl)-2,6-dimethylphenoxy)pyrimidin-2-yl)amino)-2-(4-(methylsulfonyl)piperazin-1-yl)benzonitrile (***18b1***). White solid, yield: 65.5%, mp: 240-242℃. ^{1}H NMR (400 MHz, DMSO-d_6) δ 9.78 (s, 1H, NH), 8.42 (d, *J* = 5.3 Hz, 1H, pyrimidinyl-H), 7.75 (s, 1H, PhH), 7.61 (d, *J* = 16.7 Hz, 1H, CH=), 7.51 (s, 3H, PhH), 6.95 (d, *J* = 7.9 Hz, 1H, PhH), 6.58 (d, *J* = 5.6 Hz, 1H, pyrimidinyl-H), 6.42 (d, *J* = 16.7 Hz, 1H, CH=), 3.28 (d, *J* = 5.0 Hz, 4H, piperazinyl-4H), 3.18 – 3.03 (m, 4H, piperazinyl-4H), 2.96 (s, 3H, CH_3), 2.10 (s, 6H, CH_3 × 2). ^{13}C NMR (100 MHz, DMSO-d_6) δ 168.76, 160.85, 159.86, 152.05, 150.67, 149.31, 136.03, 131.82, 131.55, 129.80, 128.87, 124.94, 123.05, 120.40, 119.41, 118.14, 106.22, 98.45, 96.70, 51.67, 46.08, 34.48, 16.54. ESI-MS: 528.38 m/z $(M - H)^-$, $C_{27}H_{27}N_7O_3S$ (529.19). HPLC purity: 98.35% (λ = 254 nM).

(E)-5-((4-(4-(2-cyanovinyl)-2,6-dimethylphenoxy)pyrimidin-2-yl)amino)-2-(4-(ethylsulfonyl)piperazin-1-yl)benzonitrile (***18b2***). White solid, yield: 68.1%, mp: 254-256℃. ^{1}H NMR (400 MHz, DMSO-d_6) δ 9.76 (s, 1H, NH), 8.41 (d, *J* = 5.6 Hz, 1H, pyrimidinyl-H), 7.75 (s, 1H, PhH), 7.61 (d, *J* = 16.7 Hz, 1H, CH=), 7.51 (s, 3H, PhH), 6.94 (d, *J* = 8.6 Hz, 1H, PhH), 6.58 (d, *J* = 5.6 Hz, 1H, pyrimidinyl-H), 6.41 (d, *J* = 16.7 Hz, 1H, CH=), 3.41 – 3.33 (m, 4H, piperazinyl-4H), 3.16 – 3.10 (m, 2H, CH_2), 3.10 – 2.97 (m, 4H, piperazinyl-4H), 2.09 (s, 6H, CH_3 × 2), 1.25 (t, *J* = 7.4 Hz, 3H, CH_3). ^{13}C NMR (100 MHz, DMSO-d_6) δ 168.75, 160.84, 159.86, 152.05, 150.66, 149.36, 136.01, 131.82, 131.55, 128.87, 124.93, 123.06, 120.40, 119.41, 118.15, 106.22, 98.52, 96.70, 52.00, 45.92, 42.99, 16.54, 7.96. ESI-MS: 544.5 m/z $(M + H)^+$, $C_{28}H_{29}N_7O_3S$ (543.21).

HPLC purity: 100% (λ = 254 nM).

*(E)-5-((4-(4-(2-cyanovinyl)-2,6-dimethylphenoxy)pyrimidin-2-yl)amino)-2-(4-(isopropylsulfonyl)piperazin-1-yl)benzonitrile (**18b3**).* White solid, yield: 71.4%, mp: 244-246°C. ^{1}H NMR (400 MHz, DMSO-d_6) δ 9.76 (s, 1H, NH), 8.41 (d, *J* = 5.5 Hz, 1H, pyrimidinyl-H), 7.74 (s, 1H, PhH), 7.61 (d, *J* = 16.7 Hz, 1H, CH=), 7.51 (s, 3H, PhH), 6.93 (d, *J* = 8.0 Hz, 1H, PhH), 6.57 (d, *J* = 5.6 Hz, 1H, pyrimidinyl-H), 6.41 (d, *J* = 16.7 Hz, 1H, CH=), 3.41 (s, 5H, piperazinyl-4H + CH), 3.02 (s, 4H, piperazinyl-4H), 2.09 (s, 6H, CH_3 × 2), 1.26 (d, *J* = 6.8 Hz, 6H, CH_3 × 2). ^{13}C NMR (100 MHz, DMSO-d_6) δ 168.76, 160.84, 159.88, 152.06, 150.63, 149.45, 135.98, 131.82, 131.55, 129.80, 128.86, 124.95, 123.12, 120.41, 119.37, 118.16, 106.24, 98.48, 96.71, 52.46, 46.32, 16.95, 16.53. ESI-MS: 558.21 m/z $(M + H)^+$, 580.28 m/z $(M + Na)^+$, $C_{29}H_{31}N_7O_3S$ (557.22). HPLC purity: 100% (λ = 254 nM).

*(E)-4-(2-cyano-4-((4-(4-(2-cyanovinyl)-2,6-dimethylphenoxy)pyrimidin-2-yl)amino)phenyl)-N,N-dimethylpiperazine-1-sulfonamide (**18b4**).* White solid, yield: 69.2%, mp: 234-236°C. ^{1}H NMR (400 MHz, DMSO-d_6) δ 9.77 (s, 1H, NH), 8.41 (d, *J* = 5.5 Hz, 1H, pyrimidinyl-H), 7.72 (s, 1H, PhH), 7.61 (d, *J* = 16.7 Hz, 1H, CH=), 7.51 (s, 3H, PhH), 6.89 (d, *J* = 8.0 Hz, 1H, PhH), 6.58 (d, *J* = 5.6 Hz, 1H, pyrimidinyl-H), 6.43 (d, *J* = 16.7 Hz, 1H, CH=), 3.33 – 3.26 (m, 4H, piperazinyl-4H), 3.13 – 2.98 (m, 4H, piperazinyl-4H), 2.82 (s, 6H, CH_3 × 2), 2.09 (s, 6H, CH_3 × 2). ^{13}C NMR (100 MHz, DMSO-d_6) δ 168.74, 160.85, 159.85, 152.09, 150.64, 149.32, 135.95, 131.80, 131.57, 129.80, 128.85, 124.93, 123.17, 120.25, 119.39, 118.15, 106.16, 98.48, 96.70, 51.82, 46.71, 38.37, 16.53. ESI-MS: 559.14 m/z $(M + H)^+$, 581.17 m/z $(M + Na)^+$, $C_{28}H_{30}N_8O_3S$ (558.22). HPLC purity: 100% (λ = 254 nM).

*(E)-5-((4-(4-(2-cyanovinyl)-2,6-dimethylphenoxy)pyrimidin-2-yl)amino)-2-(4-(cyclopropylsulfonyl)piperazin-1-yl)benzonitrile (**18b5**).* White solid, yield: 63.4%, mp: 246-248°C. ^{1}H NMR (400 MHz, DMSO-d_6) δ 9.78 (s, 1H, NH), 8.41 (d, *J* = 5.6 Hz, 1H, pyrimidinyl-H), 7.74 (s, 1H, PhH), 7.61 (d, *J* = 16.7 Hz, 1H, CH=), 7.51 (s, 3H, PhH), 6.94 (d, *J* = 7.9 Hz, 1H, PhH), 6.58 (d, *J* = 5.6 Hz, 1H, pyrimidinyl-H), 6.42 (d, *J* = 16.7 Hz, 1H, CH=), 3.36 (d, *J* = 4.7 Hz, 4H, piperazinyl-4H), 3.07 (s, 4H, piperazinyl-4H), 2.71 (ddd, *J* = 12.5, 7.9, 4.8 Hz, 1H, CH), 2.09 (s, 6H, CH_3 × 2), 1.08

– 0.90 (m, 4H, CH_2 × 2). ^{13}C NMR (100 MHz, DMSO-d_6) δ 168.75, 160.85, 159.87, 152.06, 150.66, 149.34, 136.03, 131.81, 131.55, 129.80, 128.86, 124.95, 123.10, 120.38, 119.41, 118.16, 106.24, 98.46, 96.69, 51.79, 46.48, 25.20, 16.54, 4.36. ESI-MS: 556.13 m/z $(M + H)^+$, 578.19 m/z $(M + Na)^+$, $C_{29}H_{29}N_7O_3S$ (555.21). HPLC purity: 99.95% (λ = 254 nM).

*(E)-2-(4-acryloylpiperazin-1-yl)-5-((4-(4-(2-cyanovinyl)-2,6-dimethylphenoxy)pyrimidin-2-yl)amino)benzonitrile (**18b6**)*. White solid, yield: 61.2%, mp: 200-202℃. ^1H NMR (400 MHz, DMSO-d_6) δ 9.76 (s, 1H, NH), 8.41 (d, *J* = 5.5 Hz, 1H, pyrimidinyl-H), 7.76 (s, 1H, PhH), 7.62 (d, *J* = 16.7 Hz, 1H, CH=), 7.51 (s, 3H, PhH), 6.90 (d, *J* = 7.5 Hz, 1H, PhH), 6.84 (dd, *J* = 16.8, 10.4 Hz, 1H, CH=), 6.58 (d, *J* = 5.6 Hz, 1H, pyrimidinyl-H), 6.43 (d, *J* = 16.7 Hz, 1H, CH=), 6.15 (dd, *J* = 16.7, 2.2 Hz, 1H, CH=), 5.72 (dd, *J* = 10.5, 2.2 Hz, 1H, CH=), 3.72 (s, 4H, piperazinyl-4H), 2.99 (s, 4H, piperazinyl-4H), 2.09 (s, 6H, CH_3 × 2). ^{13}C NMR (100 MHz, DMSO-d_6) δ 168.77, 164.84, 160.83, 159.91, 152.08, 150.62, 149.49, 135.83, 131.82, 131.57, 129.80, 128.85, 128.62, 127.93, 125.01, 123.16, 120.22, 119.36, 118.24, 106.04, 98.42, 96.74, 52.56, 51.89, 45.78, 42.03, 16.52.ESI-MS: 506.39 m/z $(M + H)^+$, 528.35 m/z $(M + Na)^+$, $C_{29}H_{27}N_7O_2$ (505.22). HPLC purity: 100% (λ = 254 nM).

*3,5-dimethyl-4-((2-((4-(4-(methylsulfonyl)piperazin-1-yl)-3-(trifluoromethyl)phenyl)amino)pyrimidin-4-yl)oxy)benzonitrile (**17c1**)*. White solid, yield: 61.2%, mp: 180-182℃. ^1H NMR (600 MHz, DMSO-d_6) δ 9.82 (s, 1H, NH), 8.44 (d, *J* = 5.6 Hz, 1H, pyrimidinyl-H), 7.75 (s, 1H, PhH), 7.72 (s, 2H, PhH), 7.59 (s, 1H, PhH), 7.34 (d, *J* = 8.3 Hz, 1H, PhH), 6.62 (d, *J* = 5.6 Hz, 1H, pyrimidinyl-H), 3.20 (s, 4H, piperazinyl-4H), 2.94 (s, 3H, CH_3), 2.87 (t, *J* = 4.7 Hz, 4H, piperazinyl-4H), 2.11 (s, 6H, CH_3 × 2). ^{13}C NMR (150 MHz, DMSO-d_6) δ 168.38, 161.00, 159.95, 153.66, 145.31, 138.08, 133.10, 132.97, 125.77 (J_{CF} = 186 Hz), 125.34, 123.67, 119.03, 117.18 (J_{CF} = 3.8 Hz), 109.01, 98.64, 53.05, 46.48, 34.47, 16.25.ESI-MS: m/z 547.12 $(M + H)^+$, $C_{25}H_{25}F_3N_6O_3S$ (546.17). HPLC purity: 100% (λ = 254 nM).

*4-((2-((4-(4-(ethylsulfonyl)piperazin-1-yl)-3-(trifluoromethyl)phenyl)amino)pyrimidin-4-yl)oxy)-3,5-dimethylbenzonitrile (**17c2**)*. White solid, yield: 63.4%, mp: 174-176℃. ^1H NMR (400 MHz, DMSO-d_6) δ 9.83 (s,

1H, NH), 8.44 (d, *J* = 5.6 Hz, 1H, pyrimidinyl-H), 7.74 (d, *J* = 11.8 Hz, 3H, PhH), 7.58 (s, 1H, PhH), 7.33 (d, *J* = 8.5 Hz, 1H, PhH), 6.63 (d, *J* = 5.6 Hz, 1H, pyrimidinyl-H), 3.27 (s, 4H, piperazinyl-4H), 3.13 (q, *J* = 7.3 Hz, 2H, CH_2), 2.90 – 2.79 (m, 4H, piperazinyl-4H), 2.11 (s, 6H, CH_3 × 2), 1.24 (t, *J* = 7.3 Hz, 3H, CH_3). ^{13}C NMR (150 MHz, DMSO-d_6) δ 168.38, 161.01, 159.95, 153.66, 145.34, 138.06, 133.10, 132.99, 125.83 (J_{CF} = 191 Hz), 125.31, 123.65, 117.18 (J_{CF} = 3.8 Hz), 109.01, 98.63, 53.38, 46.32, 43.02, 16.25, 7.96. ESI-MS: m/z 561.13 $(M + H)^+$, $C_{26}H_{27}F_3N_6O_3S$ (560.18). HPLC purity: 100% (λ = 254 nM).

4-((2-((4-(4-(isopropylsulfonyl)piperazin-1-yl)-3-(trifluoromethyl)phenyl)amino)pyrimidin-4-yl)oxy)-3,5-dimethylbenzonitrile ***(17c3)****.* White solid, yield: 73.4%, mp: 178-180℃. ^{1}H NMR (600 MHz, DMSO-d_6) δ 9.79 (s, 1H, NH), 8.44 (d, *J* = 5.3 Hz, 1H, pyrimidinyl-H), 7.76 (s, 1H, PhH), 7.71 (s, 2H, PhH), 7.58 (s, 1H, PhH), 7.30 (d, *J* = 8.3 Hz, 1H, PhH), 6.62 (d, *J* = 5.3 Hz, 1H, pyrimidinyl-H), 3.37 (d, *J* = 32.1 Hz, 5H, piperazinyl-4H + CH), 2.82 (s, 4H, piperazinyl-4H), 2.12 (s, 6H, CH_3 × 2), 1.26 (d, *J* = 6.6 Hz, 6H, CH_3 × 2). ^{13}C NMR (100 MHz, DMSO-d_6) δ 168.37, 161.03, 159.91, 153.65, 145.35, 138.04, 133.11, 132.97, 125.52 (J_{CF} = 135 Hz), 125.33, 123.60, 119.05, 117.14 (J_{CF} = 4.8 Hz), 108.99, 98.66, 53.82, 52.30, 46.70, 16.96, 16.26. ESI-MS: m/z 575.13 $(M + H)^+$, $C_{27}H_{29}F_3N_6O_3S$ (574.20). HPLC purity: 100% (λ = 254 nM).

4-(4-((4-(4-cyano-2,6-dimethylphenoxy)pyrimidin-2-yl)amino)-2-(trifluoromethyl)phenyl)-N,N-dimethylpiperazine-1-sulfonamide ***(17c4)****.* White solid, yield: 66.3%, mp: 190-192℃. ^{1}H NMR (400 MHz, DMSO-d_6) δ 9.82 (s, 1H, NH), 8.44 (d, *J* = 5.6 Hz, 1H, pyrimidinyl-H), 7.74 (d, *J* = 8.5 Hz, 3H, PhH), 7.56 (s, 1H, PhH), 7.30 (d, *J* = 8.8 Hz, 1H, PhH), 6.63 (d, *J* = 5.6 Hz, 1H, pyrimidinyl-H), 3.24 (s, 4H, piperazinyl-4H), 2.83 (d, *J* = 4.5 Hz, 4H, piperazinyl-4H), 2.81 (s, 6H, CH_3 × 2), 2.11 (s, 6H, CH_3 × 2). ^{13}C NMR (150 MHz, DMSO-d_6) δ 168.38, 161.01, 159.95, 153.68, 145.33, 138.03, 133.09, 132.99, 125.66 (J_{CF} = 178 Hz), 125.16, 123.65, 119.03, 117.25 (J_{CF} = 3.8 Hz), 109.01, 98.61, 53.25, 47.07, 38.38, 16.24. ESI-MS: m/z 576.14 $(M + H)^+$, m/z 598.16 $(M + Na)^+$, $C_{26}H_{28}F_3N_7O_3S$ (575.19). HPLC purity: 99.15% (λ = 254 nM).

4-((2-((4-(4-(cyclopropylsulfonyl)piperazin-1-yl)-3-(trifluoromethyl)phenyl)amino)pyrimidin-4-yl)oxy)-3,5-dimethylbenzonitrile ***(17c5)****.* White solid, yield: 63.2%, mp: 204-206℃. ^{1}H NMR (600 MHz, DMSO-d_6) δ 9.72 (s, 1H, NH), 8.37 (d, *J* = 5.6 Hz, 1H, pyrimidinyl-H), 7.69 (s, 1H, PhH), 7.64 (s, 2H, PhH), 7.52 (s, 1H, PhH), 7.25 (d, *J* = 8.5 Hz, 1H, PhH), 6.54 (d, *J* = 5.6 Hz, 1H, pyrimidinyl-H), 3.22 (s, 4H, piperazinyl-4H), 2.79 (t, *J* = 4.6 Hz, 4H, piperazinyl-4H), 2.61 (ddd, *J* = 12.7, 7.9, 4.8 Hz, 1H, CH), 2.04 (s, 6H, CH_3 × 2), 0.96 (dt, *J* = 7.0, 3.4 Hz, 2H, CH_2), 0.92 – 0.83 (m, 2H, CH_2). ^{13}C NMR (150 MHz, DMSO-d_6) δ 168.39, 161.00, 159.96, 153.67, 145.32, 138.09, 133.10, 132.98, 125.88 (J_{CF} = 194 Hz), 125.33, 123.65, 119.03, 117.16 (J_{CF} = 3.8 Hz), 109.01, 98.63, 53.17, 46.85, 25.38, 16.24, 4.32. ESI-MS: m/z 573.11 $(M + H)^+$, m/z 595.13 $(M + Na)^+$, $C_{27}H_{27}F_3N_6O_3S$ (572.18). HPLC purity: 99.47% (λ = 254 nM).

(E)-3-(3,5-dimethyl-4-((2-((4-(4-(methylsulfonyl)piperazin-1-yl)-3-(trifluoromethyl)phenyl)amino)pyrimidin-4-yl)oxy)phenyl)acrylonitrile ***(18c1)****.* White solid, yield: 61.3%, mp: 220-222℃. ^{1}H NMR (400 MHz, DMSO-d_6) δ 9.78 (s, 1H, NH), 8.41 (d, *J* = 5.6 Hz, 1H, pyrimidinyl-H), 7.81 (s, 1H, PhH), 7.66 (s, 1H, PhH), 7.62 (s, *J* = 16.7 Hz, 1H, CH=), 7.51 (s, 2H, PhH), 7.28 (d, *J* = 8.7 Hz, 1H, PhH), 6.56 (d, *J* = 5.6 Hz, 1H, pyrimidinyl-H), 6.44 (d, *J* = 16.7 Hz, 1H, CH=), 3.21 (t, *J* = 4.9 Hz, piperazinyl-4H), 2.94 (s, 3H, CH_3), 2.86 (t, *J* = 4.6 Hz, piperazinyl-4H), 2.09 (s, 6H, CH_3 × 2). ^{13}C NMR (100 MHz, DMSO-d_6) δ 168.77, 160.74, 160.00, 152.02, 150.50, 145.15, 138.20, 129.72, 128.30, 125.81 (J_{CF} = 221 Hz), 125.58, 123.55, 119.32, 117.21 (J_{CF} = 5.6 Hz), 98.64, 96.73, 53.07, 46.48, 34.47, 16.52. ESI-MS: 573.4 m/z $(M + H)^+$, $C_{27}H_{27}F_3N_6O_3S$ (572.18). HPLC purity: 99.82% (λ = 254 nM).

(E)-3-(4-((2-((4-(4-(ethylsulfonyl)piperazin-1-yl)-3-(trifluoromethyl)phenyl)amino)pyrimidin-4-yl)oxy)-3,5-dimethylphenyl)acrylonitrile ***(18c2)****.* White solid, yield: 63.2%, mp: 210-212℃. ^{1}H NMR (400 MHz, DMSO-d_6) δ 9.78 (s, 1H, NH), 8.41 (d, *J* = 5.6 Hz, 1H, pyrimidinyl-H), 7.80 (s, 1H, PhH), 7.64 (d, *J* = 16.6 Hz, 2H, PhH + CH=), 7.51 (s, 2H, PhH), 7.26 (d, *J* = 8.1 Hz, 1H, PhH), 6.56 (d, *J* = 5.6 Hz, 1H, pyrimidinyl-H), 6.45 (d, *J* = 16.7 Hz, 1H, CH=), 3.27 (s, 4H, piperazinyl-4H), 3.12 (q, *J* = 7.3 Hz, 2H, CH_2), 2.90 – 2.78 (m, 4H, piperazinyl-4H),

2.09 (s, 6H, CH_3 × 2), 1.25 (t, *J* = 7.3 Hz, 3H, CH_3). ^{13}C NMR (150 MHz, DMSO-d_6) δ 168.78, 160.74, 160.02, 152.04, 150.49, 145.20, 138.18, 131.82, 131.62, 129.72, 128.75, 125.66 (J_{CF} = 175 Hz), 125.20, 123.55, 119.29, 117.27 (J_{CF} = 4.1 Hz), 98.62, 96.74, 53.41, 46.31, 43.12, 16.51, 7.99. ESI-MS: 587.13 m/z $(M + H)^+$, 609.10 m/z $(M + Na)^+$, $C_{28}H_{29}F_3N_6O_3S$ (586.20). HPLC purity: 99.79% (λ = 254 nM).

*(E)-3-(4-((2-((4-(4-(isopropylsulfonyl)piperazin-1-yl)-3-(trifluoromethyl)phenyl)amino)pyrimidin-4-yl)oxy)-3,5-dimethylphenyl)acrylonitrile (**18c3**).* White solid, yield: 68.4%, mp: 200-202°C. ^{1}H NMR (600 MHz, DMSO-d_6) δ 9.71 (s, 1H, NH), 8.34 (d, *J* = 5.6 Hz, 1H, pyrimidinyl-H), 7.72 (s, 1H, PhH), 7.59 (d, *J* = 7.6 Hz, 1H, PhH), 7.57 (d, *J* = 16.3 Hz, 2H, CH=), 7.44 (s, 2H, PhH), 7.17 (s, 1H, PhH), 6.49 (d, *J* = 5.6 Hz, 1H, pyrimidinyl-H), 6.38 (d, *J* = 16.7 Hz, 1H, CH=), 3.32 (p, *J* = 6.8 Hz, 1H, CH), 3.26 (s, 4H, piperazinyl-4H), 2.72 (t, *J* = 4.9 Hz, 4H, piperazinyl-4H), 2.02 (s, 6H, CH_3 × 2), 1.19 (d, *J* = 6.8 Hz, 6H, CH_3 × 2). ^{13}C NMR (150 MHz, DMSO-d_6) δ 168.78, 160.74, 160.02, 152.04, 150.47, 145.24, 138.15, 131.81, 131.62, 129.72, 128.74, 125.73 (J_{CF} = 181 Hz), 125.16, 123.55, 119.27, 117.28 (J_{CF} = 5.3 Hz), 98.61, 96.74, 53.83, 52.35, 46.69, 16.97, 16.50. ESI-MS: 601.15 m/z $(M + H)^+$, 623.18 m/z $(M + Na)^+$, $C_{29}H_{31}F_3N_6O_3S$ (600.21). HPLC purity: 98.88% (λ = 254 nM).

*(E)-4-(4-((4-(4-(2-cyanovinyl)-2,6-dimethylphenoxy)pyrimidin-2-yl)amino)-2-(trifluoromethyl)phenyl)-N,N-dimethylpiperazine-1-sulfonamide (**18c4**).* White solid, yield: 71.2%, mp: 246-248°C. ^{1}H NMR (400 MHz, DMSO-d_6) δ 9.78 (s, 1H, NH), 8.41 (d, *J* = 5.5 Hz, 1H, pyrimidinyl-H), 7.77 (s, 1H, PhH), 7.65 (d, *J* = 16.3 Hz, 2H, CH=), 7.51 (s, 2H, PhH), 7.19 (d, *J* = 8.4 Hz, 1H, PhH), 6.56 (d, *J* = 5.6 Hz, 1H, pyrimidinyl-H), 6.45 (d, *J* = 16.7 Hz, 1H, CH=), 3.22 (s, 4H, piperazinyl-4H), 2.81 (s, 10H, piperazinyl-4H + CH_3 × 2), 2.09 (s, 6H, CH_3 × 2). ^{13}C NMR (100 MHz, DMSO-d_6) δ 168.75, 160.80, 159.95, 152.09, 150.51, 145.12, 138.13, 131.80, 131.64, 129.73, 128.75, 125.34 (J_{CF} = 125 Hz), 125.04, 123.44, 119.32, 117.25 (J_{CF} = 6.8 Hz), 98.58, 96.71, 53.26, 47.04, 38.37, 16.52.ESI-MS: 602.13 m/z $(M + H)^+$, 624.28 m/z $(M + Na)^+$, $C_{28}H_{30}F_3N_7O_3S$ (601.21). HPLC purity: 97.02% (λ = 254 nM).

(E)-3-(4-((2-((4-(4-(cyclopropylsulfonyl)piperazin-1-yl)-3-(trifluoromethyl)phenyl)amino)pyrimidin-4-yl)oxy)-3,5-dimethylphenyl)acrylonitrile

(18c5). White solid, yield: 65.1%, mp: 240-242°C. ^{1}H NMR (400 MHz, DMSO-d_6) δ 9.70 (s, 1H, NH), 8.34 (d, J = 5.6 Hz, 1H, pyrimidinyl-H), 7.74 (s, 1H, PhH), 7.59 (s, 1H, PhH), 7.57 (d, J = 16.7 Hz, 1H, CH=), 7.43 (s, 2H, PhH), 7.21 (d, J = 8.5 Hz, 1H, PhH), 6.49 (d, J = 5.6 Hz, 1H, pyrimidinyl-H), 6.37 (d, J = 16.7 Hz, 1H, CH=), 3.21 (d, J = 6.0 Hz, 4H, piperazinyl-4H), 2.78 (t, J = 4.7 Hz, 4H, piperazinyl-4H), 2.66 – 2.57 (m, 1H, CH), 2.02 (s, 6H, CH_3 × 2), 1.01 – 0.94 (m, 2H, CH_2), 0.89 (dt, J = 5.3, 3.2 Hz, 2H, CH_2). ^{13}C NMR (100 MHz, DMSO-d_6) δ 168.77, 160.75, 160.00, 152.01, 150.51, 145.18, 138.20, 131.81, 131.60, 129.72, 128.75, 125.59 (J_{CF} = 136 Hz), 125.29, 123.55, 119.33, 117.21 (J_{CF} = 4.8 Hz), 98.64, 96.72, 53.18, 46.86, 25.25, 16.53, 4.31. ESI-MS: 599.10 m/z $(M + H)^+$, 621.27 m/z $(M + Na)^+$, $C_{29}H_{29}F_3N_6O_3S$ (598.20). HPLC purity: 99.49% (λ = 254 nM).

(E)-3-(4-((2-((4-(4-acryloylpiperazin-1-yl)-3-(trifluoromethyl)phenyl)amino)pyrimidin-4-yl)oxy)-3,5-dimethylphenyl)acrylonitrile ***(18c5)***. White solid, yield: 62.1%, mp: 254-256°C. ^{1}H NMR (400 MHz, DMSO-d_6) δ 9.69 (s, 1H, NH), 8.34 (d, J = 5.6 Hz, 1H, pyrimidinyl-H), 7.72 (s, 1H, PhH), 7.58 (s, 1H, PhH), 7.54 (s, 1H, PhH), 7.43 (s, 2H, PhH), 7.13 (d, J = 8.4 Hz, 1H, PhH), 6.74 (dd, J = 16.7, 10.5 Hz, 1H, CH=), 6.48 (d, J = 5.6 Hz, 1H, pyrimidinyl-H), 6.37 (d, J = 16.7 Hz, 1H, CH=), 6.07 (dd, J = 16.7, 2.2 Hz, 1H, CH=), 5.63 (dd, J = 10.5, 2.2 Hz, 1H, CH=), 3.57 (s, 4H, piperazinyl-4H), 2.68 (s, 4H, piperazinyl-4H), 2.02 (s, 6H, CH_3 × 2). ^{13}C NMR (100 MHz, DMSO-d_6) δ 168.76, 164.80, 160.76, 159.98, 152.05, 150.51, 145.27, 138.07, 131.81, 131.62, 128.76, 128.72, 127.79, 125.81 (J_{CF} = 146 Hz), 125.15, 123.53, 119.32, 117.25 (J_{CF} = 5.0 Hz), 98.59, 96.73, 54.06, 53.52, 46.17, 42.49, 16.52. ESI-MS: 549.22 m/z $(M + H)^+$, 571.21 m/z $(M + Na)^+$, $C_{29}H_{27}F_3N_6O_2$ (548.21). HPLC purity: 99.99% (λ = 254 nM).

Section 3. *In vitro* anti-HIV assays

Evaluation of the antiviral activity and cytotoxicity of the synthesized compounds was performed using the MTT assay as previously described.[2] Stock solutions (10 × final concentration) of test compounds were added in 25 μL volumes to two series of triplicate wells to allow simultaneous evaluation of their effects on mock- and HIV-infected cells at the beginning of each experiment. Serial five-fold dilutions of test

compounds were made directly in flat-bottomed 96-well microtiter trays by adding 100 μL medium to the 25 μL stock solution and transferring 25 μL of this solution to another well that contained 100 μL medium using a Biomek 3000 robot (Beckman Instruments, Fullerton, CA). Untreated control HIV- and mock-infected cell samples were included for each sample. HIV-1 WT strain (III_B), HIV-1 drug-resistant strains including K103N/Y181C double mutant strain (RES056), E138K, L100I, K103N, Y181C, Y188L, and F227L/V106A or HIV-2 strain (ROD) stock (50 μL) at 100- 300 CCID50 (50% cell culture infectious dose) or culture medium was added to either the infected or mock-infected wells of the microtiter tray. Mock-infected cells were used to evaluate the effect of test compounds on uninfected cells in order to assess its cytotoxicity. Exponentially growing MT-4 cells were centrifuged for 5 min at 1000 rpm and the supernatant was discarded. The MT-4 cells were resuspended at 6×10^5 cells/mL, and 50 μL volumes were transferred to the microtiter tray wells. Five days after infection, the viability of mock- and HIV-infected cells was examined spectrophotometrically by the MTT method. The 50% cytotoxic concentration (CC_{50}) was defined as the concentration of the test compound that reduced the viability of the mock-infected MT-4 cells by 50%. The concentration achieving 50% protection from the cytopathic effect of the virus in infected cells was defined as the 50% effective concentration (EC_{50}).

Section 4. HIV-1 RT inhibition assays

A reverse transcriptase (RT) assay kit produced by Roche was selected for the RT inhibition assay.[3] All the reagents for performing the RT reaction came with the kit and the ELSIA procedures for RT inhibition assay was carried out following the description in the kit protocol. Briefly, the reaction mixture containing template/primer complex, viral nucleotides (dNTPs) and RT in the incubation buffer with or without inhibitors was incubated for 1 h at 37 °C. After that, the reaction mixture was transferred to a streptavidine coated microtiter plate and incubated for another 1 h at 37 °C to make sure retranscriptional cDNA chain that consisted biotin labeled dNTPs bound to streptavidine. Then unbound dNTPs were removed using washing buffer and anti-DIG-POD working solution was added. After incubation for 1 h at 37 °C, the DIG-labeled dNTPs incorporated in cDNA were bound to the anti-DIG-POD antibody. The unbound

anti-DIG-PODs were removed and the peroxidesub strate (ABST) solution was added to the MTPs. A colored reaction proceed during cleavage of the substrate catalyzed by POD. The absorbance of the sample was determined at OD 405 nm using a microtiter plate ELISA reader. The percentage inhibitory activity of RT inhibitors was calculated by the formula given below:

%Inhibition = [OD value with RT but without inhibitors - OD value with RTand inhibitors]/[OD value with RTand inhibitors -OD value without RT and inhibitors]

The IC_{50} values corresponded to the concentrations of the inhibitors required to inhibit biotin-dUTP incorporation by 50%.

Section 5. HIV-1 RT Crystallization and Structure Determination

An engineered HIV-1 RT construct, RT52A, here referred to as wild-type (WT) RT, was expressed and purified as described previously.[4-5] Prior to crystallization, RT52A (20 mg mL^{-1}) was incubated with **18b1** at a 1:1.5 protein:drug molar ratio at room temperature for 30 min. Co-crystals of RT with **18b1** were produced in hanging drops at 4°C with a 1:1 ratio of protein solution and well solution consisting of 10%(*v*/*v*) PEG 8000, 4%(*v*/*v*) PEG 400, 100 mM MES pH 6.3, 10 mM spermine, 15 mM $MgSO_4$, 100 mM ammonium sulfate, and 5 mM tris(2-carboxyethyl)phosphine together with an experimentally optimized concentration of microseeds from previously generated and crushed RT/rilpivirine crystals (pre-seeding). The crystals were cryo-protected by dipping them into the above solution with 25% ethylene glycol and plunge-frozen in liquid N_2. X-ray data were collected from two of the plunge-frozen crystals at the APS 23-ID-B beamline. Three isomorphous datasets (as determined with BLEND [6]) indexed with XDS [7] within the Fast DP pipeline [8] were merged and scaled with the crystallographic software packages Pointless and Aimless from the CCP4 suite [9]. The structure was solved by molecular replacement using the HIV-1 RT/**25a** complex (PDB ID 6C0N) as the template. Phenix,[10] and COOT [11] were used for structure refinement, and model building, respectively. The diffraction data and refinement statistics are summarized in Table S1.

Table S1. Data collection and refinement statistics

	**RT52A-18b1**
**PDB ID**	8FE8
**Number of datasets**	3
**Wavelength (Å)**	1.03317
**Resolution range (Å)**	29.64 - 2.5 (2.589 - 2.5)
**Space group**	C2
**Unit cell (Å | °)**	162.1 72.6 108.4 | 90.0 99.9 90.0
**Unique reflections**	43179 (4507)
**Redundancy**	21.1 (21.0)
**Completeness (%)**	99.9 (99.8)
**Mean I/sigma(I)**	9.4 (1.1)
**Wilson *B*-factor ($Å^2$)**	69.13
**R_{merge}**	0.291 (14.689)
**R_{meas}**	0.306 (15.493)
**R_{pim}**	0.093 (4.845)
**$CC_{1/2}$**	0.984 (0.652)
**R_{work}**	0.2363 (0.3599)
**R_{free}**	0.2771 (0.4161)
**Number of atoms**	8193
**Macromolecules**	8024
**Ligands**	72
**Solvent**	97
**Protein residues**	968
**RMS bonds (Å)**	0.003
**RMS angles (°)**	0.56
**Ramachandran favored (%)**	95.32
**Ramachandran allowed (%)**	4.57
**Ramachandran outliers (%)**	0.10
**Rotamer outliers (%)**	0.57
**Clashscore**	8.19
**Average B-factor (Å2)**	100.40
**Macromolecules**	100.54
**Ligands**	102.61
**Solvent**	86.86
**Number of TLS groups**	13

Statistics for the highest-resolution shell are shown in parentheses.

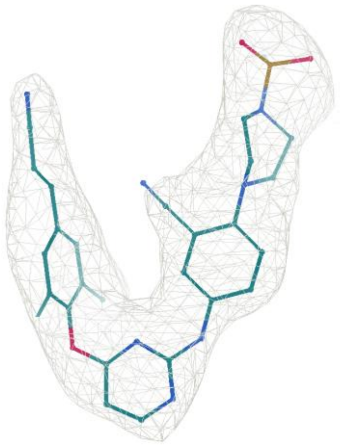

Figure S1. Electron density of compound **18b1** (calculated through a Polder omit map [12] at 4.5 σ contour) in complex with HIV-1 RT, determined at 2.5 Å resolution.

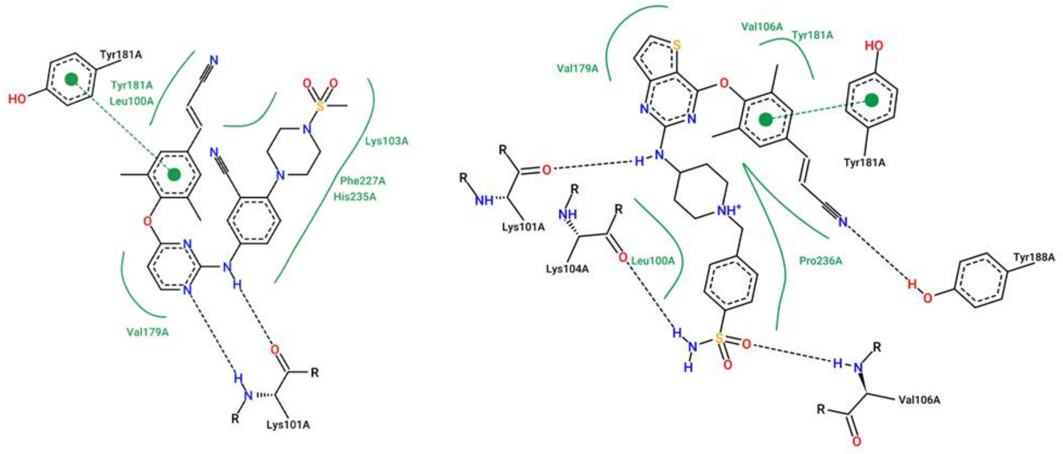

Figure S2. 2D representation of the interactions between RT-**18b1** (left) and RT-**25a** (right, PDB ID 6C0N). Created with PoseView at (https://proteins.plus/). Hydrogen bonds are represented as dashed lines, stacking contacts as green dashed lines connecting centroids (green dots), and hydrophobic contacts as green spline segment denoting the hydrophobic part of the ligand.

Section 6. Molecular dynamics simulation methods

Initial Structures Preparation. K103N RT (pdb code 6C0O with a resolution of 1.9 Å), E138K RT (pdb code 6C0P with a resolution of 2.05 Å) and V106A/F227L RT (pdb code 6DUF with a resolution of 1.96 Å) were downloaded from RCSB PDB (rcsb.org).[13] Marvin was used to sketch **18b1** (in E-configuration) and Etravirine RT inhibitors, and to check their ionization states, Marvin 20.21, ChemAxon (chemaxon.com). No ionisable groups were detected at physiological pH. **18b1** and Etravirine conformers were generated by OMEGA module of OPENEYE Scientific Software Inc, and then they were docked into the binding site of each RT variant by OEDOCKING 3.0.1 with chemgauss4 scoring function.[14-15] Conformers of either **18b1**

or Etravirine with the highest chemgauss4 score docked to each of the four RT variants were used (as complex of the inhibitor and RT variant) in the subsequent molecular dynamics simulation.

Molecular Dynamics Simulation Production. Atomic point charges for **18b1** and Etravirine were derived using ANTECHAMBER module of AMBER14 from AM1-BCC charge model. Force field ff14sb was used to generate parameters of the protein residues, and the GAFF force field for **18b1** and Etravirine. Complex of each inhibitor-RT variant was neutralized by Cl ions and then solvated using TIP3PBOX octahedral solvent box model with 8 Å cut. Initially, the solvent was minimized for 10000 cycles using steepest descent followed by conjugate gradient algorithms. Then, the whole system was minimized for 5000 cycles using steepest descent and then by conjugate gradient algorithms. Then, water molecules were equilibrated for 20 ps at constant volume and periodic boundaries. The whole system (water and the complex) was then equilibrated for 40 ps using constant pressure periodic boundaries. Finally, the equilibrated structure was used in 300 ns NPT MD simulation. Non-bonded forces were calculated at a Cutoff distance of 8 Å, and the SHAKE algorithm for hydrogen atoms was turned on.

Clustering. All frames were imaged using autoimage of cpptraj and then water molecules and Cl ions were removed. All frames were aligned against the first frame of the MD simulation using protein residues only. Then, all frames were clustered by DBSCAN algorithm[16] implemented in CPPTRAJ of AMBER14 on the inhibitor compound with no frame orientation (no fit), which clustered all frames according to either **18b1** or Etravirine. This will explore their binding to each RT variant.

Table S2. Hydrogen Bonds and Hydrophobic Interactions between Inhibitors and the Three RT Variants.

	18b1						ETR					
	K103N		E138K		V106A/F227L		K103N		E138K		V106A/F227L	
**Hydrogen bonds**	K101@O	99	K101@O	99	K101@O	99	K101@O	99	K101@O	99	K101@O	100
	K101@H	22	K101@H	86	K101@H	53	K101@H	70	K101@H	88	K101@H	57
	N103@H	51	-	-	-	-	-	-	-	-	-	-
	N103@HD	24	-	-	-	-	N103@HD	22	-	-	K103@HD-N3	22
	-	-	-	-	-	-	-	-	-	-	V108@CG-N2	15
	-	-	-	-	-	-	Y188@CD2-N2	67	-	-	-	-
	-	-	-	-	-	-	Y188@CE2-N2	66	-	-	-	-
	-	-	P225@CA-O2	47	-	-	-	-	-	-	-	-
	P225@CB-O2	10	P225@CB-O2	61	-	-	-	-	-	-	-	-
	F227@CB-N2	49	F227@CB-N2	23	-	-	-	-	-	-	-	-
	-	-	F227@CE1-O2	23	-	-	-	-	-	-	-	-
	F227@CE2-O2	25	-	-	-	-	-	-	-	-	-	-
	F227@CD1-N2	22	F227@CD1-O2	11	-	-	F227@CD2-N2	13	F227@CD1-N1	32	L227@CD1	22
	F227@CZ-O2	16	-	-	-	-	-	-	-	-	-	-
	-	-	-	-	-	-	-	-	W229@CB-N2	63	-	-
	-	-	-	-	-	-	L234@CB-N1	22	L234@CB-N1	19	L234@CB-N1	14
	-	-	L234@O-C23	68	-	-	-	-	-	-	-	-
	-	-	L234@O-C21	47	-	-	-	-	-	-	-	-
	-	-			-	-	H235@H-N1	60	-	-	-	-
	-	-	-	-	H235@O-C23	96	-	-	-	-	-	-
	H235@O-C24	86	-	-	-	-	-	-	-	-	-	-
	H235@O-C22	61	-	-	-	-	-	-	-	-	-	-
	-	-	-	-	P236@CA-N6	52	-	-	P236@CA-N1	49	-	-
			-	-	P236@O-C22	20	-	-	P236@O-C3	60	-	-
	P236@CA-N6	82	-	-			-	-	-	-	-	-
	P236@CD-O1	20	P236@CD-O1	42	-	-	P236@CD-N1	60	-	-	-	-
	P236@CG-O1	13	P236@CG-O2	58	-	-	-	-	-	-	-	-
	-	-	-	-	-	-	H235@CA-N1	60	H235@CA-N1	70	-	-
	-	-	-	-	-	-	E138@OE1	28	-	-	E138@OE1	24
	-	-	-	-	-	-	E138@OE2	23	-	-	E138@OE2	16
**Hydrophobic interactions**	-		P95		-		-		-		-	
	L100		L100		L100		L100		L100		L100	
	-		-		K103		-		K103		-	
	V106		V106		-		-		-		-	
	V179		V179		V179		V179		V179		V179	
	Y181		Y181		Y181		Y181		Y181		-	
	Y188		Y188		Y188		Y188		Y188		Y188	
	Val189		-		V189		-		-		V189	
	-		F227		L227		-		-		L227	
	W229		-		-		-		-		W229	
	L234		L234		L234		L234		-		L234	
	H235		-		-		H235		H235		-	
	P236		P236		P236		-		-		-	
	Y318		Y318		-		Y318		Y318		Y318	
	E138		-		-		-		-		-	

N1 and N2 are the nitril nitrogen atom of the right and the left wings respectively. N3 is one nitrogen atom of piperazinyl. O1 and O2 are oxygen atoms of the sulfonyl in **18b1**. Hydrogen bonds are represented with their corresponding frequencies. Amino acids are represented in FASTA format. – The interaction was not detected. C21, C22, C23 and C24 are the carbon atoms in the piperazine moiety of **18b1**. C3 is the carbon atom in the phenyl ring of ETR.

Table S3. Binding Free Energies and Their Components for 18b1 and ETR Binding to the Four RT Variants.

compound	Variant	EPB_{Ligand}	$ENPOLAR_{Ligand}$	E_{elec}	E_{vdw}	$G_{solv\text{-}polar}$	$G_{solv\text{-}non\text{-}polar}$	$\Delta G_{binding}$
	K103N	-34.8	55.9	-27.1	-72.9	52.4	-46.6	-13.4
**18b1**	E138K	-31.4	55.7	-13.4	-74.9	35.3	-46.4	-17.0
	V106/F227L	-35.0	55.7	-26.9	-76.3	54.3	-47.4	-12.8
	K103N	-23.2	41.7	-29.6	-59.7	44.3	-37.7	-18.5
**ETR**	E138K	-22.8	41.6	-9.0	-61.0	24.2	-37.2	-18.0
	V106/F227L	-24.2	41.7	-24.6	-61.4	40.6	-37.4	-17.6

EPB_{Ligand}: the electrostatic contribution to the solvation free energy of the ligand. $ENPOLAR_{Ligand}$: nonpolar contribution to the solvation free energy. E_{elec}: electrostatic energy (complex-enzyme-ligand). E_{vdw}: Van der Waals contribution (complex-enzyme-ligand). $G_{solv\text{-}polar}$: the electrostatic contribution to the solvation free energy (complex-enzyme-ligand). $G_{solv\text{-}non\text{-}polar}$: nonpolar contribution to the solvation free energy (complex-enzyme-ligand).

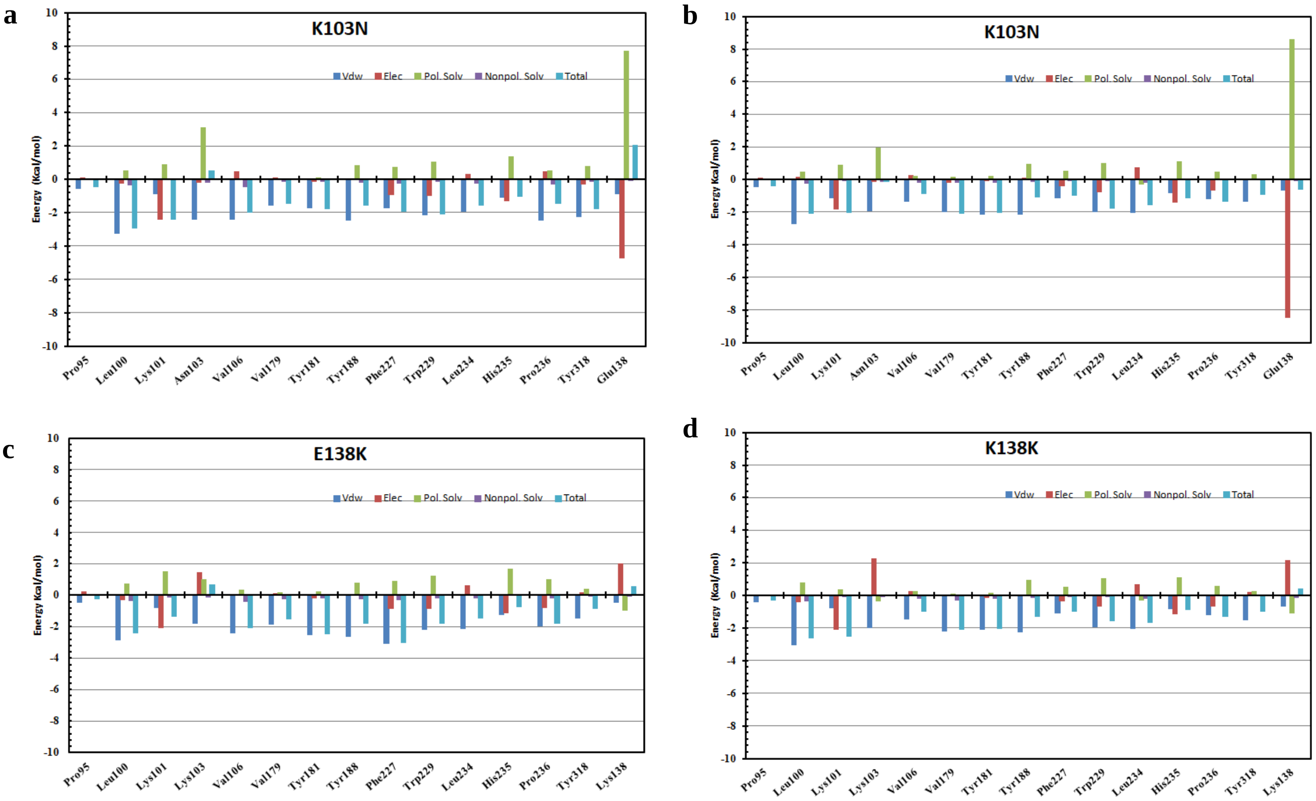

e

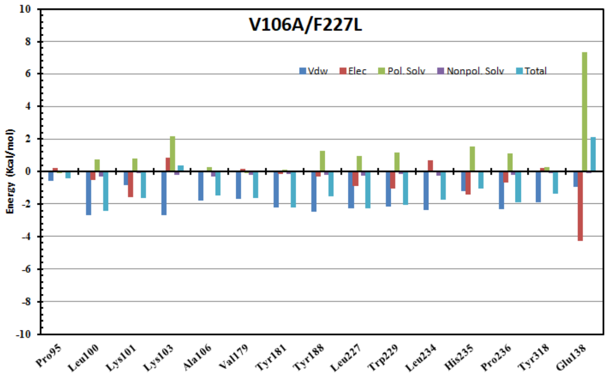

f

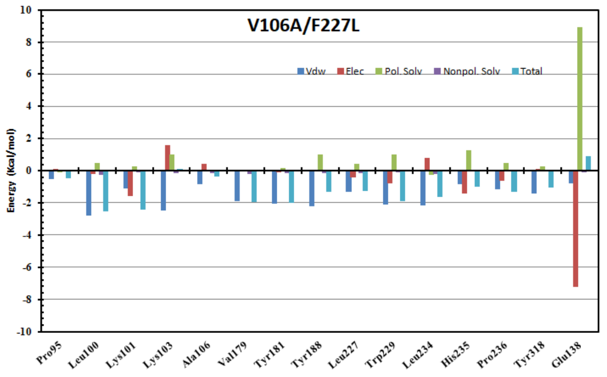

Fig. **S3** Decomposition of the binding free energy (per-residue) into contributions from electrostatic energy, *vdw energy*, polar solvation energy, and non-polar solvation energy. **a** K103N-**18b1**. **b** K103N-ETR. **c** E138K-**18b1**. **d** E138K-ETR. **e** V106A/F227L-**18b1**. **f** V106A/F227L-ETR.

Section 7. Water solubility measurements

Water solubility was measured in phosphate buffer at pH 7.0 by using an HPLC-UV method.[17] Compounds were initially dissolved in DMSO at 10 mg/mL 10 μL of this stock solution was spiked into purified water (1 mL) with the final DMSO concentration being 1%. The mixture was ultrasonic for 2 h at room temperature and restored at room temperature overnight. The saturated solution was filtrated through a filter membrane (pore size = 0.22 μm) and transferred to other Eppendorf tubes for analysis by HPLC-UV. The sample was performed in triplicate. For quantification, a model LC-20AT HPLC-UV (SHIMADZU) system was used with an Inertsil ® ODS-SP-C18 column (150 mm × 4.6 mm, 5 μm) and MeOH/water as eluant. The flow rate was 1.0 mL/min, and injection volume was 20 μL. Aqueous concentration was determined by comparison of the peak area of the saturated solution with a standard curve plotted peak area versus known concentrations, which were prepared by solutions in MeOH at 200, 40, 8.0, 1.6, and 0.32 μg/mL.

Section 8. CYP enzyme inhibition assay

Different concentrations of **18b1** (0, 0.05, 0.15, 0.5, 1.5, 5.0, 15, and 50 μmol/L) were incubated with human liver microsome (0.25 mg/mL) and NADPH (10 mmol/L) in the present of CYP1A2 probe substrate phenacetin (100 μmol/L), CYP2C9 probe substrate diclofenac (50 μmol/L), CYP2C19 probe substrate S-mephenytoin (300 μ mol/L), CYP2D6 probe substrate dextromethorphan (50 μmol/L), and CYP3A4M probe substrate midazolam (20 μmol/L) for 10 min at 37°C, respectively. Then the

corresponding selective CYP enzyme inhibitors were screened alongside **18b1** as positive controls. And the cold acetonitrile solution (400 μL) containing 200 ng/mL tolbutamide and 200 ng/mL labetalol as the internal standard was used for terminating the assay. Positive inhibitors and duplicates of **18b1** for each concentrations were performed in parallel.

Section 9. Pharmacokinetics studies

All animal treatments were performed strictly in accordance with the institutional guidelines of Animal Care and Use Committee at Shandong University, after gaining approval from the Animal Ethical and Welfare Committee (AEWC). Ten male SpragueDawley (SD) rats (180−200 g) were randomly divided into two groups to receive oral administration (10 mg/kg) or intravenous (2 mg/kg). A solution of compound **18b1** was prepared by dissolving in a mixture of polyethylene glycol (PEG) 400/normal saline (70/30, V/V) before the experiment. Blood samples (200 μL of blood each times) of the intravenous group were collected from the sinus jugular into heparinized centrifugation tubes at 5 min, 15 min, 30 min, 1 h, 2 h, 4 h, 6 h, 8 h and 12 h after dosing, and blood samples (200 μL of blood each times) of the oral administration group were collected at 5 min, 15 min, 30 min, 1 h, 2 h, 4 h, 6 h, 8 h and 12 h after dosing. All the plasma samples were obtained by centrifugation at 8000 rpm for 8 min, and immediately stored at −80 °C until analysis. LC-MS/MS analysis was used to determine the concentration of **18b1** in plasma. Briefly, 50 μL of plasma was added to 50 μL of internal standard and 300 μL of methanol in a 5 mL centrifugation tube, which was centrifuged at 3000g for 10 min. The supernatant layer was collected and a 20 μL aliquot was injected for LCMS/MS analysis. Standard curves for **18b1** in blood were generated by the addition of S10 various concentrations of **18b1** together with an internal standard to blank plasma. Then all samples were quantified with an Agilent 1200 LC/MSD (Agilent, USA). The mobile phase was methanol/1.5% glacial acetic acid (50/50, V/V) at a flow rate of 1.0 mL/min and the test wavelength was 225 nm. All blood samples were centrifuged in an Eppendorf 5415D centrifuge and quantified by Agilent 1200 LC/MSD (Agilent, USA).

Supplementary References

1. Lansdon, E. B.; Brendza, K. M.; Hung, M.; Wang, R.; Mukund, S.; Jin, D. B.; Birkus, G.; Kutty, N.; Liu, X. H., Crystal Structures of HIV-1 Reverse Transcriptase with Etravirine (TMC125) and Rilpivirine (TMC278): Implications for Drug Design. *J Med Chem* **2010,** *53* (10), 4295-4299.
2. Pauwels, R.; Balzarini, J.; Baba, M.; Snoeck, R.; Schols, D.; Herdewijn, P.; Desmyter, J.; De Clercq, E., Rapid and automated tetrazolium-based colorimetric assay for the detection of anti-HIV compounds. *J Virol Methods* **1988,** *20* (4), 309-21.
3. Suzuki, K.; Craddock, B. P.; Okamoto, N.; Kano, T.; Steigbigel, R. T., Poly A-linked colorimetric microtiter plate assay for HIV reverse transcriptase. *J Virol Methods* **1993,** *44* (2-3), 189-98.
4. Bauman, J. D.; Das, K.; Ho, W. C.; Baweja, M.; Himmel, D. M.; Clark, A. D., Jr.; Oren, D. A.; Boyer, P. L.; Hughes, S. H.; Shatkin, A. J.; Arnold, E., Crystal engineering of HIV-1 reverse transcriptase for structure-based drug design. *Nucleic Acids Res* **2008,** *36* (15), 5083-92.
5. Das, K.; Bauman, J. D.; Clark, A. D., Jr.; Frenkel, Y. V.; Lewi, P. J.; Shatkin, A. J.; Hughes, S. H.; Arnold, E., High-resolution structures of HIV-1 reverse transcriptase/TMC278 complexes: strategic flexibility explains potency against resistance mutations. *Proceedings of the National Academy of Sciences of the United States of America* **2008,** *105* (5), 1466-71.
6. Foadi, J.; Aller, P.; Alguel, Y.; Cameron, A.; Axford, D.; Owen, R. L.; Armour, W.; Waterman, D. G.; Iwata, S.; Evans, G., Clustering procedures for the optimal selection of data sets from multiple crystals in macromolecular crystallography. *Acta Crystallogr D Biol Crystallogr* **2013,** *69* (Pt 8), 1617-32.
7. Kabsch, W., Xds. *Acta Crystallogr D Biol Crystallogr* **2010,** *66* (Pt 2), 125-32.
8. Winter, G.; McAuley, K. E., Automated data collection for macromolecular crystallography. *Methods* **2011,** *55* (1), 81-93.
9. Winn, M. D.; Ballard, C. C.; Cowtan, K. D.; Dodson, E. J.; Emsley, P.; Evans, P. R.; Keegan, R. M.; Krissinel, E. B.; Leslie, A. G.; McCoy, A.; McNicholas, S. J.; Murshudov, G. N.; Pannu, N. S.; Potterton, E. A.; Powell, H. R.; Read, R. J.; Vagin, A.; Wilson, K. S., Overview of the CCP4 suite and current developments. *Acta Crystallogr D Biol Crystallogr* **2011,** *67* (Pt 4), 235-42.
10. Adams, P. D.; Afonine, P. V.; Bunkoczi, G.; Chen, V. B.; Davis, I. W.; Echols, N.; Headd, J. J.; Hung, L. W.; Kapral, G. J.; Grosse-Kunstleve, R. W.; McCoy, A. J.; Moriarty, N. W.; Oeffner, R.; Read, R. J.; Richardson, D. C.; Richardson, J. S.; Terwilliger, T. C.; Zwart, P. H., PHENIX: a comprehensive Python-based system for macromolecular structure solution. *Acta Crystallogr D Biol Crystallogr* **2010,** *66* (Pt 2), 213-21.
11. Emsley, P.; Cowtan, K., Coot: model-building tools for molecular graphics. *Acta crystallographica. Section D, Biological crystallography* **2004,** *60* (Pt 12 Pt 1), 2126-32.
12. Liebschner, D.; Afonine, P. V.; Moriarty, N. W.; Poon, B. K.; Sobolev, O. V.; Terwilliger, T. C.; Adams, P. D., Polder maps: improving OMIT maps by excluding bulk solvent. *Acta Crystallogr D Struct Biol* **2017,** *73* (Pt 2), 148-157.
13. Yang, Y.; Kang, D. W.; Nguyen, L. A.; Smithline, Z. B.; Pannecouque, C.; Zhan, P.; Liu, X. Y.; Steitz, T. A., Structural basis for potent and broad inhibition of HIV-1 RT by thiophene[3,2-d] pyrimidine non-nucleoside inhibitors. *Elife* **2018,** *7*.
14. Kelley, B. P.; Brown, S. P.; Warren, G. L.; Muchmore, S. W., POSIT: Flexible Shape-Guided Docking For Pose Prediction. *J Chem Inf Model* **2015,** *55* (8), 1771-1780.
15. McGann, M., FRED Pose Prediction and Virtual Screening Accuracy. *J Chem Inf Model* **2011,** *51* (3), 578-596.

16. Shao, J. Y.; Tanner, S. W.; Thompson, N.; Cheatham, T. E., Clustering molecular dynamics trajectories: 1. Characterizing the performance of different clustering algorithms. *J Chem Theory Comput* **2007,** *3* (6), 2312-2334.
17. Sun, L. Q.; Zhu, L.; Qian, K. D.; Qin, B. J.; Huang, L.; Chen, C. H.; Lee, K. H.; Xie, L., Design, Synthesis, and Preclinical Evaluations of Novel 4-Substituted 1,5-Diarylanilines as Potent HIV-1 Non-Nucleoside Reverse Transcriptase Inhibitor (NNRTI) Drug Candidates. *J Med Chem* **2012,** *55* (16), 7219-7229.
