# Supplementary material for: Discovery of diarylpyrimidine derivatives bearing piperazine sulfonyl as potent HIV-1 nonnucleoside reverse transcriptase inhibitors
Source: Commun Chem. 2023 Apr 29;6:83. doi: 10.1038/s42004-023-00888-4 (PMC10148624; doi:10.1038/s42004-023-00888-4)
Supplement: Supplementary file 3 — Description of Additional Supplementary Files [file 42004_2023_888_MOESM3_ESM.pdf]

Description of Additional Supplementary Files

File name: Supplementary Data 1

Description: PDB File.

File name: Supplementary Data 2

Description: Original spectra of compounds
