# Supplementary material for: Discovery of diarylpyrimidine derivatives bearing piperazine sulfonyl as potent HIV-1 nonnucleoside reverse transcriptase inhibitors
Source: Commun Chem. 2023 Apr 29;6:83. doi: 10.1038/s42004-023-00888-4 (PMC10148624; doi:10.1038/s42004-023-00888-4)
Supplement: Supplementary file 4 — Supplementary Data 1 [file 42004_2023_888_MOESM4_ESM.pdf]

Preliminary Full wwPDB X-ray Structure Validation Report (i)

Dec 5, 2022 – 10:09 PM EST

Deposition ID : D_1000270532

This wwPDB validation report is NOT for manuscript review

This is a Preliminary Full wwPDB X-ray Structure Validation Report.

This report is produced by the wwPDB Deposition System during initial deposition but before annotation of the structure.

We welcome your comments at *validation@mail.wwpdb.org*
A user guide is available at
https://www.wwpdb.org/validation/2017/XrayValidationReportHelp
with specific help available everywhere you see the (i) symbol.

The types of validation reports are described at
http://www.wwpdb.org/validation/2017/FAQs#types.

Not For Manuscript Review

The following versions of software and data (see references (i)) were used in the production of this report:

MolProbity	:	4.02b-467
Mogul	:	1.8.5 (274361), CSD as541be (2020)
Xtriage (Phenix)	:	1.13
EDS	:	2.31.3
Percentile statistics	:	20191225.v01 (using entries in the PDB archive December 25th 2019)
Refmac	:	5.8.0158
CCP4	:	7.0.044 (Gargrove)
Ideal geometry (proteins)	:	Engh & Huber (2001)
Ideal geometry (DNA, RNA)	:	Parkinson et al. (1996)
Validation Pipeline (wwPDB-VP)	:	2.31.3

1 Overall quality at a glance (i)

The following experimental techniques were used to determine the structure:
X-RAY DIFFRACTION

The reported resolution of this entry is 2.50 Å.

Percentile scores (ranging between 0-100) for global validation metrics of the entry are shown in the following graphic. The table shows the number of entries on which the scores are based.

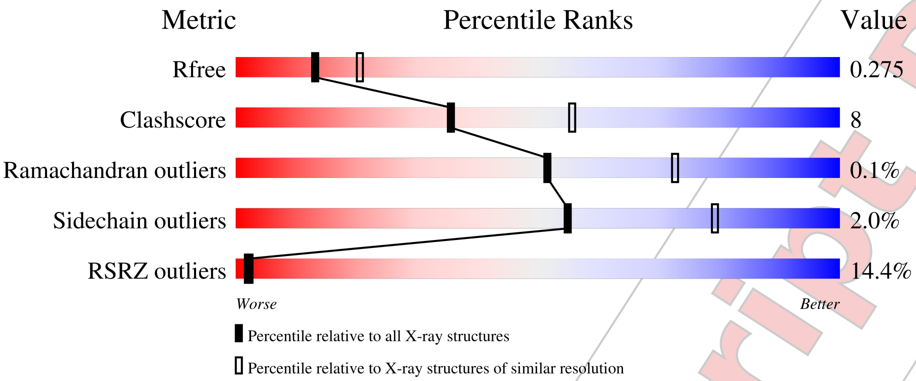

Metric	Whole archive (#Entries)	Similar resolution (#Entries, resolution range(Å))
R_{free}	130704	4661 (2.50-2.50)
Clashscore	141614	5346 (2.50-2.50)
Ramachandran outliers	138981	5231 (2.50-2.50)
Sidechain outliers	138945	5233 (2.50-2.50)
RSRZ outliers	127900	4559 (2.50-2.50)

The table below summarises the geometric issues observed across the polymeric chains and their fit to the electron density. The red, orange, yellow and green segments of the lower bar indicate the fraction of residues that contain outliers for >=3, 2, 1 and 0 types of geometric quality criteria respectively. A grey segment represents the fraction of residues that are not modelled. The numeric value for each fraction is indicated below the corresponding segment, with a dot representing fractions <=5% The upper red bar (where present) indicates the fraction of residues that have poor fit to the electron density. The numeric value is given above the bar.

Mol	Chain	Length	Quality of chain
1	A	556	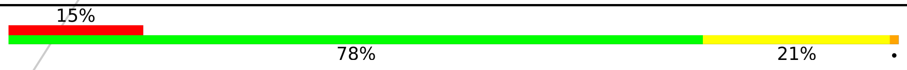
2	B	412	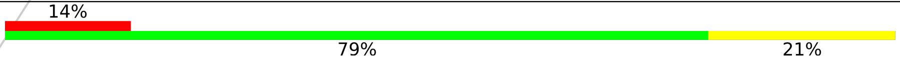

Not For Manuscript Review

2 Entry composition (i)

There are 6 unique types of molecules in this entry. The entry contains 8193 atoms, of which 0 are hydrogens and 0 are deuteriums.

In the tables below, the ZeroOcc column contains the number of atoms modelled with zero occupancy, the AltConf column contains the number of residues with at least one atom in alternate conformation and the Trace column contains the number of residues modelled with at most 2 atoms.

- Molecule 1 is a protein.

Mol	Chain	Residues	Atoms					ZeroOcc	AltConf	Trace
			Total	C	N	O	S			
1	A	556	4568	2960	758	842	8	0	6	0

- Molecule 2 is a protein.

Mol	Chain	Residues	Atoms					ZeroOcc	AltConf	Trace
			Total	C	N	O	S			
2	B	412	3456	2253	571	625	7	0	6	0

- Molecule 3 is a ligand with the chemical component id 18B but its atom names do not match the existing wwPDB Chemical Component Dictionary definition for 18B. Consequently no firm identification of ligand chemistry can be made. Once the structure is annotated then an identification and diagram will be given here.

Mol	Chain	Residues	Atoms					ZeroOcc	AltConf
			Total	C	N	O	S		
3	A	1	38	27	7	3	1	0	0

- Molecule 4 is 1,2-ETHANEDIOL (three-letter code: EDO) (formula: $C_2H_6O_2$).

Not For Manuscript Review

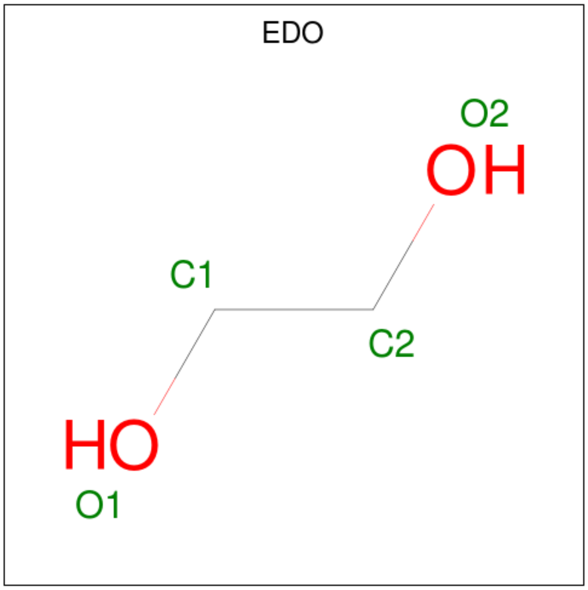

Mol	Chain	Residues	Atoms	ZeroOcc	AltConf
4	B	1	Total C O<br>4 2 2	0	0

- Molecule 5 is SULFATE ION (three-letter code: SO4) (formula: O_4S).

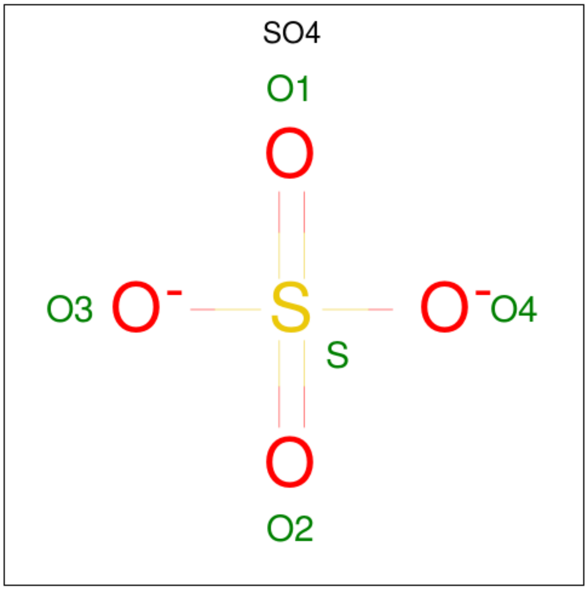

Mol	Chain	Residues	Atoms	ZeroOcc	AltConf
5	C	1	Total O S<br>5 4 1	0	0
5	C	1	Total O S<br>5 4 1	0	0
5	C	1	Total O S<br>5 4 1	0	0

Continued on next page...

Not For Manuscript Review

Continued from previous page...

Mol	Chain	Residues	Atoms	ZeroOcc	AltConf
5	C	1	Total O S<br>5 4 1	0	0
5	C	1	Total O S<br>5 4 1	0	0
5	C	1	Total O S<br>5 4 1	0	0

- Molecule 6 is water.

Mol	Chain	Residues	Atoms	ZeroOcc	AltConf
6	S	97	Total O<br>97 97	0	0

Not For Manuscript Review

3 Residue-property plots (i)

These plots are drawn for all protein, RNA, DNA and oligosaccharide chains in the entry. The first graphic for a chain summarises the proportions of the various outlier classes displayed in the second graphic. The second graphic shows the sequence view annotated by issues in geometry and electron density. Residues are color-coded according to the number of geometric quality criteria for which they contain at least one outlier: green = 0, yellow = 1, orange = 2 and red = 3 or more. A red dot above a residue indicates a poor fit to the electron density (RSRZ > 2). Stretches of 2 or more consecutive residues without any outlier are shown as a green connector. Residues present in the sample, but not in the model, are shown in grey.

- Molecule 1:

Chain A:

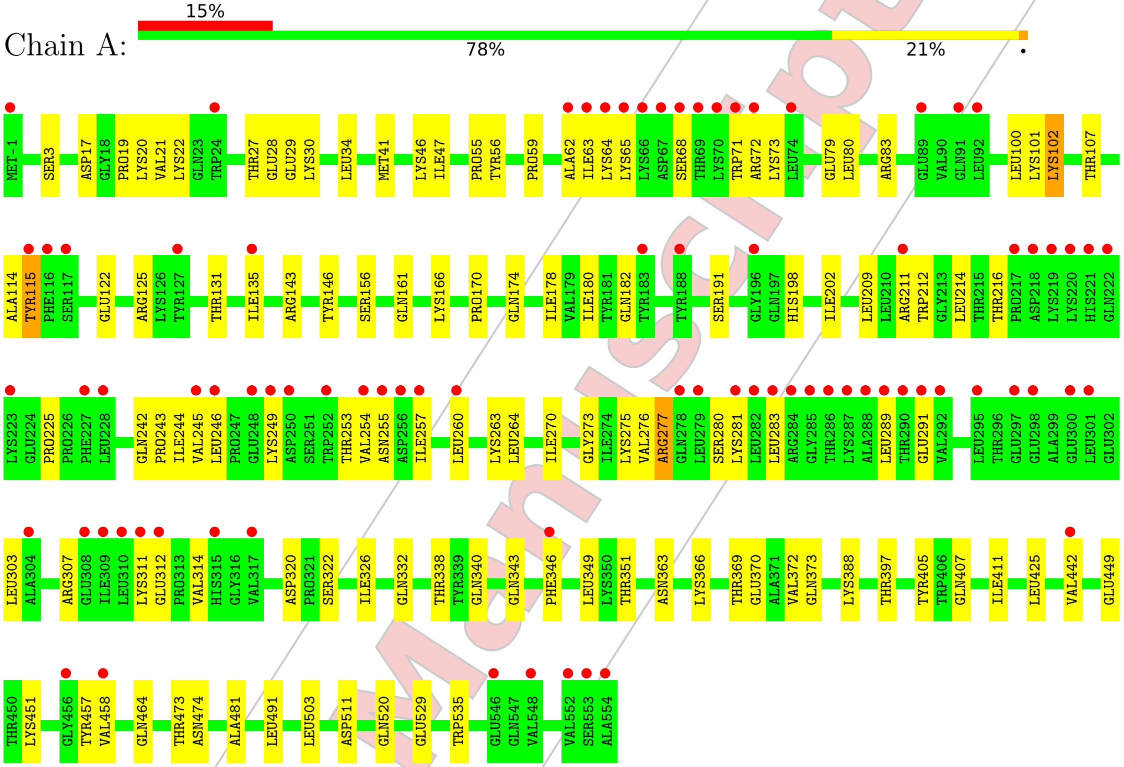

- Molecule 2:

Chain B:

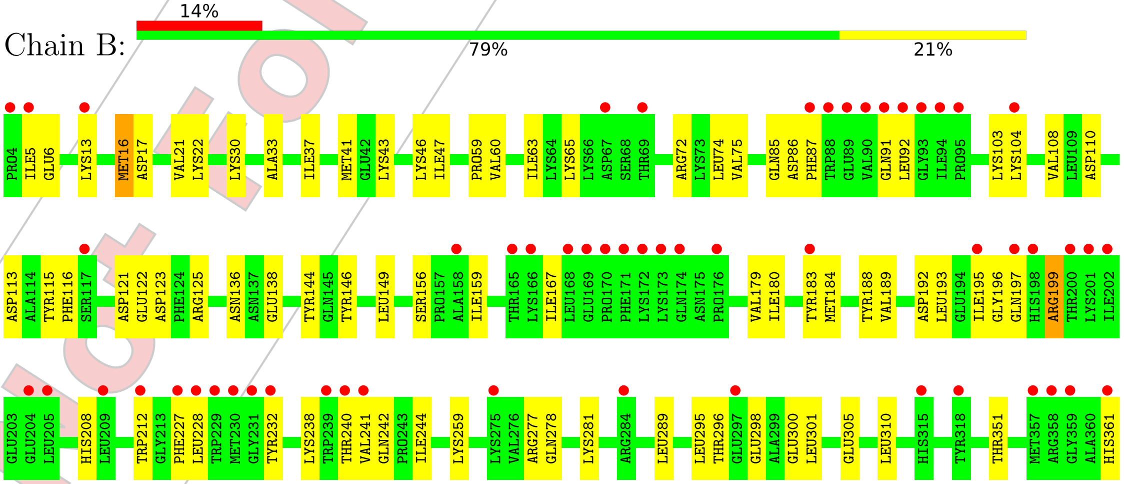

Not For Manuscript Review

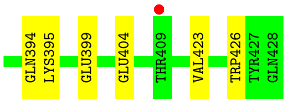

Not For Manuscript Review

4 Data and refinement statistics (i)

Property	Value	Source
Space group	C 1 2 1	Depositor
Cell constants<br>a, b, c, α, β, γ	162.08Å 72.61Å 108.43Å<br>90.00° 99.85° 90.00°	Depositor
Resolution (Å)	29.64 – 2.50<br>29.64 – 2.50	Depositor<br>EDS
% Data completeness<br>(in resolution range)	99.2 (29.64-2.50)<br>99.2 (29.64-2.50)	Depositor<br>EDS
R_{merge}	(Not available)	Depositor
R_{sym}	(Not available)	Depositor
$< I/\sigma(I) >$ [1]	1.46 (at 2.51Å)	Xtriage
Refinement program	phenix.refine 1.19.2_4158, PHENIX 1.19.2_4158	Depositor
R, R_{free}	0.236 , 0.277<br>0.236 , 0.275	Depositor<br>DCC
R_{free} test set	2038 reflections (4.76%)	wwPDB-VP
Wilson B-factor (Å^2)	69.1	Xtriage
Anisotropy	0.342	Xtriage
Bulk solvent k_{sol}(e/Å^3), B_{sol}(Å^2)	0.30 , 61.3	EDS
L-test for twinning[2]	$< \|L\| > = 0.50$, $< L^2 > = 0.33$	Xtriage
Estimated twinning fraction	No twinning to report.	Xtriage
F_o,F_c correlation	0.94	EDS
Total number of atoms	8193	wwPDB-VP
Average B, all atoms (Å^2)	100.0	wwPDB-VP

Xtriage's analysis on translational NCS is as follows: *The largest off-origin peak in the Patterson function is 5.12% of the height of the origin peak. No significant pseudotranslation is detected.*

[1] Intensities estimated from amplitudes.

[2] Theoretical values of $< |L| >$, $< L^2 >$ for acentric reflections are 0.5, 0.333 respectively for untwinned datasets, and 0.375, 0.2 for perfectly twinned datasets.

Not For Manuscript Review

5 Model quality (i)

5.1 Standard geometry (i)

Bond lengths and bond angles in the following residue types are not validated in this section: SO4, 18B, EDO

The Z score for a bond length (or angle) is the number of standard deviations the observed value is removed from the expected value. A bond length (or angle) with $|Z| > 5$ is considered an outlier worth inspection. RMSZ is the root-mean-square of all Z scores of the bond lengths (or angles).

Mol	Chain	Bond lengths		Bond angles	
		RMSZ	#$|Z|$ >5	RMSZ	#$|Z|$ >5
1	A	0.24	0/4688	0.47	0/6374
2	B	0.25	0/3555	0.47	0/4827
All	All	0.25	0/8243	0.47	0/11201

There are no bond length outliers.

There are no bond angle outliers.

There are no chirality outliers.

There are no planarity outliers.

5.2 Too-close contacts (i)

In the following table, the Non-H and H(model) columns list the number of non-hydrogen atoms and hydrogen atoms in the chain respectively. The H(added) column lists the number of hydrogen atoms added and optimized by MolProbity. The Clashes column lists the number of clashes within the asymmetric unit, whereas Symm-Clashes lists symmetry-related clashes.

Mol	Chain	Non-H	H(model)	H(added)	Clashes	Symm-Clashes
1	A	4568	0	4627	78	0
2	B	3456	0	3488	54	0
3	A	38	0	0	2	0
4	B	4	0	6	0	0
5	C	30	0	0	1	0
6	S	97	0	0	6	0
All	All	8193	0	8121	132	0

The all-atom clashscore is defined as the number of clashes found per 1000 atoms (including hydrogen atoms). The all-atom clashscore for this structure is 8.

All (132) close contacts within the same asymmetric unit are listed below, sorted by their clash

Not For Manuscript Review

magnitude.

Atom-1	Atom-2	Interatomic distance (Å)	Clash overlap (Å)
2:B:149:LEU:HD23	2:B:156:SER:HA	1.37	1.03
1:A:122:GLU:HA	1:A:125:ARG:HD2	1.69	0.75
1:A:63:ILE:HB	1:A:72:ARG:HD3	1.71	0.72
2:B:122:GLU:HA	2:B:125:ARG:HE	1.55	0.72
1:A:170:PRO:O	1:A:174:GLN:HG3	1.91	0.70
1:A:73:LYS:NZ	1:A:146:TYR:OH	2.25	0.70
1:A:56:TYR:O	1:A:143:ARG:NH2	2.27	0.68
1:A:34:LEU:HD21	1:A:62:ALA:HB2	1.80	0.64
1:A:254:VAL:HB	1:A:283:LEU:CD2	2.29	0.62
2:B:30:LYS:NZ	2:B:404:GLU:OE1	2.31	0.62
2:B:72:ARG:HH11	2:B:72:ARG:HG2	1.65	0.62
1:A:246:LEU:HD12	1:A:307:ARG:HD3	1.82	0.61
1:A:372:VAL:HG11	1:A:411[A]:ILE:HG23	1.82	0.60
2:B:259:LYS:NZ	6:S:3:HOH:O	2.35	0.60
1:A:79:GLU:OE2	1:A:83:ARG:NH1	2.35	0.59
1:A:65:LYS:HB2	1:A:68:SER:HB2	1.84	0.59
2:B:108:VAL:HB	2:B:232:TYR:HB3	1.85	0.58
2:B:91:GLN:HG3	2:B:92:LEU:H	1.69	0.58
1:A:275:LYS:HE2	1:A:332:GLN:NE2	2.19	0.58
2:B:278:GLN:HE21	2:B:298:GLU:HG3	1.70	0.57
2:B:361:HIS:CD2	6:S:50:HOH:O	2.57	0.57
2:B:193:LEU:HD13	2:B:197:GLN:HB3	1.86	0.56
2:B:41:MET:HB3	2:B:47:ILE:HG12	1.88	0.56
1:A:209:LEU:HD13	1:A:216:THR:HG21	1.87	0.56
1:A:178:ILE:HG22	1:A:191:SER:HB3	1.86	0.56
2:B:278:GLN:NE2	2:B:298:GLU:HG3	2.21	0.55
1:A:19:PRO:HG3	1:A:80:LEU:HB2	1.90	0.54
2:B:196:GLY:HA2	2:B:199:ARG:HE	1.73	0.54
1:A:47:ILE:HG22	1:A:146:TYR:HA	1.89	0.53
1:A:115:TYR:OH	1:A:156:SER:HB3	2.08	0.53
1:A:245:VAL:H	1:A:263:LYS:HZ2	1.57	0.53
1:A:366:LYS:O	1:A:370:GLU:HG3	2.10	0.52
1:A:27:THR:OG1	1:A:30:LYS:HG3	2.08	0.52
1:A:369:THR:HG23	1:A:411[A]:ILE:HD11	1.92	0.51
2:B:149:LEU:HD23	2:B:156:SER:CA	2.27	0.51
1:A:320:ASP:OD1	1:A:322:SER:OG	2.26	0.51
1:A:458:VAL:HG12	1:A:464:GLN:HG2	1.92	0.51
2:B:13:LYS:HB2	2:B:16:MET:SD	2.51	0.51
2:B:110:ASP:HB3	2:B:228:LEU:HD23	1.92	0.51
1:A:29:GLU:OE2	1:A:71:TRP:NE1	2.42	0.51
1:A:260:LEU:HG	1:A:264:LEU:HD11	1.93	0.50

Continued on next page...

Not For Manuscript Review

Continued from previous page...

Atom-1	Atom-2	Interatomic distance (Å)	Clash overlap (Å)
2:B:60[B]:VAL:HG12	2:B:75:VAL:HG22	1.94	0.50
2:B:103:LYS:HE2	2:B:179:VAL:HG23	1.92	0.50
1:A:114:ALA:HA	1:A:214:LEU:HD12	1.94	0.50
1:A:277:ARG:HH11	1:A:281:LYS:HD2	1.77	0.50
1:A:21:VAL:HB	1:A:59:PRO:HD3	1.92	0.50
2:B:46:LYS:HD3	2:B:116:PHE:HB3	1.94	0.50
2:B:180:ILE:HG12	2:B:189:VAL:HG13	1.92	0.50
1:A:178:ILE:HD13	1:A:180:ILE:HD11	1.94	0.49
2:B:296:THR:OG1	2:B:298:GLU:HG2	2.12	0.49
1:A:273:GLY:HA2	1:A:338:THR:HG21	1.93	0.49
2:B:394:GLN:HG2	6:S:32:HOH:O	2.13	0.48
2:B:301:LEU:O	2:B:305:GLU:HG3	2.12	0.48
1:A:326[B]:ILE:HD13	1:A:388:LYS:HB2	1.95	0.48
1:A:340:GLN:HG3	1:A:351:THR:HG22	1.94	0.48
2:B:395:LYS:O	2:B:399:GLU:HB2	2.13	0.48
2:B:195:ILE:O	2:B:199:ARG:HG3	2.13	0.48
1:A:503:LEU:HD22	1:A:535:TRP:HB2	1.94	0.48
2:B:63:ILE:HD13	2:B:74:LEU:HD12	1.96	0.48
2:B:103:LYS:HD2	2:B:192:ASP:OD1	2.14	0.47
1:A:303:LEU:O	1:A:307:ARG:HG2	2.14	0.47
2:B:91:GLN:HG3	2:B:92:LEU:N	2.30	0.46
1:A:41:MET:HG2	1:A:46:LYS:HD3	1.96	0.46
1:A:64:LYS:HE3	1:A:71:TRP:CE2	2.50	0.46
1:A:346:PHE:CD1	1:A:346:PHE:N	2.84	0.46
1:A:363:ASN:HA	1:A:511:ASP:OD1	2.16	0.46
1:A:131:THR:OG1	1:A:143:ARG:NH1	2.47	0.46
1:A:255:ASN:HB2	1:A:289:LEU:HB3	1.97	0.46
1:A:449:GLU:OE1	1:A:449:GLU:N	2.47	0.46
1:A:343:GLN:HG3	1:A:349:LEU:HD11	1.97	0.46
2:B:244:ILE:HG22	2:B:310:LEU:HD13	1.96	0.46
1:A:249:LYS:HE2	1:A:249:LYS:HB2	1.71	0.46
1:A:107:THR:OG1	1:A:198:HIS:NE2	2.38	0.46
1:A:281:LYS:HB3	1:A:281:LYS:HE2	1.64	0.45
1:A:100:LEU:HB3	3:A:601:18B:C14	2.47	0.45
1:A:369:THR:O	1:A:373:GLN:HG2	2.16	0.45
2:B:183:TYR:HB3	2:B:188:TYR:HE2	1.81	0.45
1:A:270:ILE:HG13	1:A:314[B]:VAL:HG13	1.97	0.45
1:A:28:GLU:HG3	1:A:135:ILE:HD12	1.99	0.45
1:A:198:HIS:O	1:A:202:ILE:HG12	2.17	0.45
2:B:5:ILE:HD12	2:B:6:GLU:N	2.31	0.45
2:B:33:ALA:O	2:B:37:ILE:HG12	2.16	0.45

Continued on next page...

Not For Manuscript Review

Continued from previous page...

Atom-1	Atom-2	Interatomic distance (Å)	Clash overlap (Å)
2:B:240:THR:OG1	2:B:241:VAL:N	2.49	0.45
1:A:277:ARG:O	1:A:280:SER:N	2.50	0.45
2:B:136:ASN:HB3	2:B:138:GLU:HG3	1.99	0.45
2:B:423:VAL:HA	2:B:426:TRP:CD1	2.52	0.45
1:A:101:LYS:HD2	1:A:102:LYS:N	2.32	0.44
2:B:115:TYR:OH	2:B:184:MET:O	2.29	0.44
2:B:199:ARG:HG3	2:B:199:ARG:H	1.65	0.44
1:A:17:ASP:O	1:A:83:ARG:HD3	2.17	0.44
2:B:21:VAL:HB	2:B:59:PRO:HD3	2.00	0.44
2:B:47:ILE:HD12	2:B:144:TYR:CD1	2.53	0.44
1:A:277:ARG:NH1	1:A:281:LYS:HD2	2.32	0.44
1:A:491:LEU:HB3	1:A:529:GLU:HG3	1.99	0.44
2:B:208:HIS:O	2:B:212:TRP:HD1	2.01	0.44
1:A:311:LYS:HG3	1:A:312:GLU:HG3	2.00	0.43
1:A:473:THR:OG1	1:A:474:ASN:N	2.51	0.43
2:B:121:ASP:O	2:B:125:ARG:HG3	2.18	0.43
1:A:3:SER:HB2	1:A:212:TRP:CD1	2.54	0.43
2:B:399:GLU:HG3	5:C:1:SO4:O1	2.19	0.43
1:A:63:ILE:O	1:A:72:ARG:N	2.34	0.43
1:A:178:ILE:HG22	1:A:191:SER:CB	2.47	0.43
1:A:277:ARG:O	1:A:281:LYS:HG3	2.19	0.43
1:A:245:VAL:H	1:A:263:LYS:NZ	2.16	0.43
2:B:149:LEU:HD21	2:B:159:ILE:HB	2.00	0.42
2:B:167:ILE:HG23	2:B:212:TRP:CG	2.54	0.42
2:B:85:GLN:HE22	2:B:87:PHE:HA	1.85	0.42
1:A:161:GLN:HG2	1:A:182:GLN:NE2	2.34	0.42
1:A:225:PRO:HB3	3:A:601:18B:O2	2.20	0.42
1:A:397:THR:HG23	1:A:425:LEU:HG	2.01	0.42
2:B:242:GLN:N	2:B:351:THR:OG1	2.49	0.42
1:A:166:LYS:HB2	1:A:166:LYS:HE3	1.80	0.42
2:B:278:GLN:HG3	2:B:298:GLU:HB2	2.01	0.42
1:A:20[B]:LYS:NZ	1:A:55:PRO:HB2	2.33	0.42
1:A:442:VAL:HG12	1:A:457:TYR:HB3	2.01	0.42
1:A:520:GLN:NE2	6:S:90:HOH:O	2.53	0.42
2:B:289:LEU:HD23	2:B:289:LEU:HA	1.86	0.42
2:B:278:GLN:HE22	2:B:281:LYS:HD3	1.83	0.41
1:A:244:ILE:HA	1:A:263:LYS:HZ2	1.85	0.41
2:B:47:ILE:HG22	2:B:146:TYR:HA	2.02	0.41
2:B:5:ILE:HD12	2:B:5:ILE:C	2.41	0.41
1:A:442:VAL:HB	1:A:481:ALA:HB1	2.01	0.41
1:A:253:THR:O	1:A:257:ILE:HG13	2.20	0.41

Continued on next page...

Not For Manuscript Review

Continued from previous page...

Atom-1	Atom-2	Interatomic distance (Å)	Clash overlap (Å)
1:A:264:LEU:HD22	1:A:276:VAL:HG12	2.03	0.41
2:B:295:LEU:HB3	2:B:300:GLU:HG3	2.03	0.41
1:A:307:ARG:HG2	1:A:307:ARG:H	1.71	0.41
2:B:65:LYS:HG3	6:S:70:HOH:O	2.21	0.41
1:A:30:LYS:HA	6:S:36:HOH:O	2.21	0.40
1:A:101:LYS:HD2	1:A:102:LYS:H	1.85	0.40
1:A:242:GLN:HB3	1:A:243:PRO:CD	2.51	0.40
1:A:405:TYR:CE2	1:A:407:GLN:HB2	2.57	0.40
2:B:121:ASP:OD1	2:B:123:ASP:N	2.51	0.40

There are no symmetry-related clashes.

5.3 Torsion angles (i)

5.3.1 Protein backbone (i)

In the following table, the Percentiles column shows the percent Ramachandran outliers of the chain as a percentile score with respect to all X-ray entries followed by that with respect to entries of similar resolution.

The Analysed column shows the number of residues for which the backbone conformation was analysed, and the total number of residues.

Mol	Chain	Analysed	Favoured	Allowed	Outliers	Percentiles	
1	A	560/556 (101%)	536 (96%)	23 (4%)	1 (0%)	47	68
2	B	414/412 (100%)	392 (95%)	22 (5%)	0	100	100
All	All	974/968 (101%)	928 (95%)	45 (5%)	1 (0%)	51	73

All (1) Ramachandran outliers are listed below:

Mol	Chain	Res	Type
1	A	291	GLU

5.3.2 Protein sidechains (i)

In the following table, the Percentiles column shows the percent sidechain outliers of the chain as a percentile score with respect to all X-ray entries followed by that with respect to entries of similar resolution.

The Analysed column shows the number of residues for which the sidechain conformation was

Not For Manuscript Review

analysed, and the total number of residues.

Mol	Chain	Analysed	Rotameric	Outliers	Percentiles	
1	A	501/495 (101%)	495 (99%)	6 (1%)	71	88
2	B	380/374 (102%)	369 (97%)	11 (3%)	42	69
All	All	881/869 (101%)	864 (98%)	17 (2%)	55	80

All (17) residues with a non-rotameric sidechain are listed below:

Mol	Chain	Res	Type
1	A	22	LYS
1	A	102	LYS
1	A	115	TYR
1	A	211	ARG
1	A	277	ARG
1	A	451	LYS
2	B	16	MET
2	B	17	ASP
2	B	22	LYS
2	B	43	LYS
2	B	86	ASP
2	B	104	LYS
2	B	113	ASP
2	B	199	ARG
2	B	227	PHE
2	B	238	LYS
2	B	277	ARG

Sometimes sidechains can be flipped to improve hydrogen bonding and reduce clashes. All (6) such sidechains are listed below:

Mol	Chain	Res	Type
1	A	182	GLN
1	A	258	GLN
1	A	336	GLN
2	B	85	GLN
2	B	182	GLN
2	B	278	GLN

5.3.3 RNA ⓘ

There are no RNA molecules in this entry.

Not For Manuscript Review

5.4 Non-standard residues in protein, DNA, RNA chains (i)

There are no non-standard protein/DNA/RNA residues in this entry.

5.5 Carbohydrates (i)

There are no monosaccharides in this entry.

5.6 Ligand geometry (i)

Of 8 ligands modelled in this entry, 1 could not be matched to an existing wwPDB Chemical Component Dictionary definition at this stage - leaving 7 for Mogul analysis.

In the following table, the Counts columns list the number of bonds (or angles) for which Mogul statistics could be retrieved, the number of bonds (or angles) that are observed in the model and the number of bonds (or angles) that are defined in the Chemical Component Dictionary. The Link column lists molecule types, if any, to which the group is linked. The Z score for a bond length (or angle) is the number of standard deviations the observed value is removed from the expected value. A bond length (or angle) with $|Z| > 2$ is considered an outlier worth inspection. RMSZ is the root-mean-square of all Z scores of the bond lengths (or angles).

Mol	Type	Chain	Res	Link	Bond lengths			Bond angles		
					Counts	RMSZ	$\#\|Z\| > 2$	Counts	RMSZ	$\#\|Z\| > 2$
5	SO4	C	4	-	4,4,4	0.14	0	6,6,6	0.08	0
5	SO4	C	2	-	4,4,4	0.13	0	6,6,6	0.09	0
5	SO4	C	1	-	4,4,4	0.14	0	6,6,6	0.10	0
5	SO4	C	5	-	4,4,4	0.40	0	6,6,6	0.05	0
5	SO4	C	6	-	4,4,4	0.14	0	6,6,6	0.06	0
5	SO4	C	3	-	4,4,4	0.14	0	6,6,6	0.08	0
4	EDO	B	501	-	3,3,3	0.45	0	2,2,2	0.29	0

In the following table, the Chirals column lists the number of chiral outliers, the number of chiral centers analysed, the number of these observed in the model and the number defined in the Chemical Component Dictionary. Similar counts are reported in the Torsion and Rings columns. '-' means no outliers of that kind were identified.

Mol	Type	Chain	Res	Link	Chirals	Torsions	Rings
4	EDO	B	501	-	-	0/1/1/1	-

There are no bond length outliers.

There are no bond angle outliers.

There are no chirality outliers.

There are no torsion outliers.

Not For Manuscript Review

There are no ring outliers.

1 monomer is involved in 1 short contact:

Mol	Chain	Res	Type	Clashes	Symm-Clashes
5	C	1	SO4	1	0

5.7 Other polymers (i)

There are no such residues in this entry.

5.8 Polymer linkage issues (i)

The following chains have linkage breaks:

Mol	Chain	Number of breaks
2	B	1

All chain breaks are listed below:

Model	Chain	Residue-1	Atom-1	Residue-2	Atom-2	Distance (Å)
1	B	213:GLY	C	227:PHE	N	7.50

Not For Manuscript Review

6 Fit of model and data (i)

6.1 Protein, DNA and RNA chains (i)

In the following table, the column labelled '#RSRZ> 2' contains the number (and percentage) of RSRZ outliers, followed by percent RSRZ outliers for the chain as percentile scores relative to all X-ray entries and entries of similar resolution. The OWAB column contains the minimum, median, 95th percentile and maximum values of the occupancy-weighted average B-factor per residue. The column labelled 'Q< 0.9' lists the number of (and percentage) of residues with an average occupancy less than 0.9.

Mol	Chain	Analysed	<RSRZ>	#RSRZ>2	OWAB(Å^2)	Q<0.9
1	A	556/556 (100%)	0.90	82 (14%) 2 2	49, 92, 189, 246	0
2	B	412/412 (100%)	0.81	57 (13%) 2 2	50, 88, 172, 259	0
All	All	968/968 (100%)	0.86	139 (14%) 2 2	49, 90, 182, 259	0

All (139) RSRZ outliers are listed below:

Mol	Chain	Res	Type	RSRZ
1	A	219	LYS	14.3
1	A	286	THR	13.7
2	B	89	GLU	11.2
2	B	92	LEU	10.5
1	A	290	THR	10.3
1	A	252	TRP	9.2
2	B	227	PHE	9.2
2	B	229	TRP	9.1
1	A	221	HIS	8.9
2	B	358	ARG	8.6
1	A	220	LYS	8.6
2	B	240	THR	7.9
1	A	62	ALA	7.9
1	A	289	LEU	7.8
1	A	66	LYS	7.5
2	B	4	PRO	7.5
1	A	71	TRP	7.3
2	B	90	VAL	7.3
2	B	239	TRP	7.2
2	B	94	ILE	7.2
1	A	257	ILE	7.1
1	A	117	SER	6.9
1	A	67	ASP	6.8
1	A	287	LYS	6.8

Continued on next page...

Not For Manuscript Review

Continued from previous page...

Mol	Chain	Res	Type	RSRZ
2	B	359	GLY	6.8
1	A	218	ASP	6.7
1	A	285	GLY	6.1
2	B	197	GLN	6.1
2	B	173	LYS	6.1
1	A	91	GLN	5.7
1	A	283	LEU	5.7
1	A	248	GLU	5.7
1	A	288	ALA	5.6
2	B	91	GLN	5.6
1	A	116	PHE	5.5
1	A	250	ASP	5.3
1	A	70	LYS	5.3
2	B	212	TRP	5.2
1	A	246	LEU	5.2
1	A	256	ASP	5.0
2	B	5	ILE	5.0
1	A	284	ARG	4.8
1	A	301	LEU	4.4
1	A	72	ARG	4.4
2	B	241	VAL	4.4
2	B	357	MET	4.4
1	A	292	VAL	4.2
1	A	295	LEU	4.2
1	A	65	LYS	4.2
1	A	63	ILE	4.1
1	A	74	LEU	4.1
1	A	92	LEU	4.1
2	B	166	LYS	4.0
1	A	282	LEU	3.9
1	A	223	LYS	3.8
1	A	304	ALA	3.8
2	B	361	HIS	3.8
2	B	232	TYR	3.8
1	A	317	VAL	3.8
1	A	222	GLN	3.7
1	A	554	ALA	3.7
2	B	315	HIS	3.7
1	A	300	GLU	3.6
2	B	169	GLU	3.6
2	B	67	ASP	3.6
1	A	24	TRP	3.6

Continued on next page...

Not For Manuscript Review

Continued from previous page...

Mol	Chain	Res	Type	RSRZ
2	B	88	TRP	3.6
1	A	254	VAL	3.5
1	A	64	LYS	3.5
1	A	297	GLU	3.4
1	A	68	SER	3.4
1	A	279	LEU	3.4
2	B	230	MET	3.4
1	A	311	LYS	3.4
1	A	309	ILE	3.4
1	A	552	VAL	3.4
2	B	176	PRO	3.3
2	B	117	SER	3.3
1	A	89	GLU	3.2
1	A	546	GLU	3.2
1	A	312	GLU	3.2
2	B	170	PRO	3.2
2	B	318	TYR	3.2
2	B	200	THR	3.2
1	A	183	TYR	3.1
2	B	172	LYS	3.1
1	A	548	VAL	3.1
2	B	87	PHE	3.0
2	B	93	GLY	3.0
1	A	553	SER	3.0
2	B	284	ARG	2.8
1	A	281	LYS	2.8
1	A	249	LYS	2.8
2	B	202	ILE	2.8
1	A	308	GLU	2.8
1	A	310	LEU	2.8
2	B	205	LEU	2.8
1	A	211	ARG	2.8
2	B	168	LEU	2.7
1	A	227	PHE	2.7
2	B	171	PHE	2.7
1	A	196	GLY	2.7
1	A	456	GLY	2.7
1	A	298	GLU	2.7
1	A	-1	MET	2.7
2	B	204	GLU	2.6
2	B	195	ILE	2.6
1	A	346	PHE	2.6

Continued on next page...

Not For Manuscript Review

Continued from previous page...

Mol	Chain	Res	Type	RSRZ
1	A	245	VAL	2.5
2	B	95	PRO	2.5
2	B	198	HIS	2.5
1	A	278	GLN	2.5
1	A	260	LEU	2.4
1	A	115	TYR	2.4
1	A	69	THR	2.4
1	A	291	GLU	2.4
2	B	209	LEU	2.4
2	B	183	TYR	2.3
1	A	315	HIS	2.3
1	A	135	ILE	2.3
2	B	231	GLY	2.3
2	B	228	LEU	2.3
2	B	201	LYS	2.2
2	B	69	THR	2.2
2	B	297	GLU	2.2
1	A	188	TYR	2.2
2	B	104	LYS	2.2
2	B	165	THR	2.2
2	B	158	ALA	2.2
2	B	409	THR	2.2
1	A	255	ASN	2.2
1	A	127	TYR	2.1
1	A	458	VAL	2.1
1	A	217	PRO	2.1
2	B	275	LYS	2.1
1	A	228	LEU	2.1
1	A	442	VAL	2.0
2	B	174	GLN	2.0
2	B	13	LYS	2.0

6.2 Non-standard residues in protein, DNA, RNA chains (i)

There are no non-standard protein/DNA/RNA residues in this entry.

6.3 Carbohydrates (i)

There are no monosaccharides in this entry.

Not For Manuscript Review

6.4 Ligands (i)

In the following table, the Atoms column lists the number of modelled atoms in the group and the number defined in the chemical component dictionary. The B-factors column lists the minimum, median, 95th percentile and maximum values of B factors of atoms in the group. The column labelled 'Q< 0.9' lists the number of atoms with occupancy less than 0.9.

Mol	Type	Chain	Res	Atoms	RSCC	RSR	B-factors($Å^2$)	Q<0.9
5	SO4	C	5	5/?	0.82	0.32	117,117,125,164	0
4	EDO	B	501	4/?	0.88	0.33	71,73,77,88	0
5	SO4	C	6	5/?	0.89	0.34	90,94,132,133	0
3	18B	A	601	38/?	0.91	0.20	87,98,127,131	0
5	SO4	C	1	5/?	0.92	0.17	72,84,97,98	0
5	SO4	C	3	5/?	0.93	0.14	115,121,131,137	0
5	SO4	C	2	5/?	0.96	0.22	80,87,96,105	0
5	SO4	C	4	5/?	0.96	0.25	90,90,102,105	0

6.5 Other polymers (i)

There are no such residues in this entry.

Not For Manuscript Review
