# Supplementary material for: Discovery of diarylpyrimidine derivatives bearing piperazine sulfonyl as potent HIV-1 nonnucleoside reverse transcriptase inhibitors
Source: Commun Chem. 2023 Apr 29;6:83. doi: 10.1038/s42004-023-00888-4 (PMC10148624; doi:10.1038/s42004-023-00888-4)
Supplement: Supplementary file 5 — Supplementary Data 2 [file 42004_2023_888_MOESM5_ESM.pdf]

Original spectra of compounds

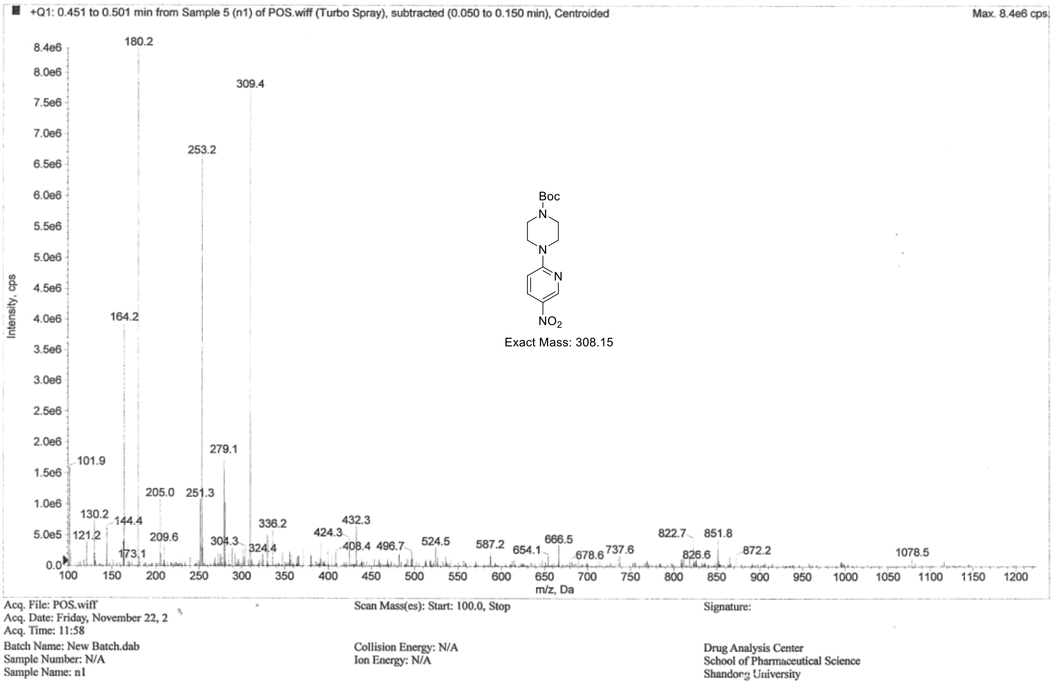

MS spectrum of **7a**

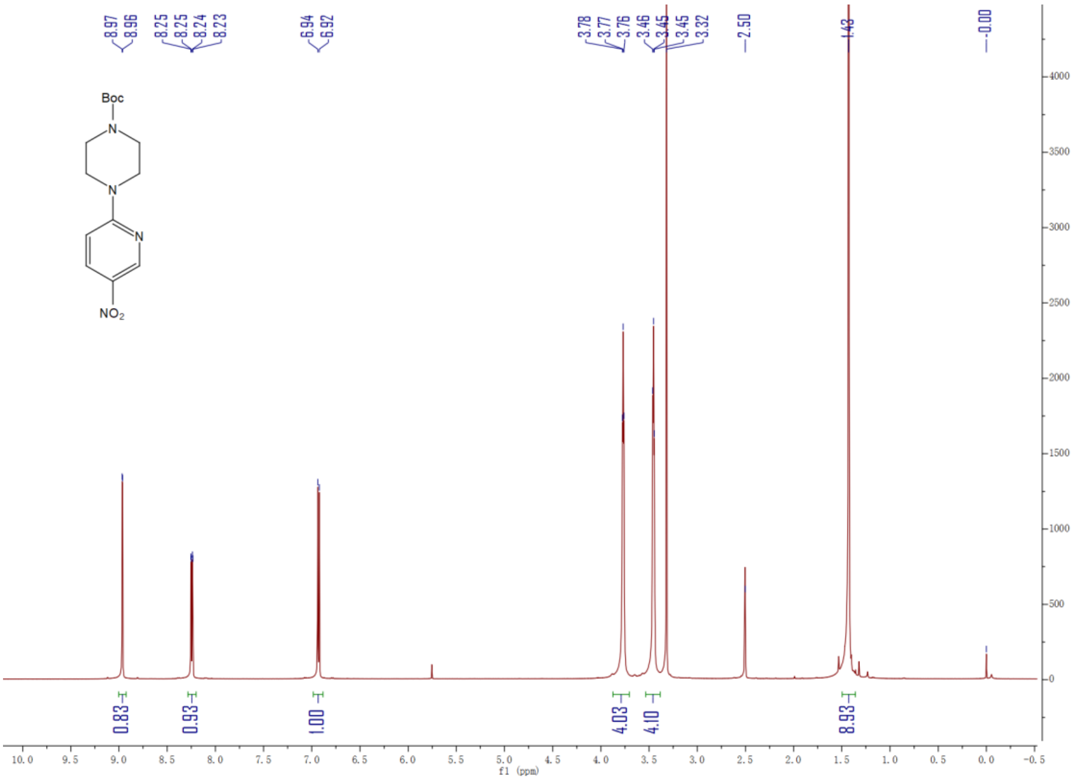

^{1}H NMR spectrum of **7a**

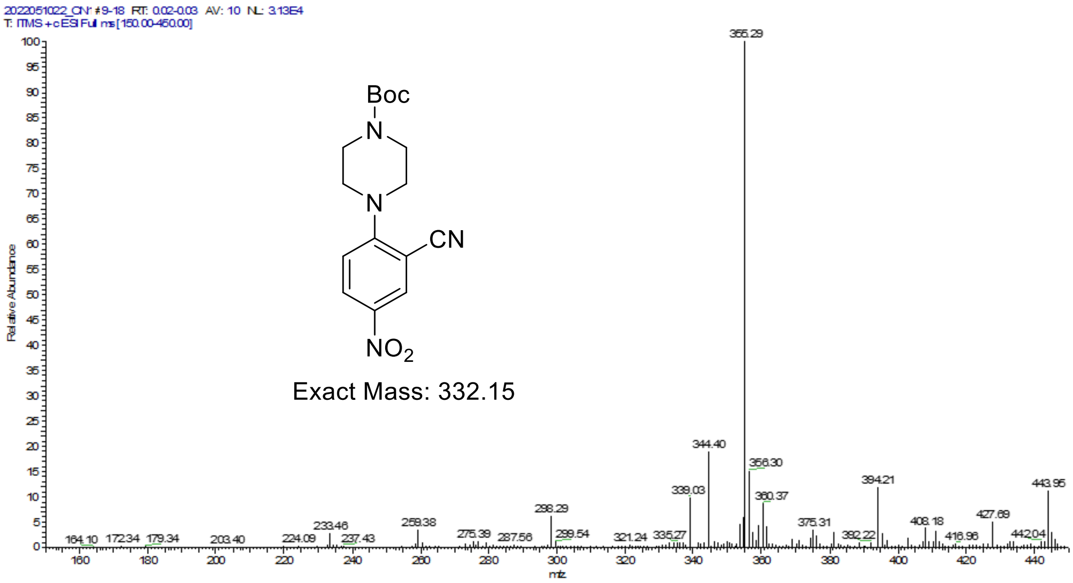

MS spectrum of **7b**

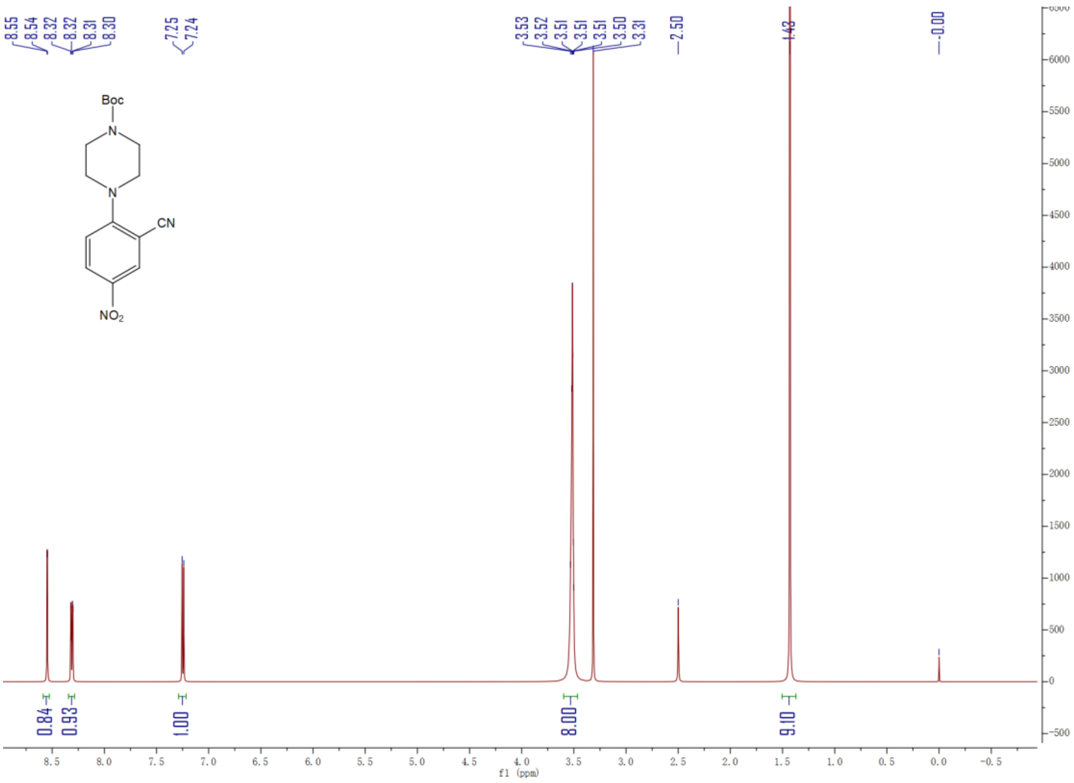

^{1}H NMR spectrum of **7b**

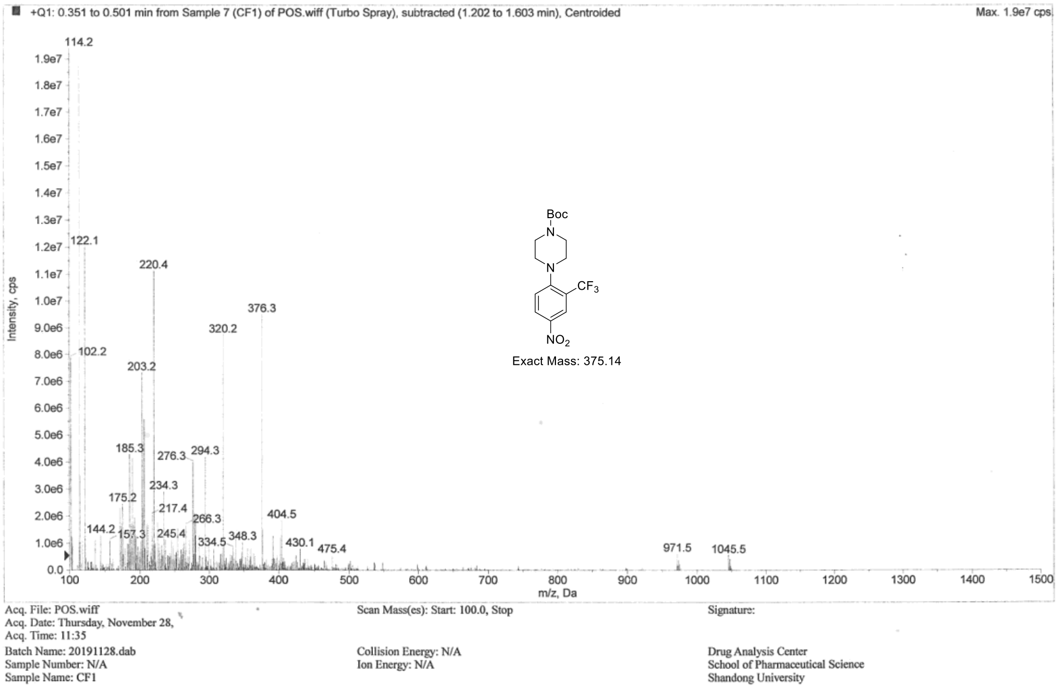

MS spectrum of **7c**

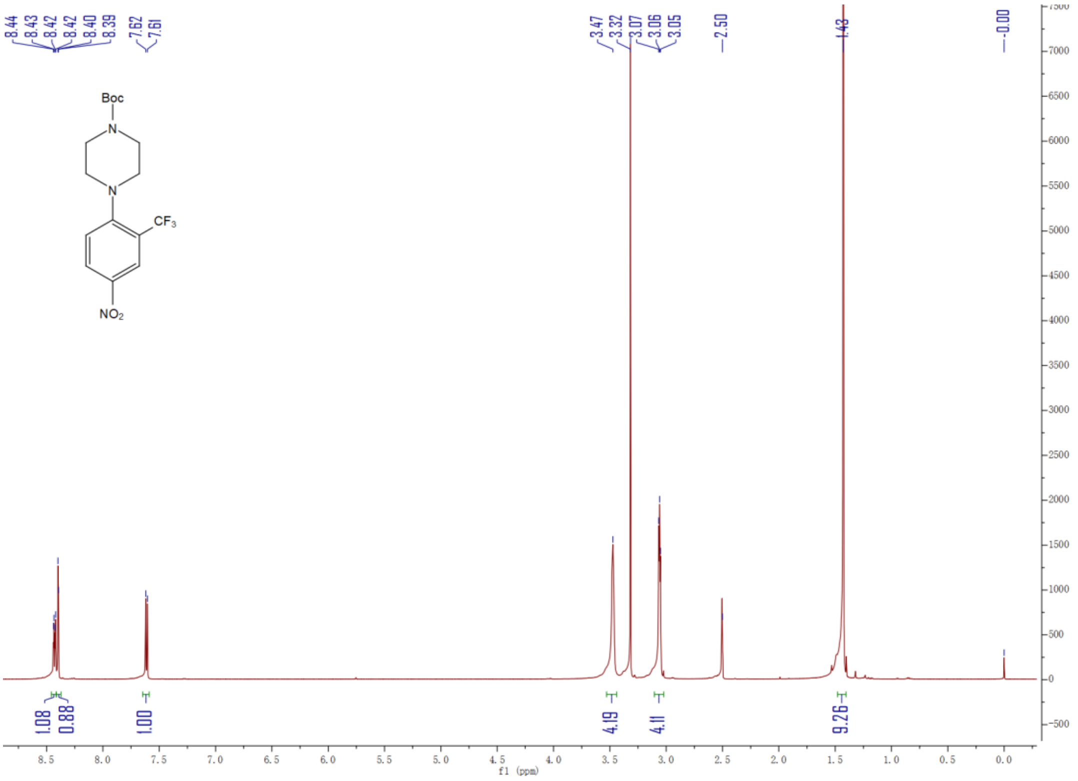

^{1}H NMR spectrum of **7c**

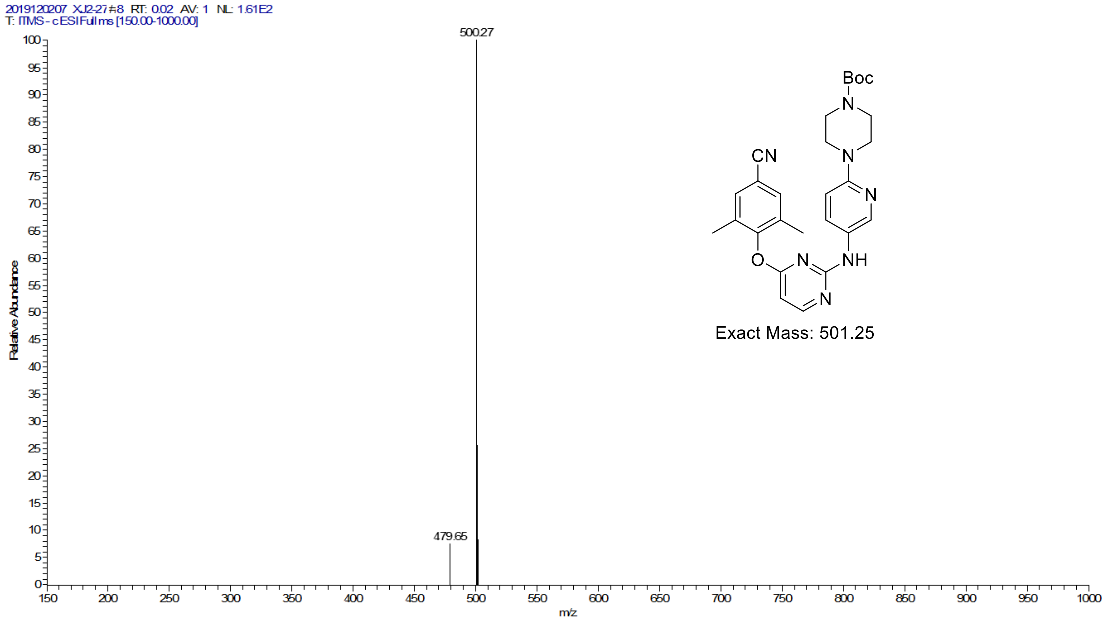

MS spectrum of **13a**

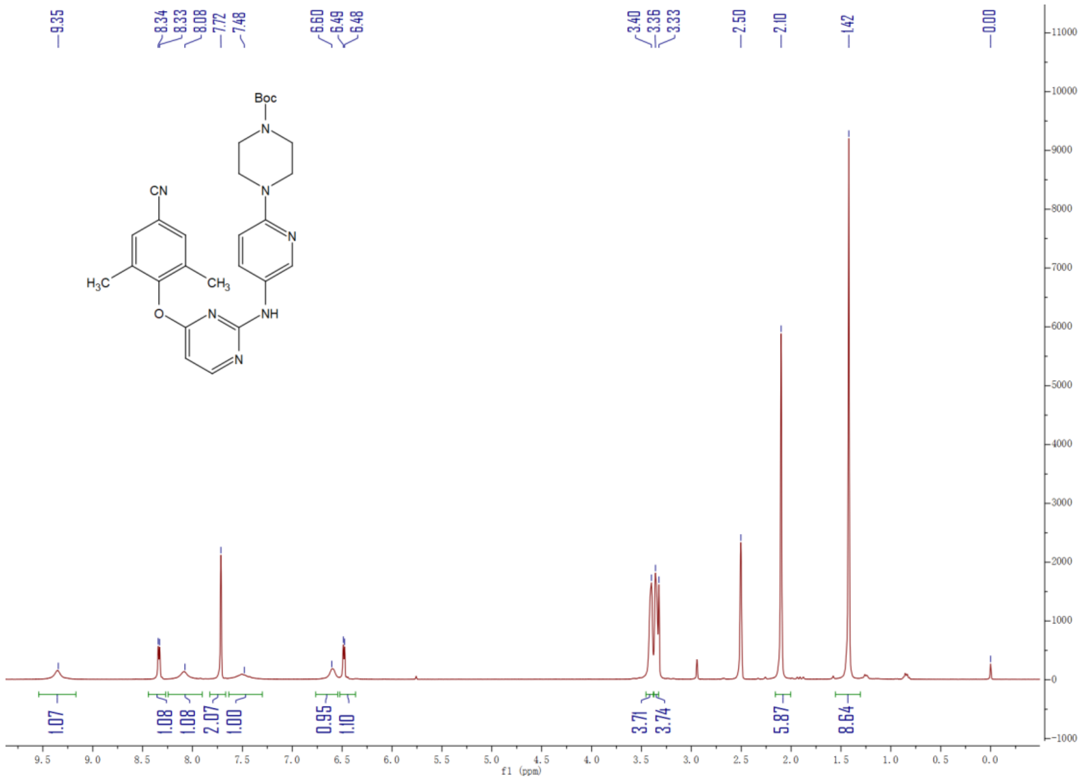

^{1}H NMR spectrum of **13a**

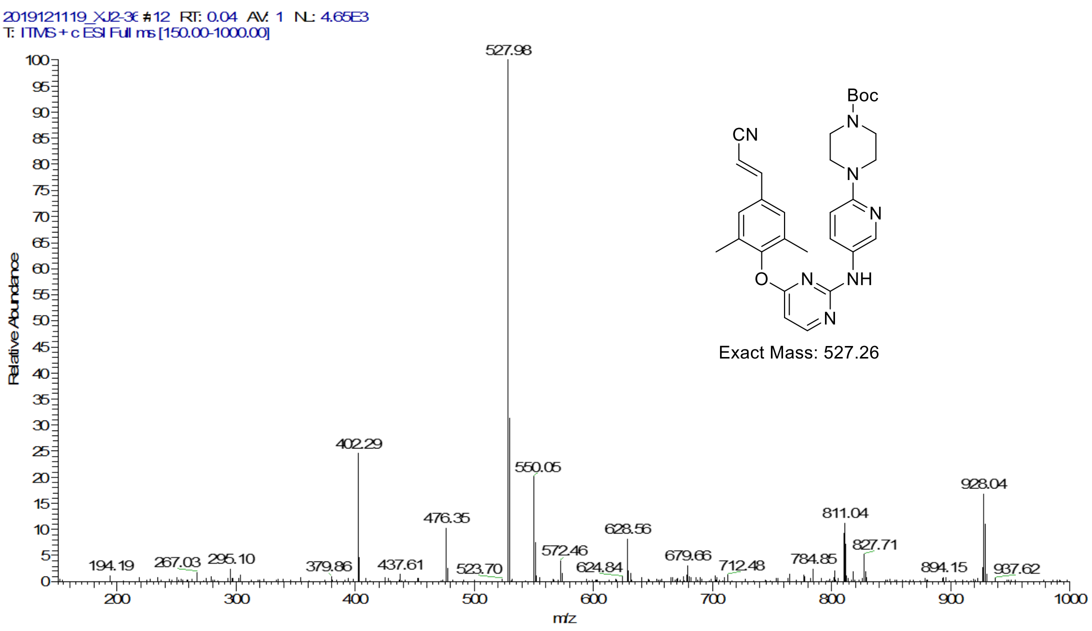

MS spectrum of **14a**

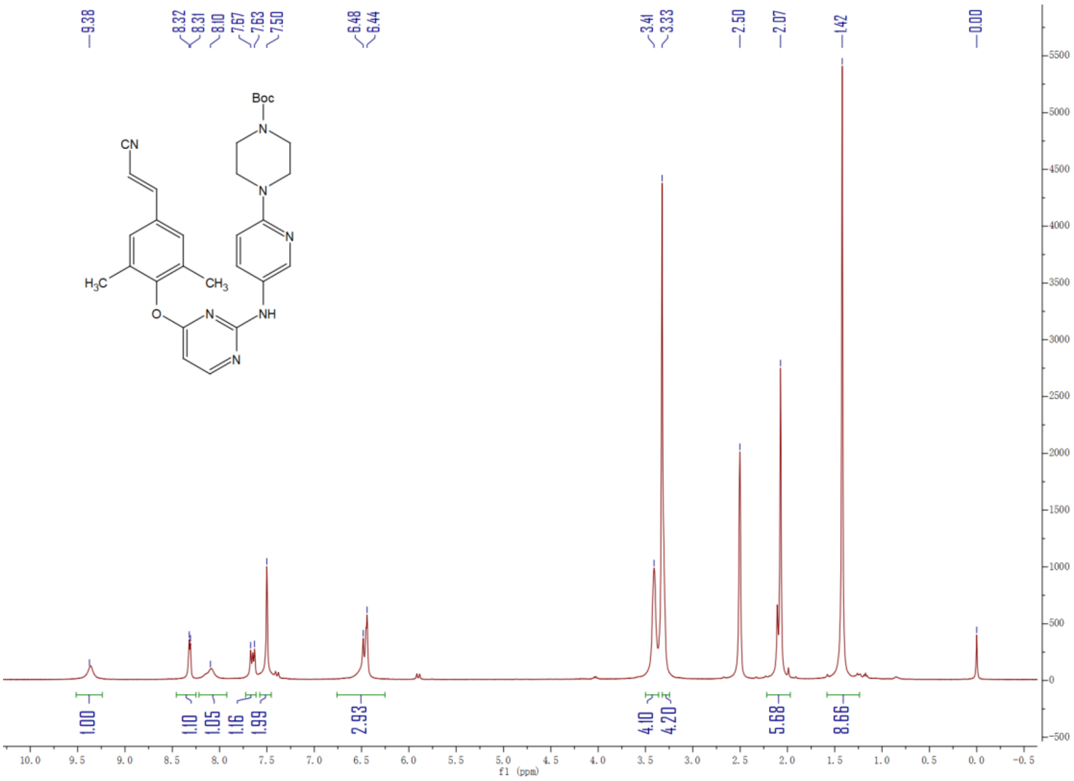

^{1}H NMR spectrum of **14a**

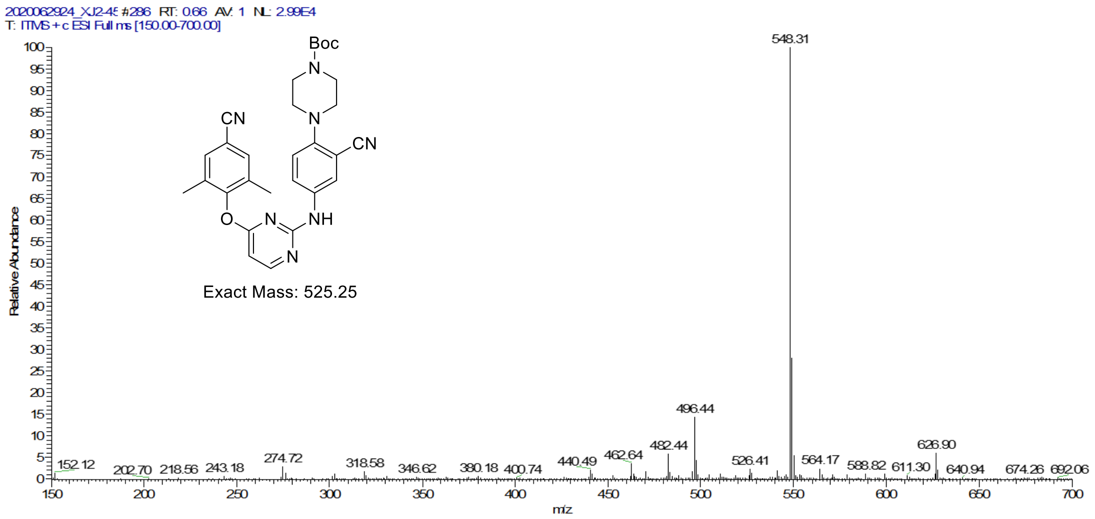

MS spectrum of **13b**

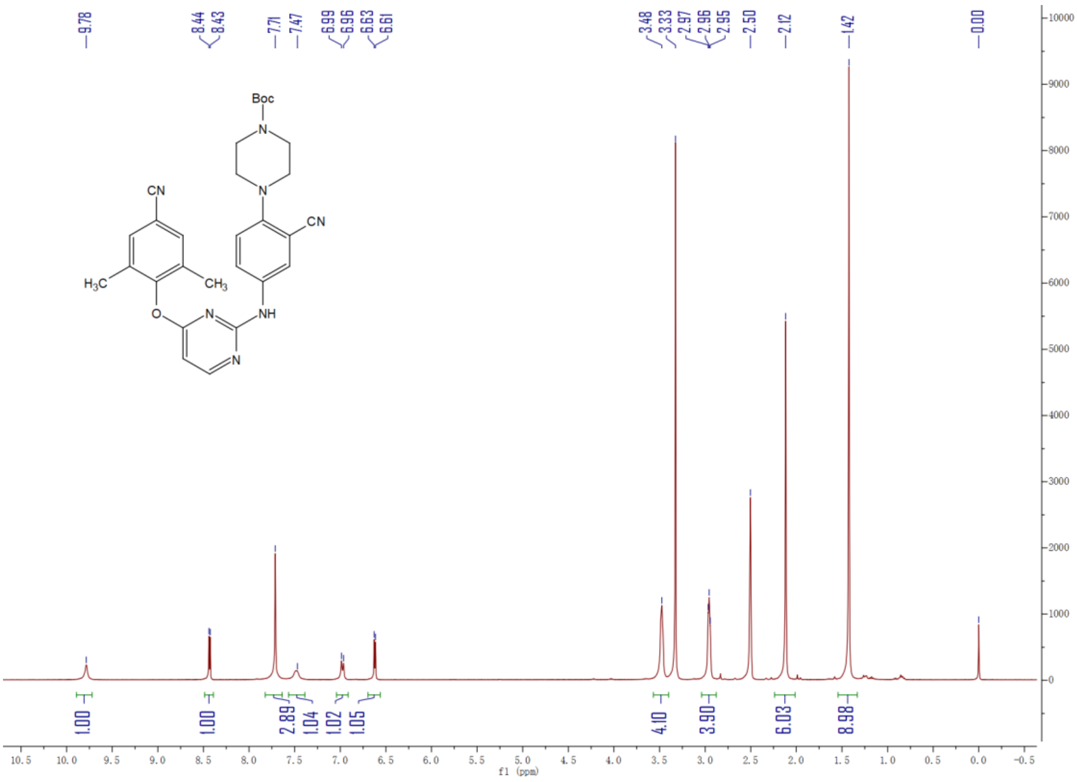

^{1}H NMR spectrum of **13b**

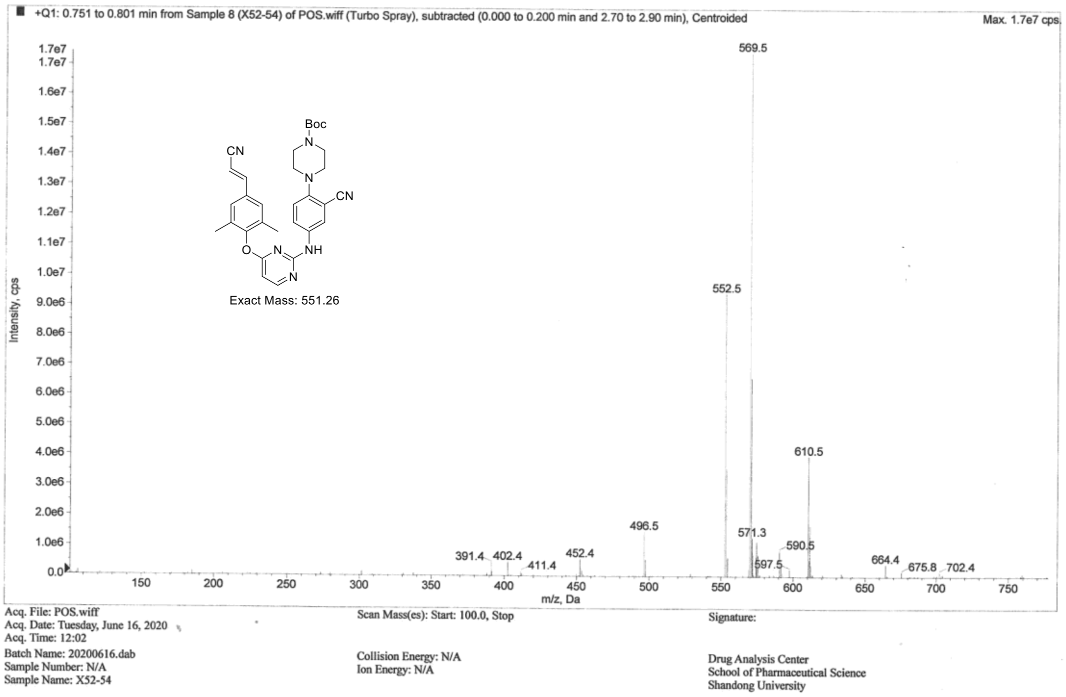

MS spectrum of **14b**

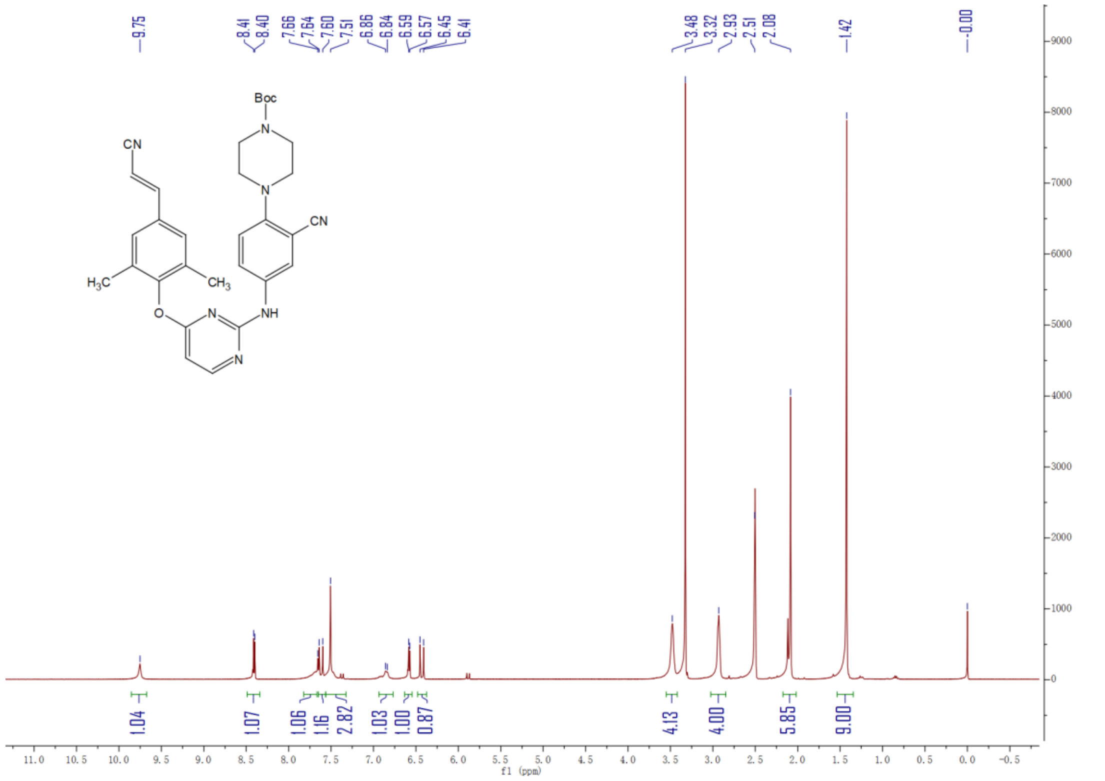

^{1}H NMR spectrum of **14b**

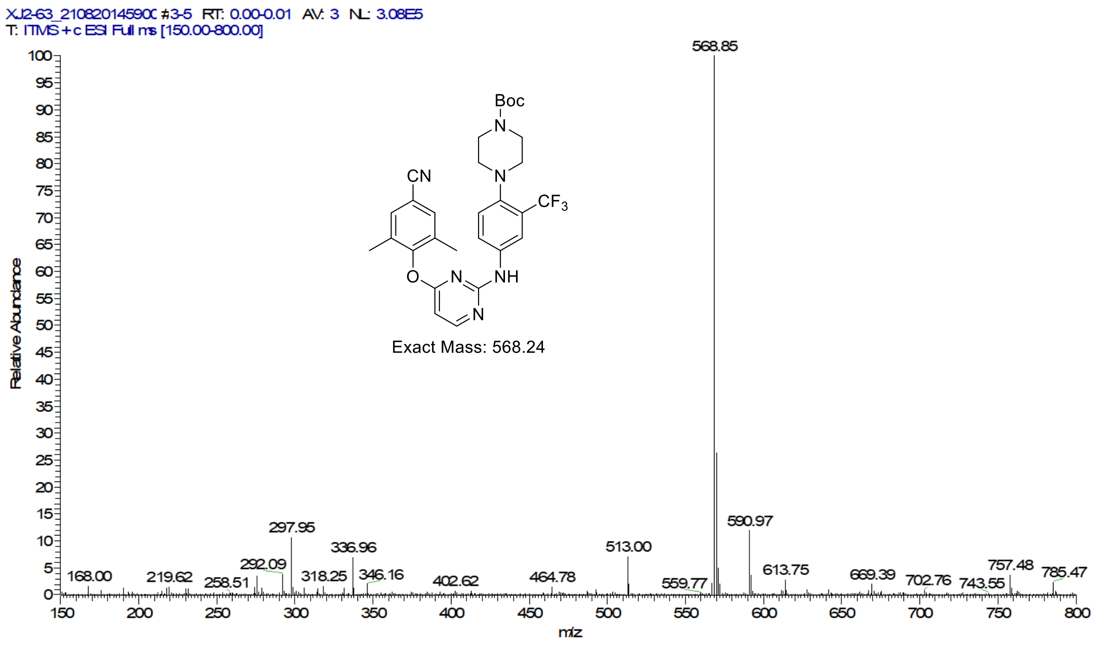

MS spectrum of **13c**

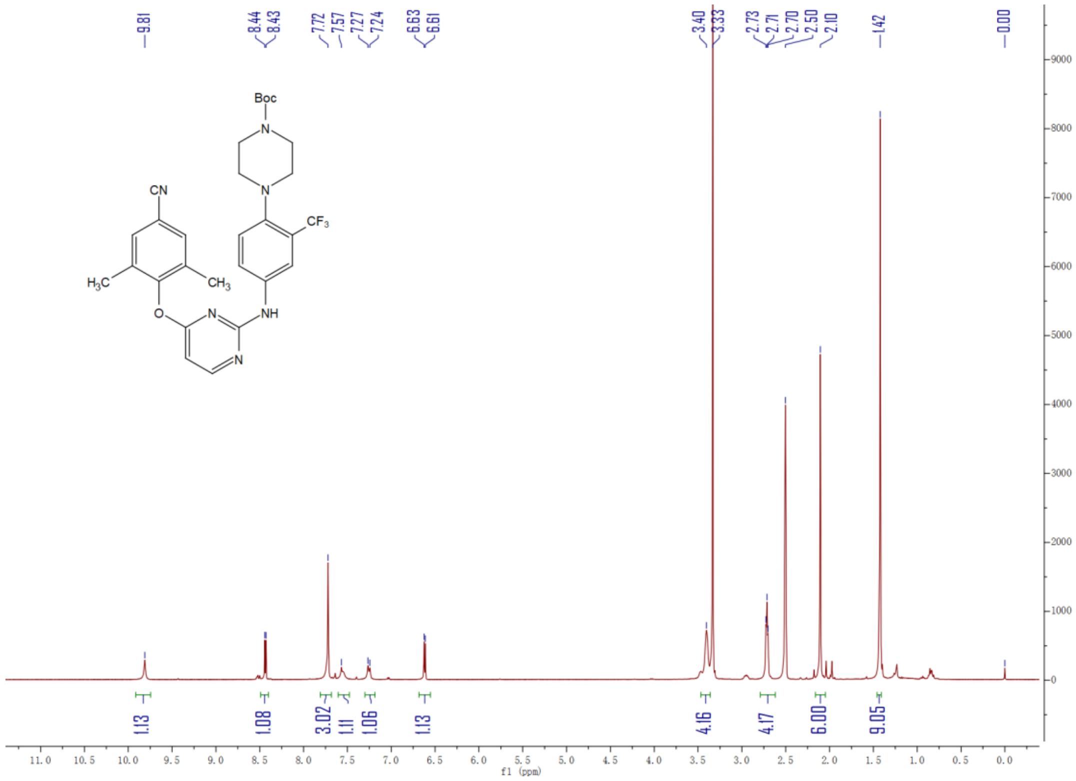

^{1}H NMR spectrum of **13c**

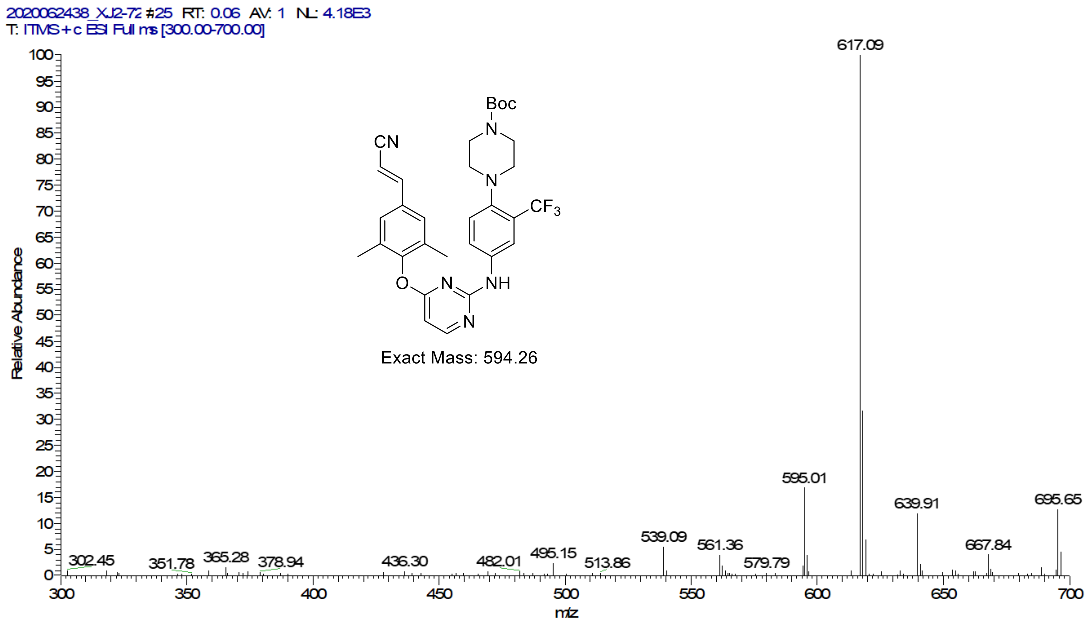

MS spectrum of **14c**

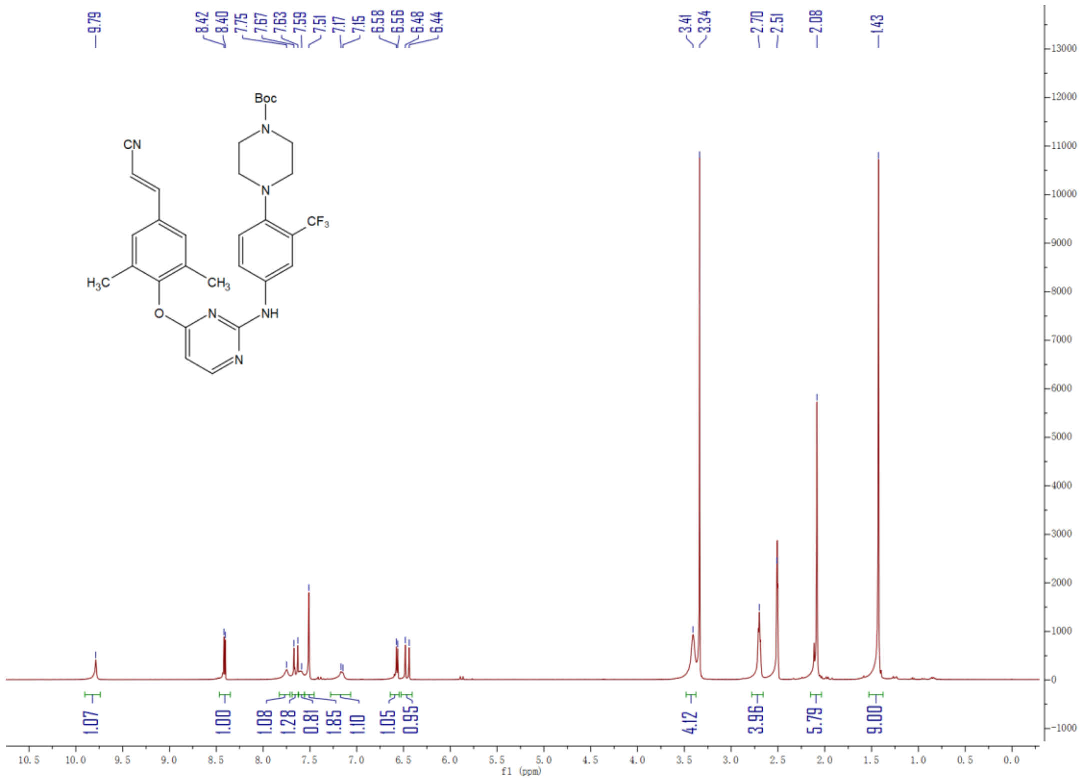

^{1}H NMR spectrum of **14c**

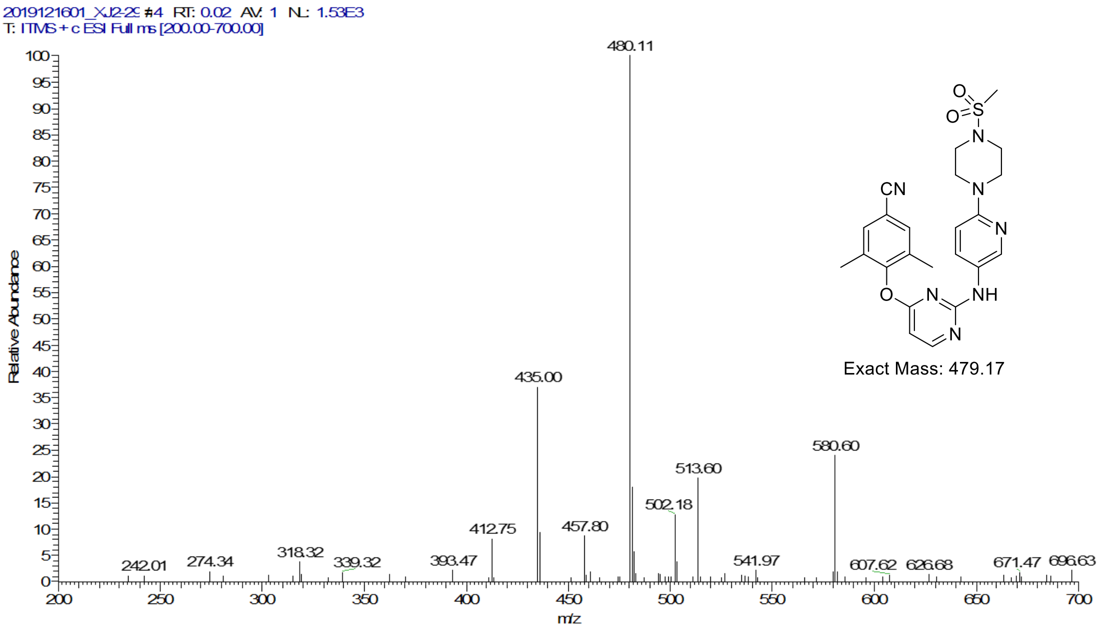

MS spectrum of **17a1**

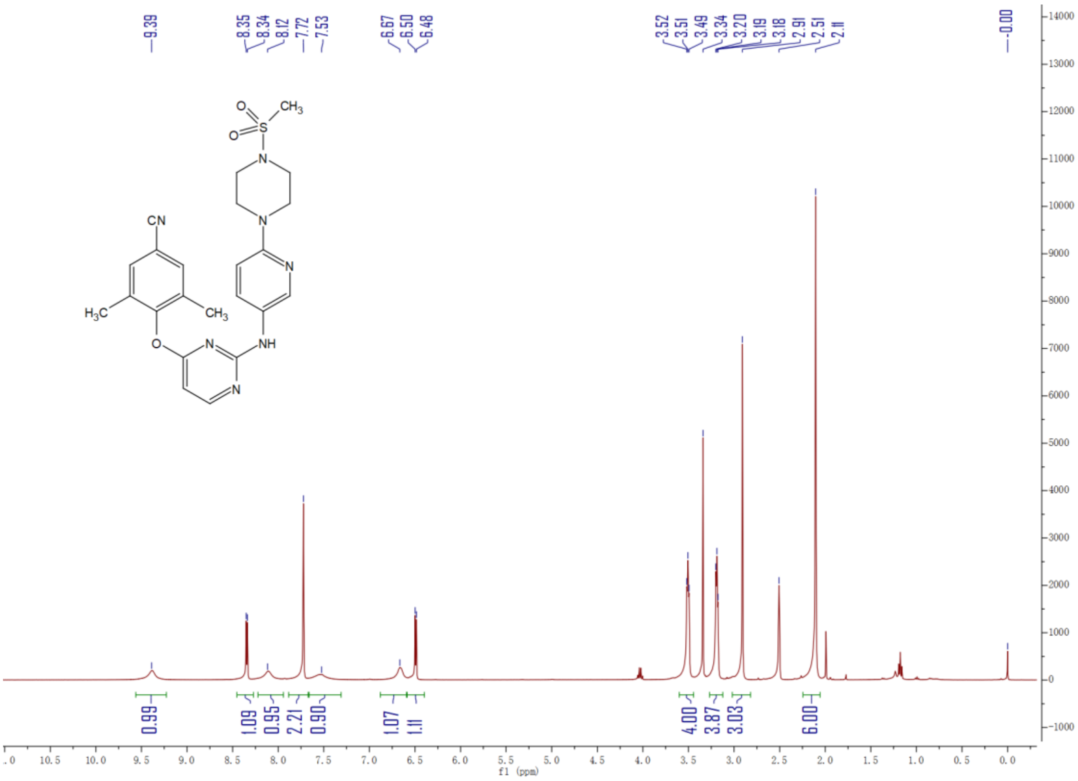

^{1}H NMR spectrum of **17a1**

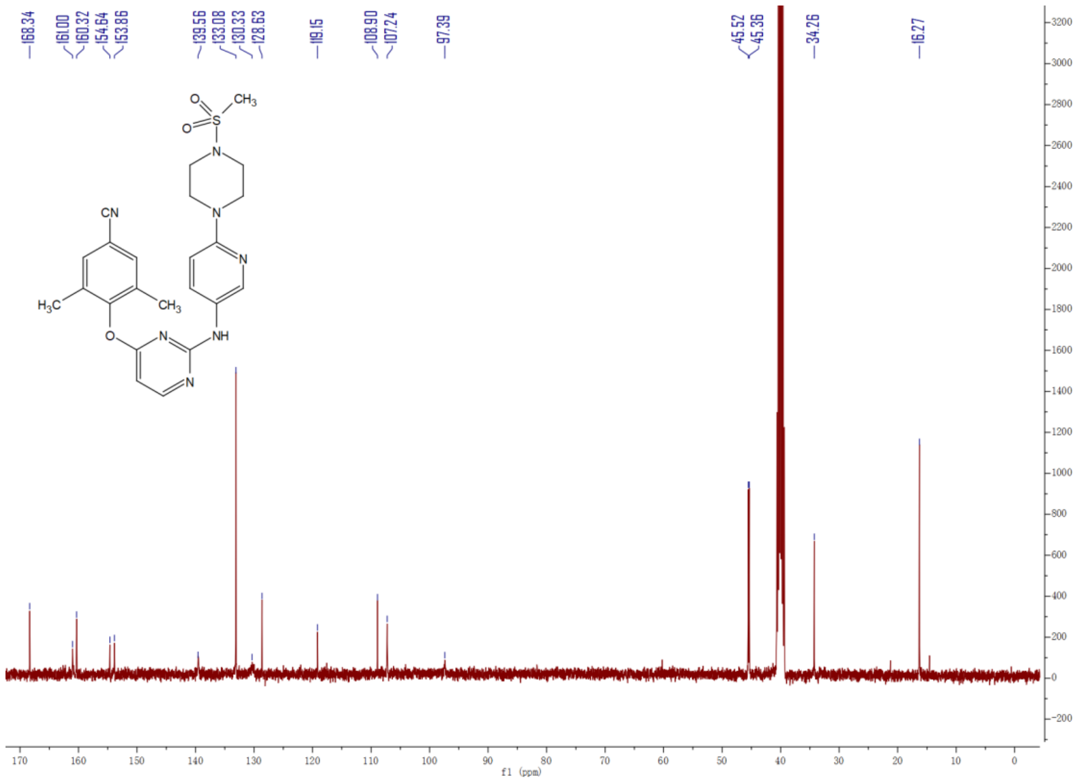

^{13}C NMR spectrum of **17a1**

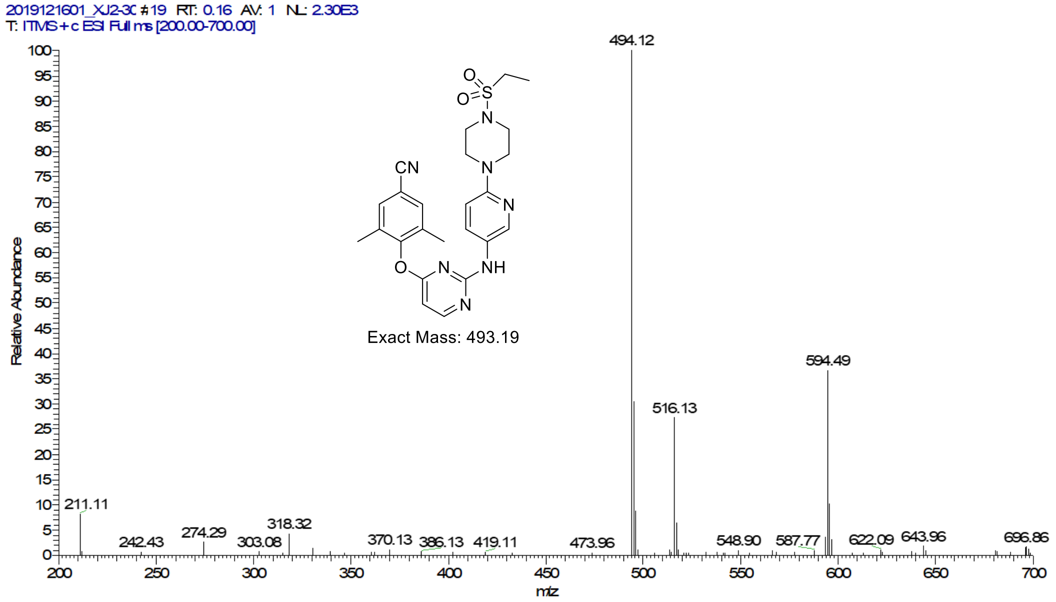

MS spectrum of **17a2**

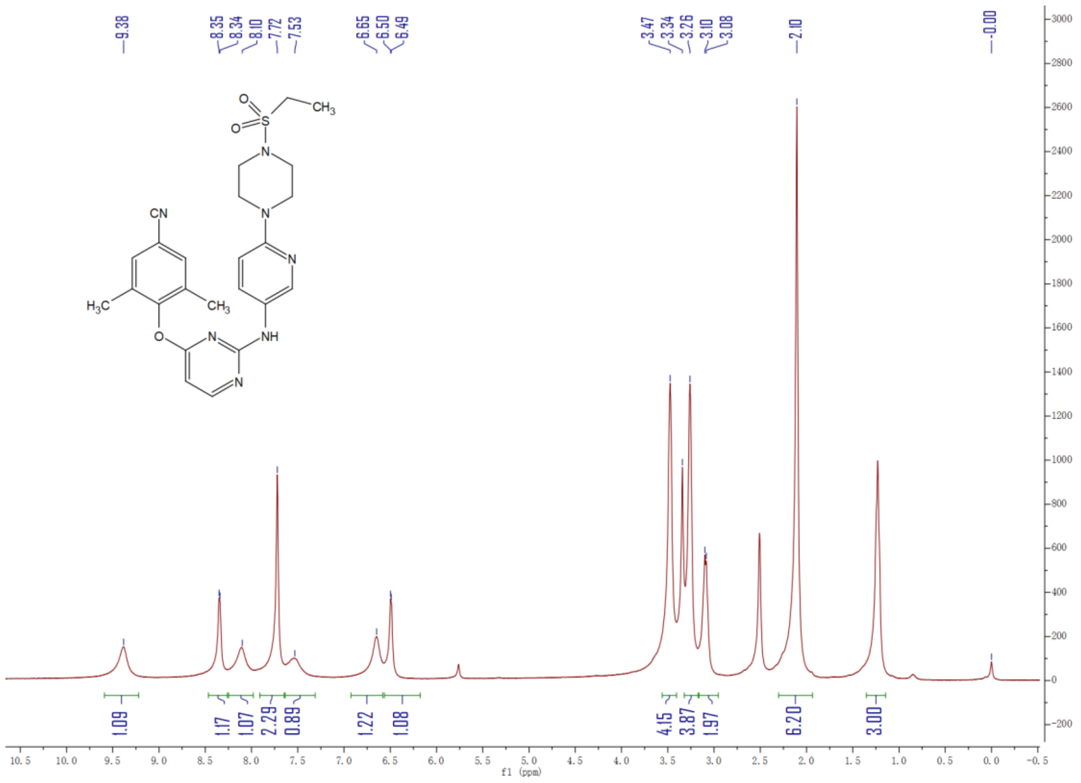

^{1}H NMR spectrum of **17a2**

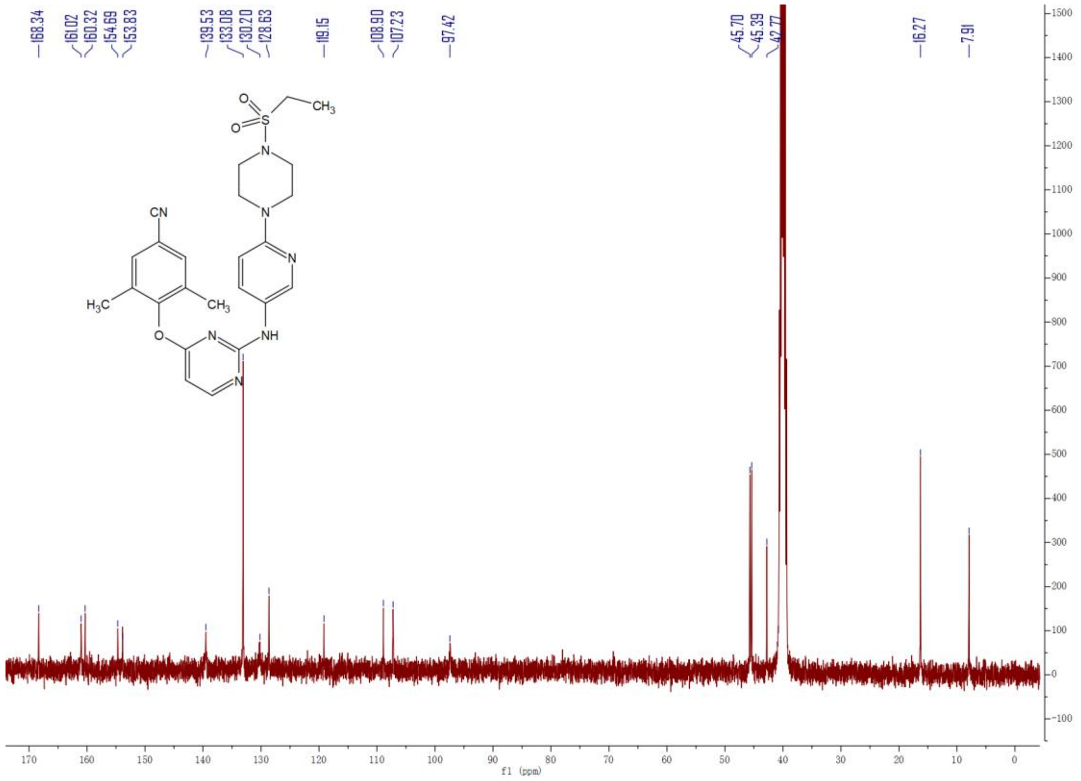

^{13}C NMR spectrum of **17a2**

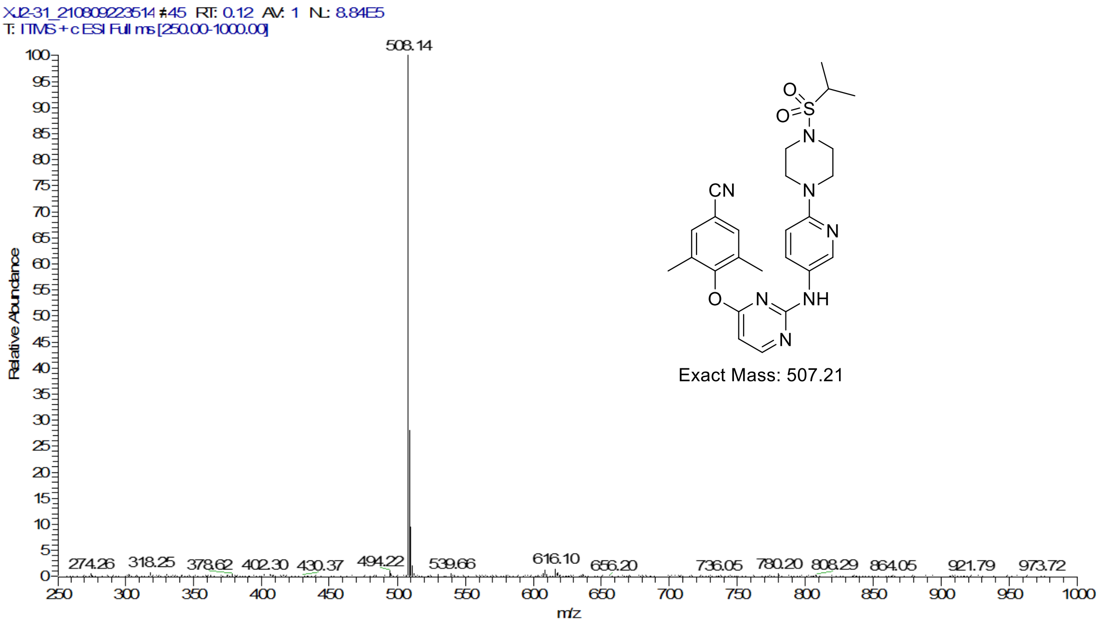

MS spectrum of **17a3**

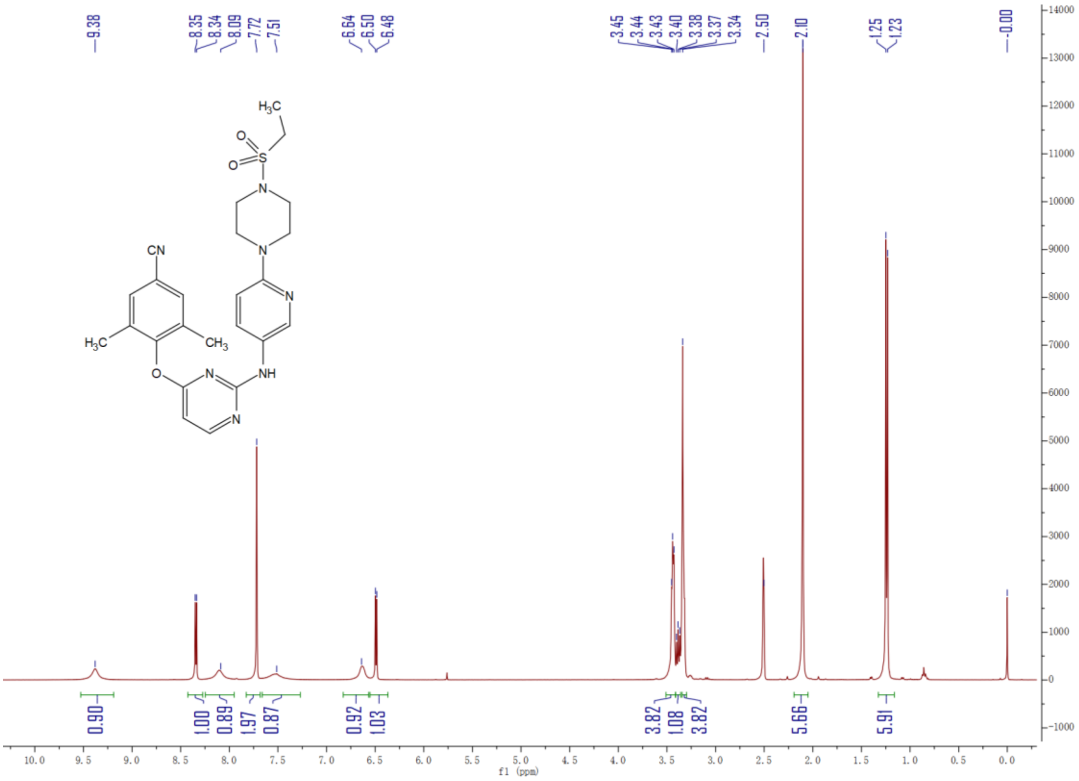

^{1}H NMR spectrum of **17a3**

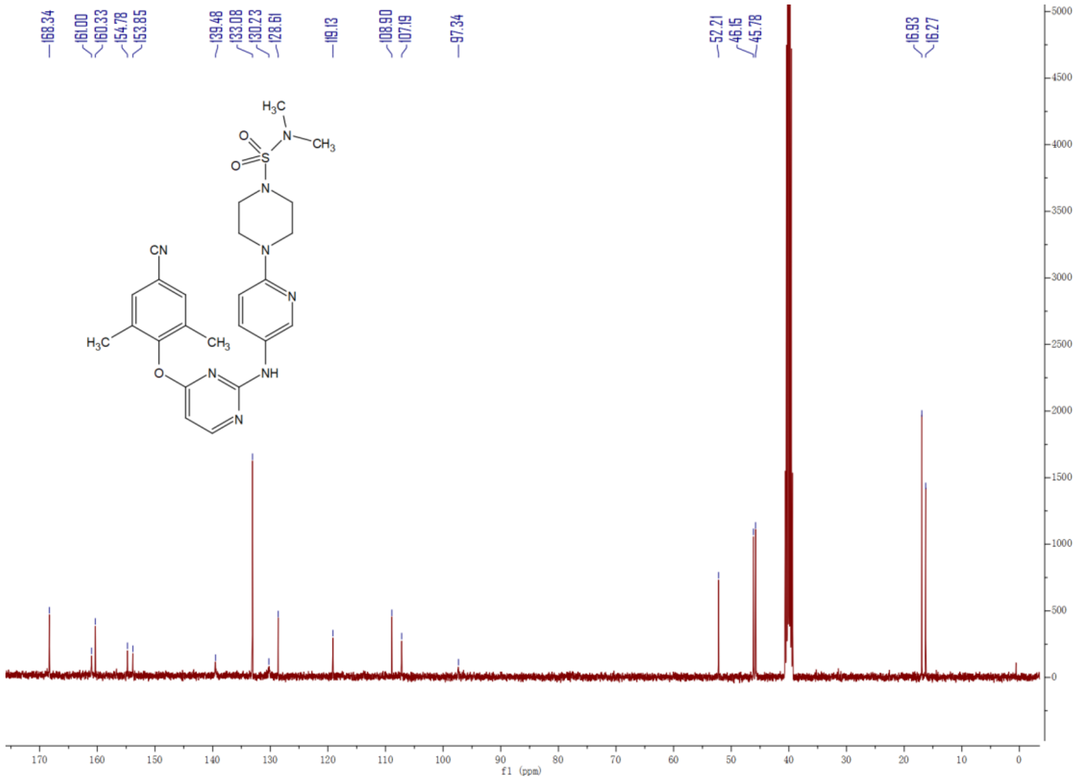

^{13}C NMR spectrum of **17a3**

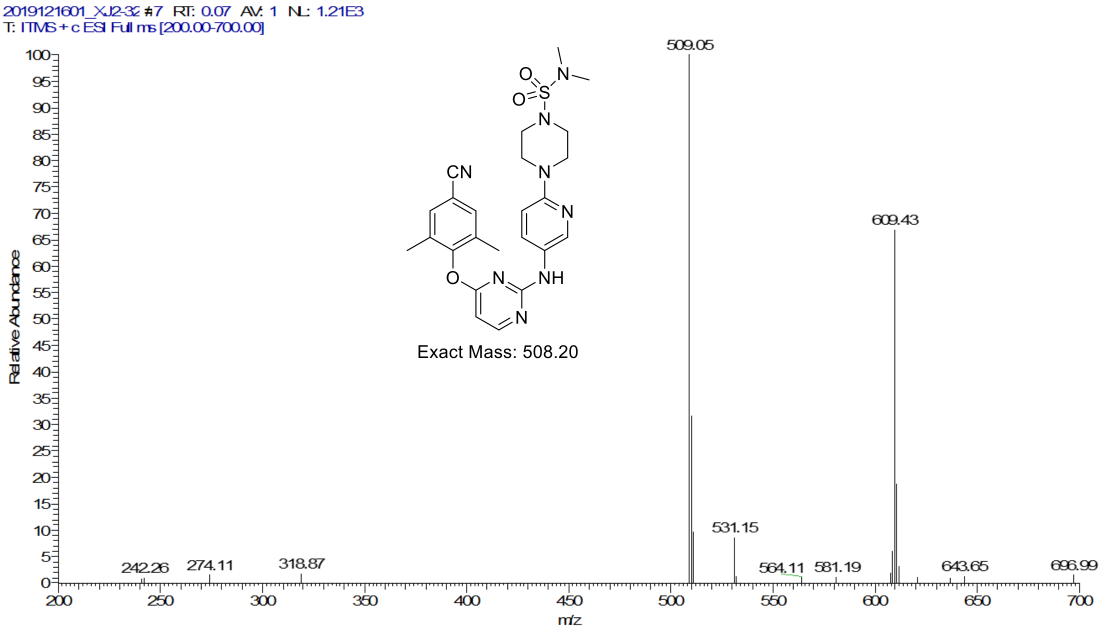

MS spectrum of **17a4**

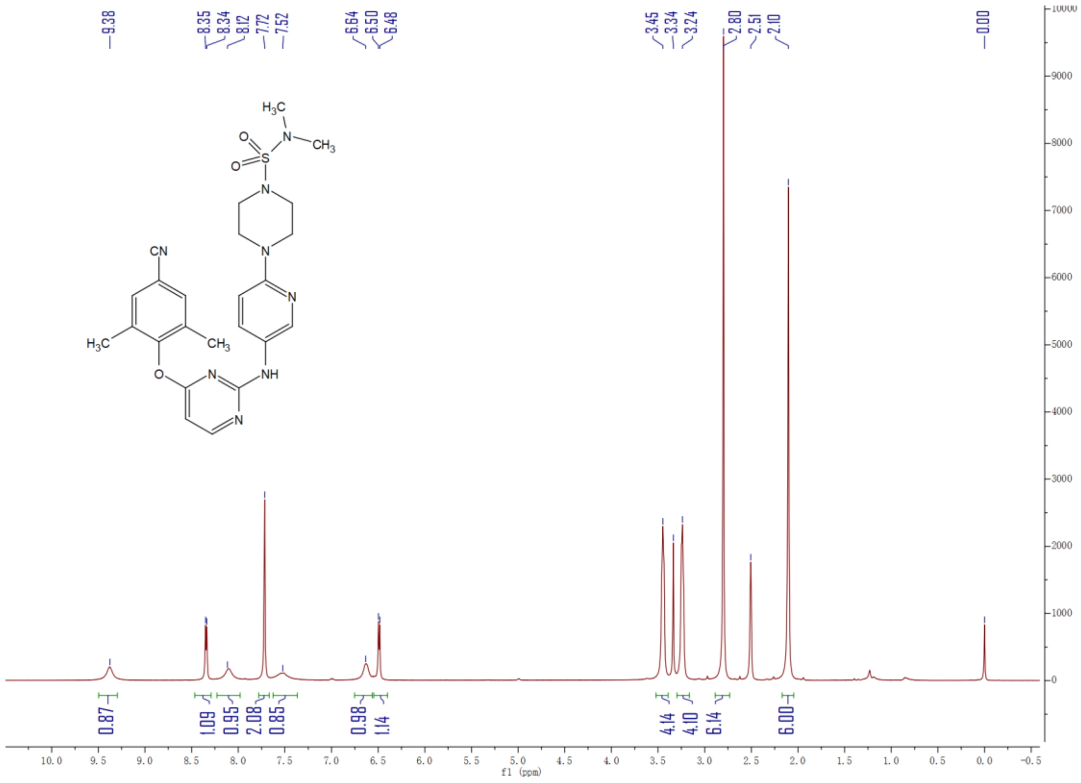

^{1}H NMR spectrum of **17a4**

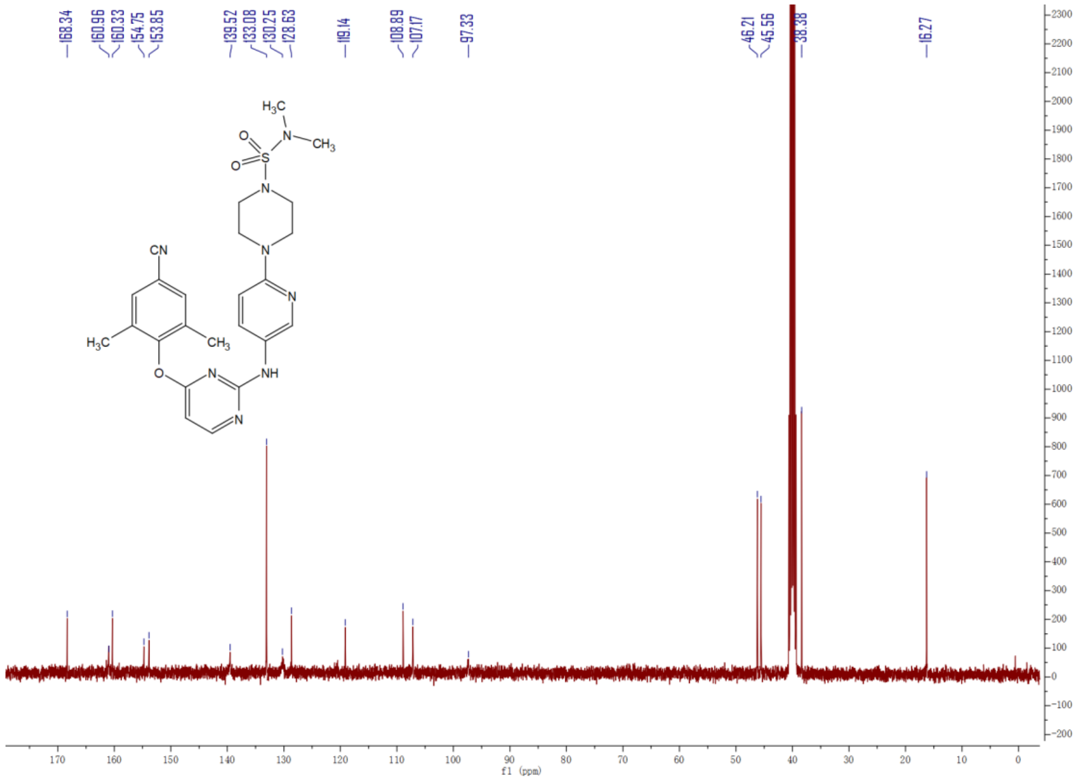

^{13}C NMR spectrum of **17a4**

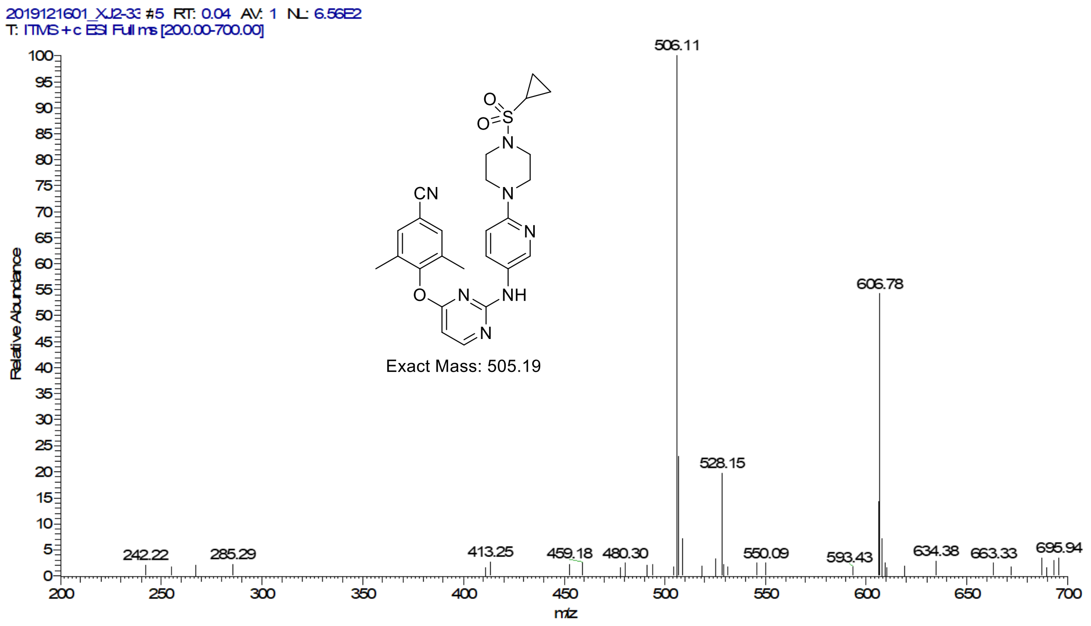

MS spectrum of **17a5**

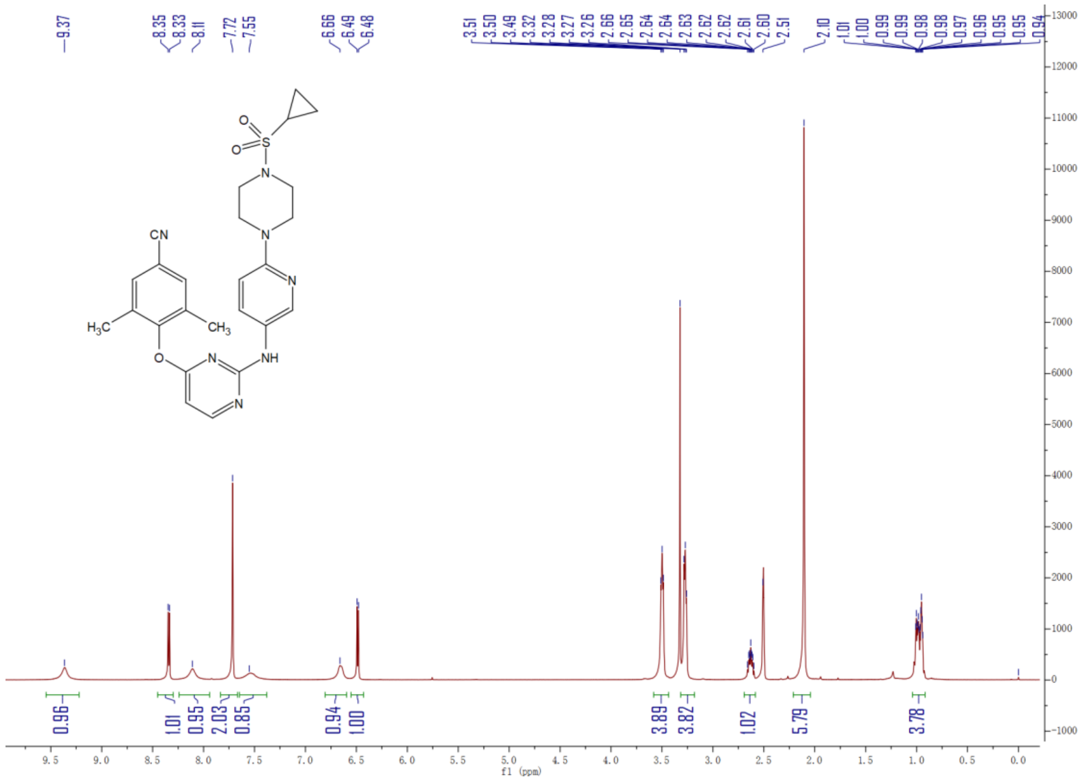

^{1}H NMR spectrum of **17a5**

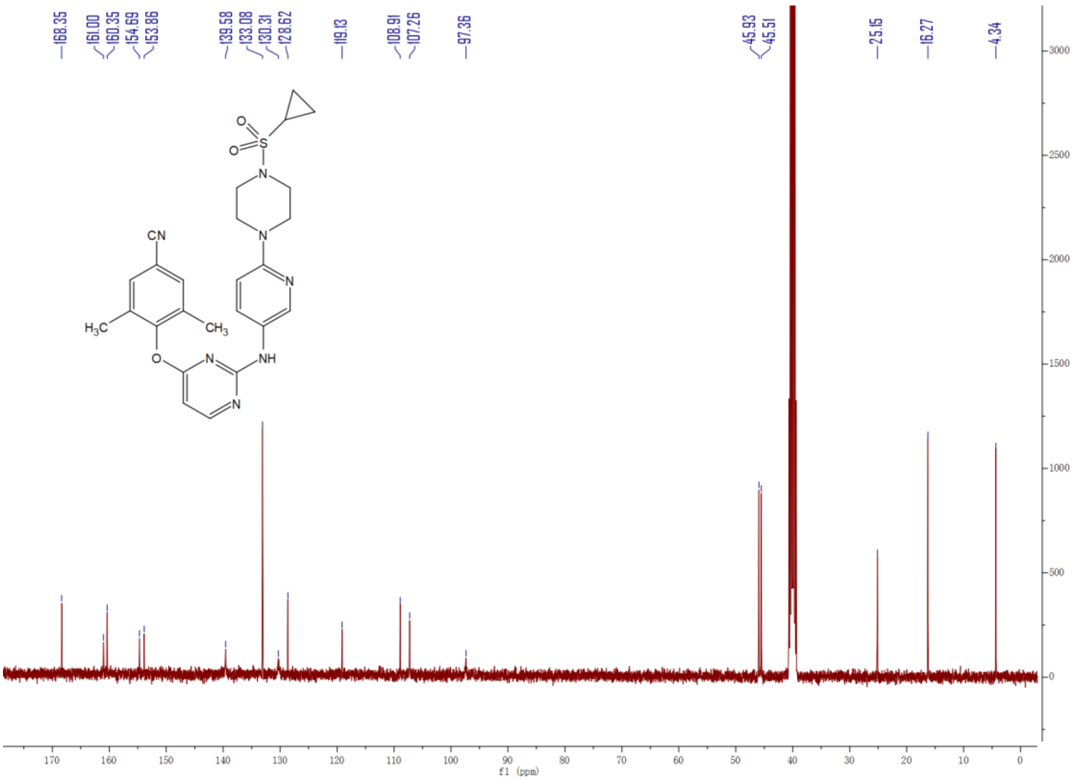

^{13}C NMR spectrum of **17a5**

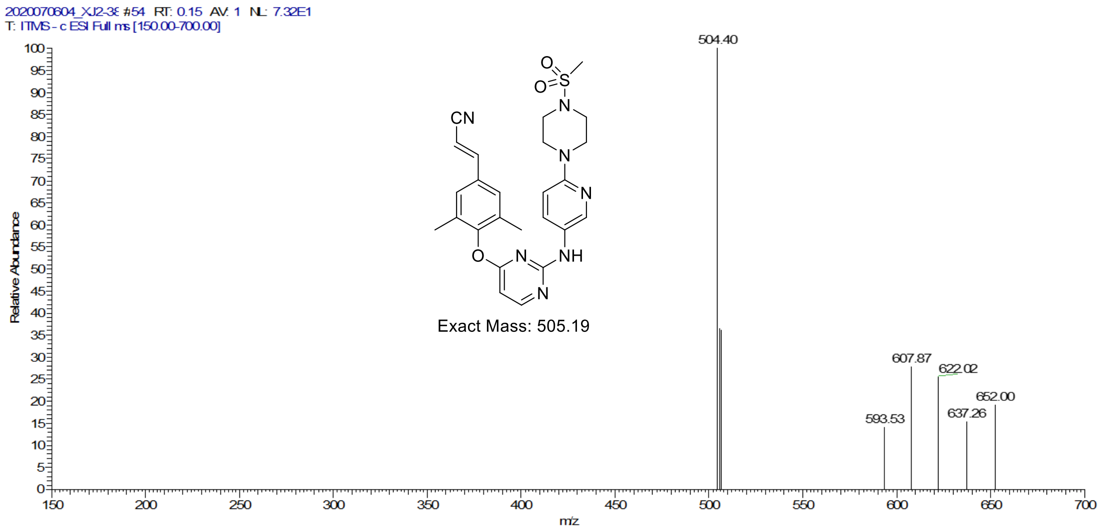

MS spectrum of **18a1**

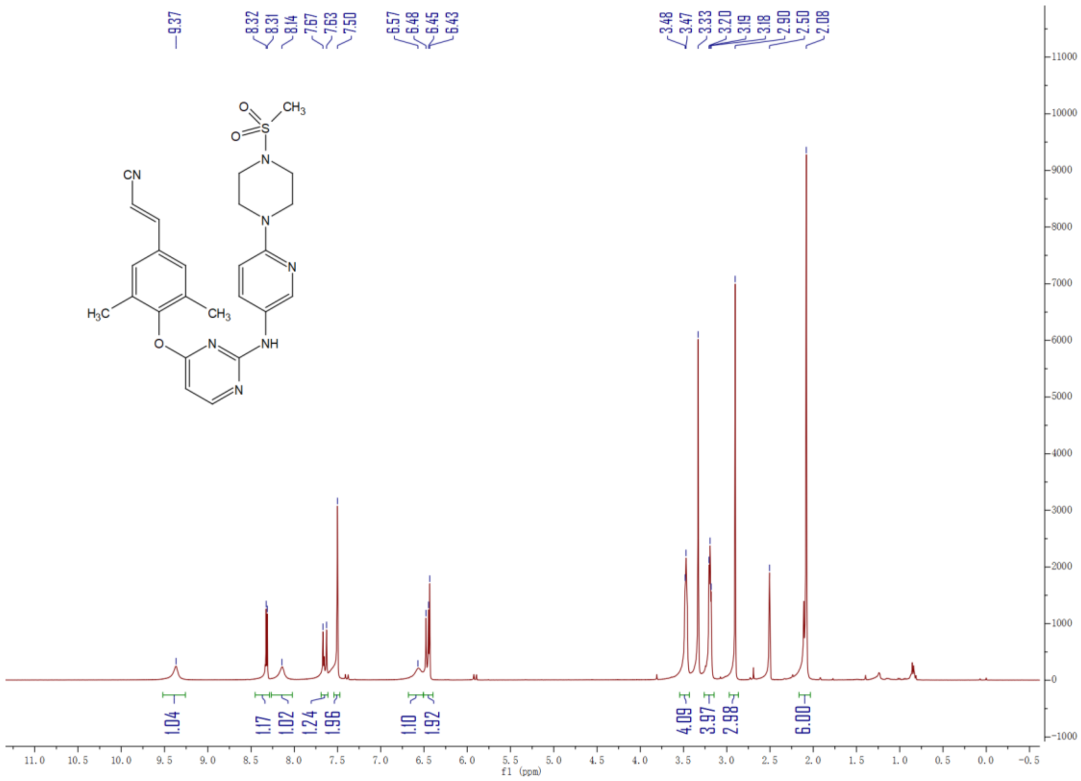

^{1}H NMR spectrum of **18a1**

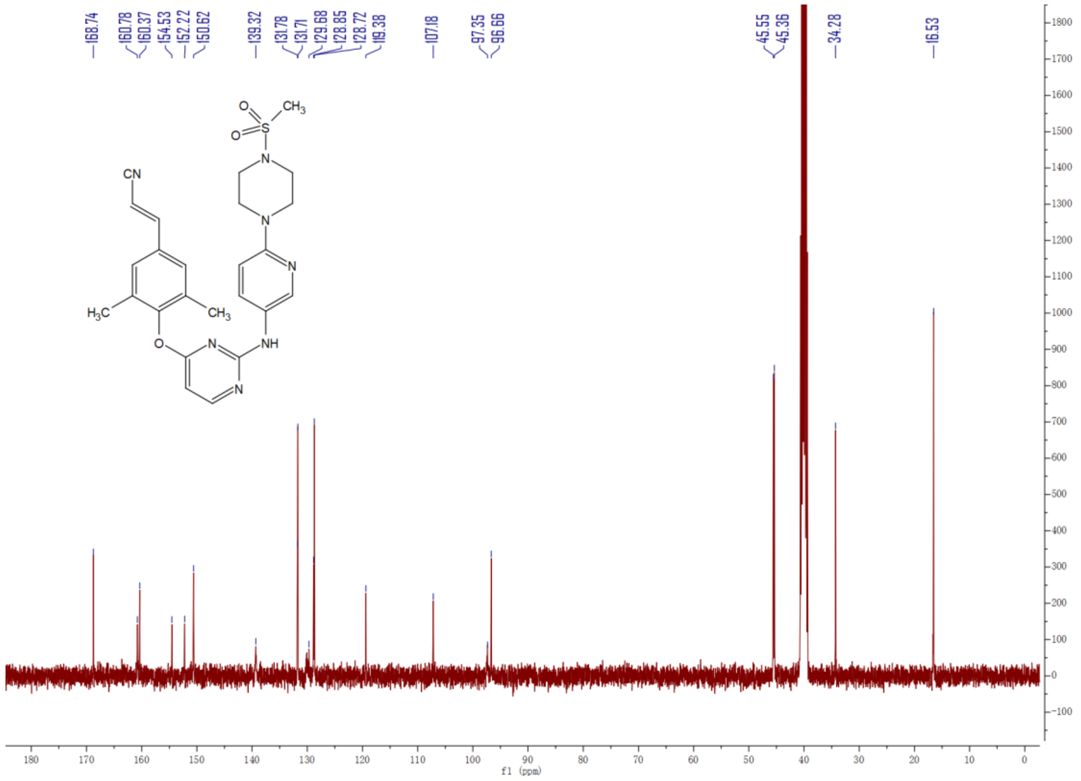

^{13}C NMR spectrum of **18a1**

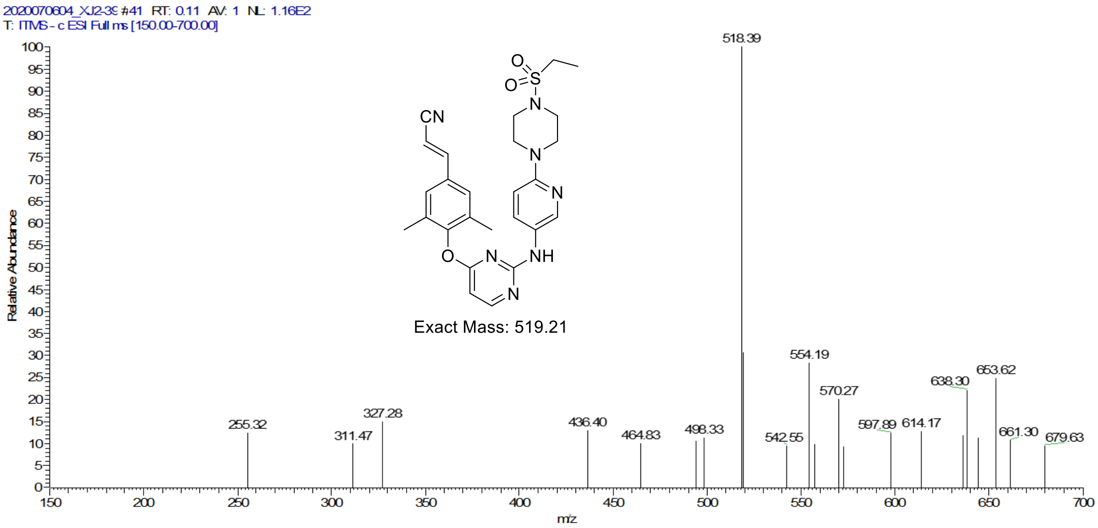

MS spectrum of **18a2**

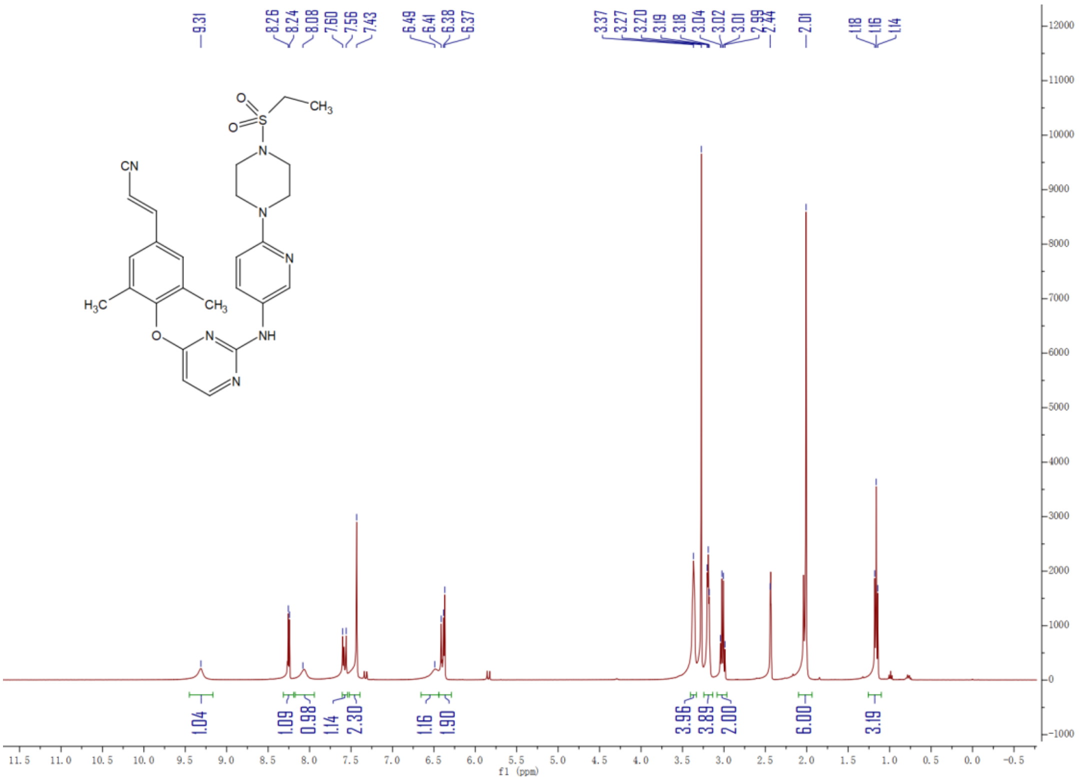

^{1}H NMR spectrum of **18a2**

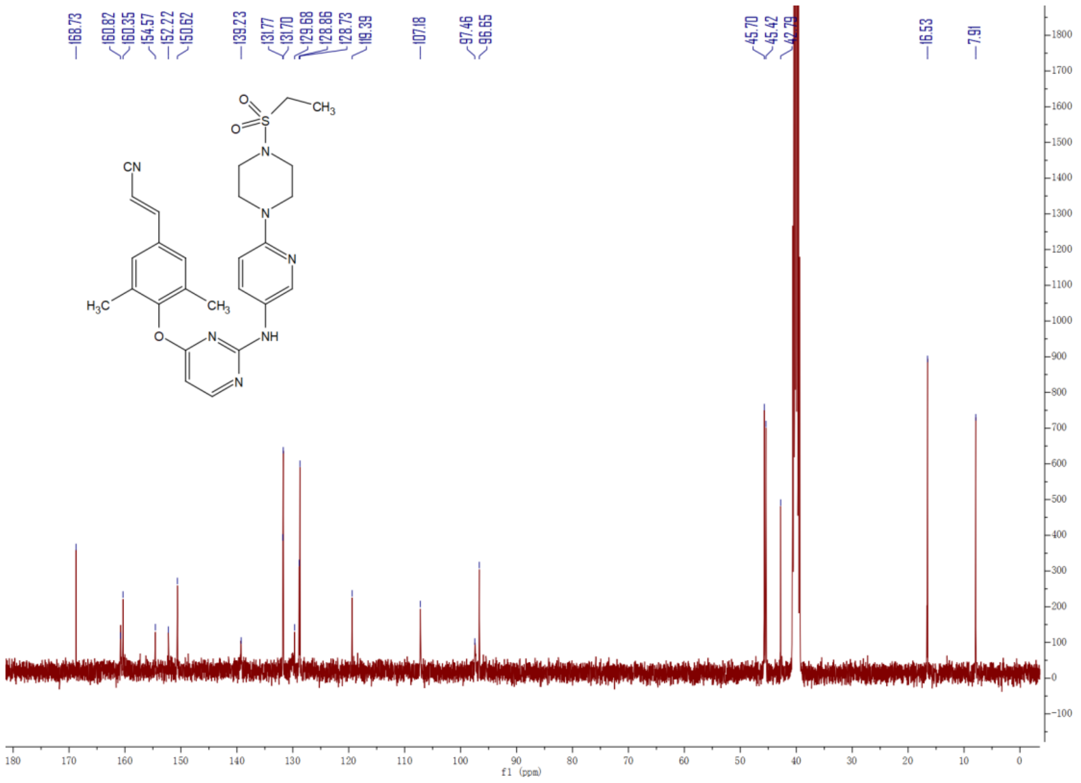

^{13}C NMR spectrum of **18a2**

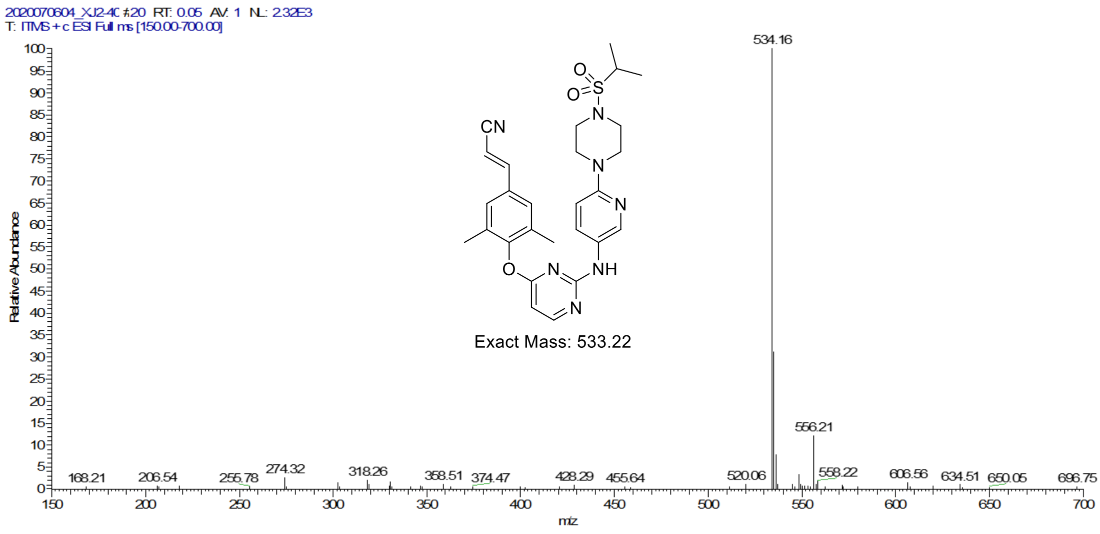

MS spectrum of **18a3**

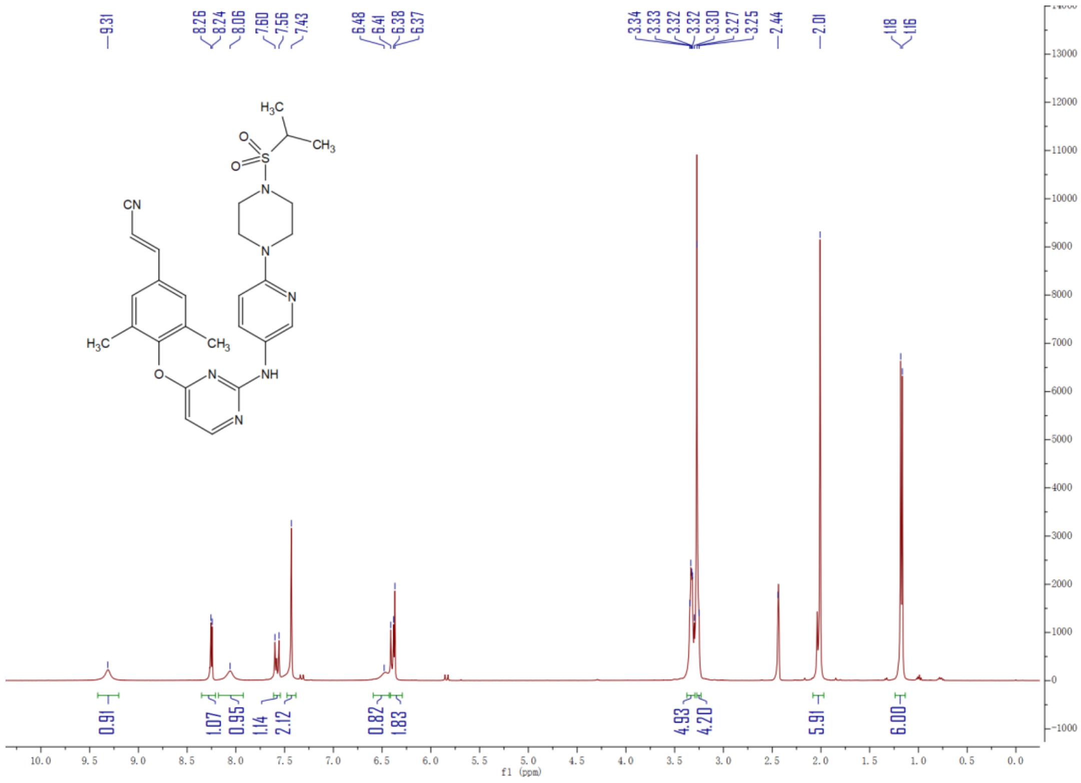

^{1}H NMR spectrum of **18a3**

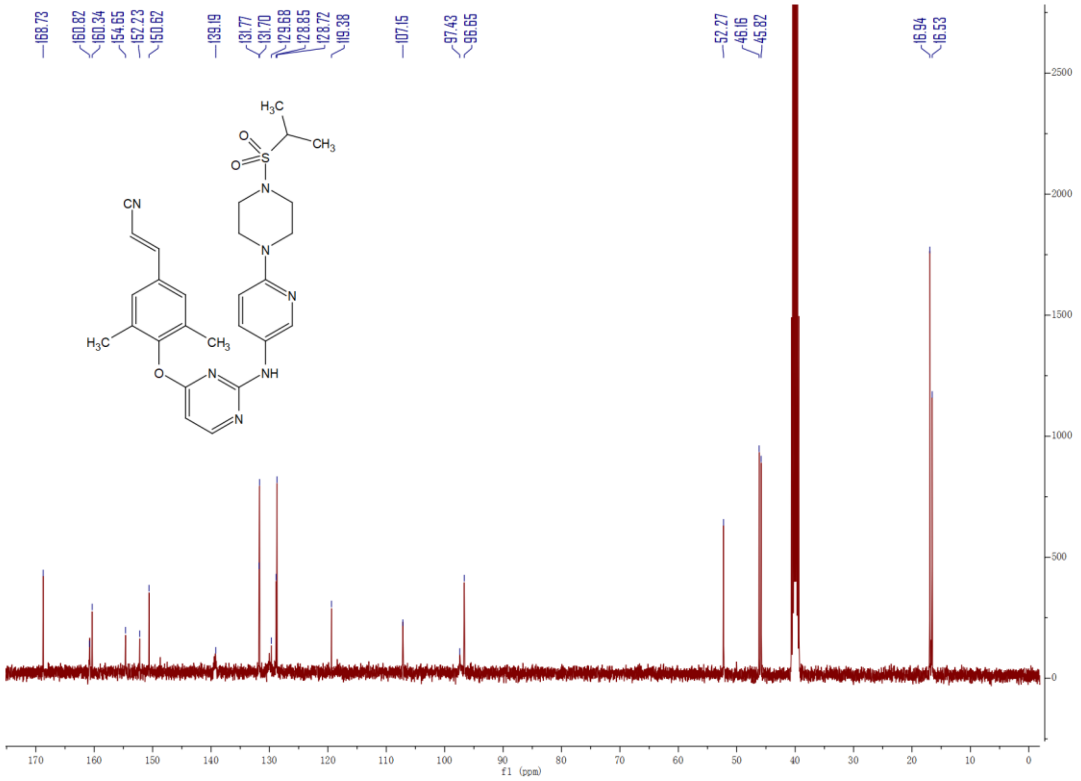

^{13}C NMR spectrum of **18a3**

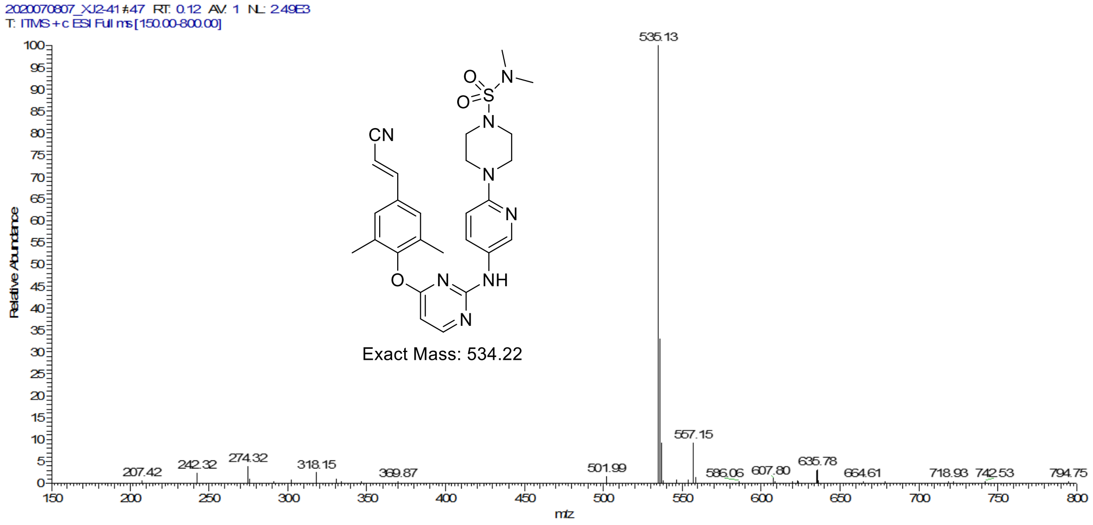

MS spectrum of **18a4**

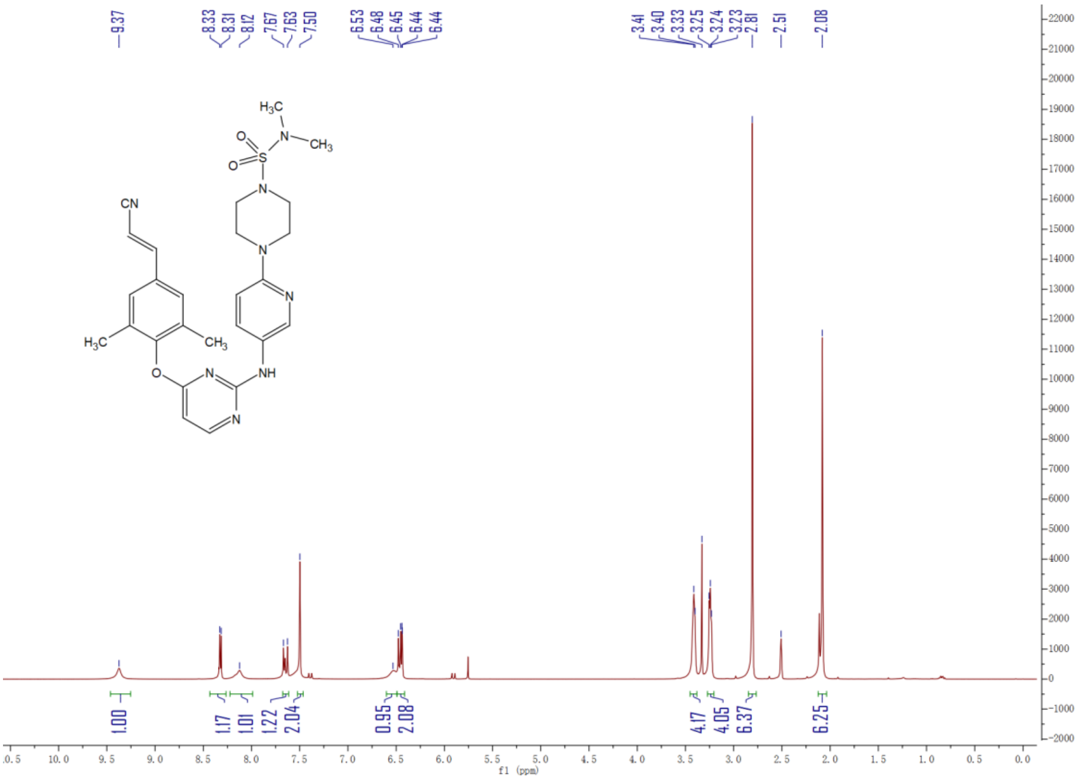

^{1}H NMR spectrum of **18a4**

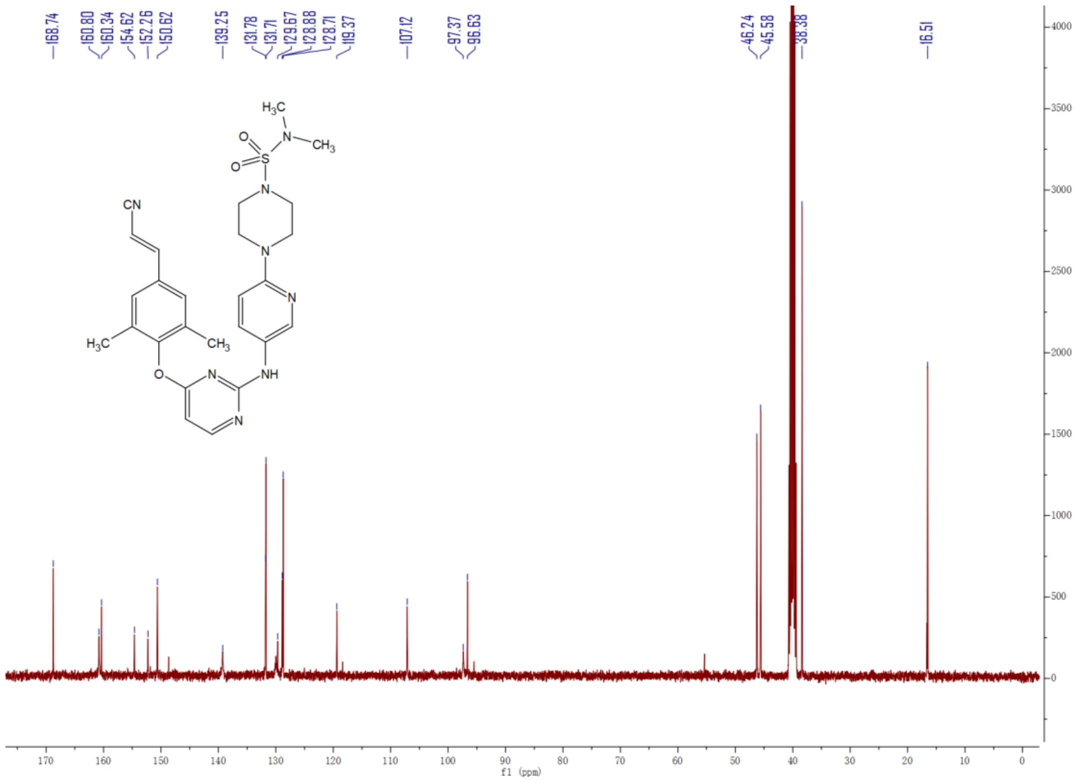

^{13}C NMR spectrum of **18a4**

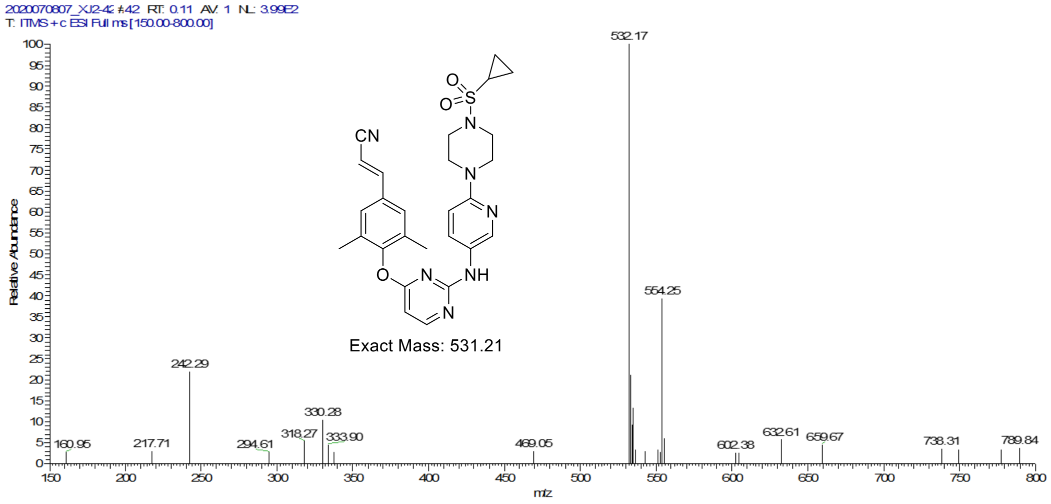

MS spectrum of **18a5**

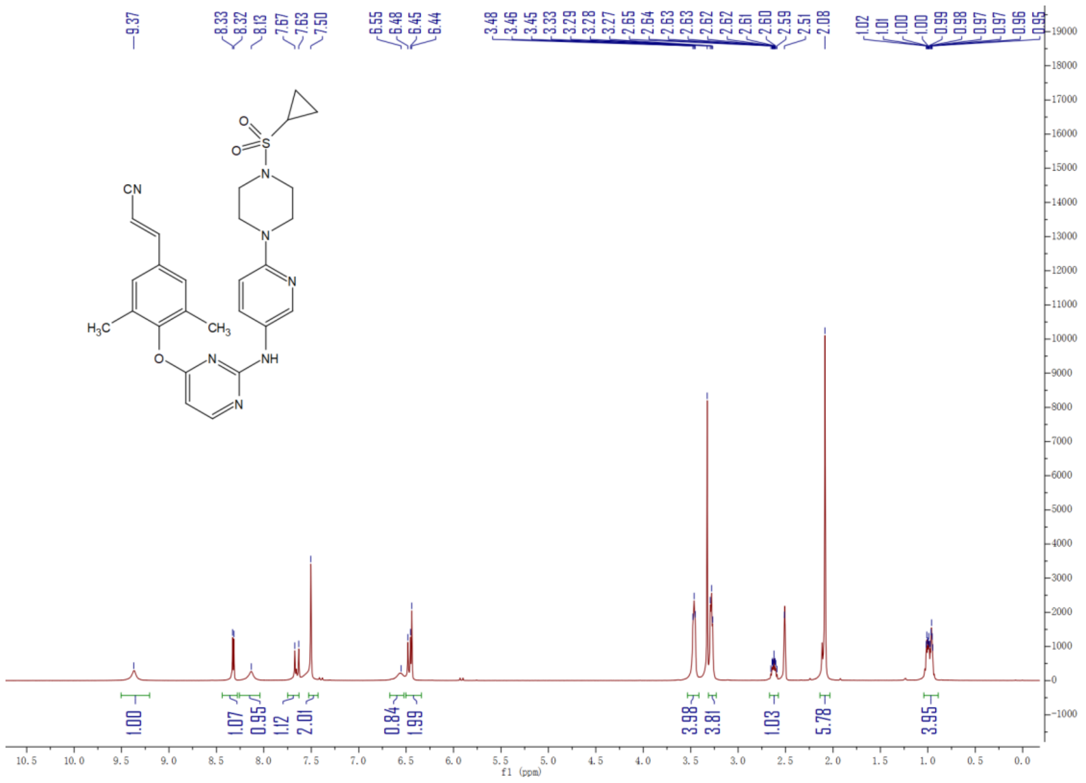

^{1}H NMR spectrum of **18a5**

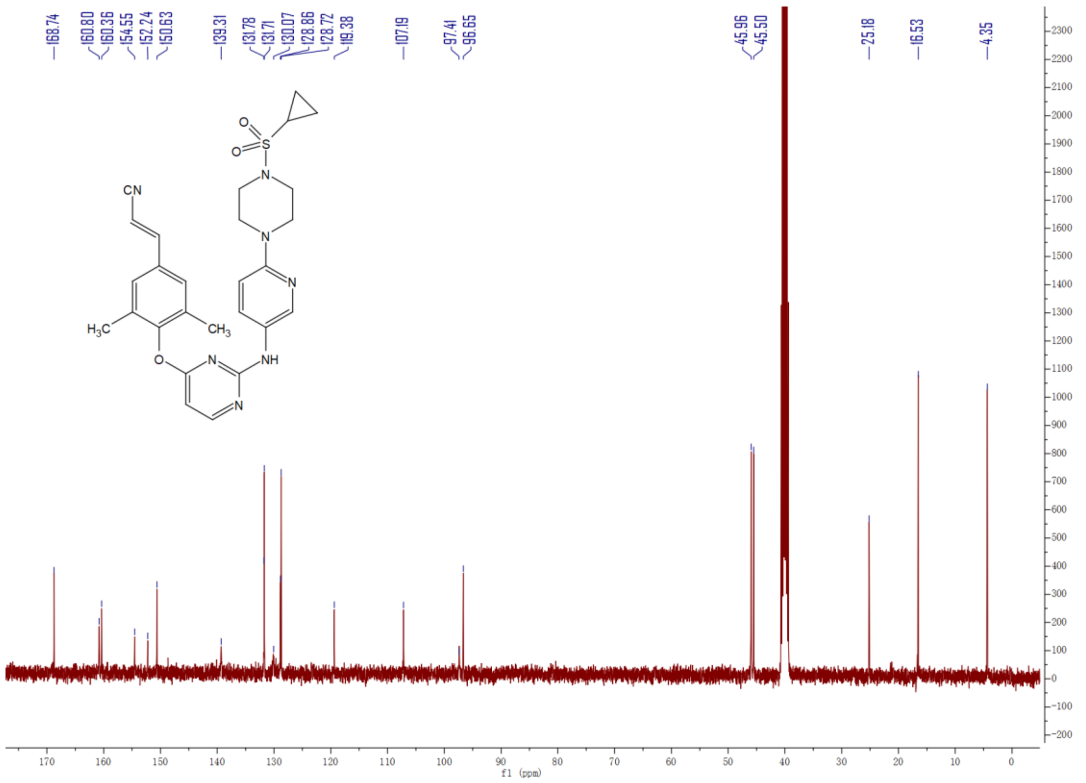

^{13}C NMR spectrum of **18a5**

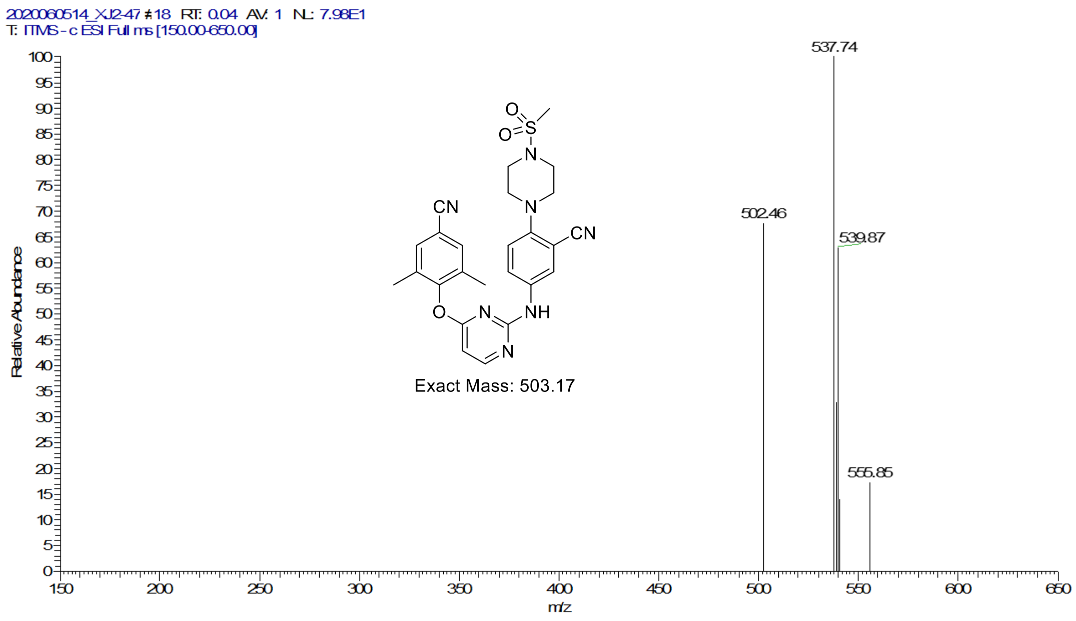

MS spectrum of **17b1**

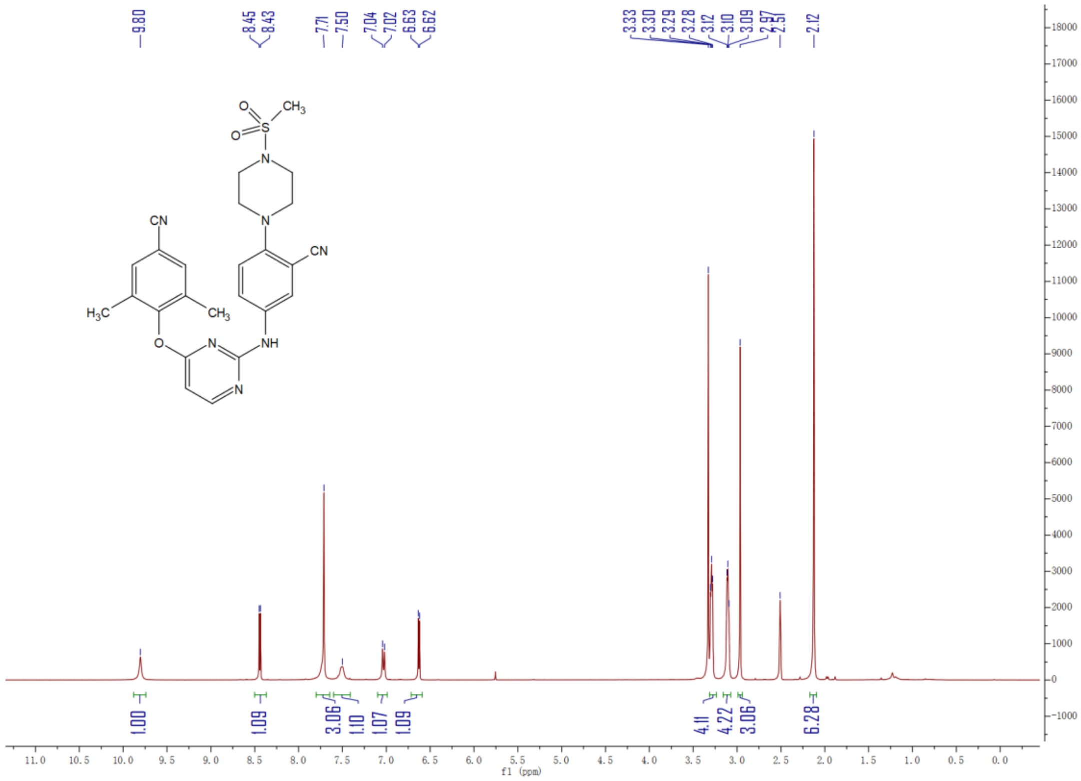

^{1}H NMR spectrum of **17b1**

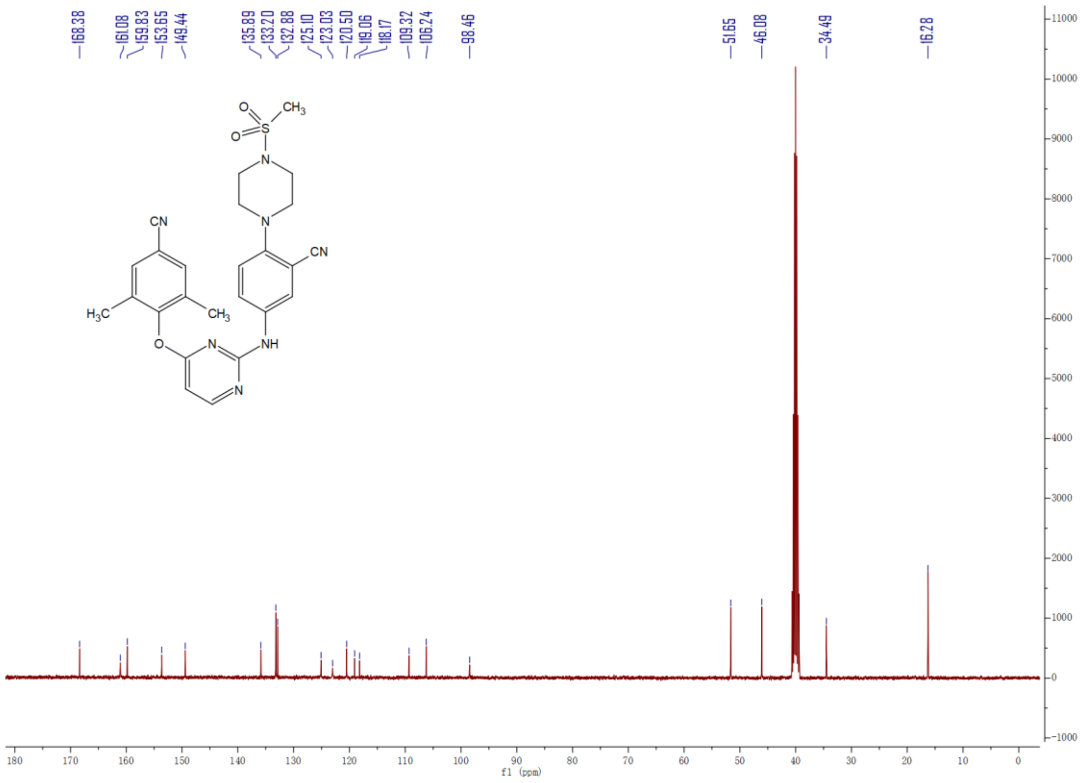

^{13}C NMR spectrum of **17b1**

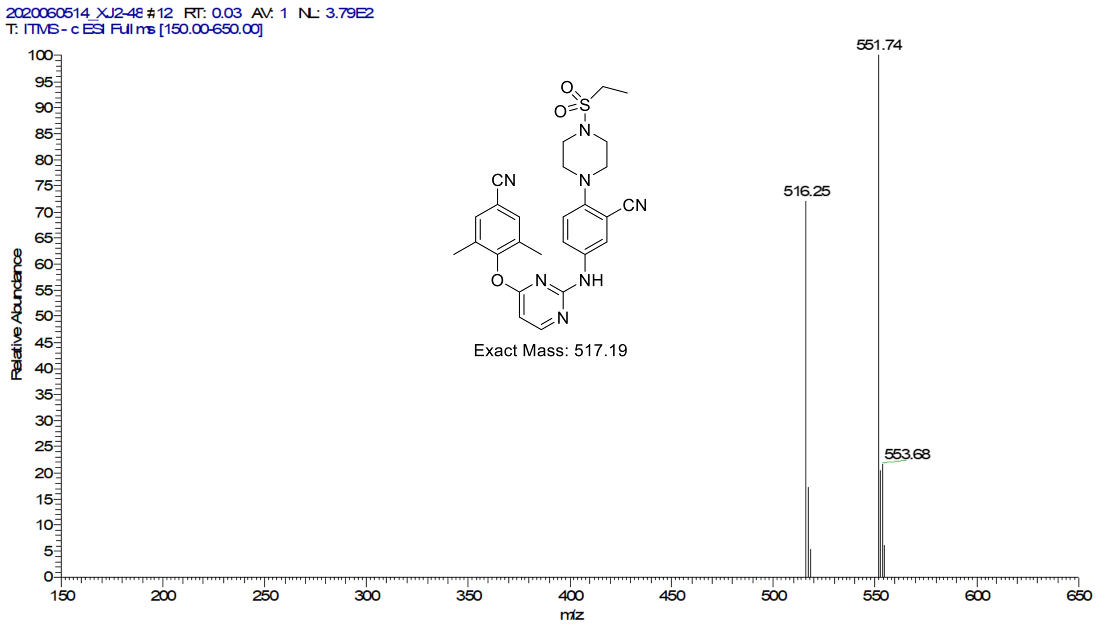

MS spectrum of **17b2**

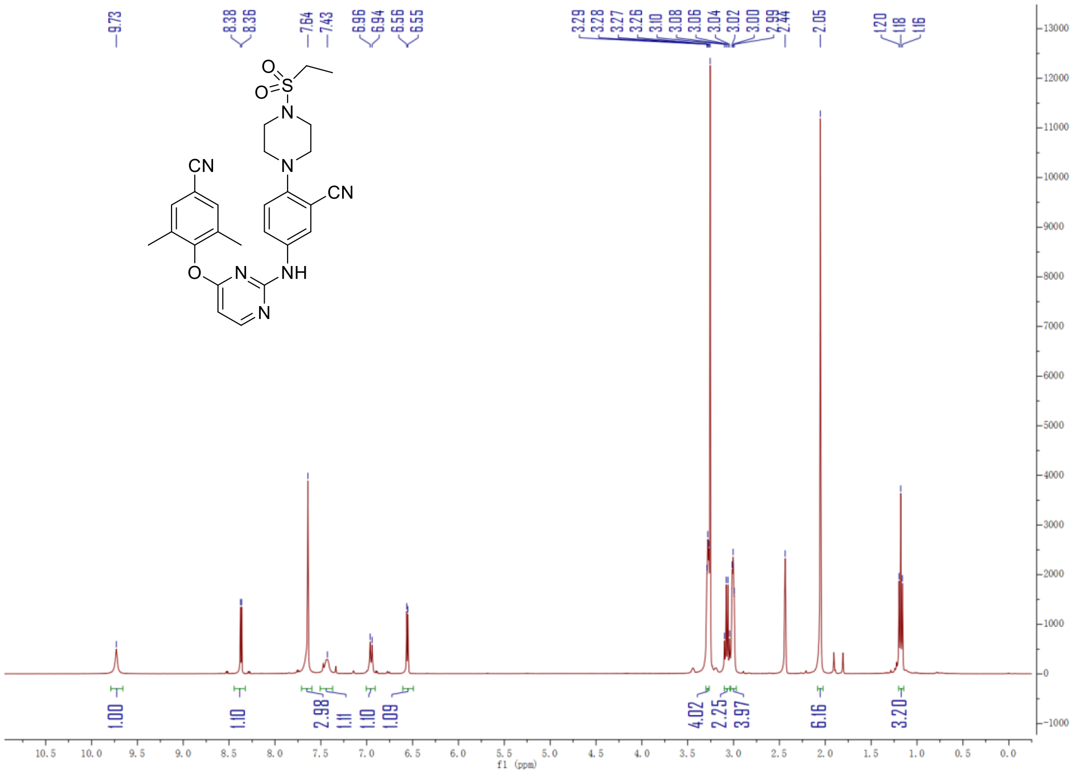

^{1}H NMR spectrum of **17b2**

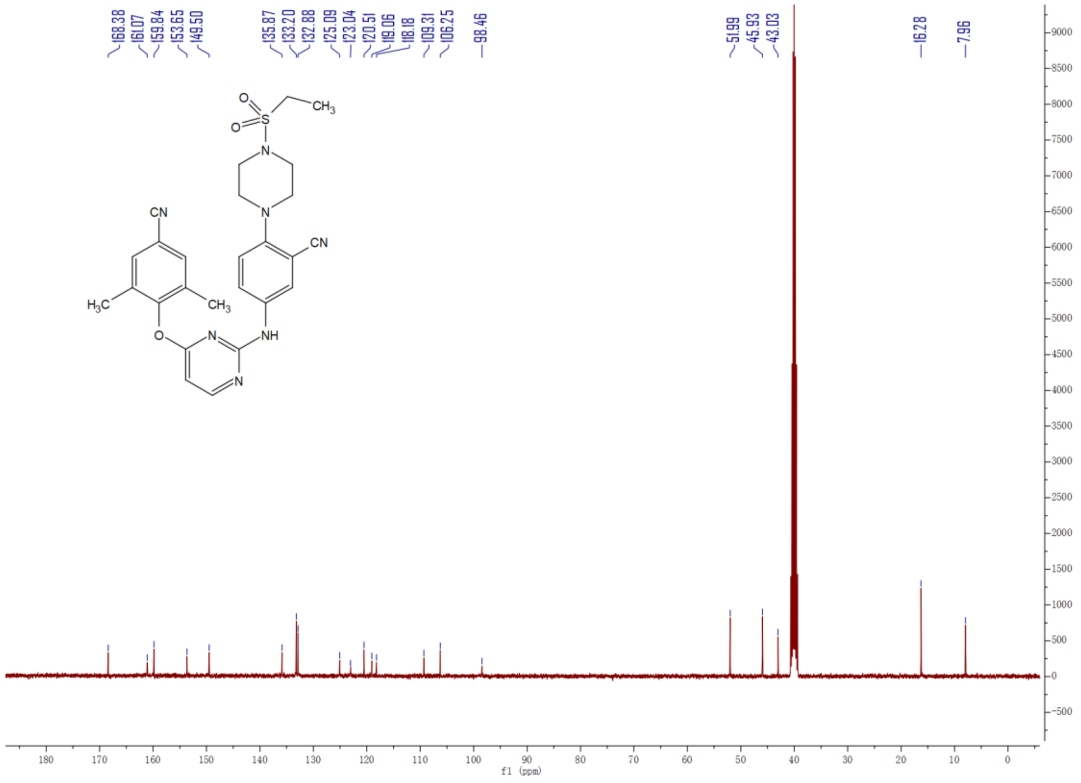

^{13}C NMR spectrum of **17b2**

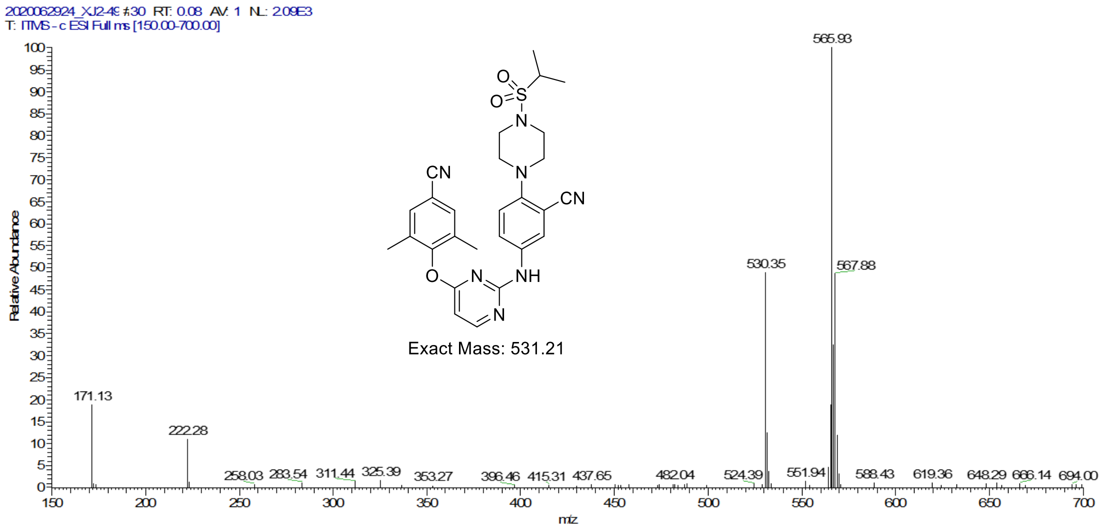

MS spectrum of **17b3**

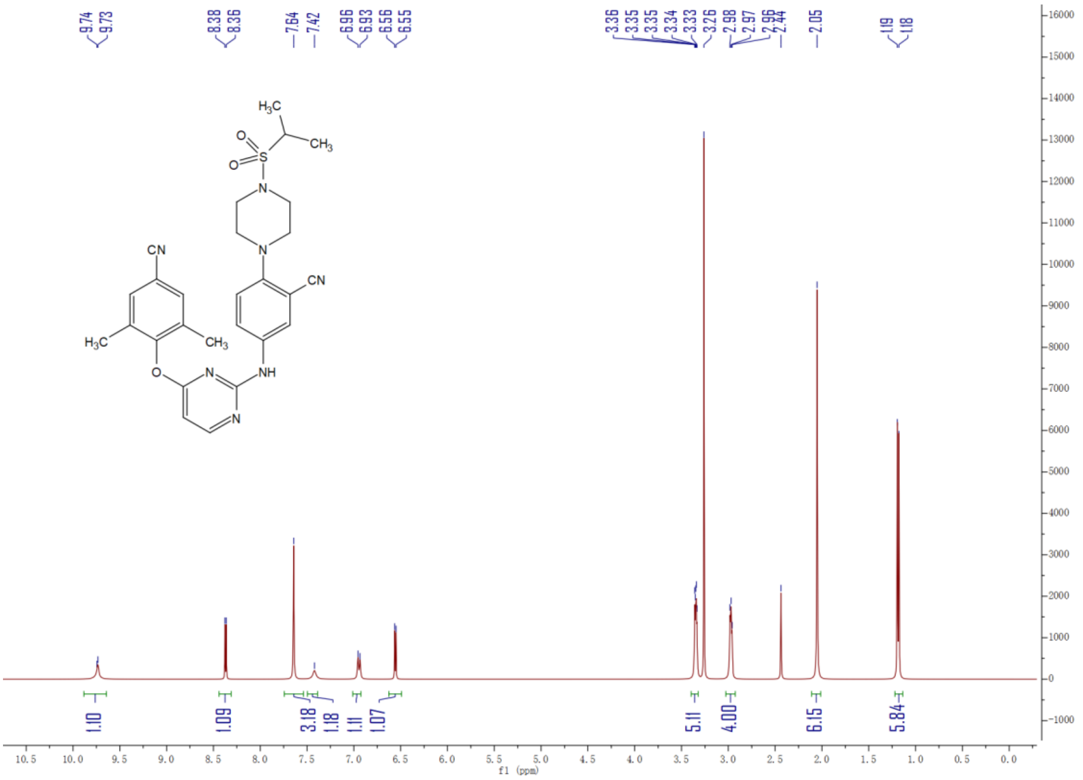

^{1}H NMR spectrum of **17b3**

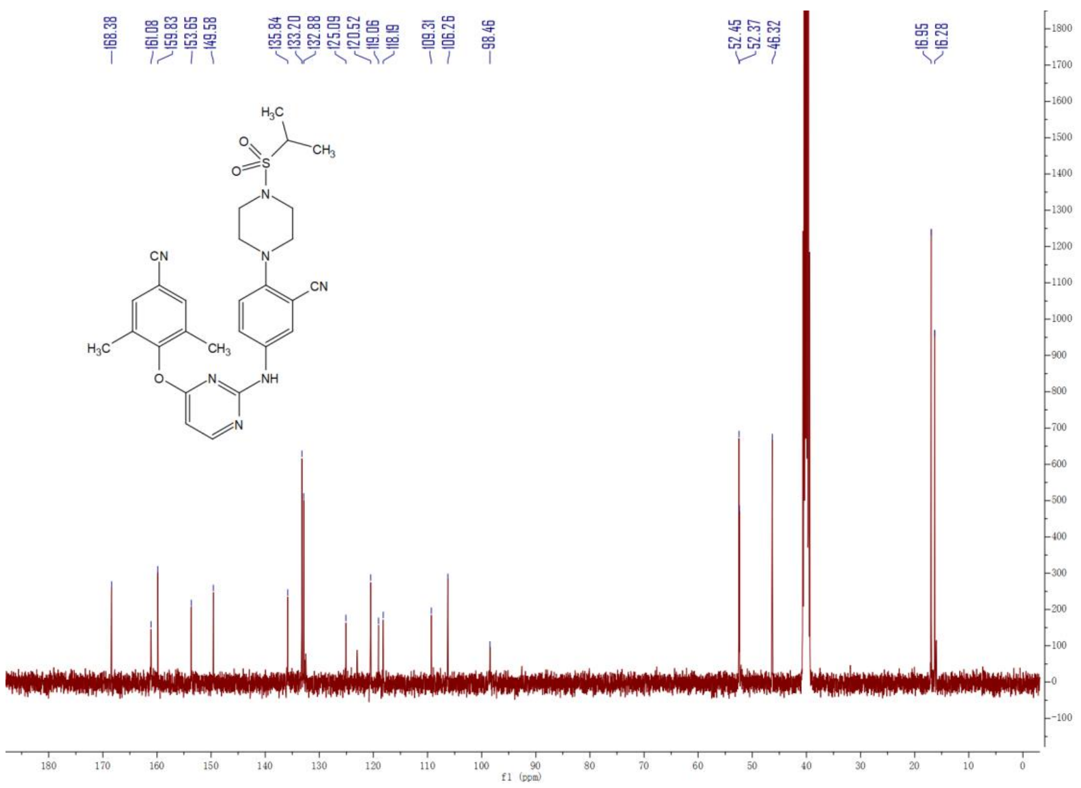

^{13}C NMR spectrum of **17b3**

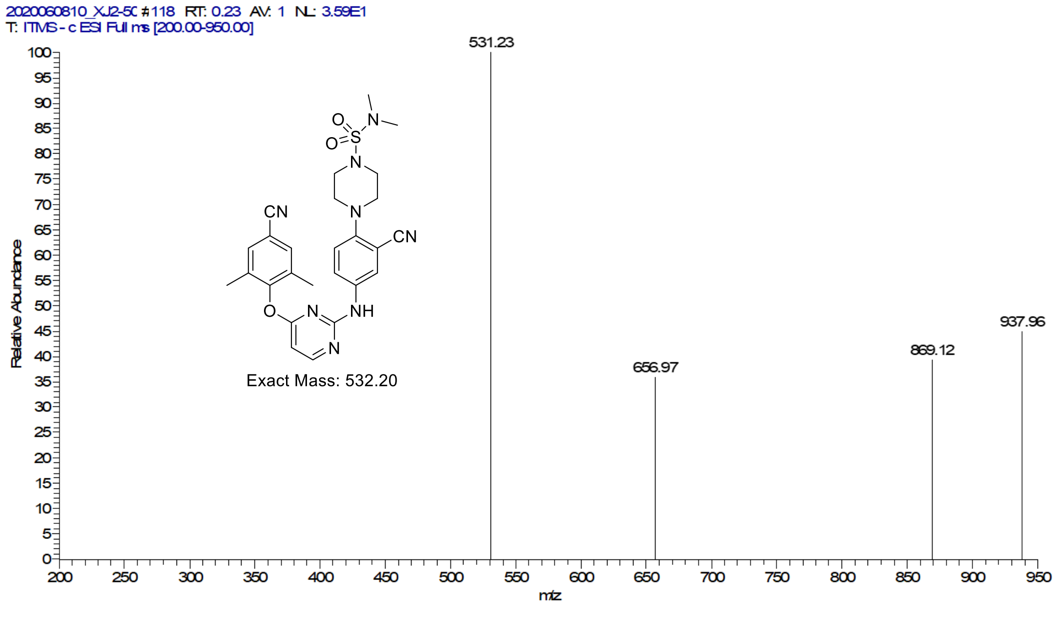

MS spectrum of **17b4**

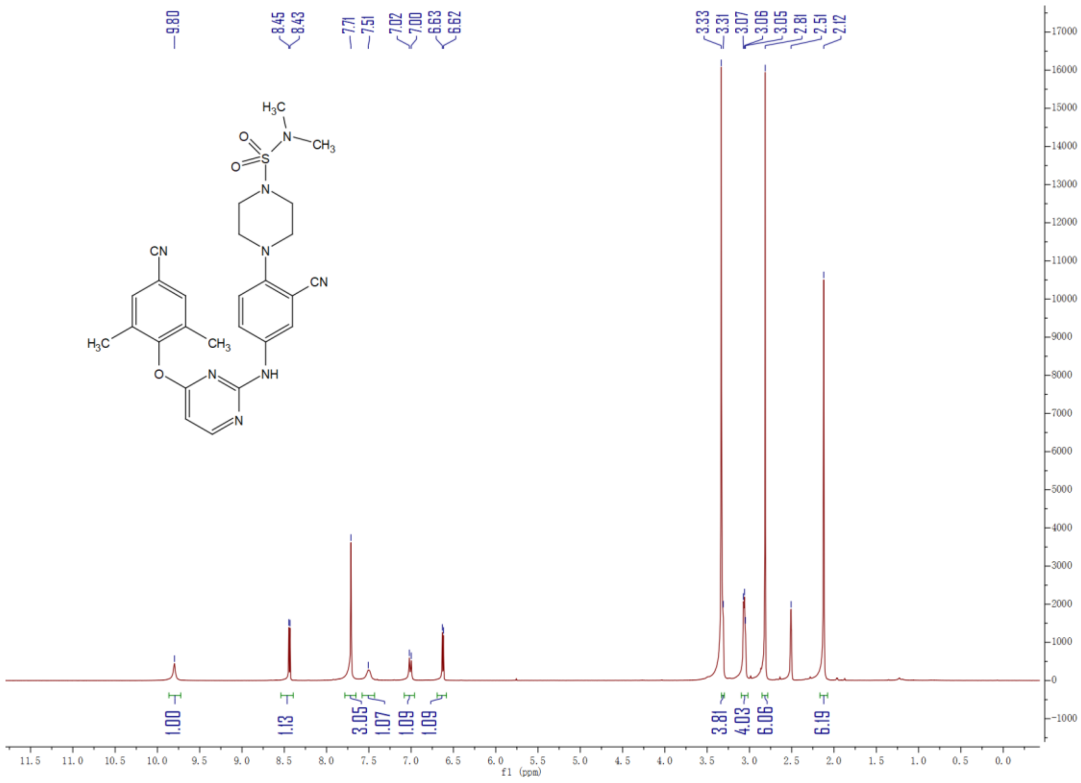

^{1}H NMR spectrum of **17b4**

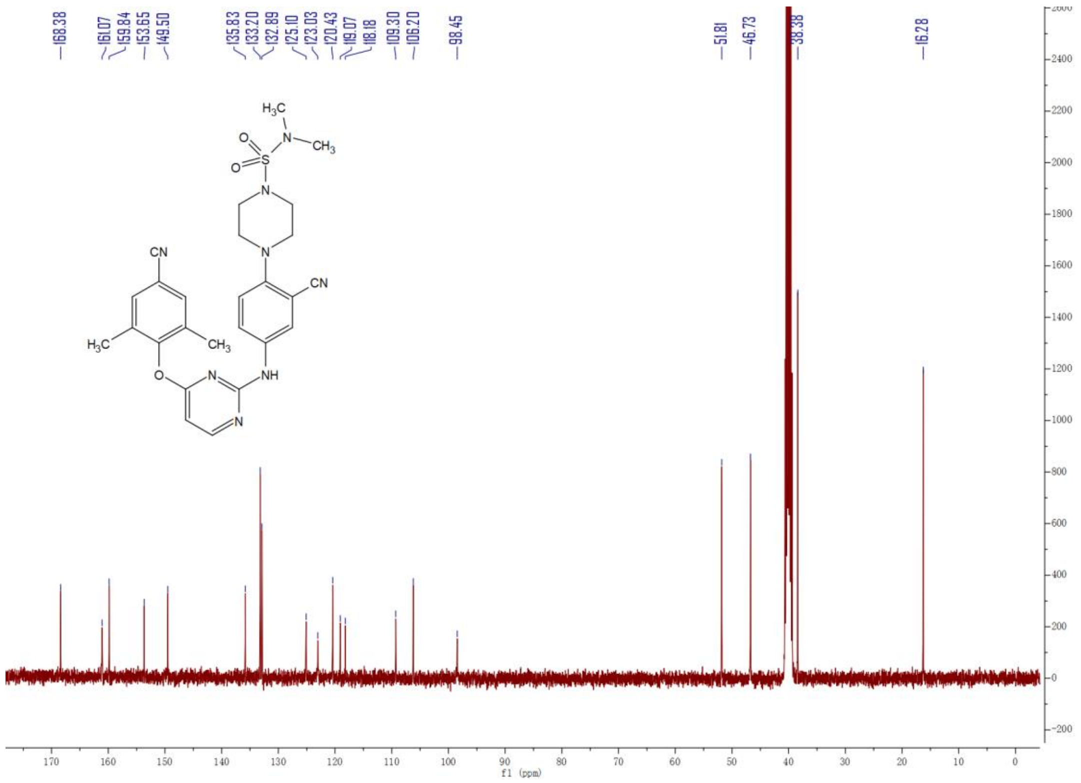

^{13}C NMR spectrum of **17b4**

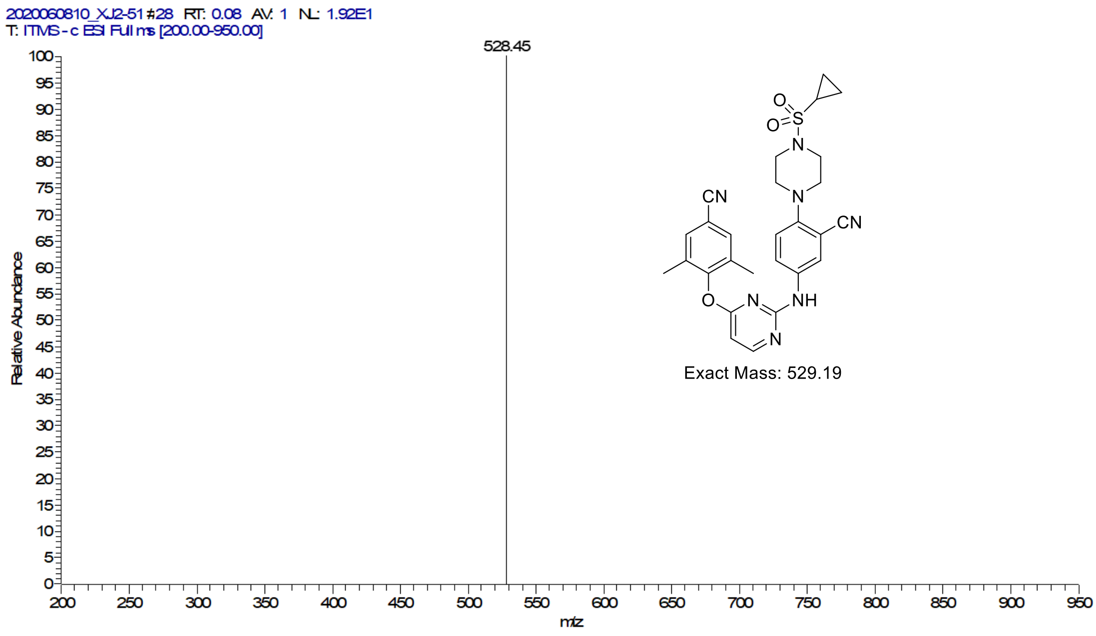

MS spectrum of **17b5**

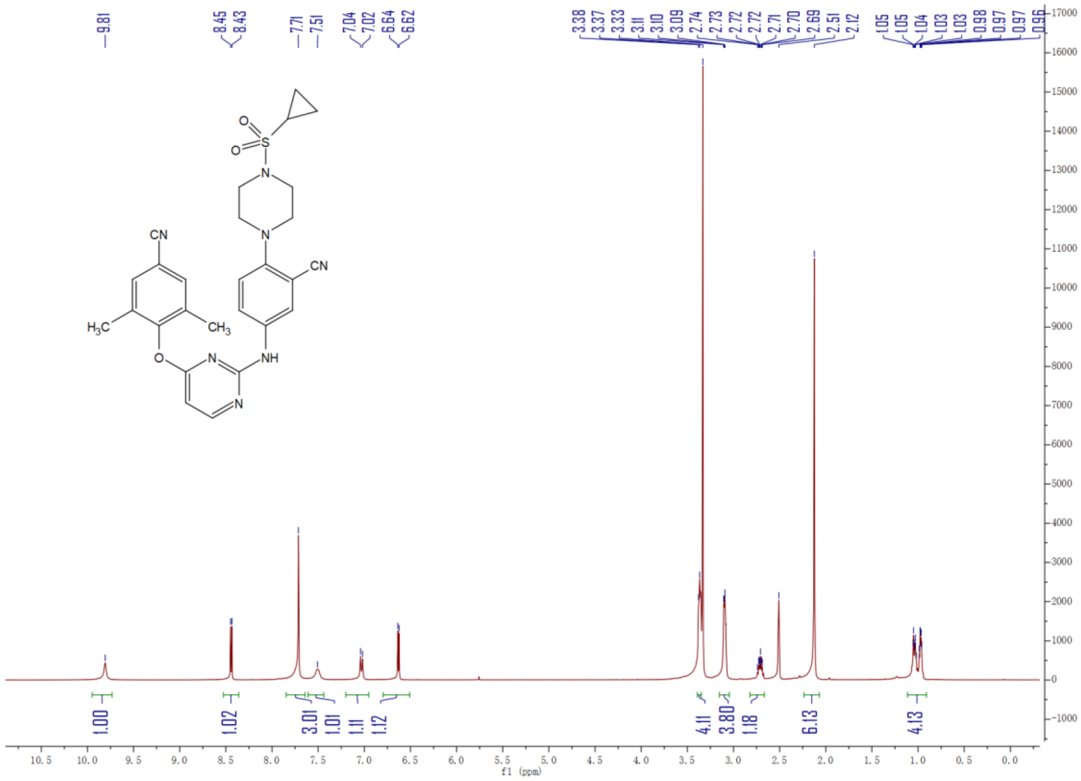

^{1}H NMR spectrum of **17b5**

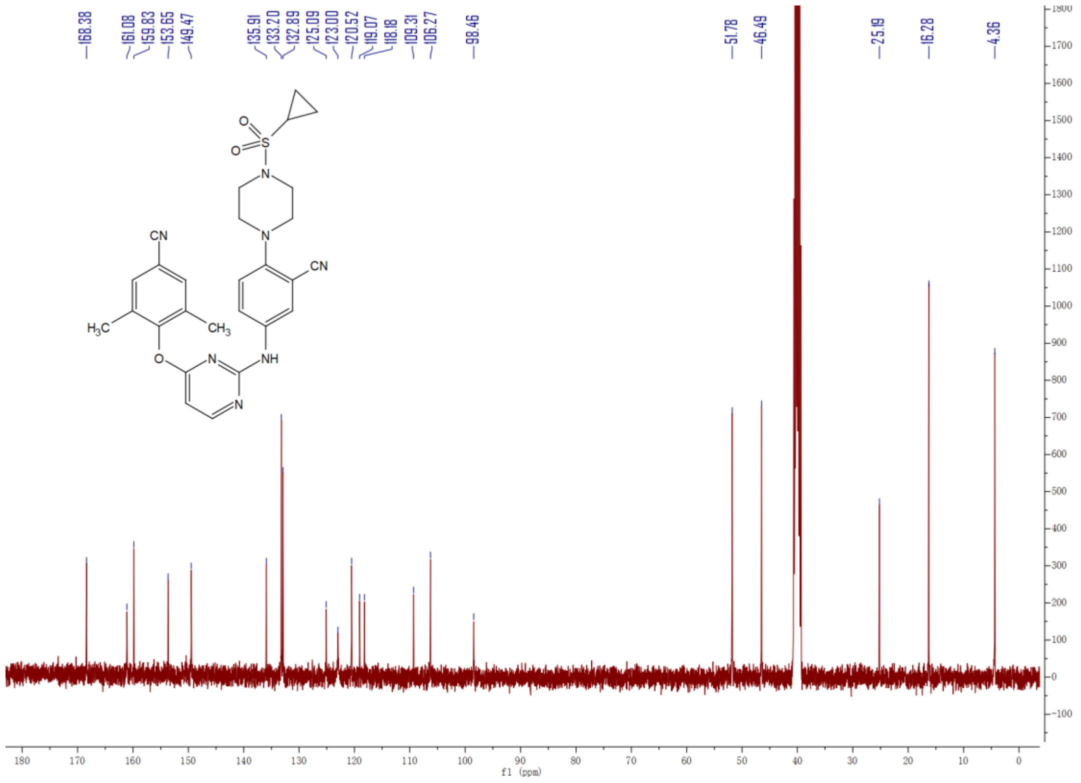

^{13}C NMR spectrum of **17b5**

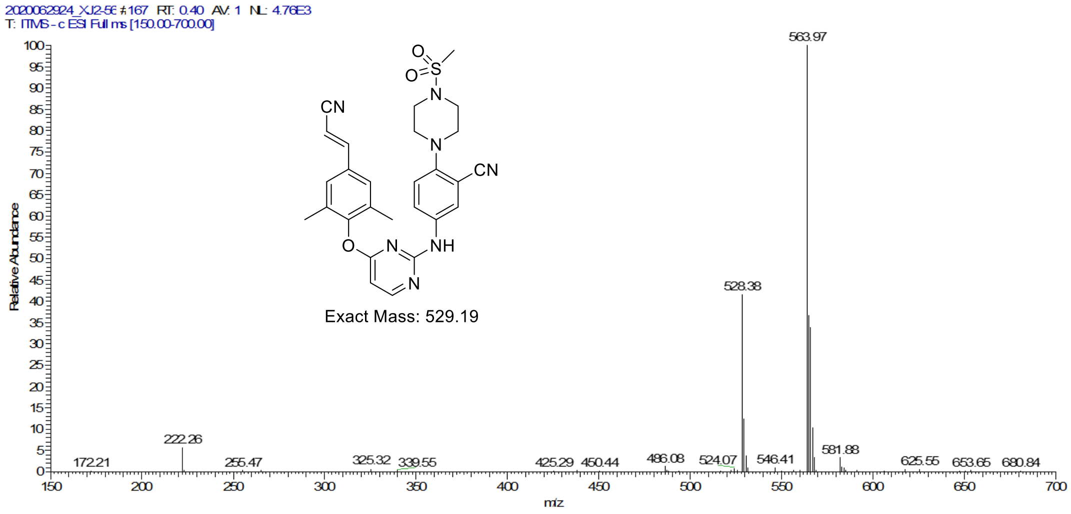

MS spectrum of **18b1**

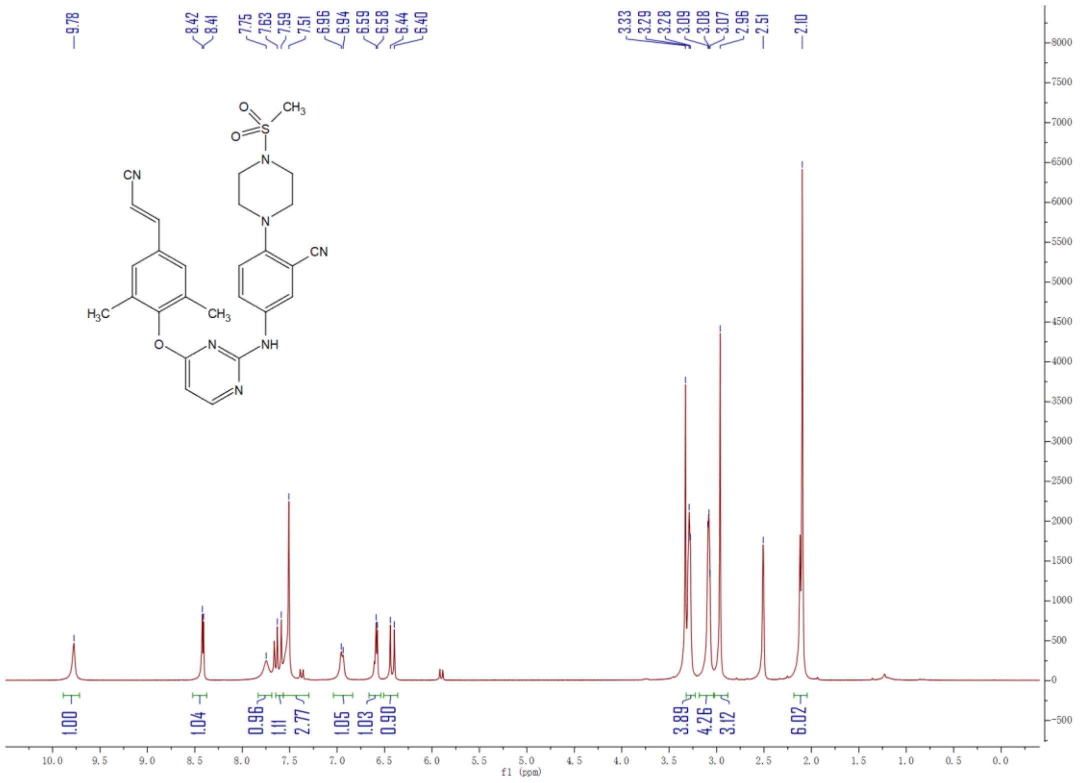

^{1}H NMR spectrum of **18b1**

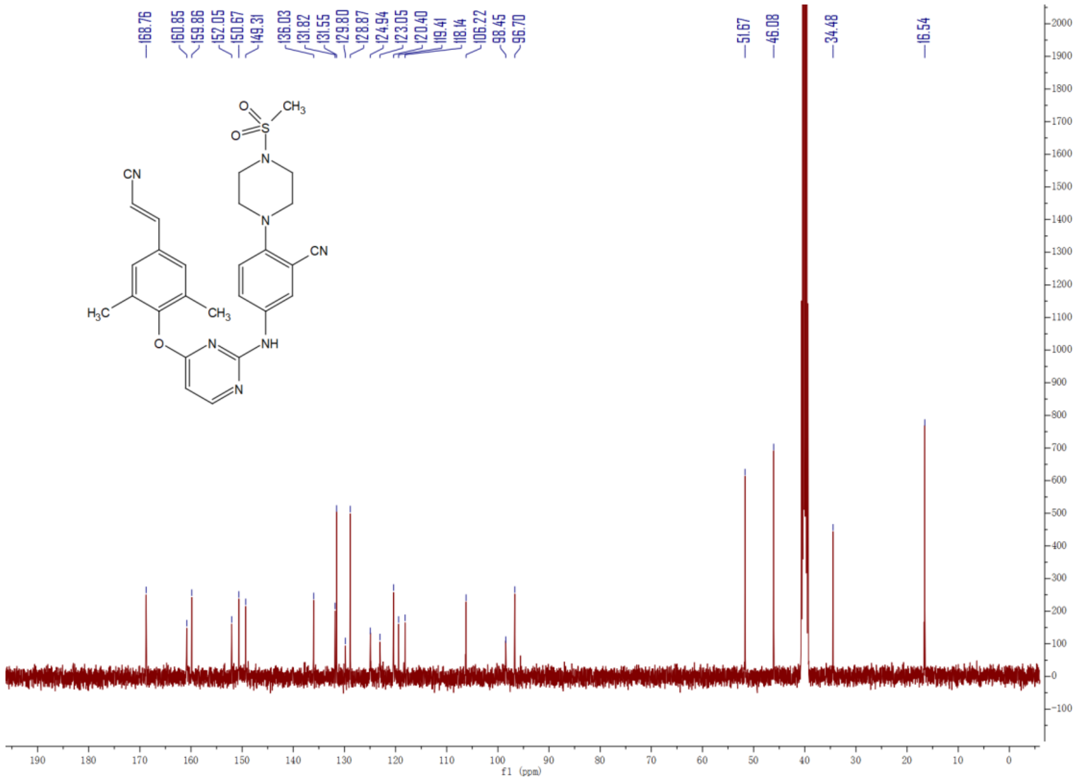

^{13}C NMR spectrum of **18b1**

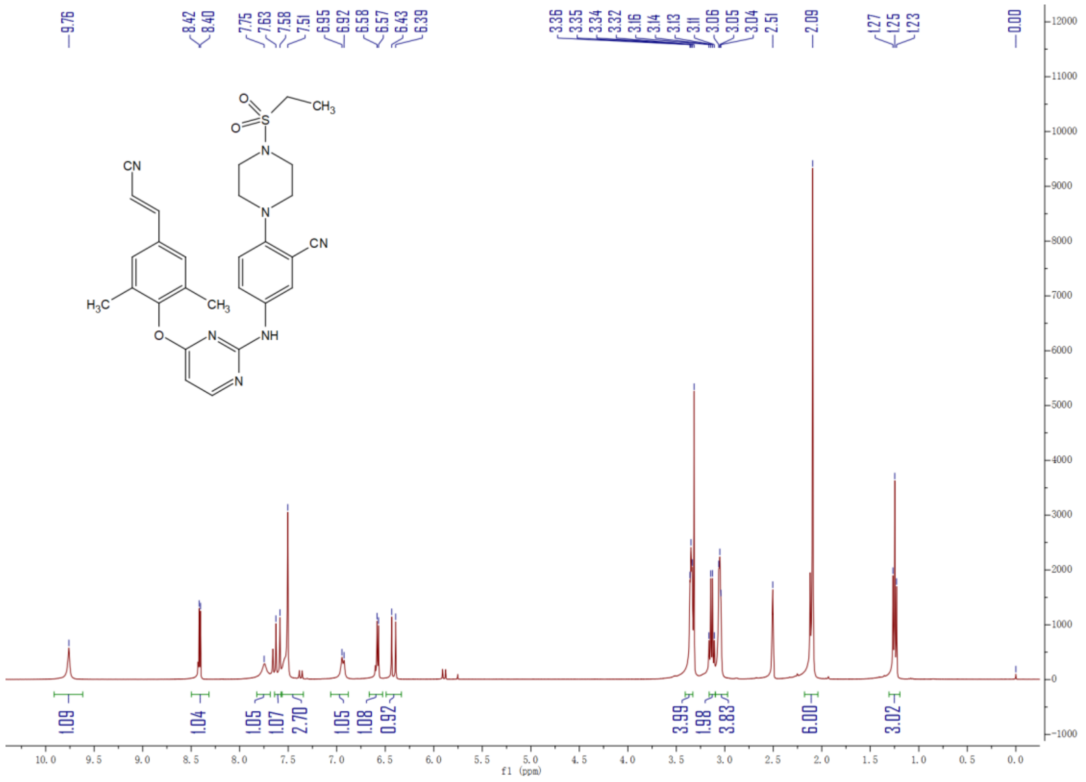

^{1}H NMR spectrum of **18b2**

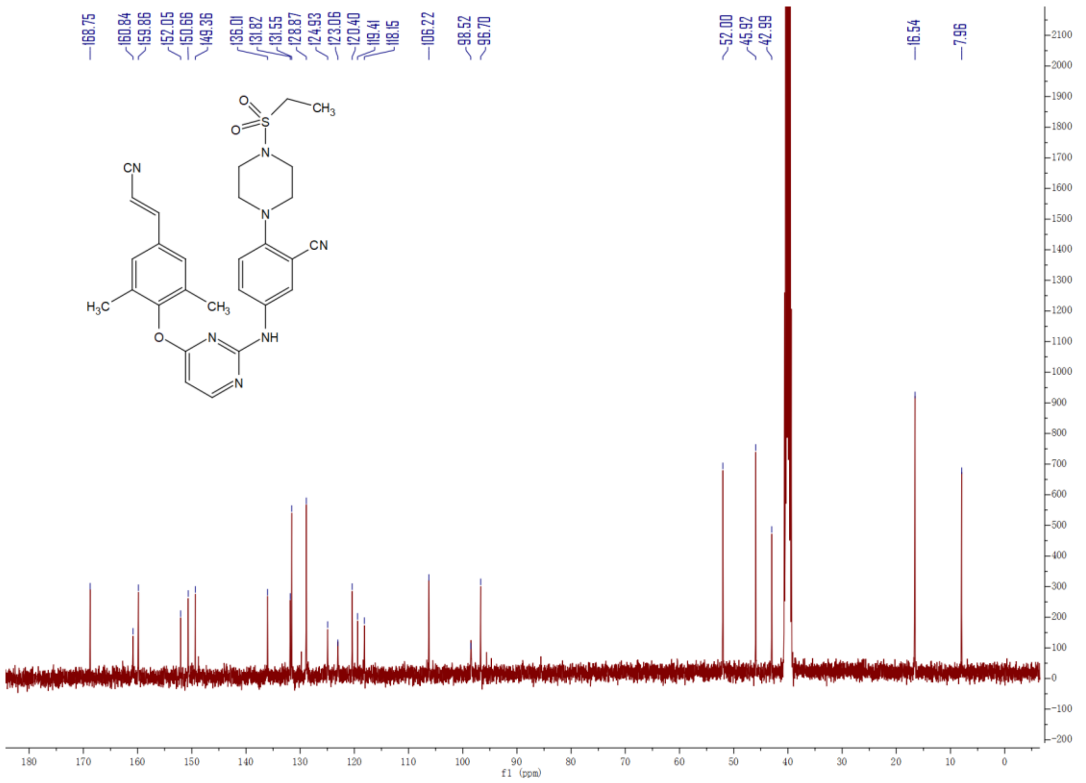

^{13}C NMR spectrum of **18b2**

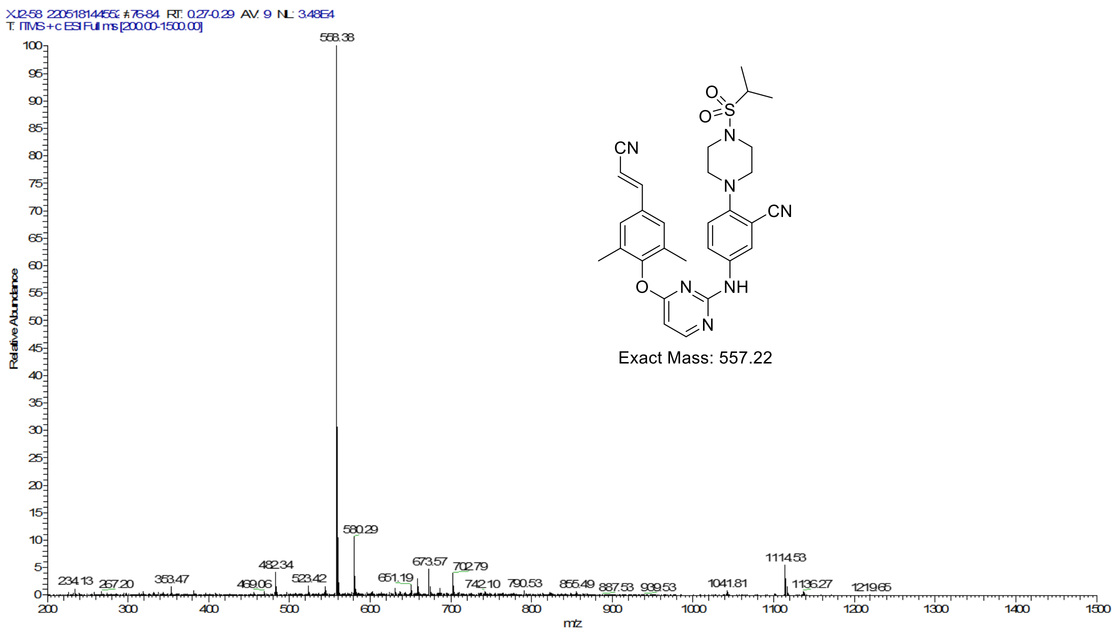

MS spectrum of **18b3**

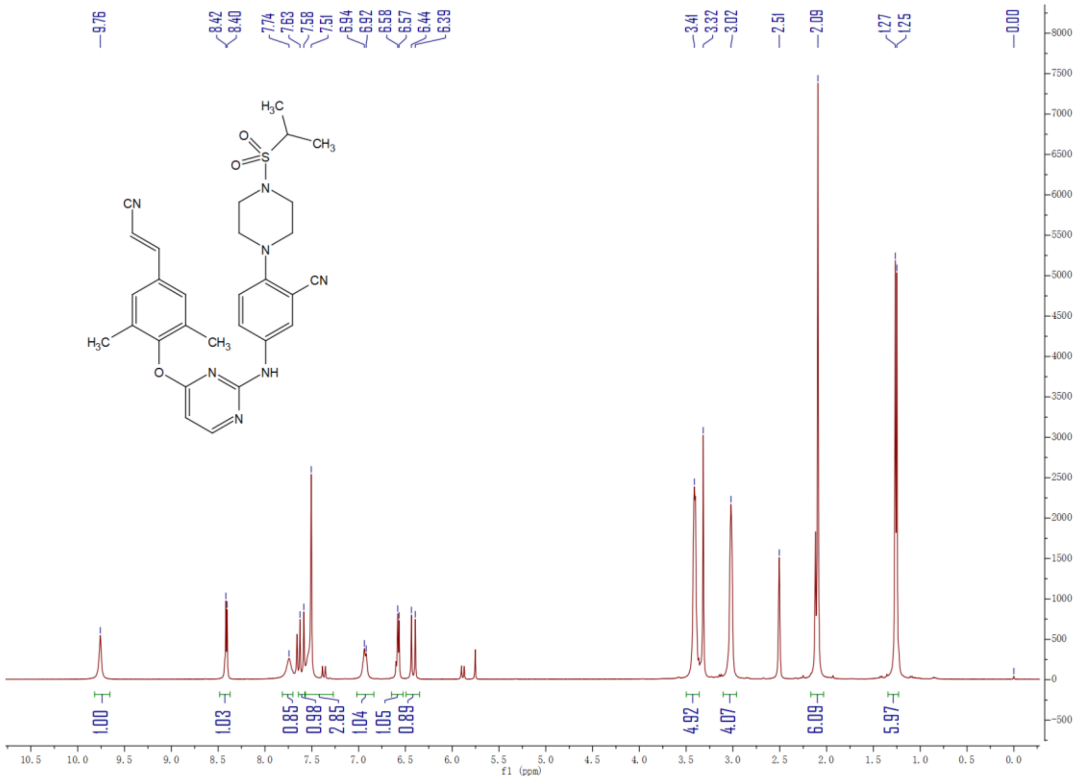

^{1}H NMR spectrum of **18b3**

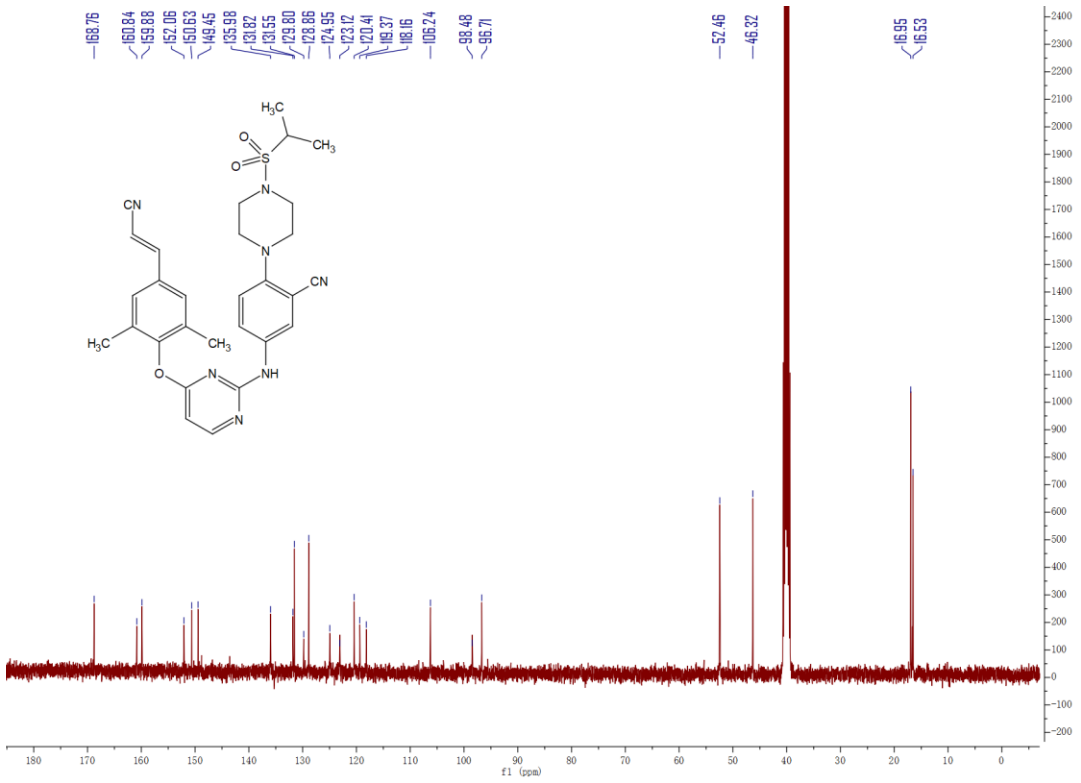

^{13}C NMR spectrum of **18b3**

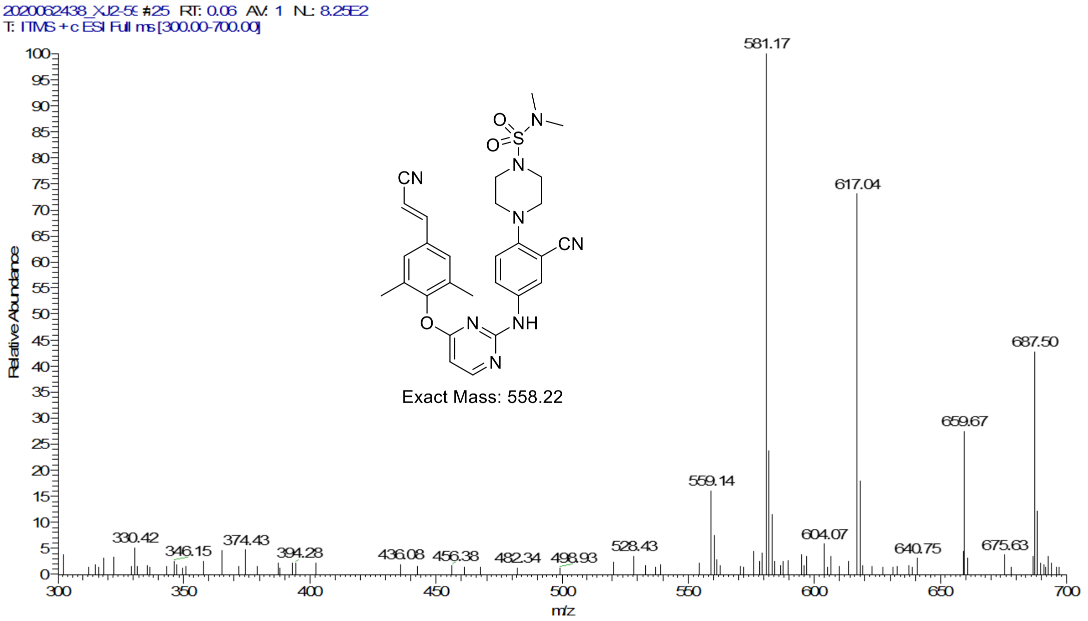

MS spectrum of **18b4**

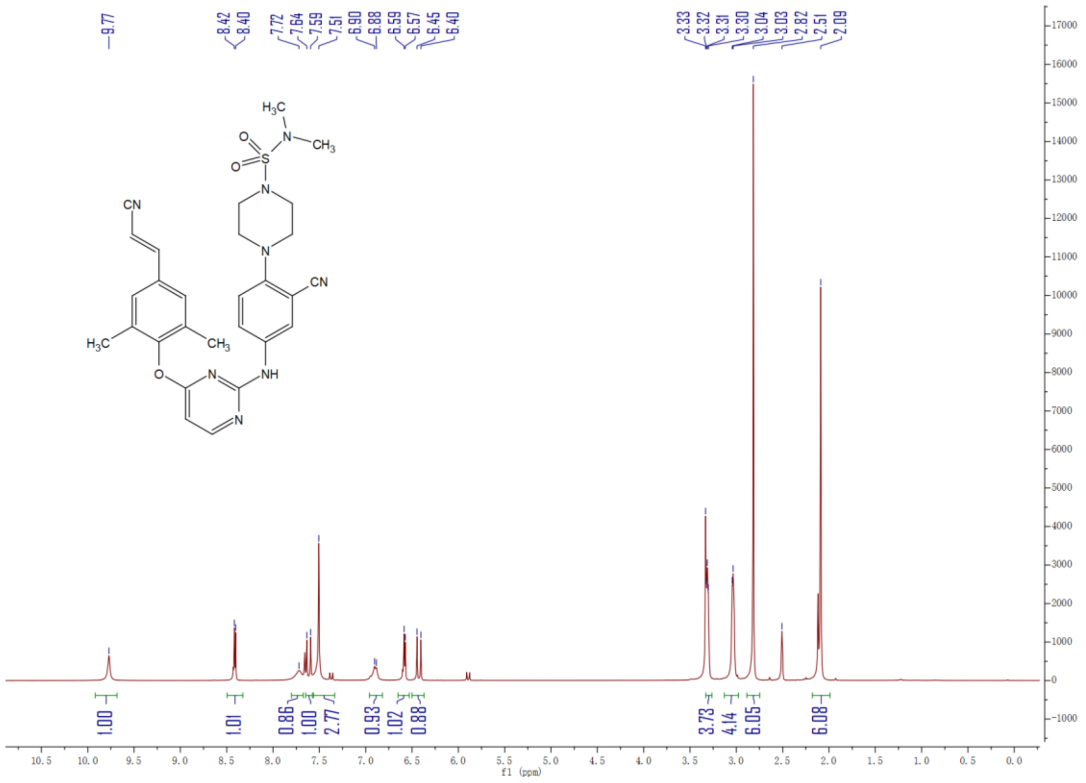

^{1}H NMR spectrum of **17b4**

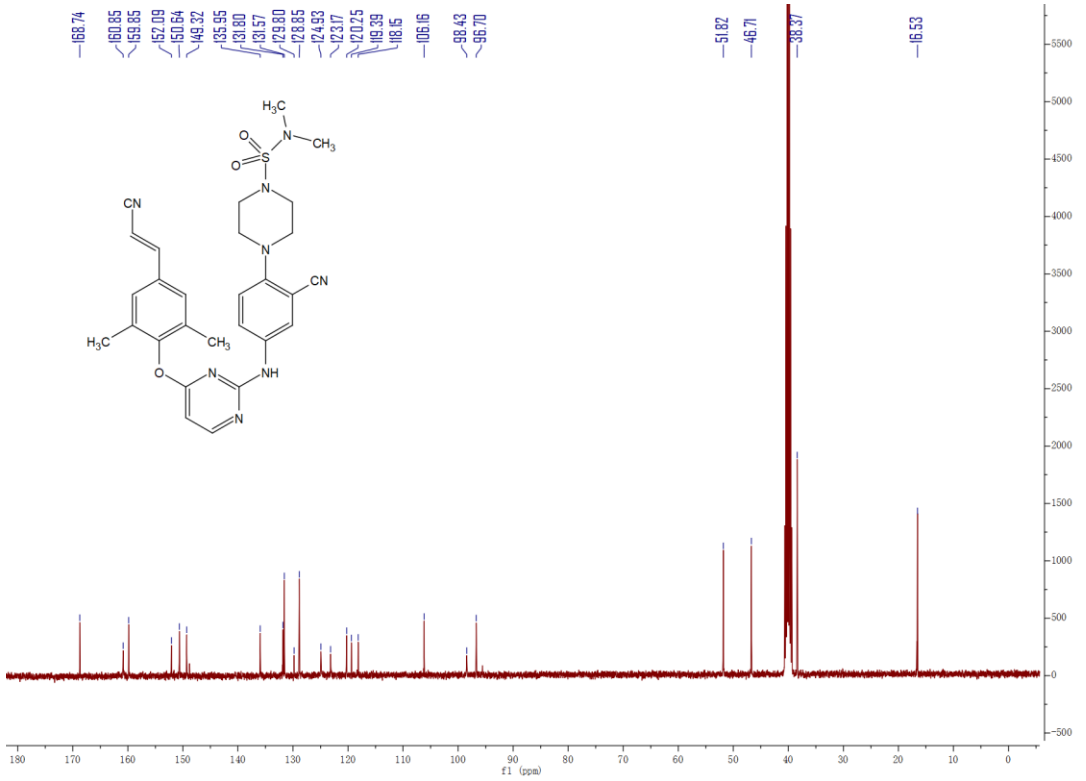

^{13}C NMR spectrum of **18b4**

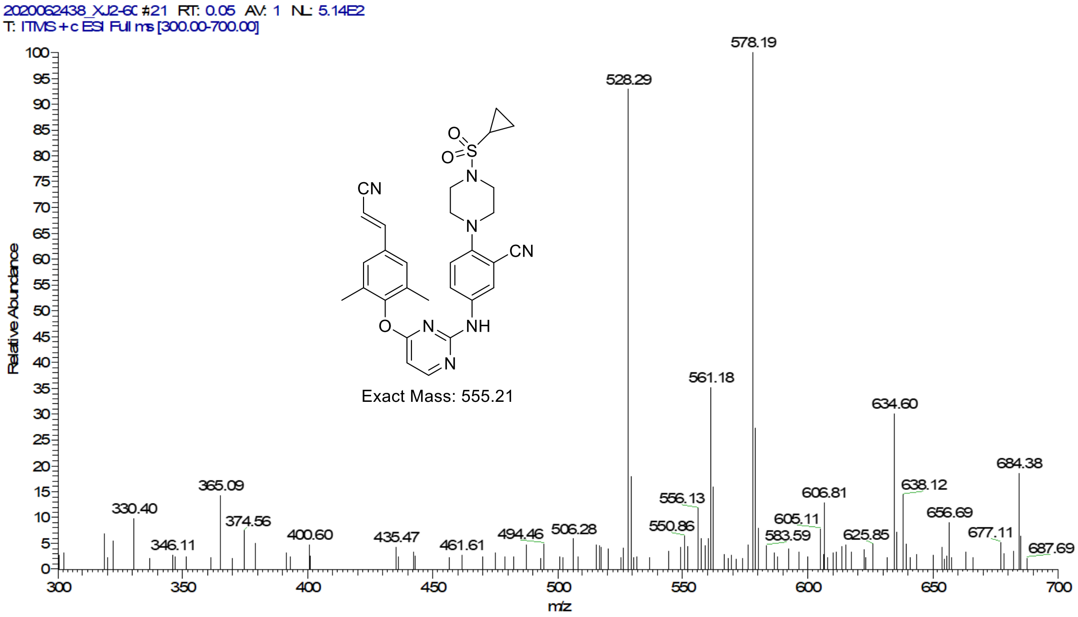

MS spectrum of **18b5**

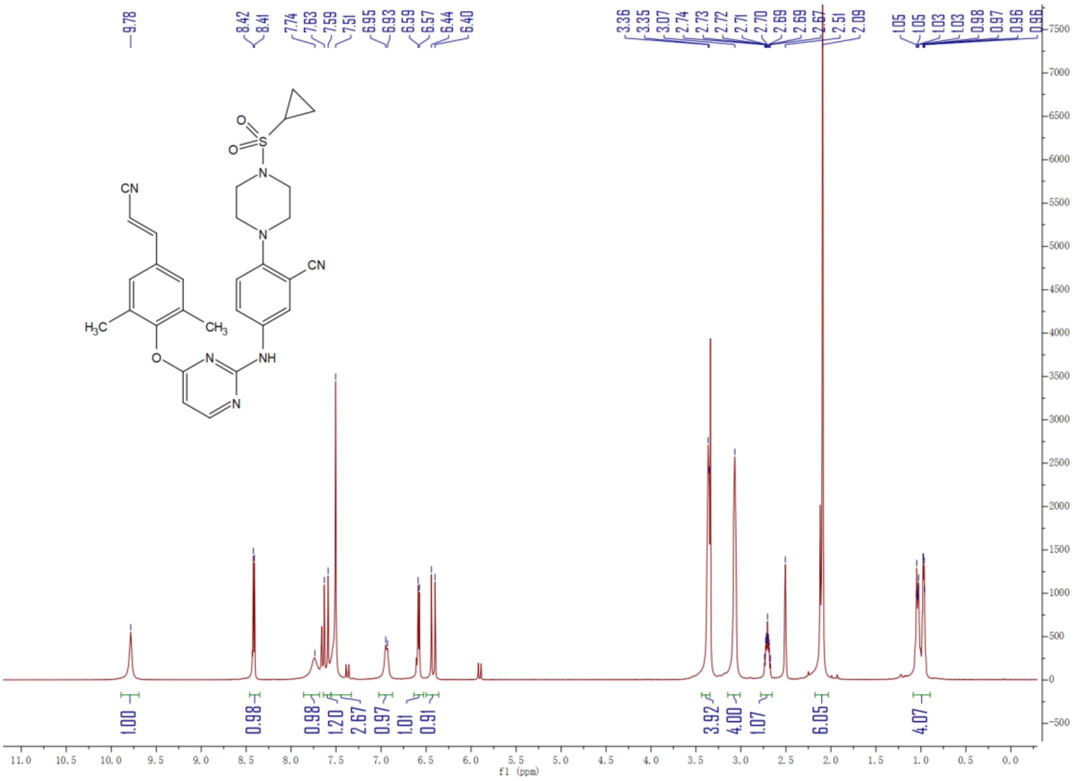

^{1}H NMR spectrum of **17b5**

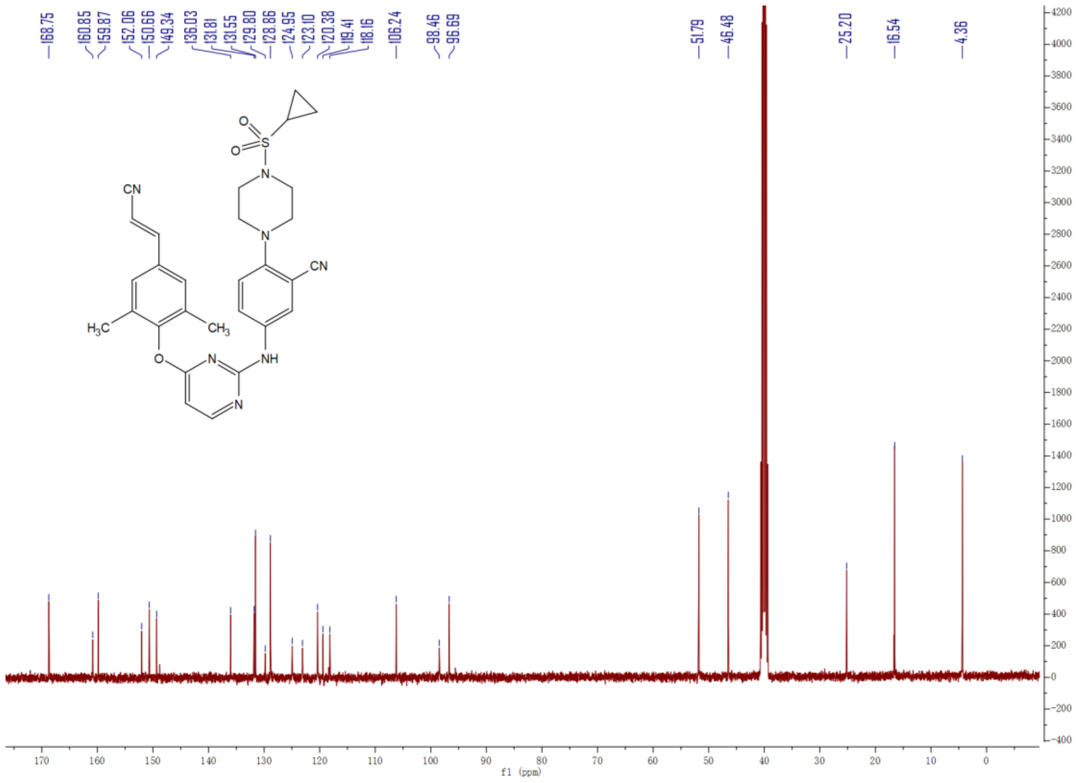

^{13}C NMR spectrum of **18b5**

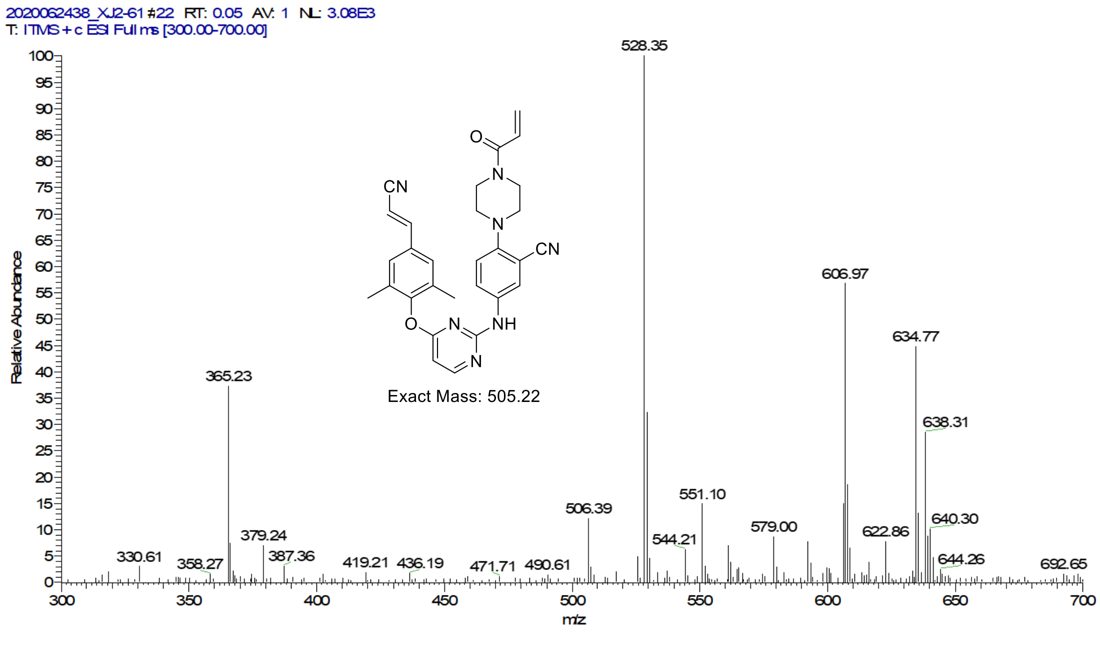

MS spectrum of **18b6**

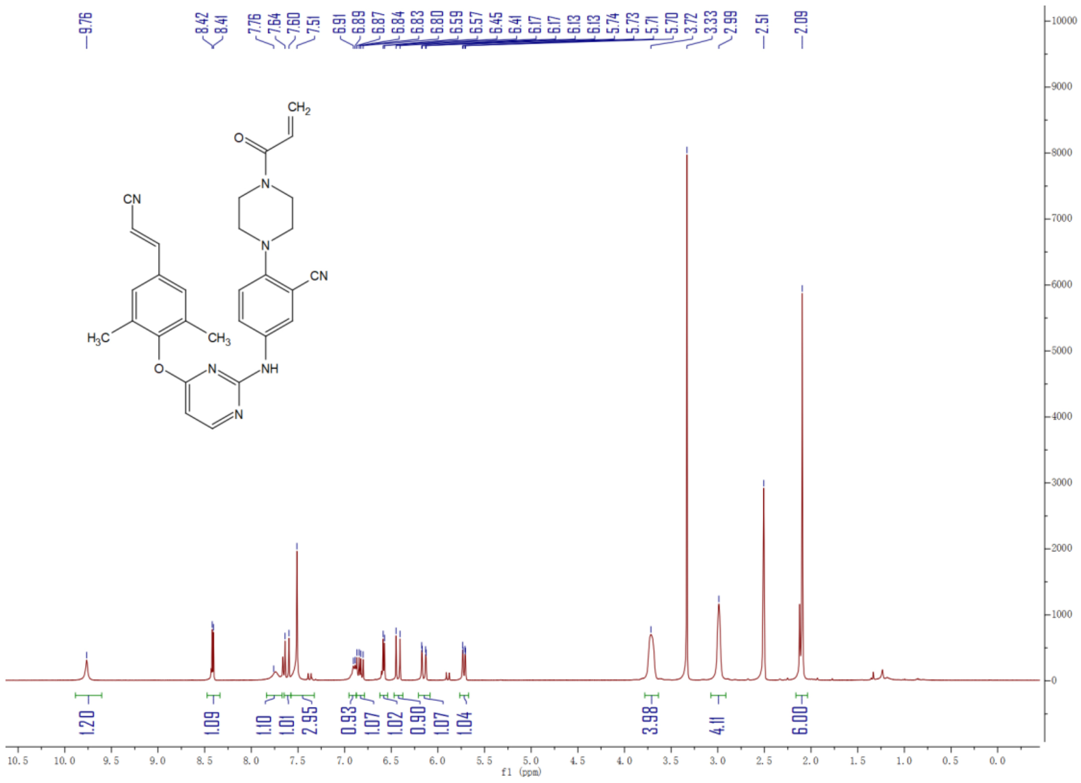

^{1}H NMR spectrum of **18b6**

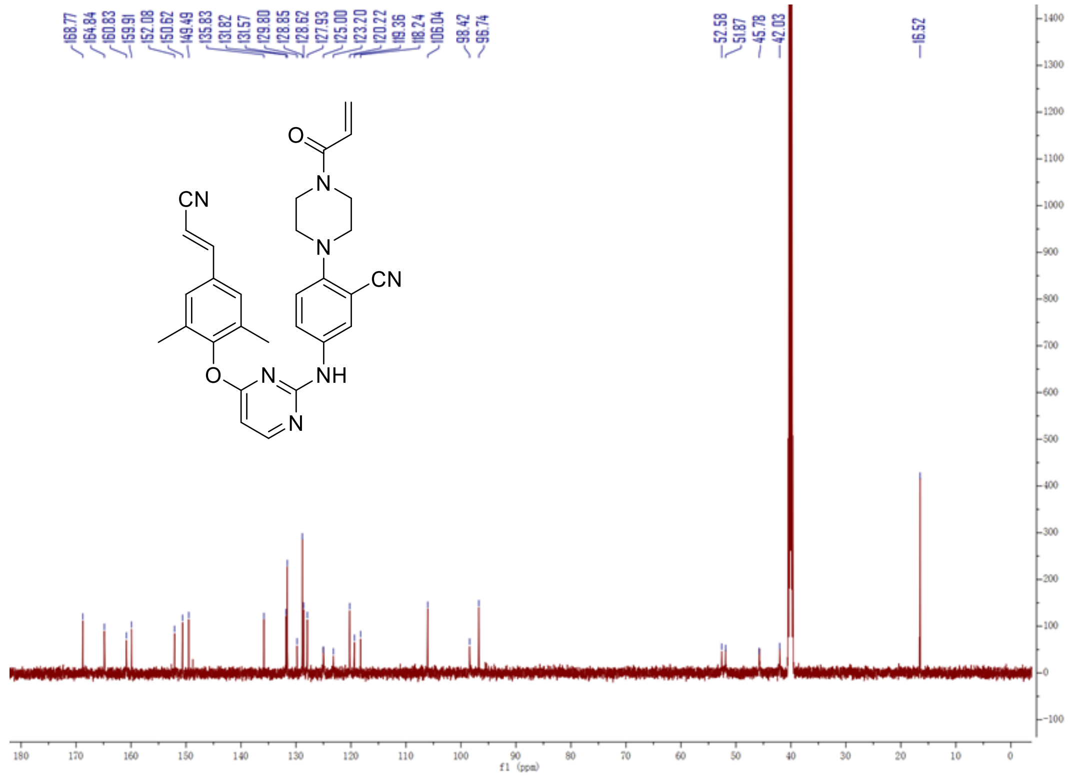

^{13}C NMR spectrum of **18b6**

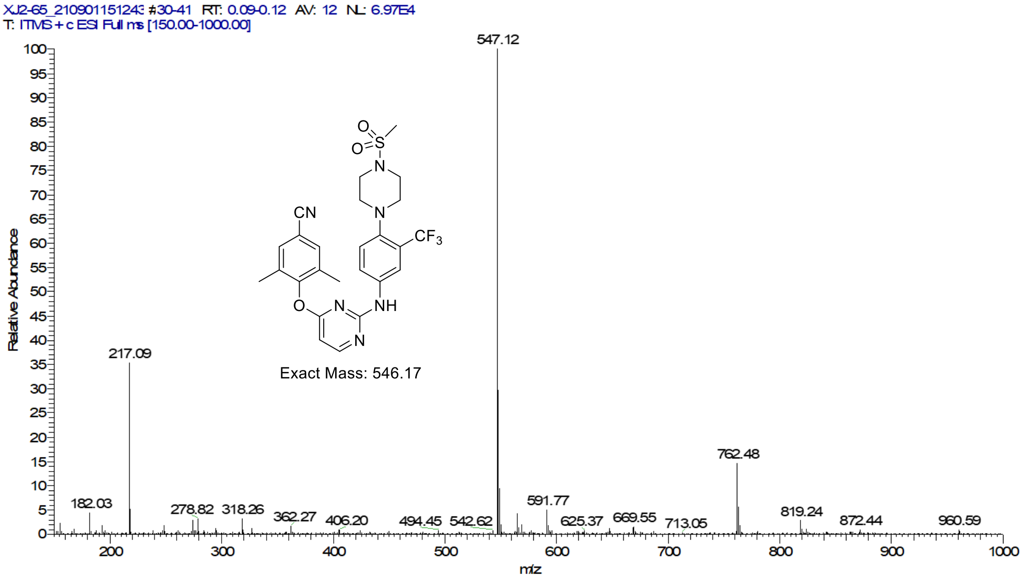

MS spectrum of **17c1**

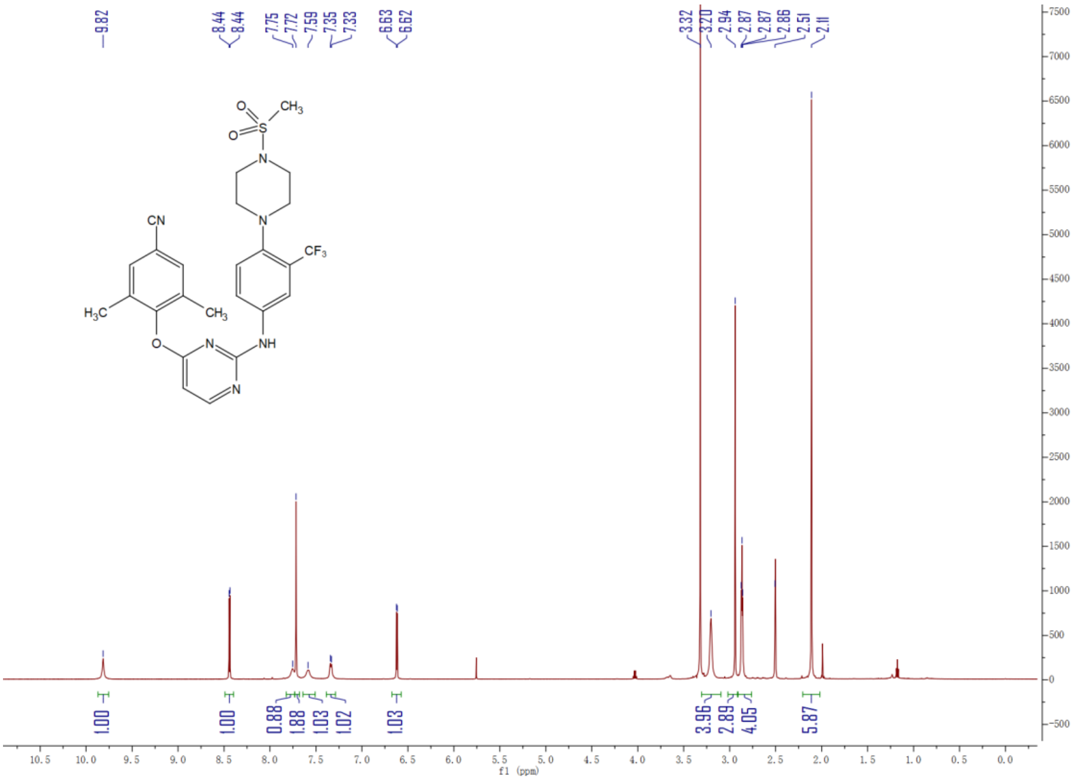

^{1}H NMR spectrum of **17c1**

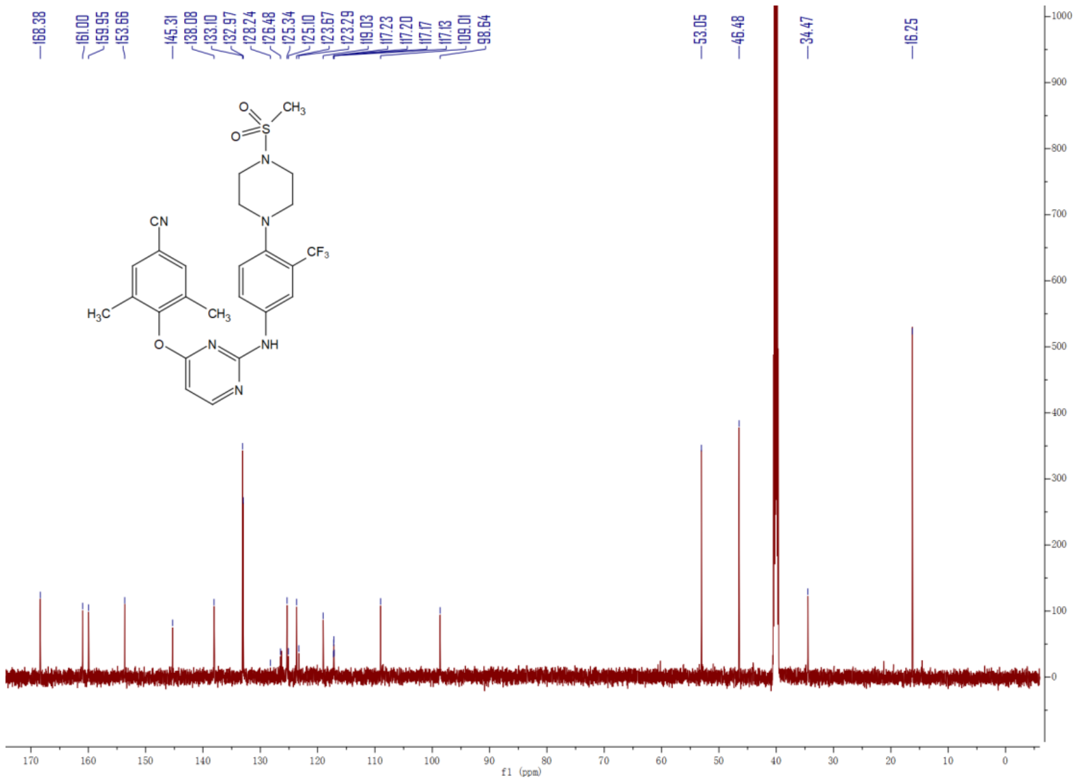

^{13}C NMR spectrum of **17c1**

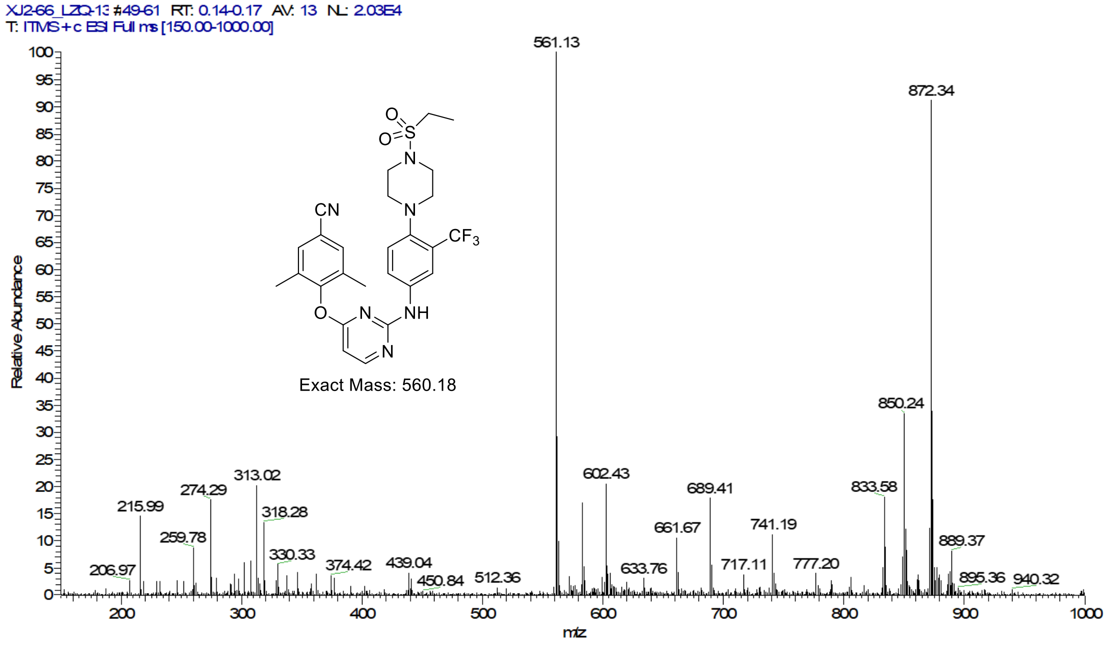

MS spectrum of **17c2**

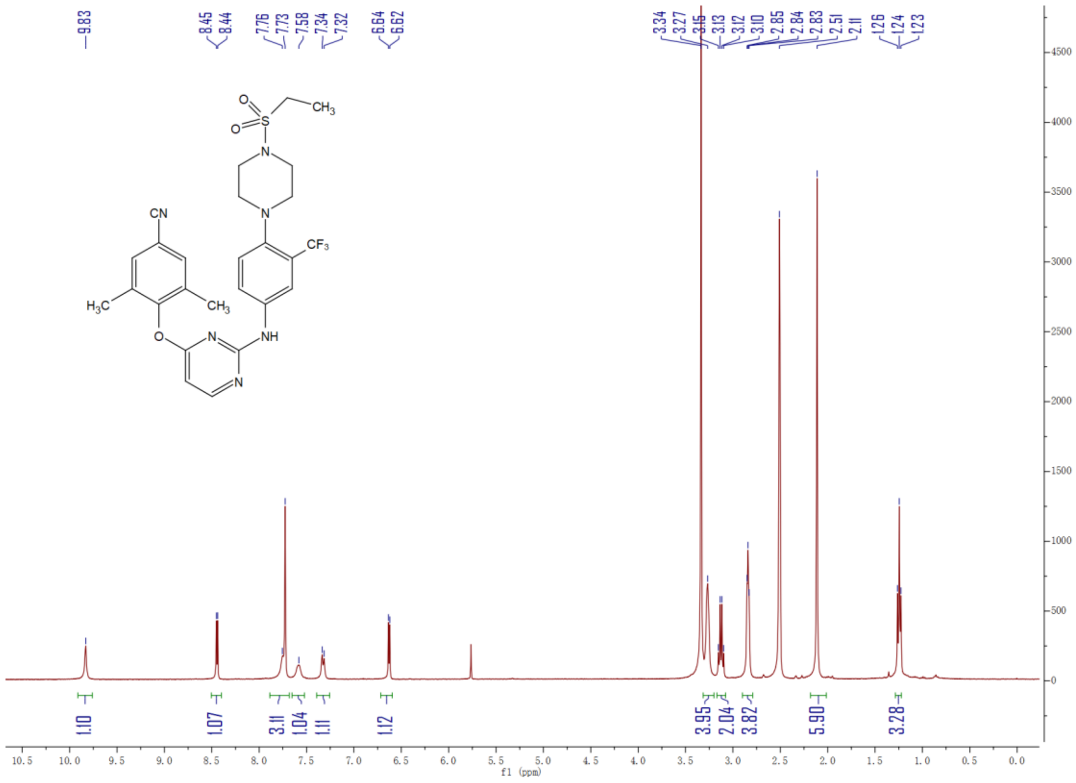

^{1}H NMR spectrum of **17c2**

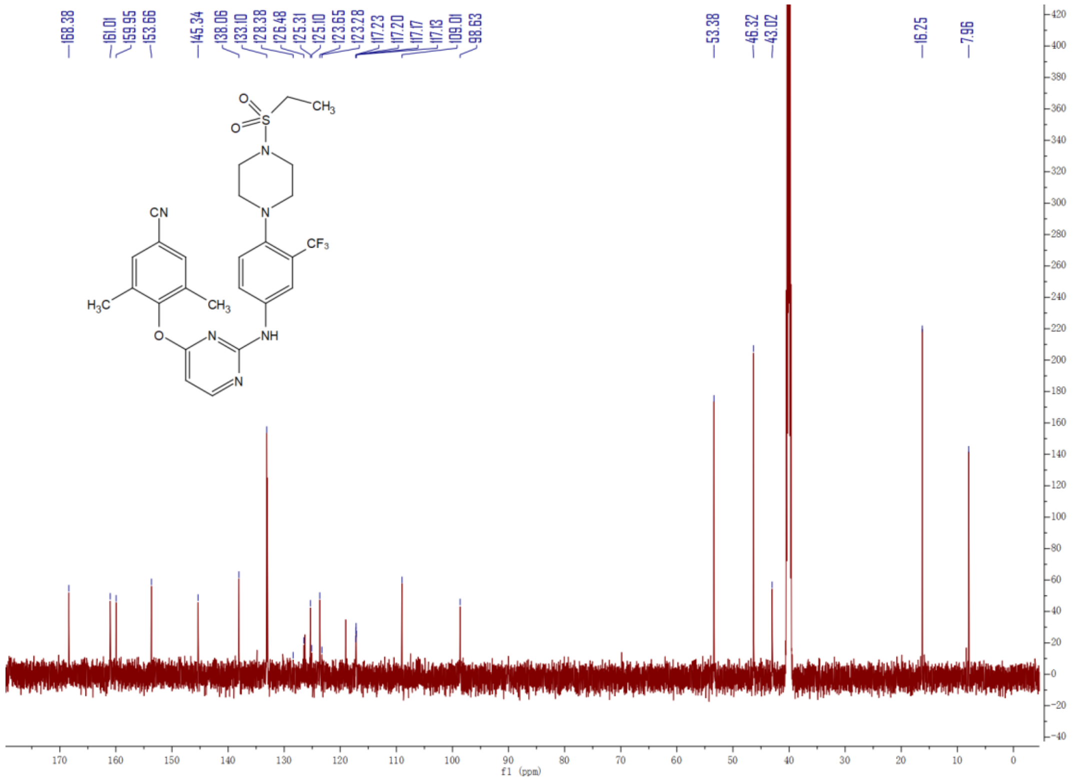

^{13}C NMR spectrum of **17c2**

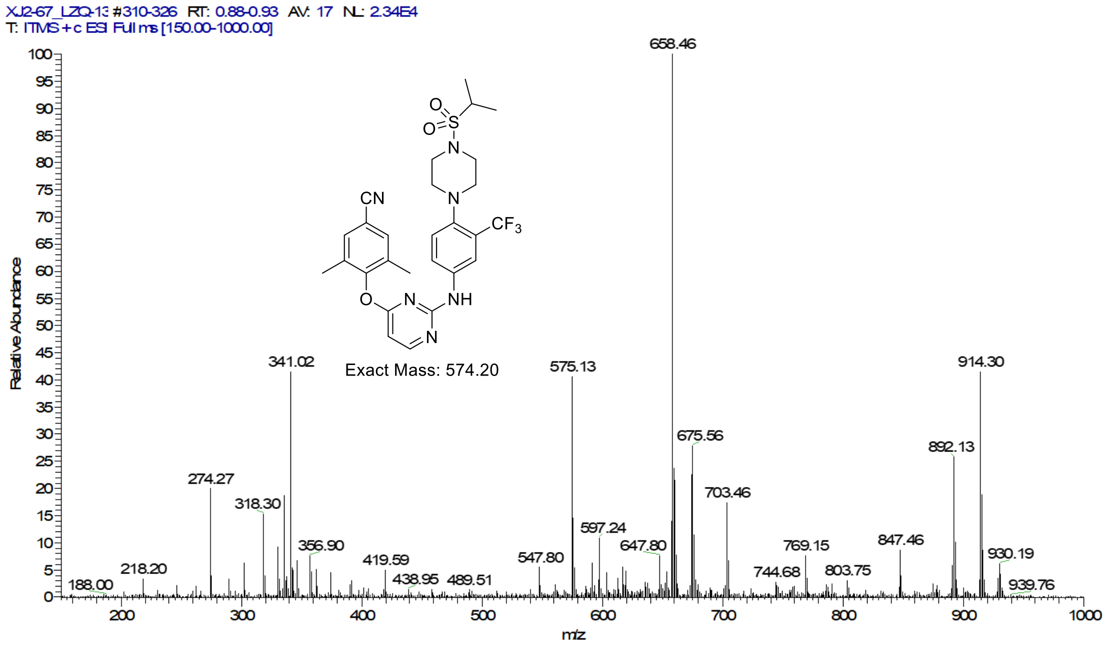

MS spectrum of **17c3**

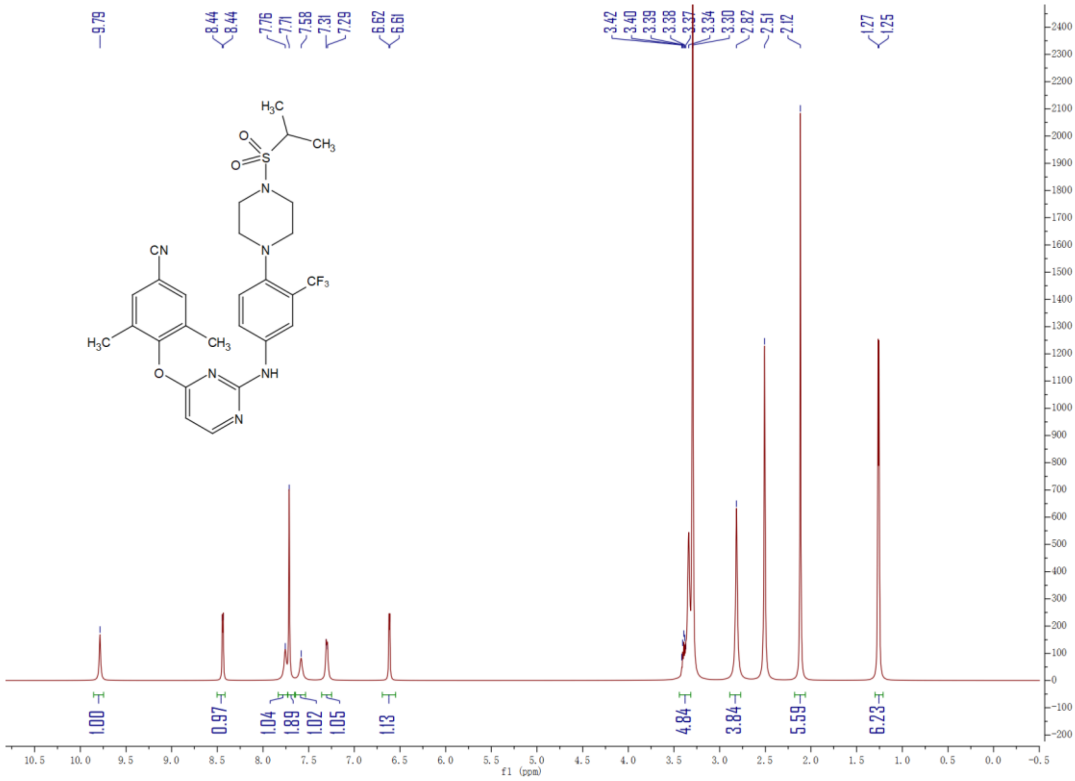

^{1}H NMR spectrum of **17c3**

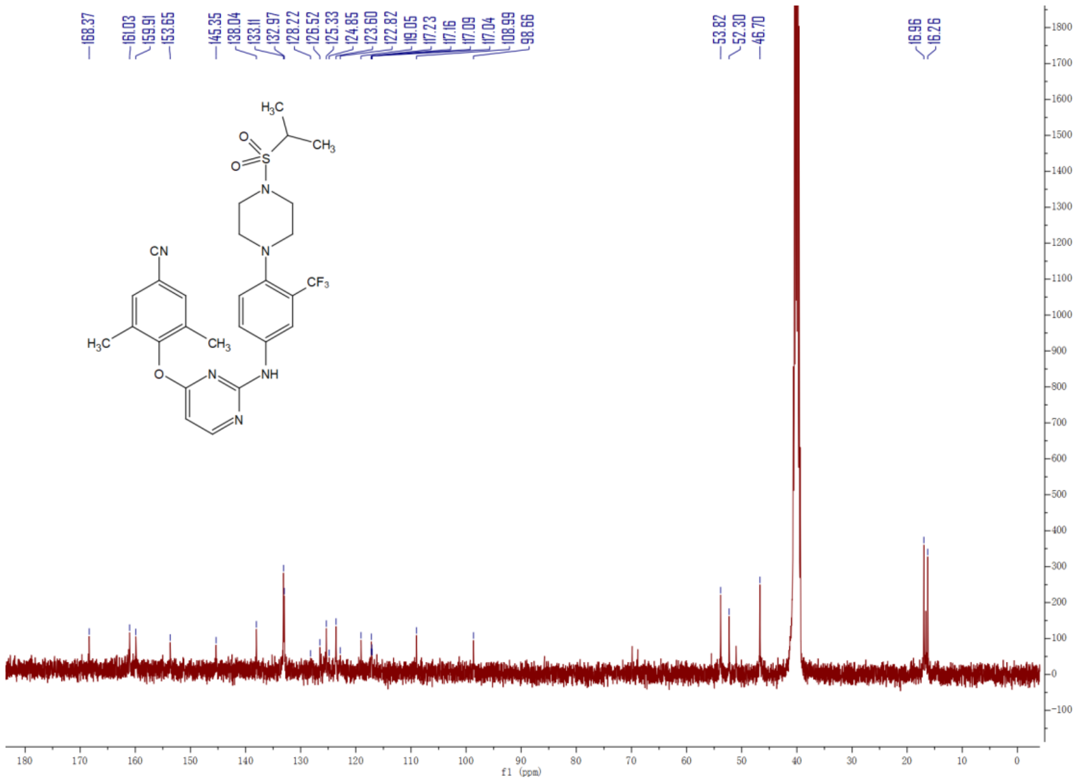

^{13}C NMR spectrum of **17c3**

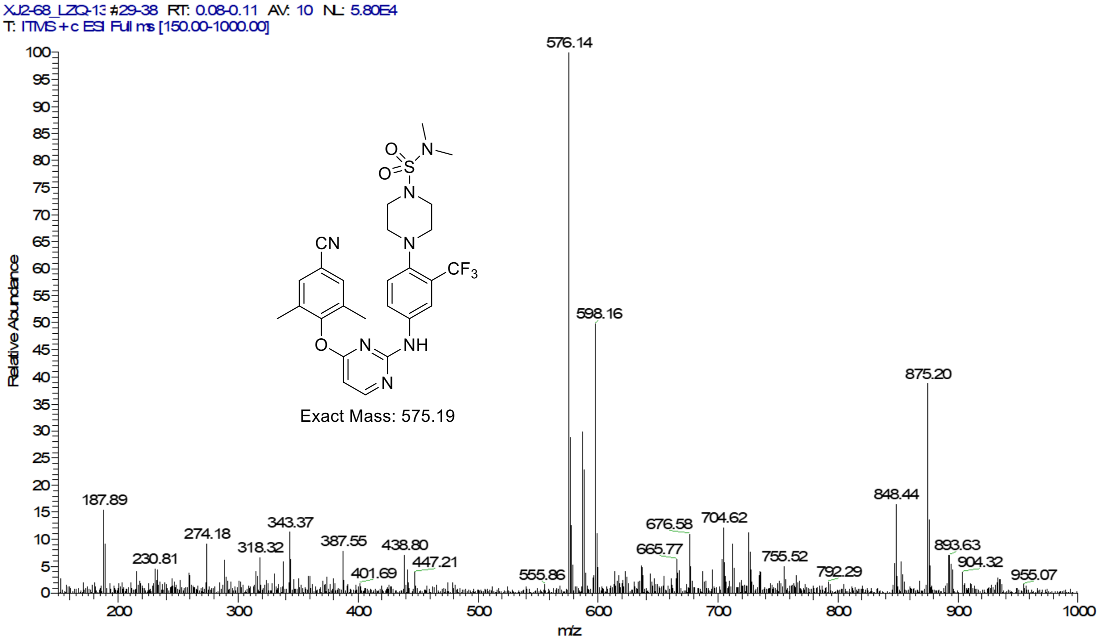

MS spectrum of **17c4**

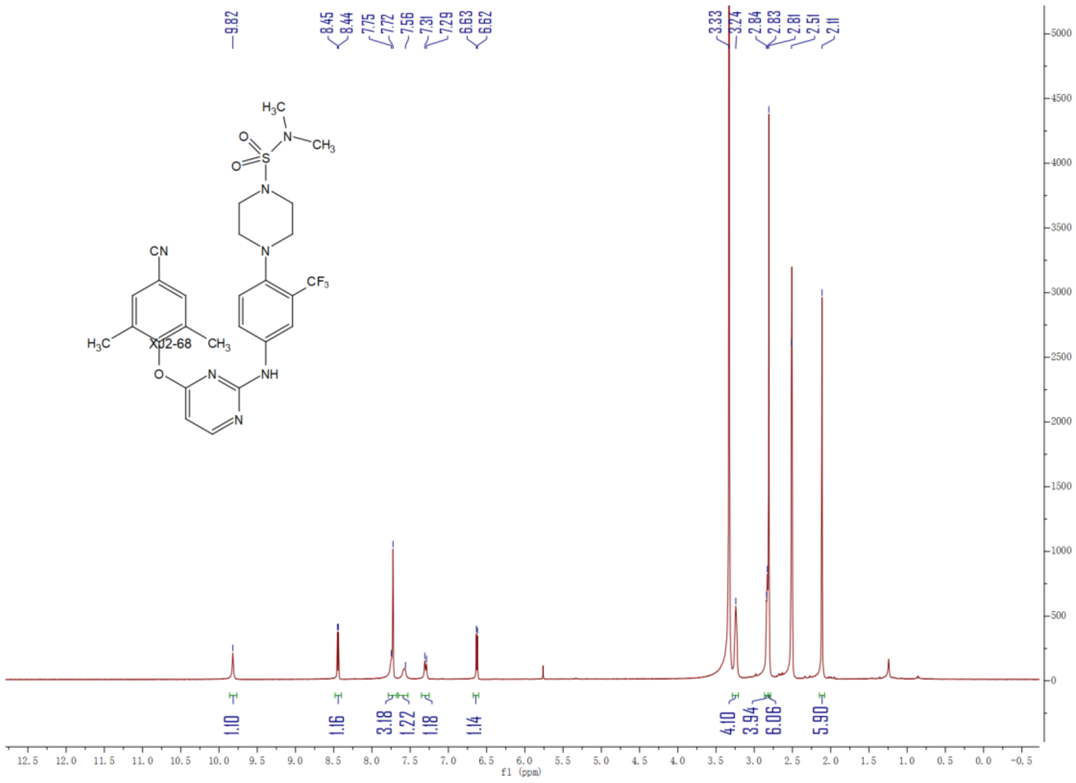

^{1}H NMR spectrum of **17c4**

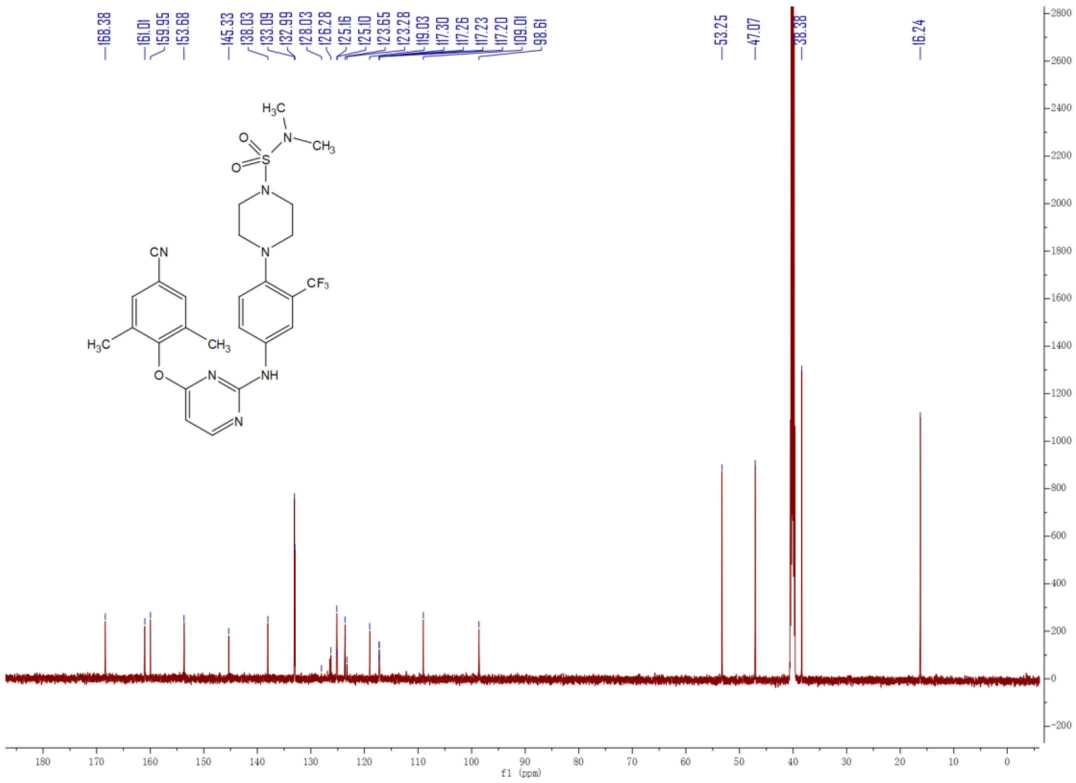

^{13}C NMR spectrum of **17c4**

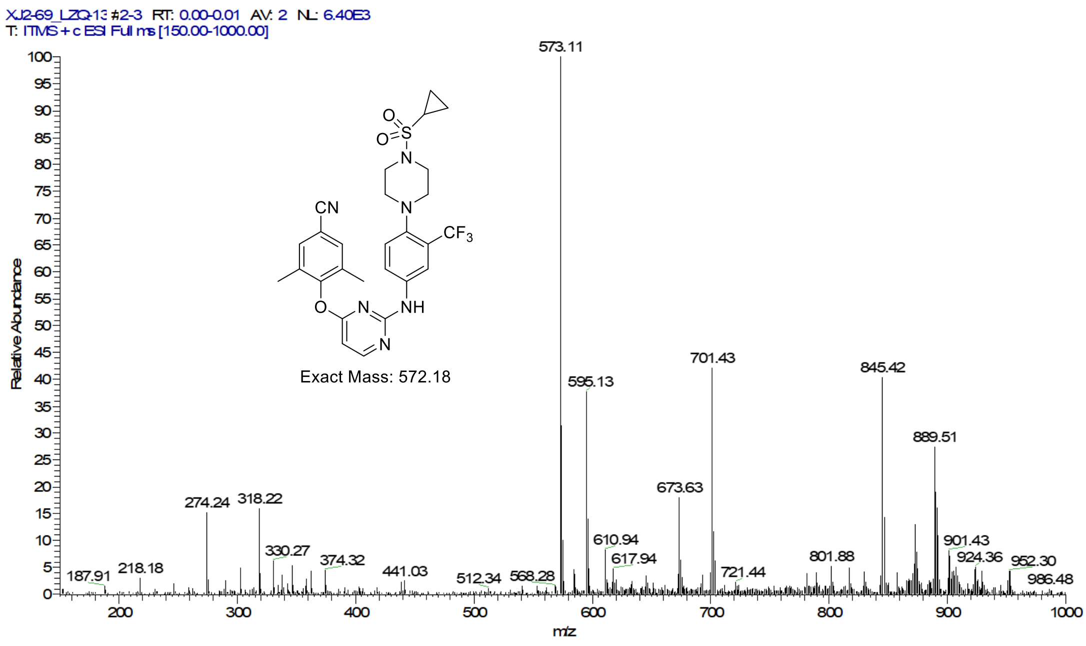

MS spectrum of **17c5**

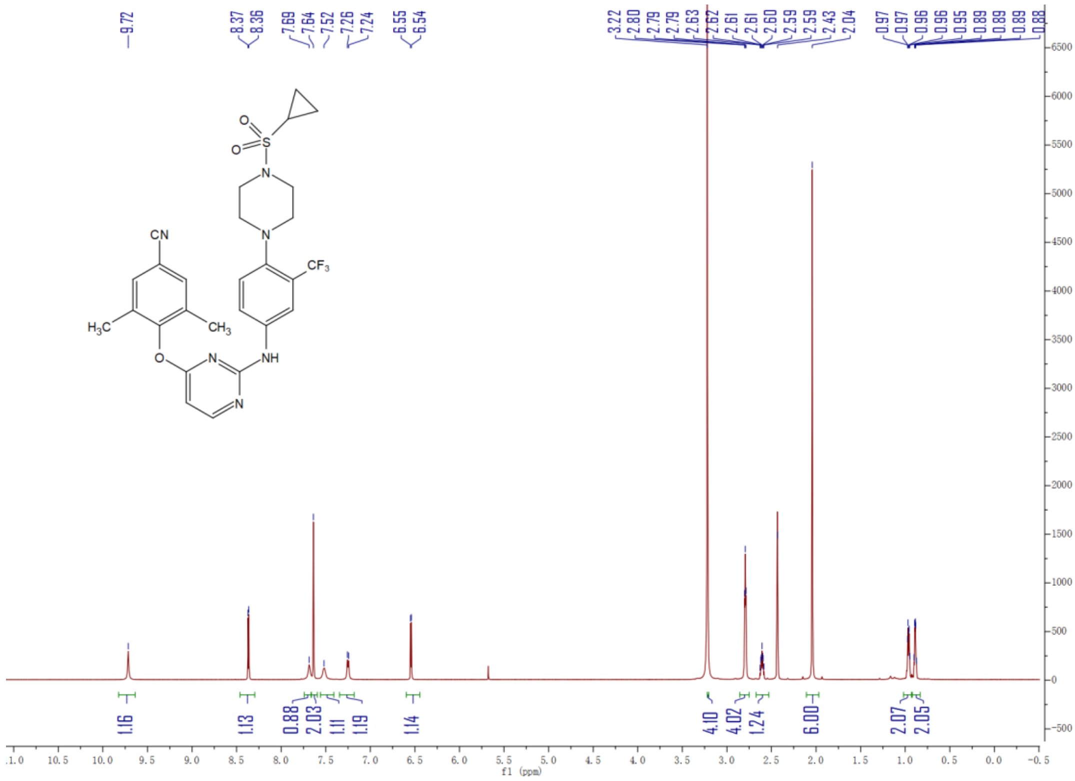

^{1}H NMR spectrum of **17c5**

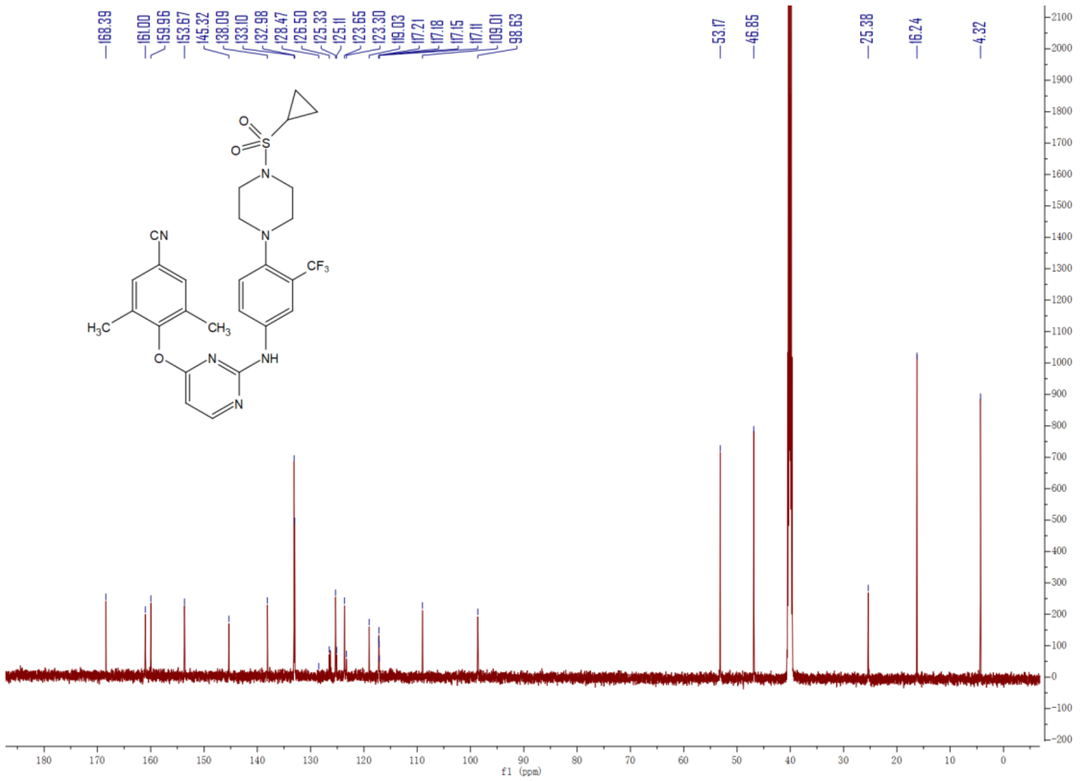

^{13}C NMR spectrum of **17c5**

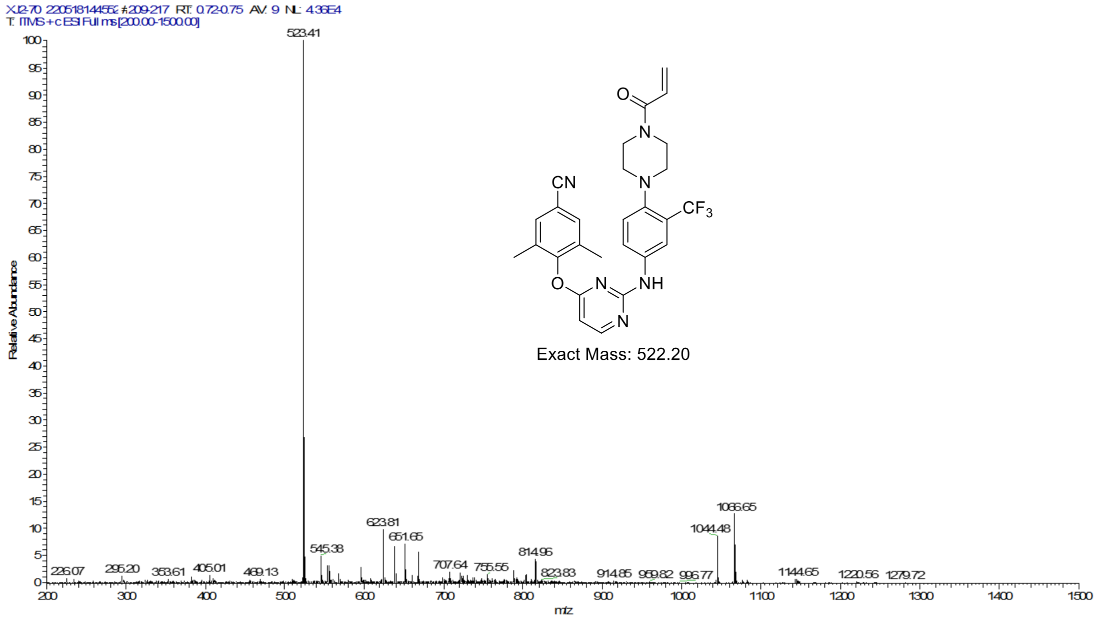

MS spectrum of **17c6**

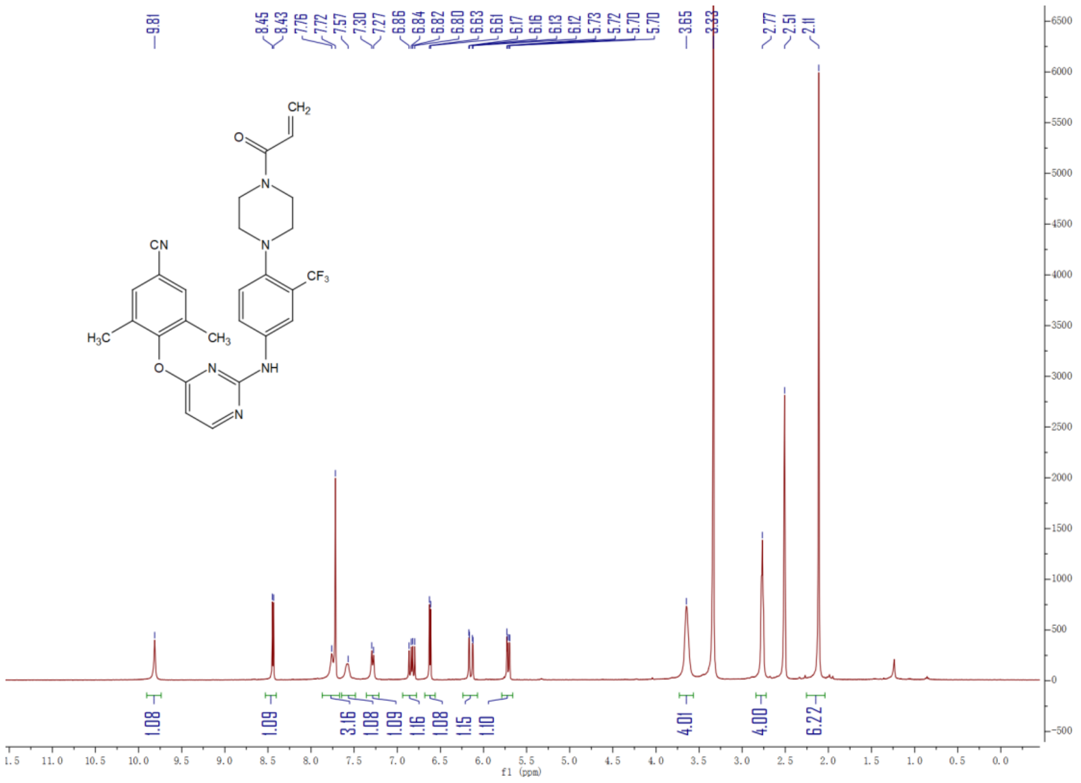

^{1}H NMR spectrum of **17c6**

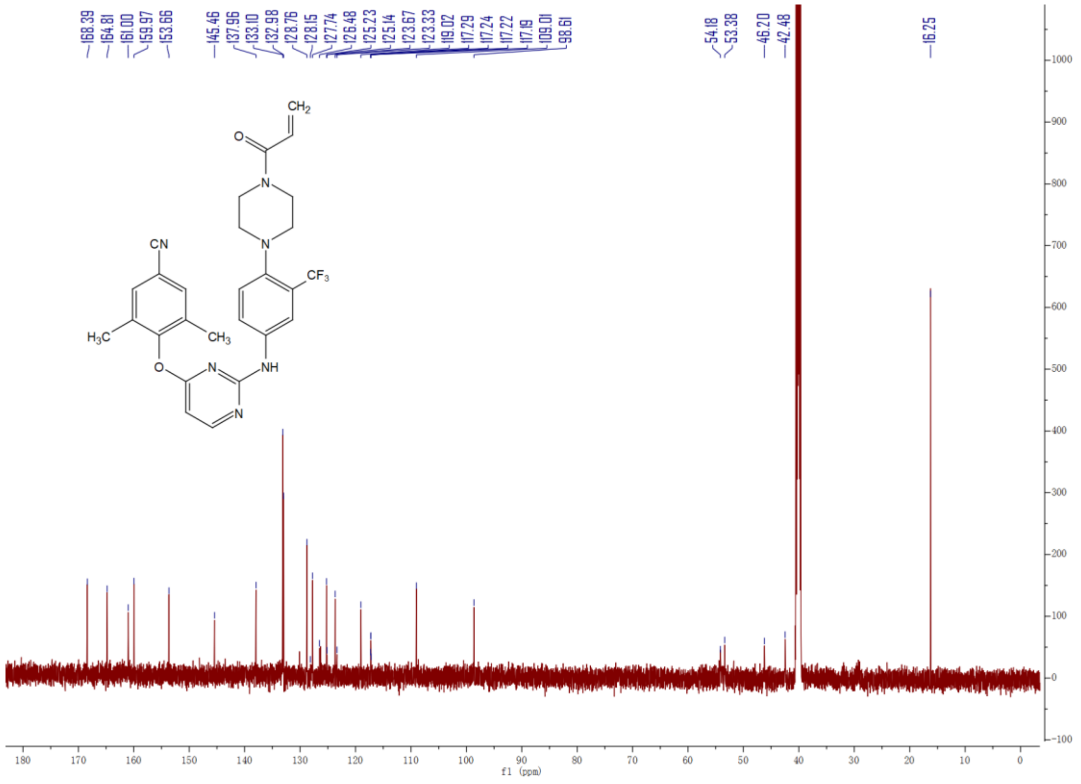

^{13}C NMR spectrum of **17c6**

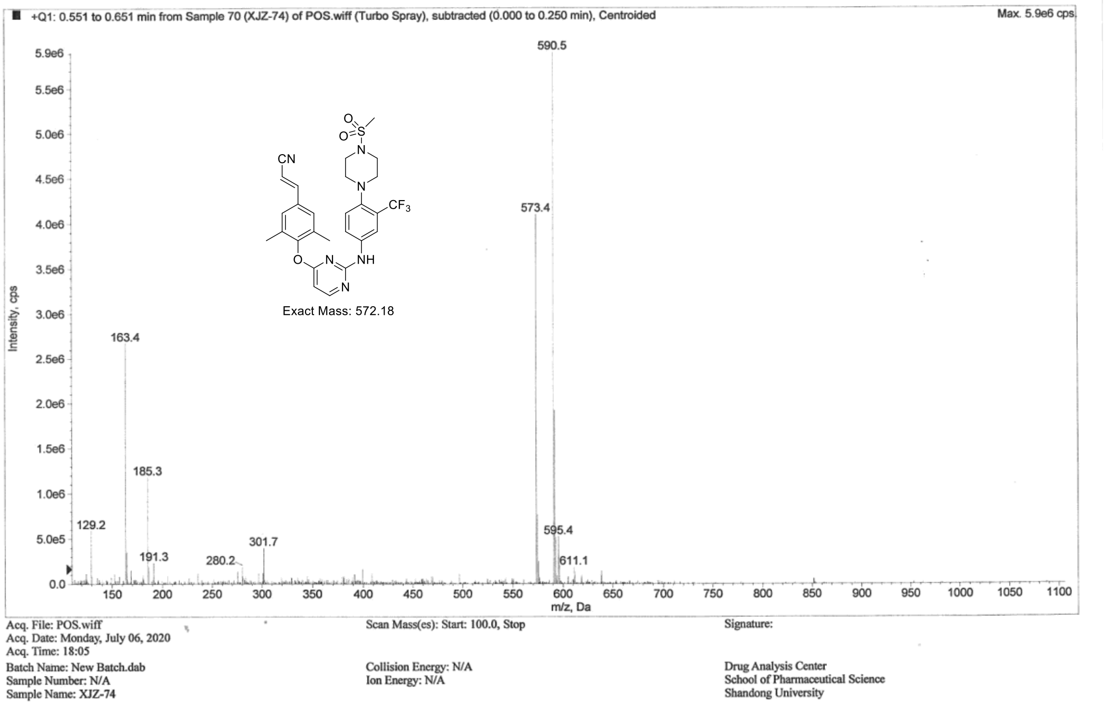

MS spectrum of **18c1**

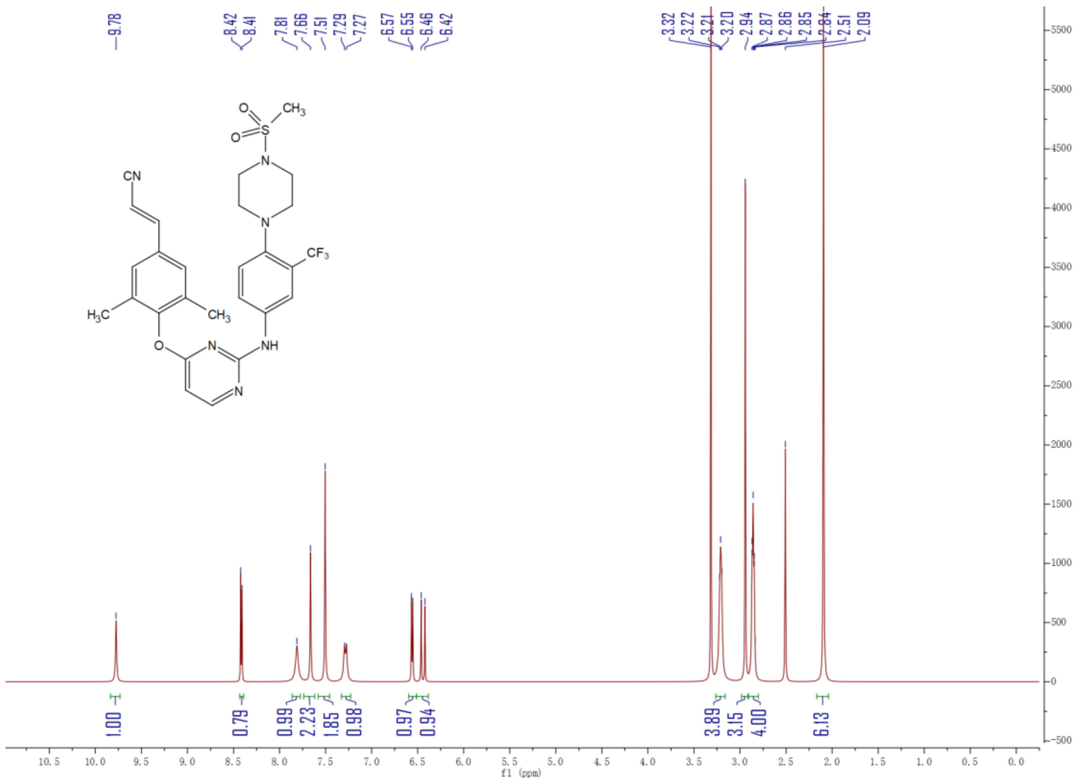

^{1}H NMR spectrum of **18c1**

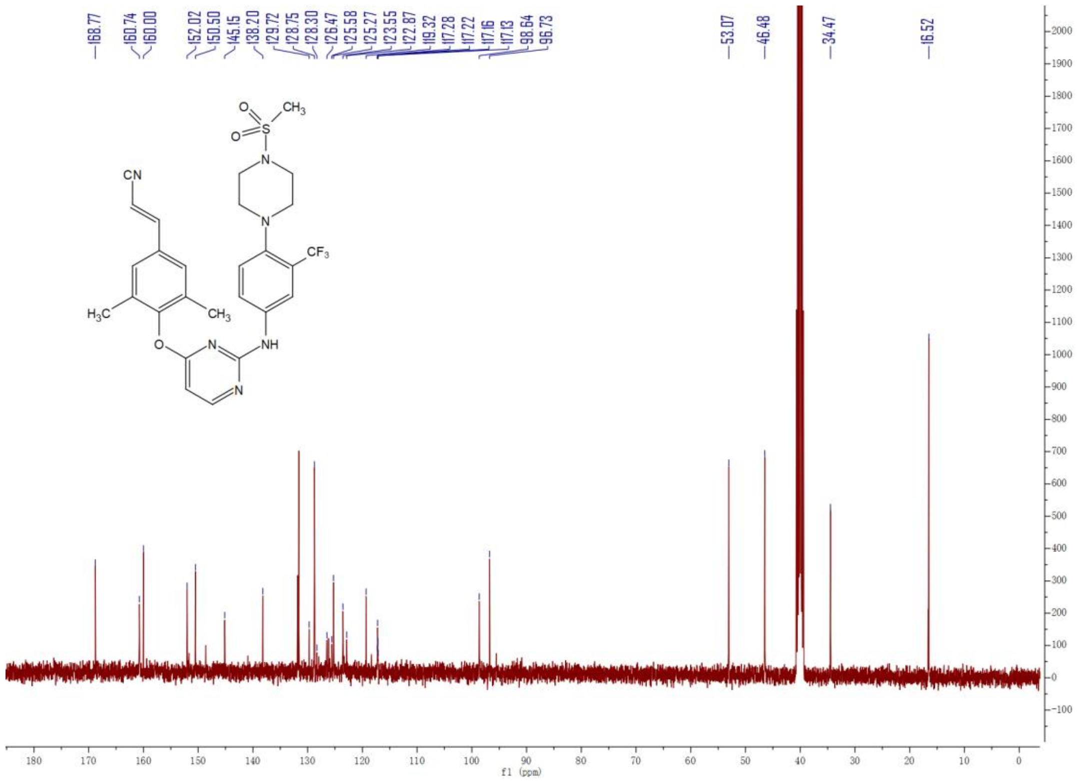

^{13}C NMR spectrum of **18c1**

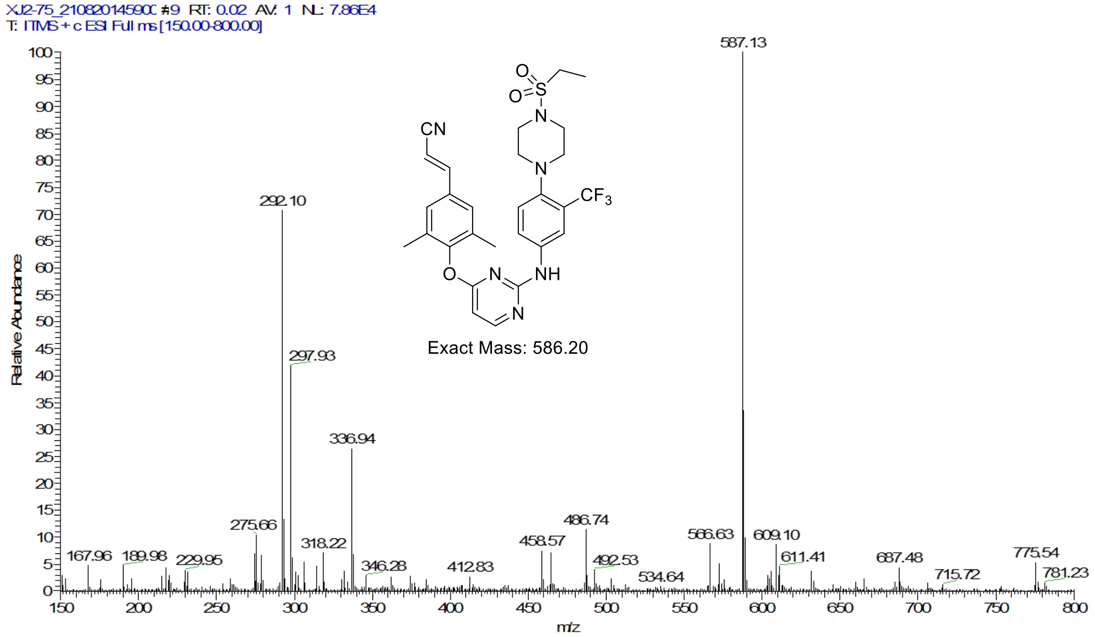

MS spectrum of **18c2**

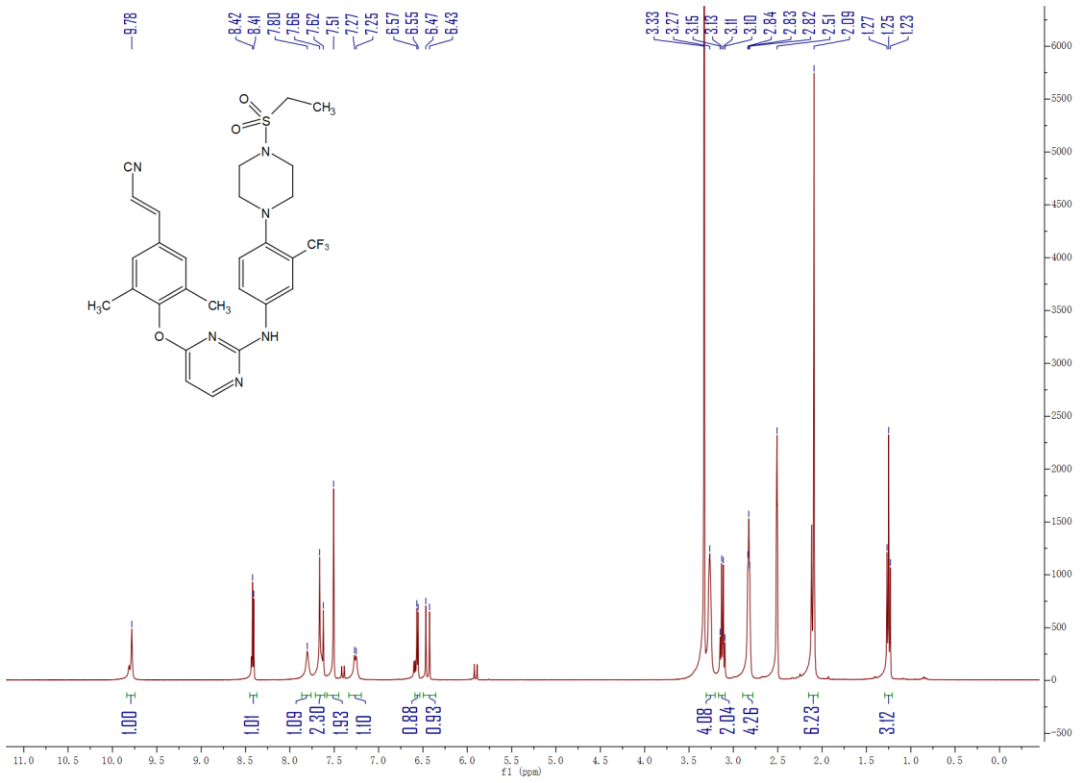

^{1}H NMR spectrum of **18c2**

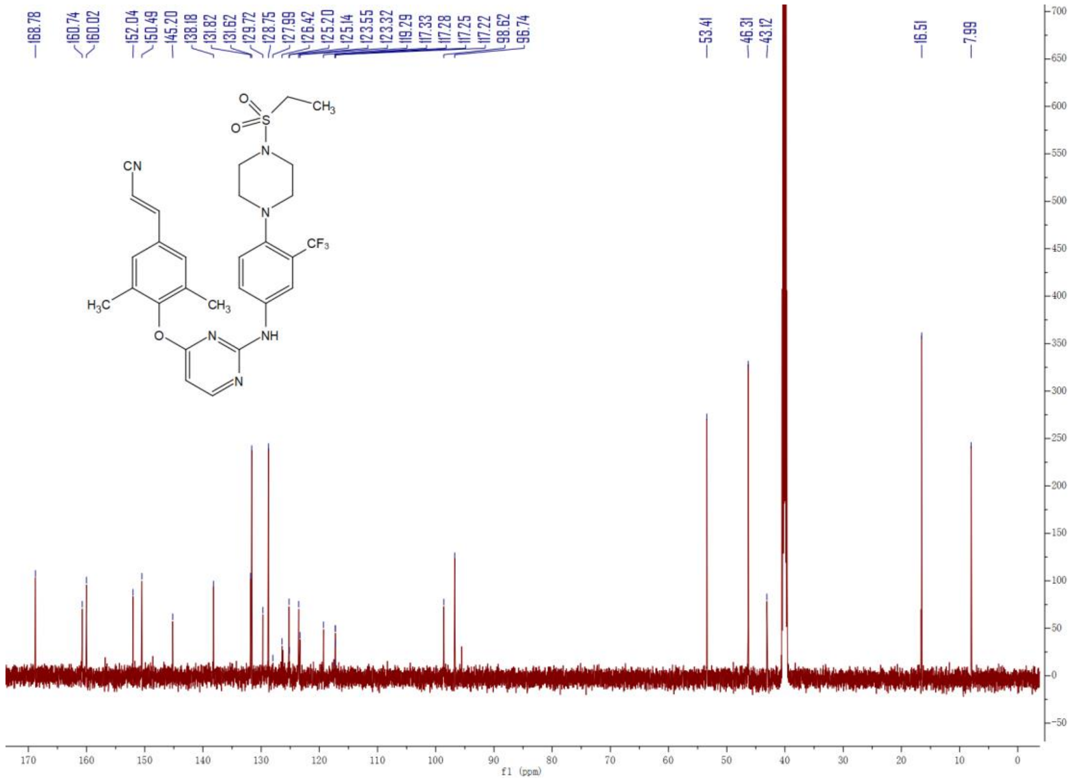

^{13}C NMR spectrum of **18c2**

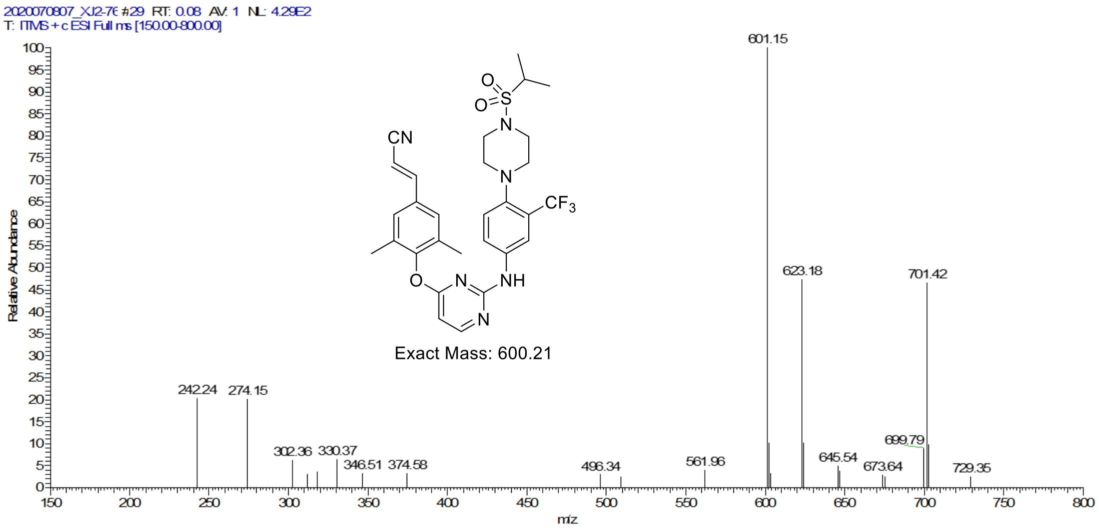

MS spectrum of **18c3**

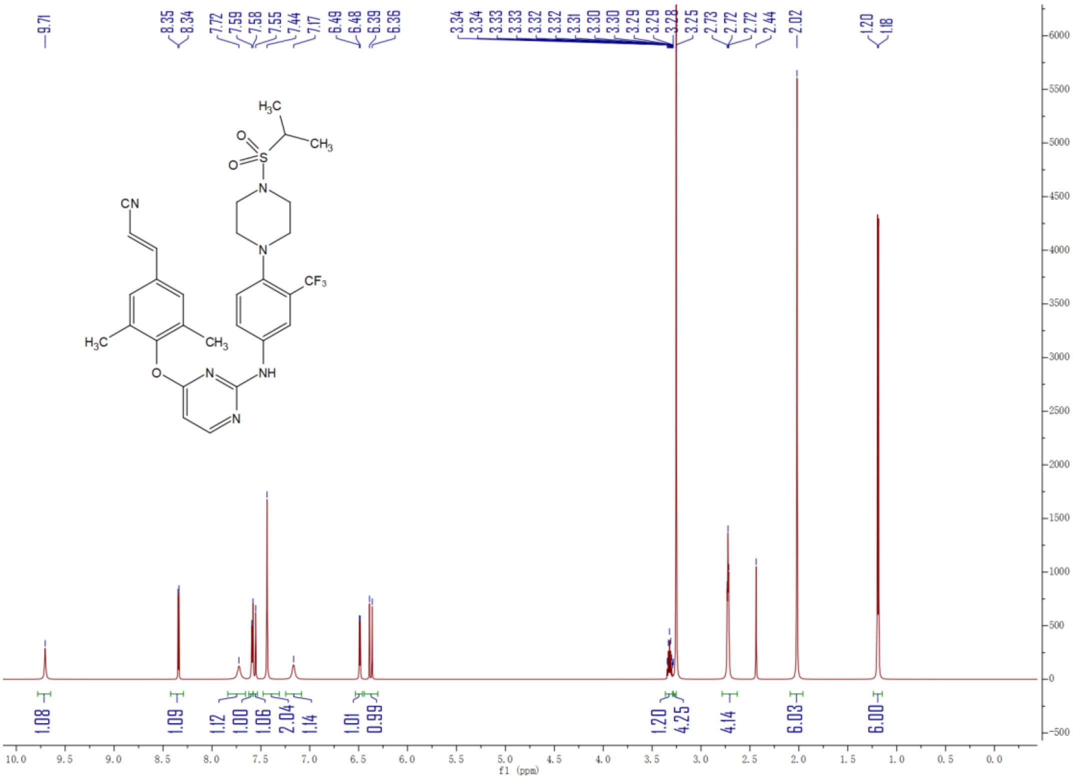

^{1}H NMR spectrum of **18c3**

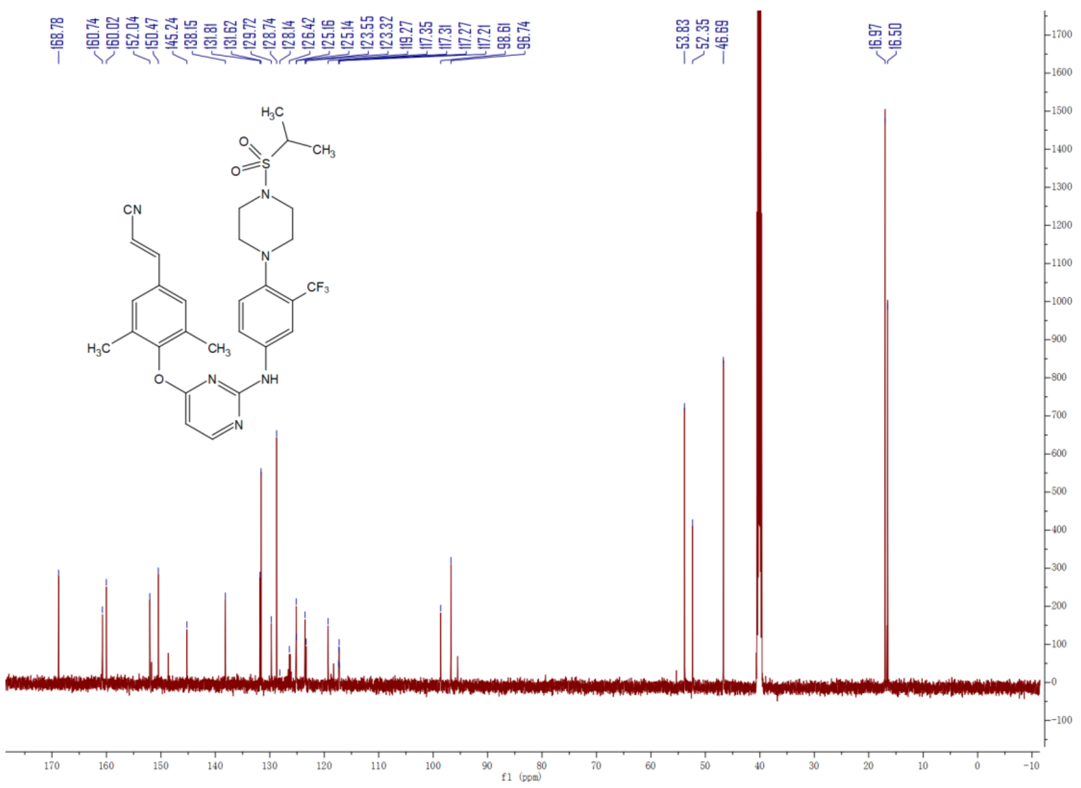

^{13}C NMR spectrum of **18c3**

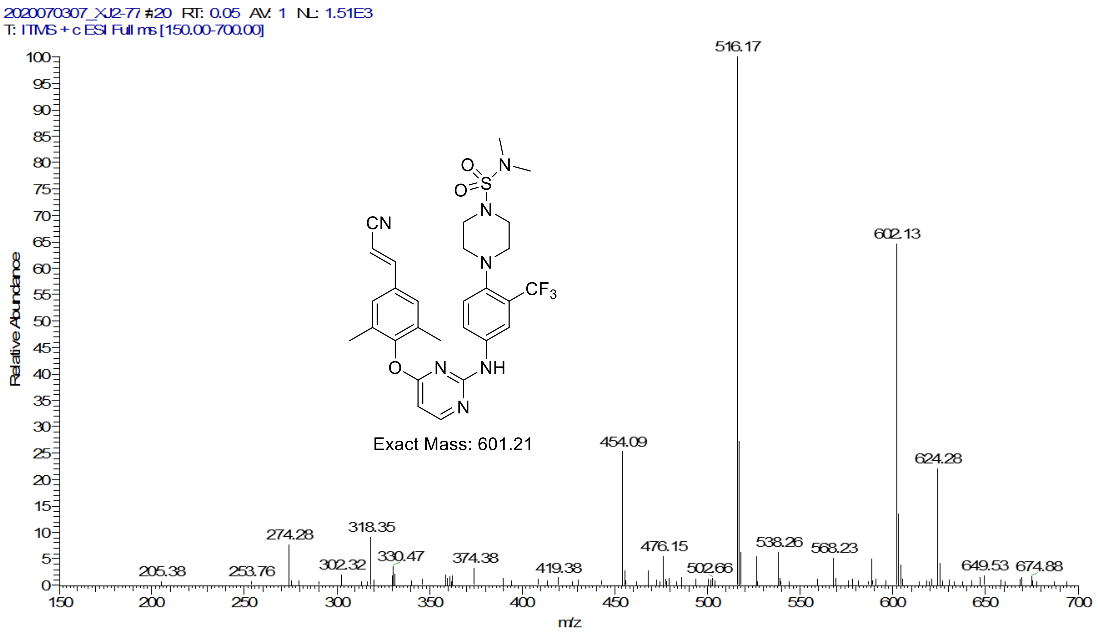

MS spectrum of **18c4**

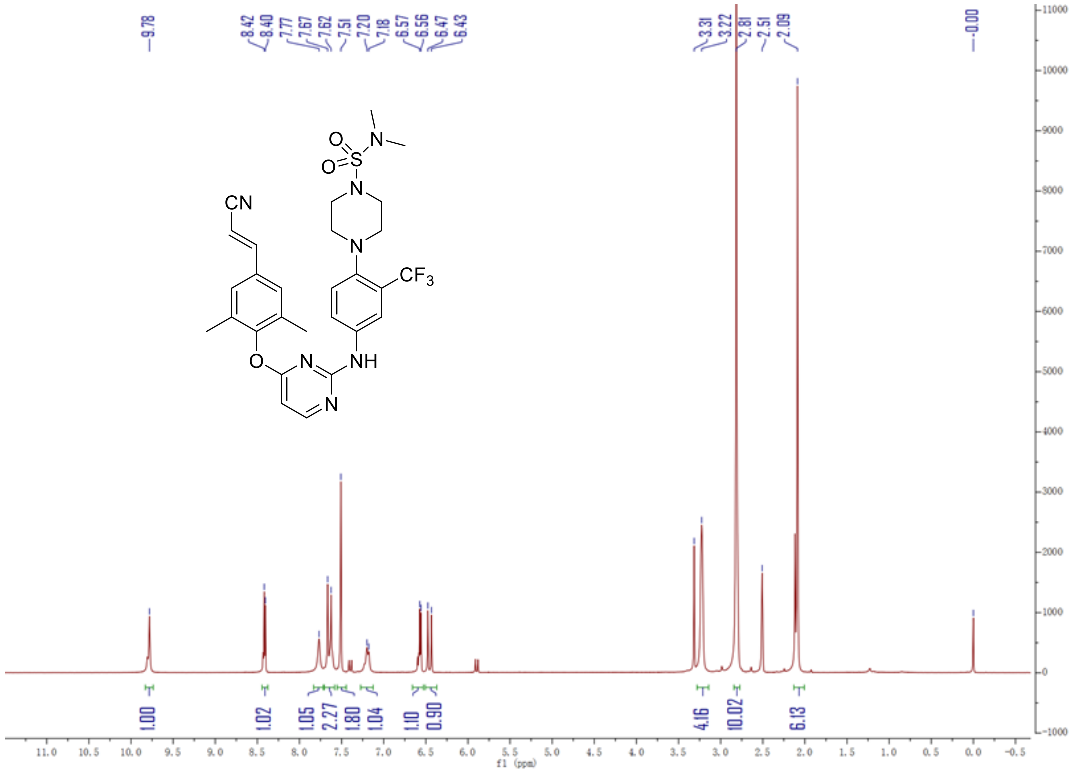

^{13}C NMR spectrum of **18c4**

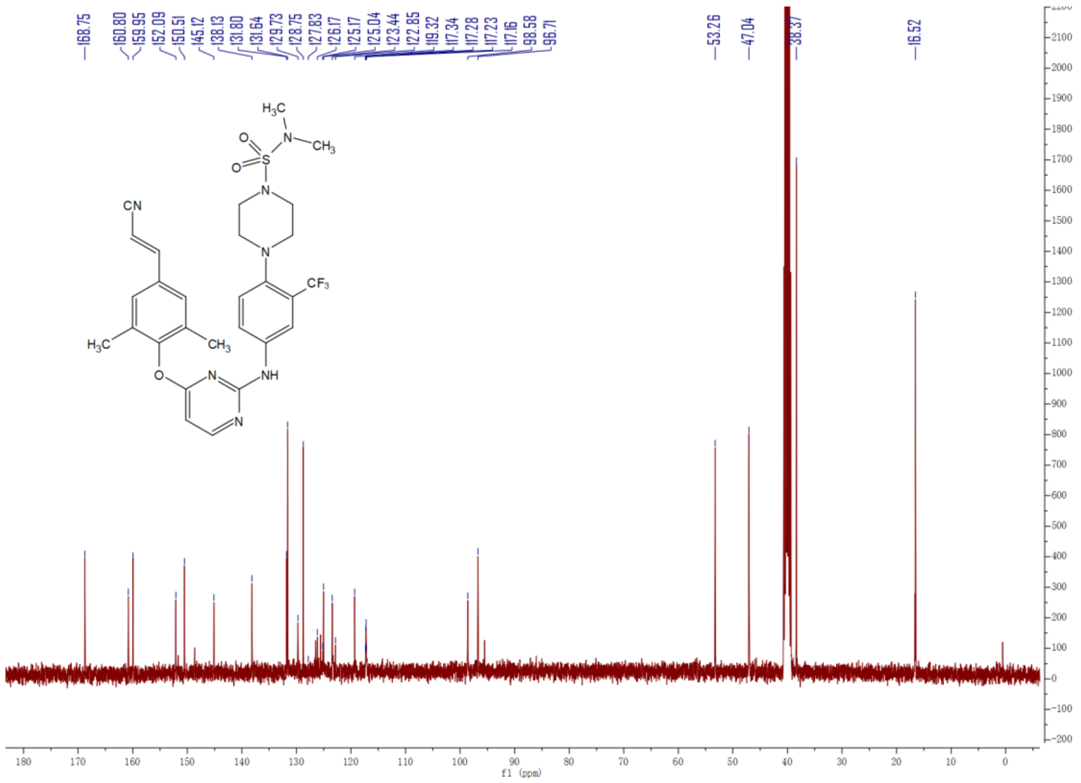

^{1}H NMR spectrum of **18c4**

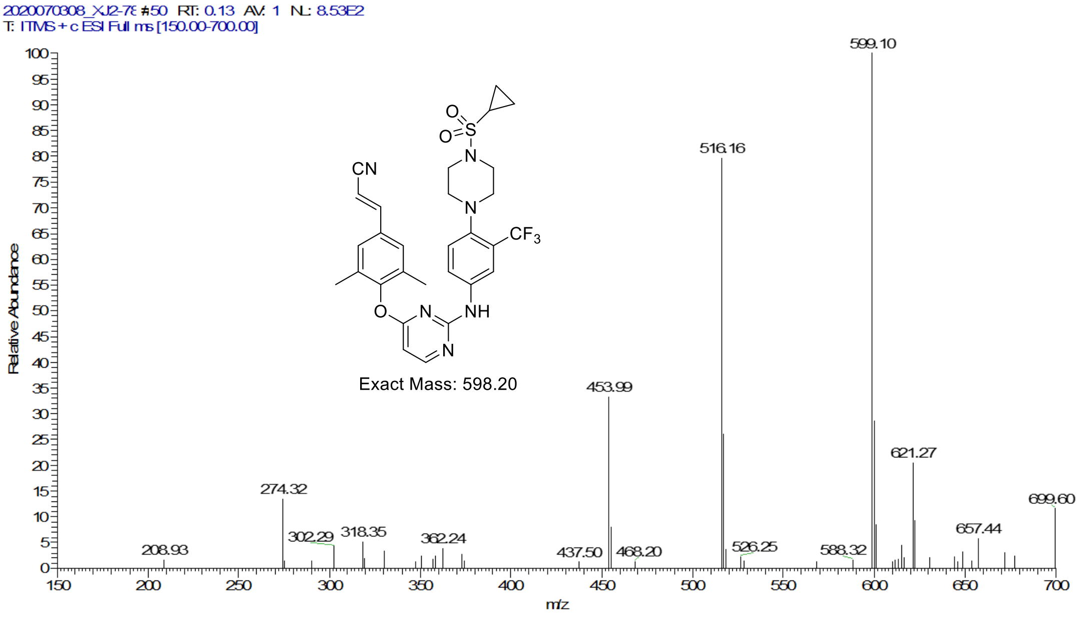

MS spectrum of **18c5**

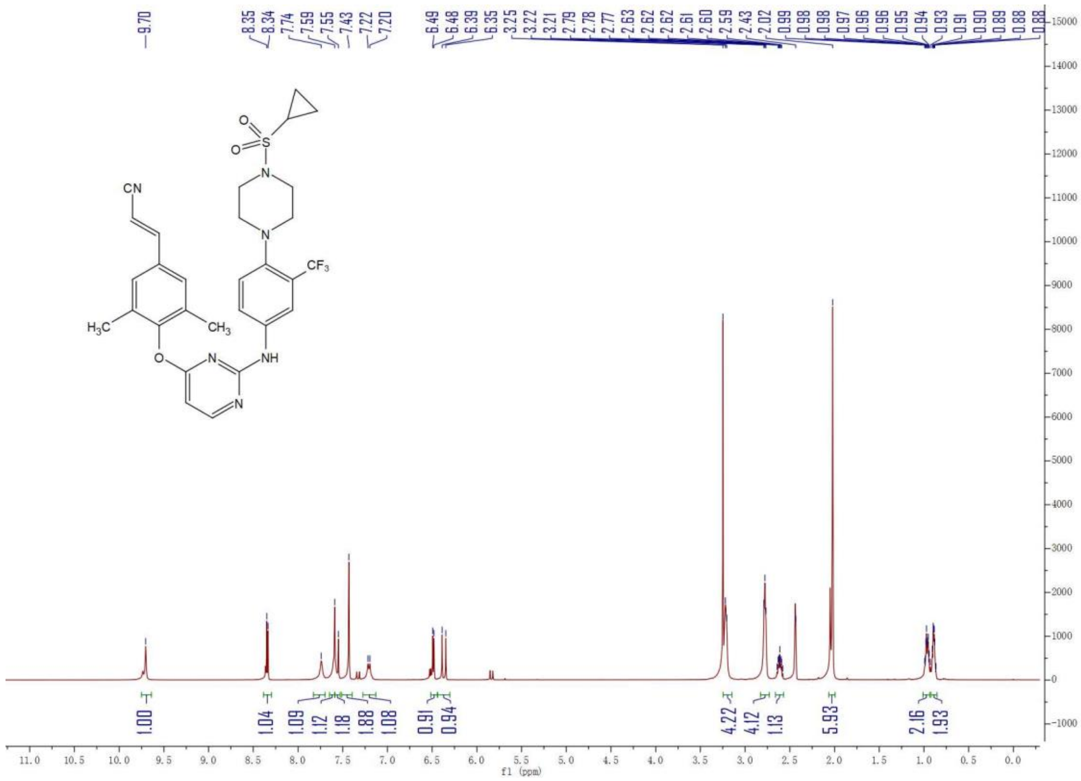

^{1}H NMR spectrum of **18c5**

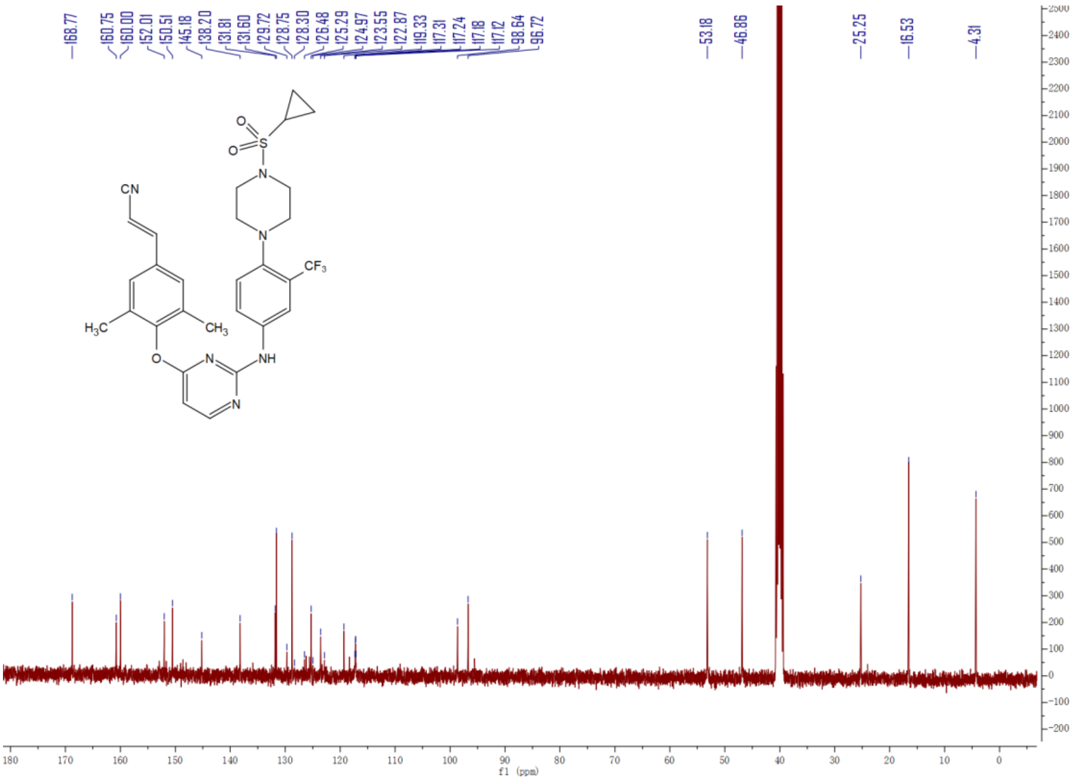

^{13}C NMR spectrum of **18c5**

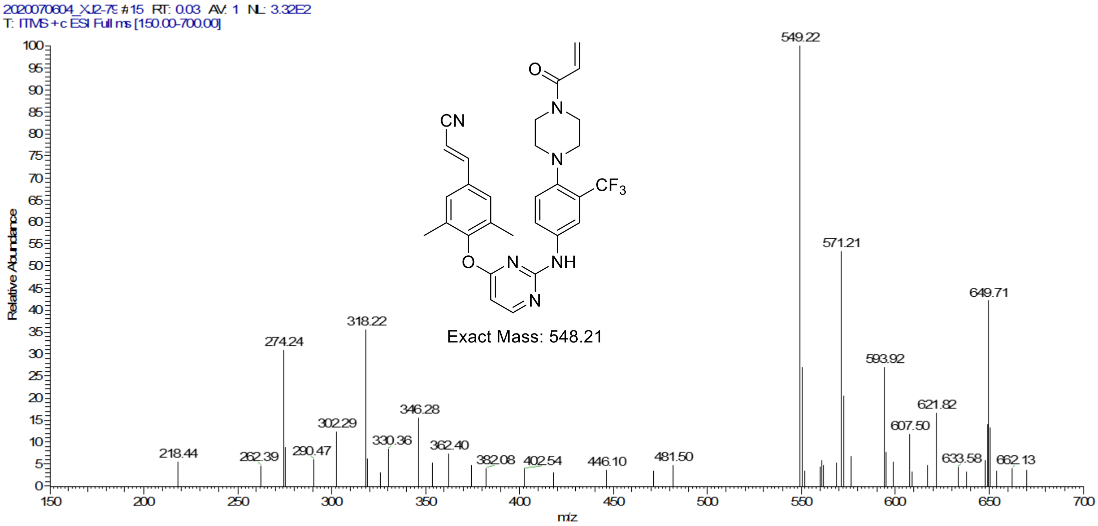

MS spectrum of **18c6**

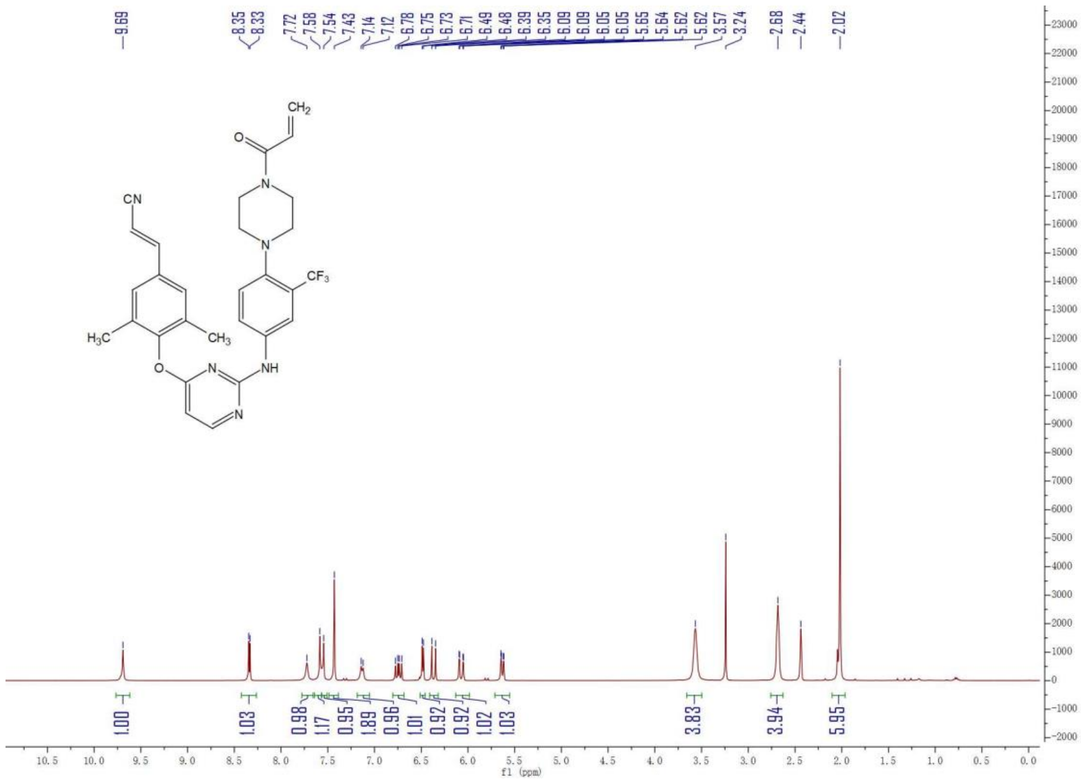

^{1}H NMR spectrum of **18c6**

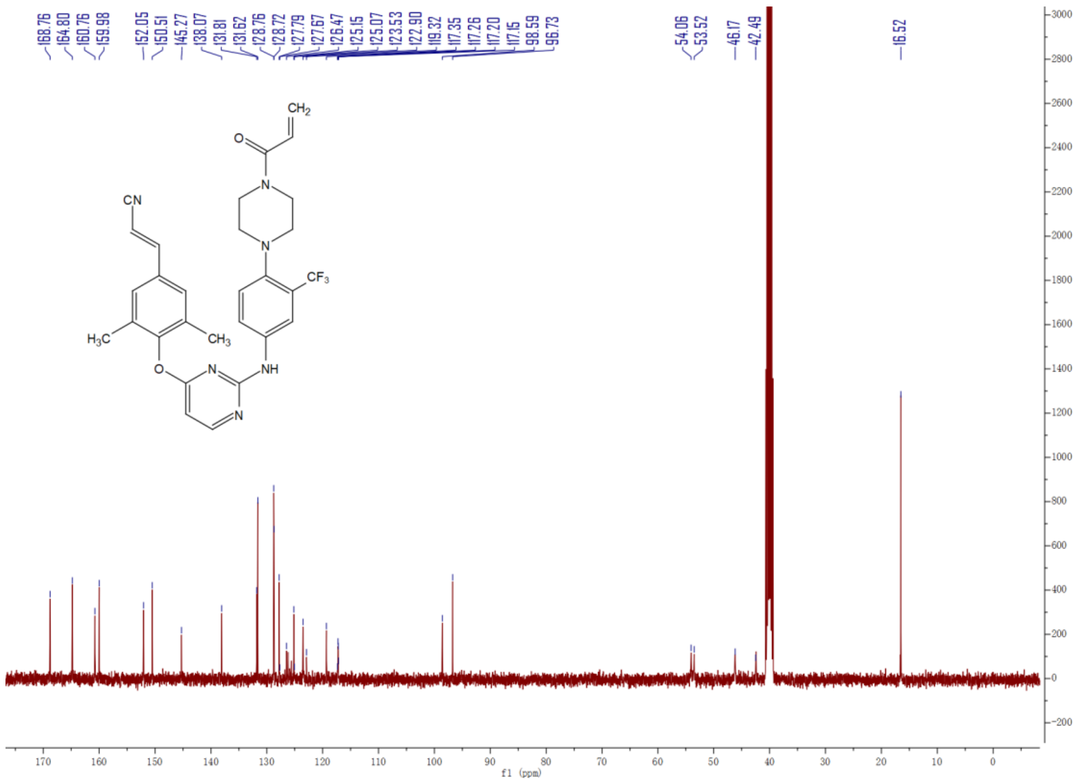

^{13}C NMR spectrum of **18c6**
