# Supplementary material for: Discovery of diarylpyrimidine derivatives bearing piperazine sulfonyl as potent HIV-1 nonnucleoside reverse transcriptase inhibitors
Source: Commun Chem. 2023 Apr 29;6:83. doi: 10.1038/s42004-023-00888-4 (PMC10148624; doi:10.1038/s42004-023-00888-4)
Supplement: Supplementary file 6 — Reporting Summary [file 42004_2023_888_MOESM6_ESM.pdf]

nature portfolio

Corresponding author(s): Peng Zhan

Last updated by author(s): Apr 16, 2023

Reporting Summary

Nature Portfolio wishes to improve the reproducibility of the work that we publish. This form provides structure for consistency and transparency in reporting. For further information on Nature Portfolio policies, see our Editorial Policies and the Editorial Policy Checklist.

Statistics

For all statistical analyses, confirm that the following items are present in the figure legend, table legend, main text, or Methods section.

n/a	Confirmed	
☐	☒	The exact sample size (*n*) for each experimental group/condition, given as a discrete number and unit of measurement
☐	☒	A statement on whether measurements were taken from distinct samples or whether the same sample was measured repeatedly
☐	☒	The statistical test(s) used AND whether they are one- or two-sided<br>*Only common tests should be described solely by name; describe more complex techniques in the Methods section.*
☐	☒	A description of all covariates tested
☒	☐	A description of any assumptions or corrections, such as tests of normality and adjustment for multiple comparisons
☐	☒	A full description of the statistical parameters including central tendency (e.g. means) or other basic estimates (e.g. regression coefficient) AND variation (e.g. standard deviation) or associated estimates of uncertainty (e.g. confidence intervals)
☒	☐	For null hypothesis testing, the test statistic (e.g. *F*, *t*, *r*) with confidence intervals, effect sizes, degrees of freedom and *P* value noted<br>*Give P values as exact values whenever suitable.*
☒	☐	For Bayesian analysis, information on the choice of priors and Markov chain Monte Carlo settings
☒	☐	For hierarchical and complex designs, identification of the appropriate level for tests and full reporting of outcomes
☐	☒	Estimates of effect sizes (e.g. Cohen's *d*, Pearson's *r*), indicating how they were calculated

Our web collection on statistics for biologists contains articles on many of the points above.

Software and code

Policy information about availability of computer code

Data collection	Surflex-Dock SYBYL-X 2.0; OpenEye Scientific Software.
Data analysis	PyMOL (http://www.pymol.org/); PDBsum (http://www.ebi.ac.uk/thornton-srv/databases/cgi-bin/pdbsum/GetPage.pl?pdbcode=index.html)

For manuscripts utilizing custom algorithms or software that are central to the research but not yet described in published literature, software must be made available to editors and reviewers. We strongly encourage code deposition in a community repository (e.g. GitHub). See the Nature Portfolio guidelines for submitting code & software for further information.

Data

Policy information about availability of data

All manuscripts must include a data availability statement. This statement should provide the following information, where applicable:

- Accession codes, unique identifiers, or web links for publicly available datasets
- A description of any restrictions on data availability
- For clinical datasets or third party data, please ensure that the statement adheres to our policy

The authors declare that the data supporting the findings of this study are available within the paper and its supplementary information, supplementary data 1 and supplementary data 2.
PDB accession code: crystal structure of HIV-1 RT in complex with the non-nucleoside inhibitor 18b1, PDB entry: 8FE8.

Research involving human participants, their data, or biological material

Policy information about studies with human participants or human data. See also policy information about sex, gender (identity/presentation), and sexual orientation and race, ethnicity and racism.

Reporting on sex and gender	The study did not involve human participants
Reporting on race, ethnicity, or other socially relevant groupings	The study did not involve human participants
Population characteristics	The study did not involve human participants
Recruitment	The study did not involve human participants
Ethics oversight	The study did not involve human participants

Note that full information on the approval of the study protocol must also be provided in the manuscript.

Field-specific reporting

Please select the one below that is the best fit for your research. If you are not sure, read the appropriate sections before making your selection.

☒ Life sciences ☐ Behavioural & social sciences ☐ Ecological, evolutionary & environmental sciences

For a reference copy of the document with all sections, see nature.com/documents/nr-reporting-summary-flat.pdf

Life sciences study design

All studies must disclose on these points even when the disclosure is negative.

Sample size	In vitro anti-HIV assays: n≥4; HIV-1 RT inhibition assays: n=2; Water solubility measurements: n=2; Pharmacokinetics studies: n=3; Sample size was determined to ensure the accuracy of the data
Data exclusions	No data were excluded from the analyses
Replication	All attempts at replication were successful
Randomization	The allocation was random
Blinding	The investigators were not blinded to group allocation during datacollection and analysis

Reporting for specific materials, systems and methods

We require information from authors about some types of materials, experimental systems and methods used in many studies. Here, indicate whether each material, system or method listed is relevant to your study. If you are not sure if a list item applies to your research, read the appropriate section before selecting a response.

Materials & experimental systems

n/a	Involved in the study	
☒	☐	Antibodies
☐	☒	Eukaryotic cell lines
☒	☐	Palaeontology and archaeology
☐	☒	Animals and other organisms
☒	☐	Clinical data
☒	☐	Dual use research of concern
☒	☐	Plants

Methods

n/a	Involved in the study	
☒	☐	ChIP-seq
☒	☐	Flow cytometry
☒	☐	MRI-based neuroimaging

Eukaryotic cell lines

Policy information about cell lines and Sex and Gender in Research

Cell line source(s)	An adult T-cell leukemia (ATL) male patient with cord blood leukocytes(Nature, 1981, 294, 770-771.)

Cell line source(s)	Rega Institute for Medical Research, Laboratory of Virology and Chemotherapy, K.U.Leuven, Belgium
Authentication	None of the cell lines used were authenticated
Mycoplasma contamination	The cell lines were not tested form ycoplasma contamination .
Commonly misidentified lines (See ICLAC register)	No commonly misidentified lines

Animals and other research organisms

Policy information about studies involving animals; ARRIVE guidelines recommended for reporting animal research, and Sex and Gender in Research

Laboratory animals	6-8 week-old Sprague-Dawley rats
Wild animals	The study did not involve wild animals
Reporting on sex	male rats. Sex is usually not considered in pharmacokinetic studies
Field-collected samples	The study did not involve samples collected from the field
Ethics oversight	All animal treatments were performed strictly in accordance with the institutional guidelines of Animal Care and Use Committee at Shandong University, after gaining approval from the Animal Ethical and Welfare Committee (AEWC).

Note that full information on the approval of the study protocol must also be provided in the manuscript.
